# Synthesis, Electronic Properties and OLED Devices of Chromophores Based on λ^5^‐Phosphinines

**DOI:** 10.1002/chem.202000932

**Published:** 2020-08-06

**Authors:** Gregor Pfeifer, Faouzi Chahdoura, Martin Papke, Manuela Weber, Rózsa Szűcs, Bernard Geffroy, Denis Tondelier, László Nyulászi, Muriel Hissler, Christian Müller

**Affiliations:** ^1^ Institut für Chemie und Biochemie Freie Universität Berlin Fabeckstrasse 34/36 14195 Berlin Germany; ^2^ ISCR—UMR CNRS 6226 Univ Rennes 35000 Rennes France; ^3^ Department of Inorganic and Analytical Chemistry and MTA-BME Computation Driven Chemistry Research Group Budapest University of Technology and Economics Szt. Gellért tér 4 1111 Budapest Hungary; ^4^ LICSEN, NIMBE, CEA, CNRS Université Paris-Saclay, CEA Saclay Gif-sur-Yvette CEDEX 91191 France

**Keywords:** DFT calculations, heterocycles, OLEDs, phosphinines, pi systems

## Abstract

A new series of 2,4,6‐triaryl‐λ^5^‐phosphinines have been synthesized that contain different substituents both on the carbon backbone and the phosphorus atom of the six‐membered heterocycle. Their optical and redox properties were studied in detail, supported by in‐depth theoretical calculations. The modularity of the synthetic strategy allowed the establishment of structure–property relationships for this class of compounds and an OLED based on a blue phosphinine emitter could be developed for the first time.

## Graphical abstract

**A new series of 2,4,6‐triaryl‐λ^5^‐phosphinines** were synthesized and characterized. Supported by DFT calculations, the structure–property relationships of these unsaturated phosphorus heterocycles were investigated in detail. For the first time, an organic light‐emitting device was developed based on a blue phosphinine emitter.

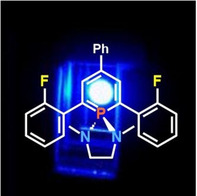

## Introduction

**Figure 1:**
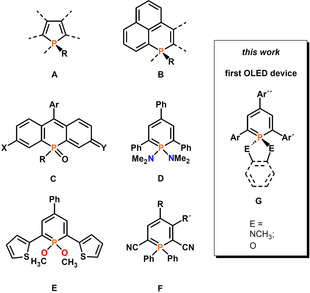
Fluorescent organophosphorus compounds **A**–**F** and schematic structure of a λ^5^‐phosphinine **G**. Ar, Ar′and Ar′′: substituted aryl‐groups.

During the last decades, phosphorus(III) heterocycles have evolved to important key structures in modern chemical research, especially concerning applications. In the field of homogeneous catalysis, for instance, transition‐metal complexes based on saturated and unsaturated 5‐ and 6‐membered phosphorus heterocycles show excellent performance.^1^ Chiral 5‐membered phospholanes are among the most efficient ligands for asymmetric homogeneous catalysis.^2^ In molecular material science, the most widely used P‐based building block is the phosphole ring (**A**, Figure 1) embedded in π‐conjugated systems, which can be used as emitter in organic light‐emitting diodes (OLEDs).^3^ The flexibility in fine‐tuning the optical and electrochemical properties of such compounds through manipulation of their chemical structure has been exploited for the preparation of tailored white‐light‐emitting devices.^4^

Apart from the 5‐membered rings, polycyclic phosphaphenalenes (**B**) as well as phospha‐fluoresceins, phospha‐rhodols and phospha‐rhodamines (**C**) with incorporated 6‐membered phosphorus heterocycles have recently been used for the development of highly fluorescent materials, also for applications in biological imaging.^5^, ^6^ In contrast, 6‐membered aromatic phosphinines have received little attention as building blocks for the construction of emissive π‐conjugated systems as it has been shown that 2,4,6‐triaryl‐λ^3^‐phosphinines are mostly non‐emissive at room temperature.^7^ In view of the strong relationship between a λ^3^−P=C and a C=C bond,^8^ it seems somewhat surprising that conjugated systems with P=C double bonds, such as phosphinines, are often non‐fluorescent, while most fluorescent molecules are based on conjugated C=C bonds. However, some notable exceptions show that not the P=C bond itself is responsible for the non‐emissive behavior.^9^, ^10^ Nevertheless, it is possible to restore the emission of phosphinine‐based π‐systems by introducing additional substituents at the P‐atom, or by coordination of the heterocycle to a transition metal center, respectively.^11^, ^12^ First quantitative photophysical measurements by one of us on λ^5^‐phosphinines, such as **D** and **E**, revealed a rather strong fluorescence emission at *λ*_max_=503 nm and a quantum yield of 20 % for **E**.^7^

More recently, 2,4,6‐triaryl‐ λ^5^‐phosphinines have been implemented in π‐conjugated, covalent phosphinine‐based frameworks.^13^ In 2018, Hayashi and co‐worker described the synthesis and optical properties of several λ^5^‐phosphinines of type **F** and fairly high quantum yields and tunable fluorophore properties were reported.^14^ These observations prompted us to envisage for the first time strategic structural variations on λ^5^‐phosphinines in order to fine‐tune their electronic properties and to elucidate structure–property relationships. Indeed, the classical synthetic route to λ^3^‐ and λ^5^‐phosphinines via pyrylium salts allows the introduction of different substituents in the 2,4,6‐positions of the phosphinine ring, while the substituents at the phosphorus atom can also be varied to a great extent by using amines or alcohols in combination with Hg(OAc)_2_ as an oxidation reagent. Here, we report on a detailed study on the chemistry of compounds of type **G**, including crystallographic characterizations, UV/Vis absorption, and fluorescence data, electrochemical behavior as well as theoretical calculations. Most importantly, we report also for the first time on the development of an OLED, based on a blue λ^5^‐phosphinine emitter of type **G**.

## Results and Discussion

**Figure 2:**
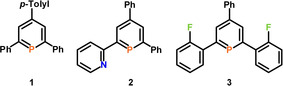
2,4,6‐Triaryl‐λ^3^‐phosphinines **1**–**3**.

The modular synthesis of 2,4,6‐triaryl‐λ^3^‐phosphinines allows the preparation of 2,6‐diphenyl‐4‐tolyl‐phosphinine **1**, pyridyl‐functionalized phosphinine **2** as well as ortho‐fluoro‐phenyl substituted phosphinine **3** (Figure 2). The pyridyl substituent was chosen, as the steric demand of a nitrogen lone pair is smaller than a CH group of a phenyl moiety, which permits a planar ground state of the conjugated ring system.^9^ In contrast, the increased steric bulk of the ortho‐fluoro‐phenyl group most likely destabilizes the planar structure. Consequently, the rotational barrier should increase considerably. λ^3^‐Phosphinines **1**–**3** were synthesized according to known literature procedures from the corresponding pyrylium‐salts and P(SiMe_3_)_3_.^7^, ^15^, ^16^

**Figure 3:**
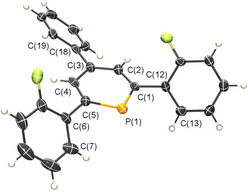
Molecular structure of **3** in the crystal. Displacement ellipsoids are shown at the 50 % probability level. Selected bond lengths (Å) and angles (°): P(1)−C(1): 1.756(6); P(1)−C(5): 1.753(6); C(1)−C(2): 1.386(7); C(2)−C(3): 1.375(8); C(3)−C(4): 1.422(7); C(4)−C(5): 1.401(7). C(5)‐P(1)‐C(1): 99.4(3); C(2)‐C(1)‐C(12)‐C(13): −134.0(5); C(4)‐C(5)‐C(6)‐C(7): 135.4(5); C(2)‐C(3)‐C(18)‐C(19): −142.6(5).

Crystals of 2,6‐di(2′‐fluorophenyl)‐4‐phenyl‐phosphinine, suitable for X‐ray diffraction, were obtained by slow crystallization from acetonitrile. The molecular structure of **3** in the crystal (Figure 3) shows the expected planar phosphorus heterocycle, while the three aryl groups are not in plane with the central hexagon. It should be noted that crystallographically characterized 2,4,6‐triaryl‐substituted λ^3^‐phosphinines are rare. This is in fact the first observation of the “statistical average” arrangement of the aryl groups attached to C(1) and C(5) for this class of compounds.^15^, ^17^ The rotational barrier of the ortho‐fluoro‐phenyl groups in **3** amounts to 4.9 kcal mol^−1^ (the rotational maximum corresponds to the planar structure, with the fluorine atom pointing toward the phosphorus atom at *ω*=0°, see Table S1). This value is higher than the typical 3 kcal mol^−1^ for 2,4,6‐triphenyl‐substituted phosphinines,^18^ which is apparently due to the presence of the sterically more demanding F‐substituent.

**Scheme 1:**
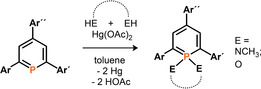
Synthesis of λ^5^‐phosphinines starting from λ^3^‐phosphinines.

The λ^3^‐phosphinines **1**–**3** were further converted quantitatively into a series of λ^5^‐phosphinines by reaction with Hg(OAc)_2_ as oxidation reagent in the presence of either 1,2‐ethanediol, catechol, or *N*,*N*'‐dimethyl‐ethylenediamine, according to a modified procedure described by Dimroth et al. (Scheme 1).^19^

**Figure 4:**
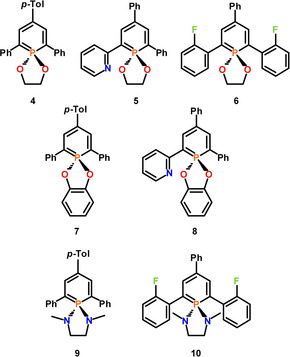
2,4,6‐Triaryl‐λ^5^‐phosphinines **4**–**10**.

After column chromatography, the λ^5^‐phosphinines **4**–**10** (Figure 4) were obtained as fairly air‐ and moisture‐stable orange and yellow solids in high isolated yields and were characterized by ^1^H‐, ^13^C‐ and ^31^P{^1^H} NMR spectroscopies. Compounds **4**–**10** show single resonances at around δ(ppm)=+60 in the ^31^P{^1^H} NMR spectrum. The shielding by approximately 130 ppm compared to the values of the corresponding λ^3^‐phosphinines is characteristic for λ^5^‐phosphinines.^19^

**Figure 5:**
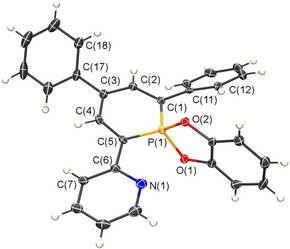
Molecular structure of **8** in the crystal. Displacement ellipsoids are shown at the 50 % probability level. Only one independent molecule is shown. Selected bond lengths (Å) and angles (°): P(1)−C(1): 1.714(3); P(1)−C(5): 1.715(3); C(1)−C(2): 1.387(4); C(2)−C(3):1.397(4); C(3)−C(4): 1.382(5); C(4)−C(5): 1.403(4); C(5)−C(6): 1.477(4); P(1)−O(1): 1.642(2); P(1)−O(2): 1.646(2); C(2)‐C(1)‐C(11)‐C(12): 138.3(3); C(4)‐C(5)‐C(6)‐C(7): 11.7(4); C(4)‐C(3)‐C(17)‐C(18): 140.3(3).

Crystals of **8** suitable for X‐ray diffraction were obtained by slow recrystallization from THF/Et_2_O. Compound **8** crystallizes with two independent molecules in the asymmetric unit and the molecular structure along with selected bond lengths and angles of one molecule is depicted in Figure 5. The crystallographic characterization of **8** shows the expected tetrahedral arrangement around the phosphorus atom, while the catechol unit is located perfectly perpendicular to the plane of the phosphorus heterocycle. Since the three aryl substituents in both the λ^5^‐phosphinine (**8**) and the λ^3^‐phosphinine (**3**) are attached at the 2‐,4‐ and 6‐position of the central core, a direct comparison of the crystallographic data is possible.

Upon oxidation of the P‐atom, the P(1)−C(1) and P(1)−C(5)‐distances become with 1.714(3) and 1.715(3) Å somewhat shorter than in a λ^3^‐phosphinine (see Figure 3), while the C−C distances are with 1.382–1.403 Å well equalized (benzene: 1.396 Å).^20^ These structural characteristics are in accordance with our earlier theoretical predictions for the parent λ^5^‐phosphinine. Its electronic structure can be explained by a specific ylidic cyclic delocalization in which the orbitals composed of the phosphorus atom and its two additional substituents are also involved (hyper‐conjugative effect).20a This non‐classical cyclic delocalization turns into a classical aromaticity when electronegative substituents (F, O, N) attached to the phosphorus atom, as shown by Schleyer and subsequently by Rzepa and co‐worker, who have attributed this observation to the dominance of negative hyperconjugation.^21^, ^22^ The modest aromaticity of the λ^5^‐phosphinines is also in agreement with our calculated NICS(1) values of −5 to −8 ppm for the investigated phosphinine rings (Table S2), which demonstrates that the aryl substituents have a minor effect on the electronic structure of the λ^5^‐phosphinines.

For compound **8**, we further found a C(2)‐C(1)‐C(11)‐C(12) torsion angle of 138.3(3)° for the phenyl group in 2‐position with the central heterocycle, while the pyridyl‐group is essentially coplanar with the phosphinine‐ring (C(7)‐C(6)‐C(5)‐C(4)=11.7(4)°). This is in accordance with our expectations for the steric demand of the nitrogen lone pair as discussed above.^9^

In contrast to 2,4,6‐triphenyl‐λ^3^‐phosphinine, for which a low barrier for the rotation of the phenyl groups was determined computationally (vide infra),^9^ we anticipated that the situation should be significantly different in 2,4,6‐triaryl‐λ^5^‐phosphinines. In these heterocycles, the two additional substituents at the phosphorus atom should have a substantial impact on the rotational barriers of the ortho‐aryl groups and their physical properties.

**Figure 6:**
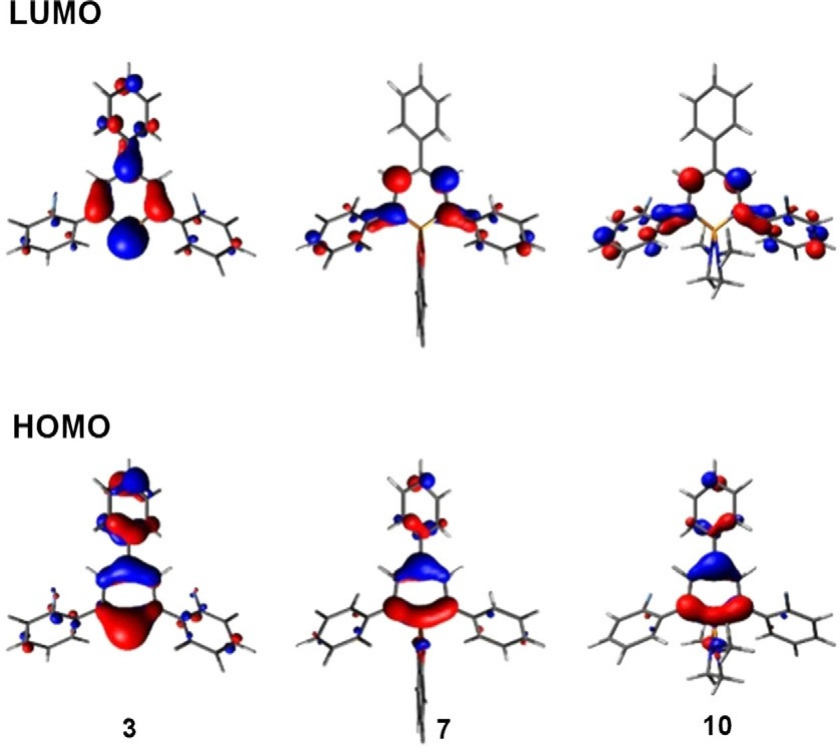
B3LYP/6‐31+G* HOMO and LUMO of **3**, **7** and **10**.

Consequently, to establish structure‐property relationships, the optical and electrochemical features of compounds **4**–**10** were examined. First, we investigated the redox properties of compounds **4**–**10** by means of cyclic voltammetry (CH_2_Cl_2_, 0.2 m, TBAPF_6_, *v*=200 mV s^−1^, see Table 2). All compounds show two oxidation waves, while no reduction processes were observed in the electrochemical window. The ionization energy of ylides in general,^23^ and particularly of λ^5^‐phosphinines,^24^ is low. This is in accordance with the partial negative charge at the carbon‐based C_5_‐fragment of the molecule (ylide‐character).20a, 24 The ylide‐character is a consequence of the electron distribution in the b_1_ type HOMO, which is influenced by the hyper‐conjugative interaction with the σ‐orbitals of the two additional phosphorus substituents, leading to its destabilization, as has been discussed before.20a Furthermore, the nature of the P‐substituents has a significant impact on the HOMO energy. An electronegative O‐substituent results in a less destabilized HOMO compared to an N‐substituent (Table 1). Thus, **9** and **10** (containing an *N*,*N*'‐dimethyl‐ethylenediamine moiety) have more destabilized HOMOs and show lower *E* ^ox1^ oxidation potentials than **4**–**8**, in which oxygen is attached to the phosphorus atom. Interestingly, compounds **7** and **8**, containing a catechol moiety at the phosphorus atom, are the most difficult to oxidize. Since the aryl rings in 2‐ and 6‐position of the P‐heterocycle do not contribute significantly to the HOMO (Figure 6 and Figure S23), the substitution of these rings has only a minor effect on the HOMO energies and the oxidation potentials. Only the aryl ring in 4‐position contributes weakly to the HOMO. The good correlation between the measured *E* ^ox1^ values and the HOMO energies is noteworthy.

**Table 1:** B3LYP/6‐31+G* HOMO energies [eV], first oxidation potentials (*E* ^ox1^ [V]) and decomposition temperatures (Td_5_ [°C]).

λ^5^	HOMO [eV]^[a]^	*E* ^ox1^ [V]^[b]^	Td_5_ [°C]^[d]^
**4**	−5.18	+0.79	264
**5**	−5.17	+0.83	271
**6**	−5.27	+0.93	249
**7**	−5.44	+1.09	240
**8**	−5.41	+1.04^[c]^	253
**9**	−4.83	+0.47	240
**10**	−4.91	+0.59	244

[a] All potentials were obtained during cyclic voltammetric investigations in 0.1 m Bu_4_NPF_6_ in CH_2_Cl_2_. Platinum electrode diameter 1 mm, sweep rate: 200 mV s−1. All reported potentials are referenced to the reversible formal potential of the decamethyl‐ferrocene/decamethylferrocenium couple. [b] Irreversible process. [c] Decomposition temperature at 5 % weight loss, measured by thermogravimetric analysis (TGA) under nitrogen.

**Figure 7:**
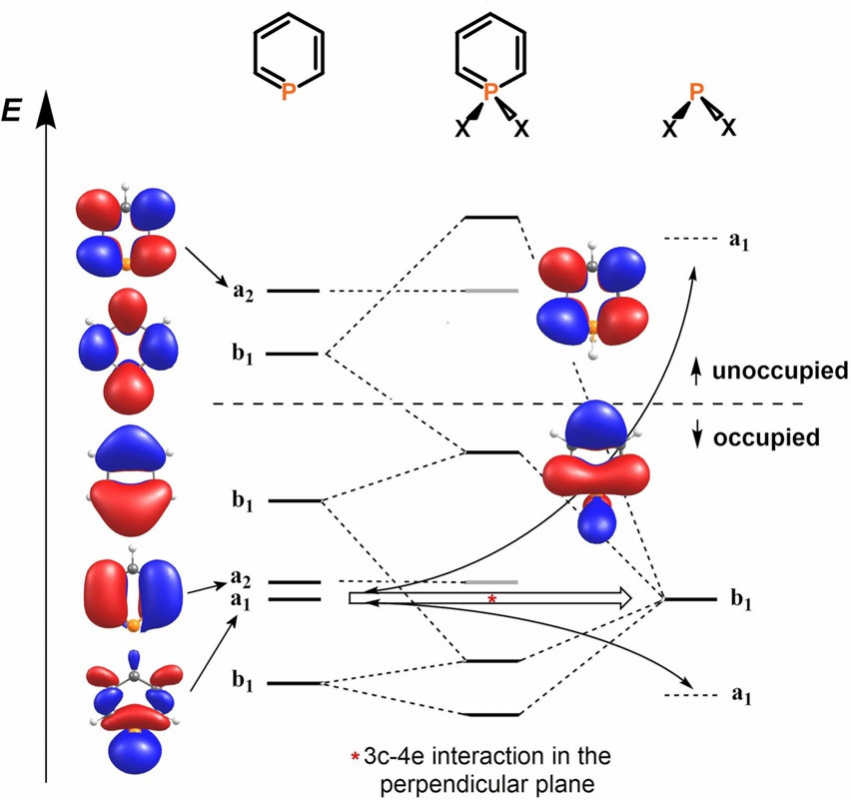
Frontier orbitals of a λ^3^‐ and a λ^5^‐phosphinine and formation of the 3c–4e bond.

The LUMO of a λ^5^‐phosphinine has a nodal plane through the heteroatom (a_2_ symmetry) and is similar to the LUMO+1 orbital of a λ^3^‐phosphinine (Figure 6 and 7), as we noted also before.20a This can be rationalized by the fact that the LUMO of a λ^3^‐phosphinine (b_1_ symmetry, likewise the HOMO) is destabilized by the hyperconjugative interactions with the σ‐orbitals at the P‐substituents being present in the λ^5^‐phosphinine. Consequently, the energy of the corresponding orbital in the λ^5^‐phosphinine is pushed above the orbital with a_2_ symmetry, which then becomes the LUMO, however, at significantly higher energies compared to the b_1_ type orbital of the λ^3^‐counterpart (Figure 7). All of this is in line with our observations, that no reduction wave was detected for λ^5^‐phosphinines **4**–**10**.

It is further noteworthy that the aryl substituents in 2‐ and 6‐positions of the heterocycle are involved in the LUMO to some extent. This is particularly clear from the bonding interactions between the connecting carbon atoms, even in those cases, where the substituent in 2‐ or 6‐position is rotated somewhat out of the plane of the heterocycle (Figure 6, LUMO of **7**, **10**).

**Figure 8:**
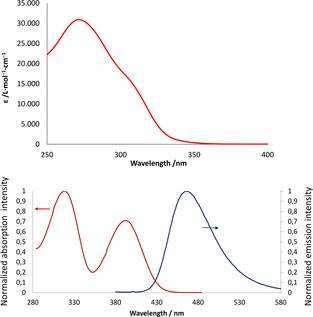
Absorption spectrum of compound **3** (top) and absorption and emission spectra of compound **10** recorded in CH_2_Cl_2_ (*c*=10^−5^ m) at room temperature.

Next, the optical properties (UV/Vis absorption and fluorescence) of compounds **4**–**10** were studied both in dichloromethane and in the solid‐state (Table 2). Compounds **4**–**10** show similar absorptions with two broad bands around *λ*=380 nm and *λ*=280 nm, respectively (Figure 8 and Figures S8–S22). The TD‐DFT calculated band maxima (vertical transition energies) are essentially HOMO–LUMO (see Figure S23) transitions and were obtained for the most stable rotamers. These values are systematically located by approximately 30 nm lower wavelengths than the experimentally observed ones (slightly different TD‐DFT calculated excitation energies for other rotamers are given in Tables S3a–S9a). The oscillator strength values are in most cases larger than 0.2 (see Table S3a–S9a) in accordance with the different (quasi)symmetry of the HOMO and LUMO. These spectra differ significantly from the spectrum recorded for λ^3^‐phosphinine **3**, which presents an intense band centered at *λ*=279 nm along with a red‐shifted shoulder tailing down to *λ*=345 nm. The lowest energy band for the λ^3^‐phosphinine was assigned to a HOMO→LUMO (π–π*) transition as was shown by our TD‐DFT calculations and corresponds to an intra‐phosphinine charge transfer with a strong contribution of the phosphorus atom in both orbitals. Since this transition is weakly allowed due to the small dipole moment change (note that in the parent λ^3^‐phosphinine both HOMO and LUMO have the same b_1_ symmetry), only a low fluorescence intensity is observed (quantum yields ≪1 %) as we noted already before.^7^

**Table 2:** Optical properties of λ^5^‐phosphinines **4**–**10**.

λ^5^	*λ*_max_ [nm]^[a]^	*ϵ* [mol^−1^⋅L cm^−1^]	*λ*_onset_ [nm]^[a]^	*λ*_em_ [nm]^[a]^	Φ_f_ [%]^[b]^	*λ*_em_ [nm]^[c]^	Φ_s_ [%]^[c]^	*λ*_calcd_ [nm]^[d]^
**4**	383	20 900	421	457	19	468	14	354
**5**	410	15 400	440	469	33	479	5	384
**6**	369	17 000	409	452	14	442	7	360
**7**	384	11 800	424	462	13	477	5	349
**8**	405	7700	435	465	27	489	3	378
**9**	411	8700	450	482	31	505	5	381
**10**	402	11 100	429	465	42	517	32	383

[a] Measured in CH_2_Cl_2_. [b] Fluorescence quantum yields determined using quinine sulfate as standard, ±15 %. [c] Measured in an integrated sphere. [d] TD‐DFT vertical absorption energy.

The λ^5^‐phosphinines **4**–**10** exhibit moderate to high fluorescence in solution (Table 2), which is in accordance with the rather large oscillator strength discussed above. Since the Stokes shift is different for each compound, the reorganization of the molecules between the ground state and the excited state should contribute to the different quantum efficiencies (vide infra).

The emission band maxima were also calculated for the most stable isomers by optimizing the excited state geometries by TD‐DFT (Table S3a–S9a). The calculated and the measured Stokes shifts are in reasonable agreement, while the largest deviation is seen in the case of compound **6**. The pyridyl‐functionalized systems **5** and **8**, in which the pyridyl‐substituent and the P‐heterocycle are coplanar both in the ground and excited states, show a small Stokes shift and, accordingly, a high quantum efficiency.

**Figure 9:**
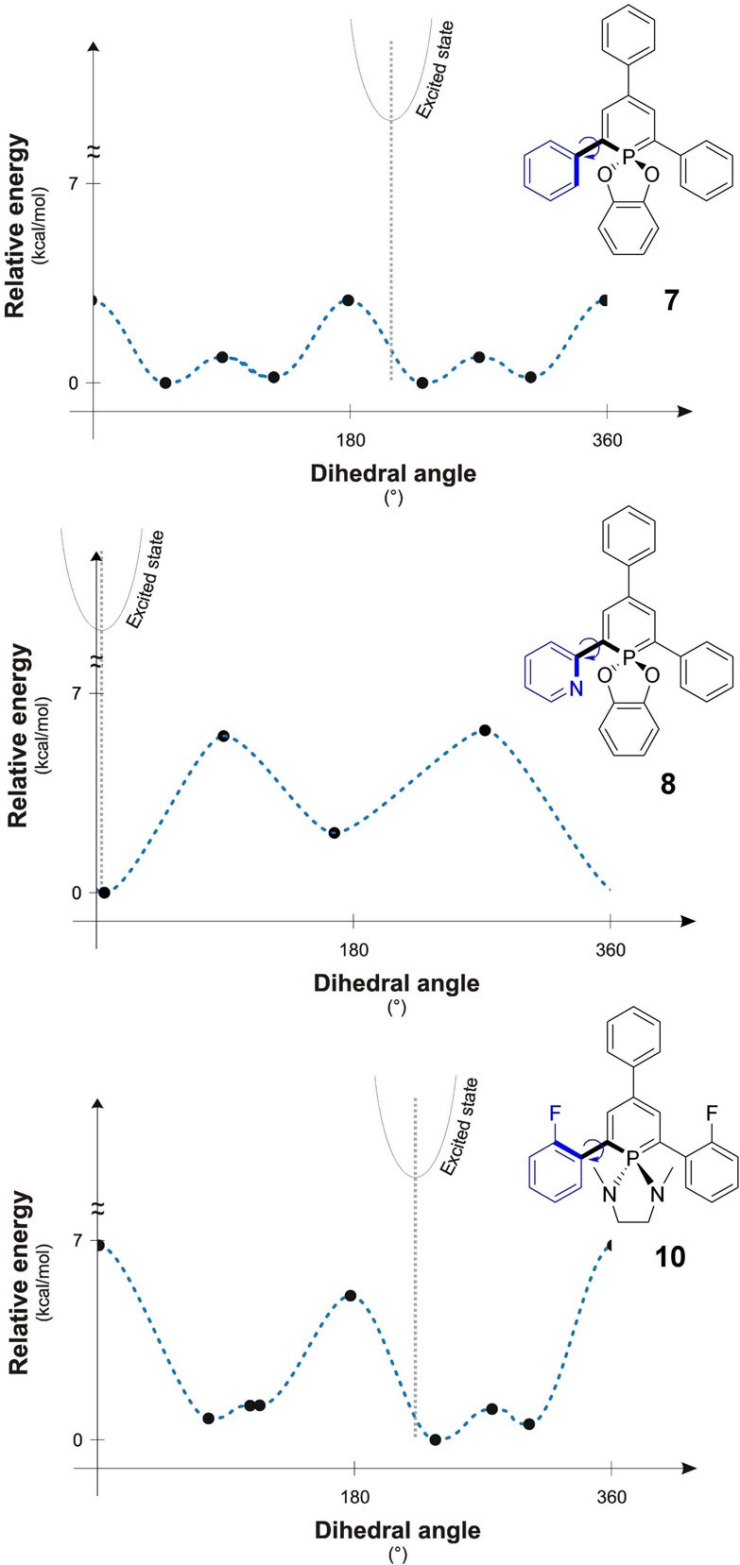
Rotational analysis for the groups at the 2‐position of **7**, **8** and **10**.

To further understand the photophysical properties of the different λ^5^‐phosphinines, we investigated the rotational barriers for all the connecting aryl groups, since this is the most likely pathway for a radiationless energy relaxation of the excited state. As a function of the angles ω, θ and φ, the highest rotational barriers for the λ^3^‐phosphinine **3** and the λ^5^‐phosphinines **4**–**10** are illustrated in Table 3 (for the more detailed rotational analysis see Tables S1b and S3b–S9b). The rotational barriers for the aryl group in 4‐position of the heterocycle (characterized by θ) in **4**–**10** are low and rather independent from the additional substituents at the phosphorus atom, including the case of λ^3^‐phosphinine **3**. As expected, the rotational barriers for the aryl groups in 2‐ and 6‐position (ω and φ) are higher for λ^5^‐phosphinines **6** and **10** than for the corresponding λ^3^‐phosphinine **3**. This hindrance of the rotation decreases the efficiency of the vibronic deactivation of the excited state and, consequently, contributes to the observed increase of the quantum yield. Also, compounds containing an α‐pyridyl group (**5** and **8**) have rather high rotational barriers. For the three representative examples **7**, **8** and **10** with rather different barriers, the full 360° relaxed rotation scan of one aryl group in 2‐position is depicted in Figure 9.

**Table 3:** Rotational barriers in kcal mol^−1^ for λ^3^‐phosphinine **3** and λ^5^‐phosphinines **4**–**10**.

	ω	θ	φ	
**3**	4.9^[a]^	3.1	4.9^[a]^	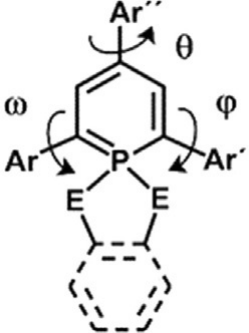
**4**	4.3^[a,b]^	2.6	4.3^[a,b]^	
**5**	6.6^[c]^	2.7	3.9^[b]^	
**6**	5.8^[b]^	2.8	5.8^[b]^	
**7**	2.9^[a,b]^	2.7	2.9^[a,b]^	
**8**	5.7^[c]^	2.8	3.2^[a,b]^	
**9**	3.4^[a,b]^	2.4	3.4^[a,b]^	
**10**	6.8^[a]^	2.5	6.8^[a]^	

[a] Rotational maximum at about *ω*=0°. [b] Rotational maximum at about *ω*=180°. [c] Rotation of the pyridyl group, rotational maximum at about *ω*=90°.

In case of **8**, the two rotational minima of the pyridine ring are located at the coplanar positions of the pyridyl and phosphinine rings due to the small steric need of the nitrogen lone pair.^9^ This allows for an efficient π‐conjugation. The rotational maxima are at the perpendicular positions of the substituent, where the overlap between the π‐systems is minimal. For **7** and **10**, the rotational potential energy surface is completely different. Repulsion of the α‐hydrogen, or the α‐fluoro atoms, with the substituents both at the 6‐membered ring and the P‐atom, hinders a coplanar arrangement. A planar form in case of **7** and **10** represents indeed a rotational maximum (with additional, but smaller rotational maxima at the perpendicular positions). For the 2‐fluorophenyl‐substituted phosphinine **10**, the rotational maxima at the coplanar positions are even higher than for **7**.

The TD‐DFT calculated excited states exhibit somewhat shortened C−C distances (by ca. 0.02–0.03 Å) between the phosphinine ring and the aromatic rings in 2‐ and 6‐position, in accordance with the π‐bonding nature of the LUMO between the two respective carbon atoms (Figures 6, 7 and Figure S23). As a consequence of the population of the LUMO in the excited state, the central P‐heterocycle and the aryl‐rings in 2‐ and 6‐position tend to reach a coplanar arrangement (see the insert showing the position of the excited state on the rotational scan in Figure 9).

The flattening is, however, less pronounced for the fluorophenyl‐substituted λ^5^‐phosphinines, which are more rigid due to the higher rotational barriers. In case of **8** (and **5**) having already a (nearly) coplanar arrangement of the pyridyl‐group and the core in the ground state (see above), the geometry change upon excitation is only minimal. Since this change is related to the Franck–Condon factor, which influences the transition probability, the fluorescent quantum yields are beneficially influenced by the small geometry change upon excitation, as it was also shown before.^8^, ^9^ As a consequence, the increased quantum yields for certain λ^5^‐phosphinines can be attributed to the high oscillator strength of the electronic transition, and also to the increase of the rotational barrier of the α‐substituent. No increase of the quantum yields is observed in the solid‐state indicating the presence of aggregate quenched emissions in this series of compounds (Table 2).

**Figure 10:**
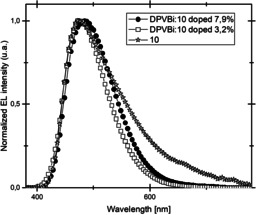
Normalized EL spectrum of doped and non‐doped OLEDs devices.

Taking into account the thermal stabilities and the optical and redox properties of compounds **4**–**10**, only compound **10** was used as emitting material (EM), either pure or doped in a DPVBi (4,4′‐bis(2,2′‐diphenylvinyl)‐1,1′‐biphenyl) matrix. In the first attempt, compound **10** was used as pure emitter in an organic light‐emitting diode (OLED) with the following configuration: Glass/ ITO/ CuPc (10 nm)/ α‐NPB (50 nm)/ **10** (40 nm)/ BCP (10 nm)/ Alq_3_ (10 nm)/ LiF (1.2 nm)/ Al (100 nm) (ITO=indium tin oxide; CuPC=Cu^II^phtalocyanine; α‐NPB=*N*,*N*′‐Bis‐(1‐naphthalenyl)‐*N*,*N*′‐bis‐phenyl‐(1,1′‐biphenyl)‐4,4′‐diamine; BCP=bathocuproine; Alq_3_=tris‐(8‐hydroxyquinolinato)aluminum). The electroluminescence (EL) performance of the resulting devices are reported in Table 4 and the EL spectra are shown in Figure 10.

**Table 4:** Electroluminescent performance of devices **A**–**C**.

Device	Emitter	Doping rate [%]	V_on_ [V]^[a]^	EQE [%]^[b]^	CE [cd A^−1^]^[b]^	PE [lm W^−1^]^[b]^
A	**10**	pure	5.3	0.04	0.09	0.04
B	**10**	3.2	5.0	0.96	1.87	0.58
C	**10**	7.9	4.9	0.67	1.40	0.46

[a] Threshold voltage recorded at luminance of 1 cd m^−2^. [b] EQE (external quantum efficiency), CE (current efficiency), and PE (power efficiency) recorded at 10 mA cm^−2^.

For the pure emitter, the EL emission peaked at *λ*=485 nm with a red‐shifted shoulder in the range of *λ*=550–600 nm. This indicates that compound **10** may form aggregates after vacuum evaporation. The EL performance is moderate (Table 4) since the charge transport in the emitting layer, containing compound **10**, maybe low and the emission is quenched by the formation of the proposed aggregates.

Nevertheless, an increase of the performance could be observed when compound **10** is used as dopant (3.2–7.9 %wt, Table 4) in a DPVBi matrix. As a result of diluting compound **10** in the DPVBi matrix, efficient charge transport properties are generated. Moreover, doping the blue matrix with 3.2 % of compound **10** leads to an OLED, which exhibits a turn‐on voltage of 5.0 V with current and power efficiencies of 1.87 cd A^−1^ and 0.58 lm W^−1^, respectively. Interestingly, the external quantum efficiency (EQE) is dramatically improved compared to the pure emitter (0.9 % for device B and 0.04 % for device A). However, an increase of the doping ratio of up to 7.9 %wt led to a decrease of the performance (device C) and a shift of the EL peak from *λ*=474 nm for device B to *λ*=485 nm for device C (Figure 10). It is noteworthy for the higher doping rate, that the EL peak appears at the same value as for the pure emitter (device A).

## Conclusion

We synthesized a series of 2,4,6‐triaryl‐λ^5^‐phosphinines by combining the highly modular pyrylium salt route for the preparation of λ^3^‐phosphinines with an efficient oxidation process to introduce additionally different R_2_N‐ or RO‐substituents at the phosphorus atom. We found that the optical and redox properties of these compounds can be varied to some extend by the nature of the substituents. Theoretical calculations helped to rationalize our observations and we could clearly demonstrate that λ^5^‐phosphinines can be efficient emitters in contrast to their λ^3^‐counterparts. In addition, the thermal stability of **10** prompted us to use this compound as a blue fluorescent emitting material for the construction of an OLED with current and power efficiencies of 1.87 cd A^−1^ and 0.58 lm W^−1^. These preliminary results demonstrate that λ^5^‐phosphinine‐based emitters can indeed be used to fabricate optoelectronic devices. Further structural variations in λ^5^‐phosphinines, supported by DFT calculations and improvements of their performance in OLED devices are currently performed in our laboratories.

## Experimental Section

**General**: Unless otherwise stated, all syntheses were performed under an inert argon atmosphere using modified Schlenk techniques or in a MBraun glovebox. All common chemicals were commercially available and were used as received. Dry or deoxygenated solvents were prepared using standard techniques or used from a MBraun solvent purification system. The NMR spectra were recorded on a JEOL ECX400 (400 MHz) spectrometer and chemical shifts are reported relative to the residual resonance in the deuterated solvents. Phosphinines **1**,^15^ **2** 7 and **3** 16 were prepared according to the literature.

UV/Vis spectra were recorded at RT with a VARIAN Cary 5000 spectrophotometer. UV/Vis/NIR emission and excitation spectra measurements were recorded with an FL 920 Edinburgh Instrument equipped with a Hamamatsu R5509‐73 photomultiplier for the NIR domain (300–1700 nm) and corrected for the response of the photomultiplier. Quantum yields were calculated relative to quinine sulfate (H_2_SO_4_, 0.1 m, φ_ref_=0.55). The electrochemical studies were carried out under argon with an Eco Chemie Autolab PGSTAT 30 potentiostat for cyclic voltammetry with the three‐electrode configuration: the working electrode was a platinum disk, the reference electrode was a saturated calomel electrode, and the counter‐electrode was a platinum wire. All potentials were internally referenced to the ferrocene/ferrocenium couple. For the measurements, concentrations of 10^−3^ m of the electroactive species were used in freshly distilled and degassed dichloromethane and 0.2 m tetrabutylammonium hexafluorophosphate. Thermogravimetric analyses were performed with a Mettler‐Toledo TGA‐DSC‐1 apparatus under dry nitrogen flow at a heating rate of 10 °C min^−1^. All measurements were performed with quartz cuvettes with a path length of 1.0 cm.

For NMR, absorption, emission and excitation spectra see Supporting Information.

**Synthesis of 1,1‐ethyleneglycolyl‐λ^5^‐2,6‐diphenyl‐4‐(*p*‐tolyl)phosphinine (4)**: Phosphinine **1** (200 mg, 0.59 mmol) and mercury acetate (207 mg, 0.65 mmol) were dissolved in 5 mL toluene in an argon atmosphere and then mixed with ethylene glycol (0.04 mL, 0.60 mmol) at room temperature. After stirring for overnight, the solution was filtered over silica (3 cm) to remove the mercury residues. The solvent of the neon yellow filtrate was then removed under vacuum and the residue was washed with pentane. After drying under high vacuum, the product is obtained as a neon yellow solid (157 mg, 0.39 mmol, 66 %). ^1^H NMR (400 MHz, CDCl_3_): *δ*=7.69 (d, ^3^ *J*_H,P_=39.5 Hz, 2 H, C_5_ *H*_2_P), 7.63–7.54 (m, 4 H, H_ar_), 7.46–7.36 (m, 4 H, H_ar_), 7.36 (d, *J=*8.1 Hz, 2 H, H_ar_), 7.31 (t, *J=*7.2 Hz, 2 H, H_ar_), 7.15 (d, *J=*7.8 Hz, 2 H), 4.19 (d, ^3^ *J*_H,P_=10.3 Hz, 4 H, CH_2_‐CH_2_), 2.35 (s, 3 H, CH_3_) ppm; ^13^C{^1^H} NMR (101 MHz, CDCl_3_): *δ*=140.3 (d, *J=*2.6 Hz), 139.5 (d, *J=*4.1 Hz), 138.9 (d, *J=*10.7 Hz), 134.5, 129.4, 129.3 (d, *J=*1.7 Hz), 128.8 (d, *J=*1.1 Hz), 126.6 (d, *J=*1.5 Hz), 126.1 (d, *J=*0.7 Hz), 116.4 (d, *J=*19.5 Hz), 97.2 (d, ^1^ *J*_P,C_=145.4 Hz, C^1, 5^(C_5_H_2_P)), 66.6 (d, ^2^ *J*_P,C_=1.8 Hz, CH_2_‐CH_2_), 21.1 (CH_3_) ppm; ^31^P{^1^H} NMR (162 MHz, CDCl_3_): *δ*=69.2 ppm. EI (*m*/*z*): 398.1580 g mol^−1^ (calculated: 398.1435 g mol^−1^) [*M*]^+^.

**Synthesis of 1,1‐ethyleneglycolyl‐λ^5^‐2‐(2‘‐pyridyl)‐4,6‐diphenylphosphinine (5)**: Phosphinine **2** (100 mg, 0.31 mmol) and mercury acetate (103 mg, 0.33 mmol) were dissolved in 5 mL toluene in an argon atmosphere and then mixed with ethylene glycol (0.02 mL, 0.33 mmol) at room temperature. After stirring for overnight, the solution was filtered over silica (3 cm) to remove the mercury residues. The solvent of the fluorescent yellow‐green filtrate was then removed under vacuum and the residue was washed with pentane. After drying in high vacuum, the product was obtained as a neon yellow solid (70.4 mg, 0.18 mmol, 59 %). ^1^H NMR (400 MHz, CDCl_3_): *δ*=8.46 (d, *J=*5.8 Hz, 1 H, H_ar_), 8.01 (dd, ^3^ *J*_H,P_=40.4 Hz, ^4^ *J*_H,H_=2.7 Hz, 1 H, H_ar_), 7.76 (dd, *J=*38.2, 2.7 Hz, 1 H, H_ar_), 7.69–7.59 (m, 2 H, H_ar_), 7.64–7.44 (m, 4 H, H_ar_), 7.47–7.26 (m, 5 H, H_ar_), 7.26–7.15 (m, 1 H, H_ar_), 7.05–6.96 (m, 1 H, H_ar_), 4.76–4.66 (m, 2 H, CH_2_‐CH_2_), 4.36–4.25 (m, 2 H, CH_2_‐CH_2_) ppm; ^13^C{^1^H} NMR (101 MHz, CDCl_3_): *δ*=148.4, 143.2, 141.6 (d, *J=*10.2 Hz), 136.9, 133.6 (d, *J=*9.2 Hz), 129.2 (d, *J=*5.6 Hz), 128.7 (d, *J=*9.6 Hz), 126.7, 126.3, 125.1, 119.4, 117.6 (d, *J=*9.2 Hz), 116.6 (d, *J=*18.9 Hz), 67.4 (d, *J=*1.1 Hz) ppm; ^31^P{^1^H} NMR (162 MHz, CDCl_3_): *δ*=72.9 ppm. EI (*m*/*z*): 385.1201 g mol^−1^ (calculated: 385.1232 g mol^−1^) [*M*]^+^.

**Synthesis of 1,1‐ethyleneglycolyl‐λ^5^‐2,6‐bis(2‐fluorophenyl)‐4‐phenyl‐phosphinine (6)**: Phosphinine **3** (100 mg, 0.28 mmol) and mercury acetate (95.0 mg, 0.30 mmol) were dissolved in 5 mL toluene in an argon atmosphere and subsequently mixed with ethylene glycol (0.02 mL, 0.30 mmol) at room temperature. After stirring for overnight, the solution was filtered over silica (3 cm) to remove the mercury residues. The solvent of the neon yellow filtrate was then removed under vacuum and the residue was washed with pentane. After drying under high vacuum, the product was obtained as a neon yellow solid (75.6 mg, 0.21 mmol, 74 %). ^1^H NMR (400 MHz, CDCl_3_): *δ*=7.68 (d, ^3^ *J*_H,P_=39.6 Hz, 2 H), 7.57–7.52 (m, 2 H, H_ar_), 7.45–7.41 (m, 2 H, H_ar_), 7.37–7.25 (m, 4 H, H_ar_), 7.23–7.11 (m, 5 H, H_ar_), 4.04 (d, ^3^ *J*_H,P_=10.4 Hz, 4 H, CH_2_‐CH_2_) ppm. ^19^F NMR (376 MHz, CDCl_3_): *δ*=−116.9 (m) ppm; ^31^P{^1^H} NMR (162 MHz, CDCl_3_): *δ*=67.6 ppm. EI (*m*/*z*): 420.1138 g mol^−1^ (calculated: 420.1091 g mol^−1^) [*M*]^+^.

**Synthesis of 1,1‐catecholyl‐λ^5^‐2,6‐diphenyl‐4‐(*p*‐tolyl)phosphinine (7)**: Phosphinine **1** (200 mg, 0.59 mmol) and mercury acetate (207 mg, 0.65 mmol) were dissolved in 5 mL toluene in an argon atmosphere and then catechol (71.0 mg, 0.65 mmol) was added at room temperature. After stirring for overnight, the solution was filtered over silica (3 cm) to remove the mercury residues. The solvent of the neon yellow filtrate was then removed under vacuum and the residue was washed with pentane. After drying under high vacuum, the product was obtained as a neon yellow solid (230 mg, 0.52 mmol, 87 %). ^1^H NMR (400 MHz, CD_2_Cl_2_): *δ*=7.82 (d, ^3^ *J*_P,H_=42.5 Hz, 2 H, C_5_ *H*_2_P), 7.51–7.43 (m, 4 H, H_ar_), 7.46–7.36 (m, 2 H, H_ar_), 7.32–7.22 (m, 4 H, H_ar_), 7.24–7.16 (m, 4 H, H_ar_), 7.01–6.93 (m, 4 H, H_ar_), 2.36 (s, 3 H, CH_3_) ppm; ^13^C{^1^H} NMR (101 MHz, CD_2_Cl_2_): *δ*=145.5 (d, *J=*1.0 Hz), 140.0 (d, *J=*3.1 Hz), 138.6 (d, *J=*10.5 Hz), 138.2 (d, *J=*4.0 Hz), 135.8, 129.9, 129.4 (d, *J=*0.9 Hz), 128.9 (d, *J=*7.1 Hz), 127.3 (d, *J=*1.5 Hz), 126.7 (d, *J=*0.9 Hz), 124.2, 119.4 (d, *J=*21.3 Hz), 111.8 (d, *J=*11.0 Hz), 98.2 (d, *J=*144.5 Hz, C_5_H_2_P), 21.2 (d, *J=*1.5 Hz, *C*H_3_) ppm; ^31^P{^1^H} NMR (162 MHz, CD_2_Cl_2_): *δ*=70.6 ppm. ^31^P NMR (162 MHz, CD_2_Cl_2_): *δ*=70.6 (t, ^3^ *J*_P,H_=42.5 Hz) ppm. EI (*m*/*z*): 446.1528 g mol^−1^ (calculated: 446.1436 g mol^−1^) [*M*]^+^.

**Synthesis of 1,1‐catecholyl‐λ^5^‐2‐(2‘‐pyridyl)‐4,6‐diphenyl‐phosphinine (8)**: Phosphinine **2** (100 mg, 0.31 mmol) and mercury acetate (103 mg, 0.33 mmol) were dissolved in 5 mL toluene in an argon atmosphere and then catechol (35.0 mg, 0.33 mmol) was added at room temperature. After stirring for overnight, the solution was filtered over silica (3 cm) to remove the mercury residues. The solvent of the fluorescent yellow–green filtrate was then removed under vacuum and the residue was washed with pentane. After drying under high vacuum, the product was obtained as a neon yellow solid (115 mg, 0.27 mmol, 86 %). ^1^H NMR (400 MHz, CDCl_3_): *δ*=8.08 (dd, ^3^ *J*_H,P_=43.9 Hz, ^4^ *J*_H,H_=2.7 Hz, 1 H, C_5_ *H*_2_P), 7.89 (dd, ^3^ *J*_H,P_=41.2 Hz, ^4^ *J*_H,H_=2.7 Hz, 1 H, C_5_ *H*_2_P), 7.76 (d, *J=*4.8 Hz, 1 H), 7.63–7.49 (m, 6 H, H_ar_), 7.41 (t, *J=*7.6 Hz, 2 H, H_ar_), 7.32–7.18 (m, 4 H, H_ar_), 7.04–6.92 (m, 4 H, H_ar_), 6.88–6.83 (m, 1 H, H_ar_) ppm; ^13^C{^1^H} NMR (101 MHz, CDCl_3_): *δ* ppm^−1^=157.6 (d, *J=*1.9 Hz), 148.5, 146.2, 142.7 (d, *J=*2.7 Hz), 141.1 (d, *J=*10.5 Hz), 138.1 (d, *J=*3.8 Hz), 136.8, 132.7 (d, *J=*8.6 Hz), 128.9 (d, *J=*0.9 Hz), 128.8, 128.7, 128.6, 126.9 (d, *J=*1.4 Hz), 126.5 (d, *J=*1.1 Hz), 125.7, 123.2, 119.9 (d, *J=*0.9 Hz), 118.7 (d, *J=*21.1 Hz), 116.8 (d, *J=*10.1 Hz), 110.9 (d, *J=*11.3 Hz), 101.6 (d, ^1^ *J*_P,C_=144.9 Hz, C^1, 5^(*C*_5_H_2_P)) ppm; ^31^P{^1^H} NMR (162 MHz, CDCl_3_): *δ*=75.2 ppm. EI (*m*/*z*): 433.1298 g mol^−1^ (calculated: 433.1232 g mol^−1^) [*M*]^+^.

**Synthesis of 1,1‐*N***,***N*** **’‐dimethylethylenediaminyl‐λ^5^‐2,6‐diphenyl‐4‐(*p*‐tolyl)‐phosphinine (9)**: Phosphinine **1** (200 mg, 0.59 mmol) and mercury acetate (207 mg, 0.65 mmol) were dissolved in 5 mL toluene in an argon atmosphere and subsequently mixed with *N*,*N*′‐dimethylethylenediamine (0.07 mL, 0.65 mmol) at room temperature. After stirring for overnight, the solution was filtered over silica (3 cm) to remove the mercury residues. The solvent of the neon yellow filtrate was then removed in a vacuum and the residue washed with pentane. After drying under high vacuum, the product was obtained as a neon yellow solid (117 mg, 0.28 mmol, 47 %). ^1^H NMR (400 MHz, CDCl_3_): *δ*=7.74 (d, ^3^ *J*_H,P_=33.5 Hz, 2 H, C_5_ *H*_2_P), 7.50–7.41 (m, 4 H, H_ar_), 7.38 (d, *J=*8.1 Hz, 2 H, H_ar_), 7.33 (t, *J=*7.6 Hz, 4 H, H_ar_), 7.23–7.17 (m, 2 H, H_ar_), 7.13 (d, *J=*7.9 Hz, 2 H, H_ar_), 3.11 (d, ^3^ *J*_H,P_=7.5 Hz, 4 H, CH_2_‐CH_2_), 2.36 (d, ^3^ *J*_H,P_=10.4 Hz, 6 H, N‐CH_3_), 2.34 (s, 3 H, Ph‐CH_3_) ppm; ^13^C{^1^H} NMR (101 MHz, CDCl_3_): *δ*=142.2 (d, *J=*6.0 Hz), 140.8, 138.1 (d, *J=*9.6 Hz), 133.4, 129.3, 128.4, 128.3 (d, *J=*5.7 Hz), 125.4–125.3 (d, *J=*1.2 Hz), 125.2, 113.5 (d, *J=*15.6 Hz), 95.8 (d, ^1^ *J*_P,C_=126.2 Hz, C^1, 5^(C_5_H_2_P)), 48.3 (d, ^2^J_P,C_=8.7 Hz, CH_2_‐CH_2_), 31.3 (d, ^2^J_P,C_=8.2 Hz, N‐CH_3_), 21.0 (Ph‐CH_3_) ppm; ^31^P{^1^H} NMR (162 MHz, CDCl_3_): *δ*=36.2 ppm. EI (*m*/*z*): 424.2075 g mol^−1^ (calculated: 424.2063 g mol^−1^) [*M*]^+^.

**Synthesis of 1,1‐*N***,***N*** **’‐dimethylethylenediaminyl‐λ^5^‐2,6‐bis(2‐fluorophenyl)‐4‐phenylphosphinine (10)**: Phosphinine **3** (500 mg, 1.39 mmol) and mercury acetate (486 mg, 1.53 mmol) are dissolved in 15 mL toluene in an argon atmosphere and subsequently mixed with *N*,*N*′‐dimethylethylenediamine (0.16 mL, 1.53 mmol) at room temperature. After stirring for overnight, the solution was filtered over silica (3 cm) to remove the mercury residues. The solvent of the neon yellow filtrate was then removed under vacuum and the residue was washed with pentane. After drying under high vacuum, the product was obtained as a neon yellow solid (141 mg, 0.32 mmol, 23 %). ^1^H NMR (400 MHz, CDCl_3_): *δ*=7.68 (d, ^3^ *J*_H,P_=33.8 Hz, 2 H, C_5_ *H*_2_P), 7.45 (d, *J=*8.0 Hz, 2 H, H_ar_), 7.35–7.18 (m, 6 H, H_ar_), 7.16–7.02 (m, 5 H, H_ar_), 2.89 (d, ^3^ *J*_H,P_=8.0 Hz, 4 H, CH_2_‐CH_2_), 2.48 (d, ^3^J_H,P_=10.4 Hz, 6 H, N‐CH_3_) ppm; ^13^C{^1^H} NMR (101 MHz, CDCl_3_): *δ*=140.4, 132.4, 128.5, 128.2, 127.7 (d, *J=*8.0 Hz), 125.1, 123.8, 123.6 (d, *J=*3.7 Hz), 123.4, 121.8, 121.5, 115.8 (d, *J=*23.5 Hz), 98.2 (d, ^1^ *J*_P,C_=131.4 Hz, C^1, 5^(C_5_H_2_P)), 47.4 (d, ^2^ *J*_P,C_=8.8 Hz, *C*H_2_‐*C*H_2_), 31.3 (d, ^2^ *J*_P,C_=8.4 Hz, *N*‐*C*H_3_) ppm; ^19^F NMR (376 MHz, CDCl_3_): *δ*=−114.7 ppm; ^31^P{^1^H} NMR (162 MHz, CDCl_3_): *δ*=34.6 ppm. EI (*m*/*z*): 446.1903 g mol^−1^ (calculated: 446.1718 g mol^−1^) [*M*]^+^.

**X‐ray crystal structure determination of 3**: C_23_H_15_F_2_P, *F*w=360.32, colorless stick, 0.01×0.03×0.17 mm^3^, orthorhombic, *Pna*2_1_, *a=*7.7176(3), *b=*19.6751(7), *c=*11.5400(5) Å, *V=*1752.29(12) Å^3^, *Z=*4, *D*_x_=1.366 g cm^−3^, *μ*=1.587 mm^−1^. 11690 reflections were measured by a Bruker D8‐Venture diffractometer with a Photon area detector (CuKα radiation; *λ*=1.54178 Å) at a temperature of *T=*100(2) K up to a resolution of *θ*_max_=79.29. The reflections were corrected for absorption and scaled on the basis of multiple measured reflections by using the SADABS program (0.77–0.98 correction range).^25^ 2572 reflections were unique (*R*_int_ *=*0.045). The structures were solved with SHELXS‐1997 by using direct methods and refined with SHELXL‐2017 on *F* ^2^ for all reflections.^26^ Non‐hydrogen atoms were refined with anisotropic displacement parameters. 235 parameters were refined without restraints. *R1=*0.057 for 2572 reflections with *I>*2s(*I*) eÅ^3^, and *wR2=*0.154 for 2878 reflections, *S=*1.081. Geometry calculations and checks for higher symmetry were performed with the PLATON program.^27^ CCDC 1968707 contains the supplementary crystallographic data for this compound. These data are provided free of charge by The Cambridge Crystallographic Data Centre.

**X‐ray crystal structure determination of 8**: C_28_H_20_NO_2_P, *F*w=433.42, orange block, 0.16×0.31×0.32 mm^3^, monoclinic, *P2_1_*, *a=*10.6265(2), *b=*7.5595(4), *c=*12.1521(3) Å, *V=*2137.37(8) Å^3^, *Z=*4, *D*_x_=1.347 g cm^−3^, *μ*=0.155 mm^−1^. 78 641 reflections were measured by a Bruker D8‐Venture diffractometer with a Photon area detector (Mo_Kα_ radiation; *λ*=0.71073 Å) at a temperature of *T=*100(2) K up to a resolution of *θ*_max_=27.16. The reflections were corrected for absorption and scaled on the basis of multiple measured reflections by using the SADABS program (0.92–1.00 correction range).^25^ 28 752 reflections were unique (*R_int_=*0.056). The structures were solved with SHELXS‐2013 by using direct methods and refined with SHELXL‐2013 on *F* ^2^ for all reflections.^26^ Non‐hydrogen atoms were refined with anisotropic displacement parameters. 578 parameters were refined without restraints. *R1=*0.035 for 8752 reflections with *I>*2s(*I*) eÅ^3^, and *wR2=*0.096 for 9479 reflections, *S=*1.119. Geometry calculations and checks for higher symmetry were performed with the PLATON program.^27^ CCDC 1968706 contains the supplementary crystallographic data for this compound. These data are provided free of charge by The Cambridge Crystallographic Data Centre.

**Computational details**: Density functional calculations were carried out with the Gaussian 09 program package.^28^ All structures were optimized using the B3LYP functional,^29^ combined with the 6‐31+G* basis set, and these results were discussed throughout. For the conformational search of **3** further calculations were carried out using the M06‐2X and the ωB97XD functionals and the cc‐pVTZ basis, which gave similar results to B3LYP/6‐31+G* (See Table S1a in the Supporting Information). At each of the optimized structures vibrational analysis was carried out to check that the stationary point located is a minimum of the potential energy hypersurface (no imaginary frequencies were obtained). Relaxed scans were calculated to describe the rotational behavior of the α‐aryl groups. To obtain vertical excitation energies and optimized excited state structures TD DFT B3LYP/6‐31+G* calculations were carried out. The optimized excited state geometries were used for the calculation of the position of the emission spectral maxima. For the visualization of the molecular orbitals the VMD program^30^ was used.

**OLED device fabrication**: The OLED devices were fabricated onto indium tin oxide (ITO) glass substrates purchased from Xin Yang Technology (90 nm thick, sheet resistance of 15 Ω m^−1^). Prior to organic layer deposition, the ITO substrates were cleaned by sonication in a detergent solution, rinsed twice in de‐ionized water and then in isopropanol solution and finally treated with UV‐ozone during 15 minutes. The OLEDs stack is the following: Glass/ ITO/ CuPc (10 nm)/ α‐NPB (40 nm)/ EML 40 nm/ BCP (10 nm)/ Alq_3_ (40 nm)/ LiF (1.2 nm)/ Al (100 nm). Cu^II^ phthalocyanine (CuPc) is used as hole injection layer (HIL), *N*,*N*′‐bis‐(1‐naphthalenyl)‐*N*,*N*′‐bis‐phenyl‐(1,1′‐biphenyl)‐4,4′‐diamine (α‐NPB) as hole transport layer (HTL), bathocuproine (BCP) as hole blocking layer (HBL), tris‐(8‐hydroxyquinoline)aluminum (Alq_3_) as electron transport layer (ETL), lithium fluoride as electron injection layer (EIL) and 100 nm of aluminum as the cathode, respectively. The emitting layer (EML) is compound **10** either as a neat film (device A) or a host‐guest system (devices B and C). The host material is 4,4′‐bis(2,2‐diphenylvinyl)‐1,1′‐biphenyl, (DPVBi). The doping ratio were 3.2 and 7.9 %wt, respectively. All the organic materials were purchased from commercial companies except molecule **10**. Organic layers were sequentially deposited onto the ITO substrate at a rate of 0.2 nm s^−1^ under high vacuum (10^−7^ mbar). The doping rate was controlled by simultaneous co‐evaporation of the host and the dopant. An in situ quartz crystal was used to monitor the thickness of the layer depositions with an accuracy of 5 %. The active area of the devices defined by the Al cathode was 0.3 cm^2^. The organic layers and the LiF/Al cathode were deposited in a one‐step process without breaking the vacuum.

**Device characterization**: After deposition, all the measurements were performed at room temperature and under ambient atmosphere with no further encapsulation of devices. The current–voltage‐luminance (I‐V‐L) characteristics of the devices were measured with a regulated power supply (ACT100 Fontaine) combined with a multimeter (Keithley) and a 1 cm^2^ area silicon calibrated photodiode (Hamamatsu). Electroluminescence (EL) spectra and chromaticity coordinates of the devices were recorded with a PR650 SpectraScan spectrophotometer, with a spectral resolution of 4 nm.

## Conflict of interest

The authors declare no conflict of interest.

## Supporting information

As a service to our authors and readers, this journal provides supporting information supplied by the authors. Such materials are peer reviewed and may be re‐organized for online delivery, but are not copy‐edited or typeset. Technical support issues arising from supporting information (other than missing files) should be addressed to the authors.

Supplementary
