# Supplementary material for: Synthesis, Electronic Properties and OLED Devices of Chromophores Based on λ5‐Phosphinines
Source: Chemistry. 2020 Aug 6;26(46):10534–43. doi: 10.1002/chem.202000932 (PMC7496645; doi:10.1002/chem.202000932)
Supplement: Supplementary file 1 — Supplementary [file CHEM-26-10534-s001.pdf]

Chemistry–A European Journal

Supporting Information

Synthesis, Electronic Properties and OLED Devices of Chromophores Based on λ^5-Phosphinines

Gregor Pfeifer,[a] Faouzi Chahdoura,[b] Martin Papke,[a] Manuela Weber,[a] Rózsa Szűcs,[c] Bernard Geffroy,[d] Denis Tondelier,[d] László Nyulászi,*[c] Muriel Hissler,*[b] and Christian Müller*[a]

Table of Content

NMR Spectra **page S2**

Photophysical properties **page S11**

Computational details **page S17**

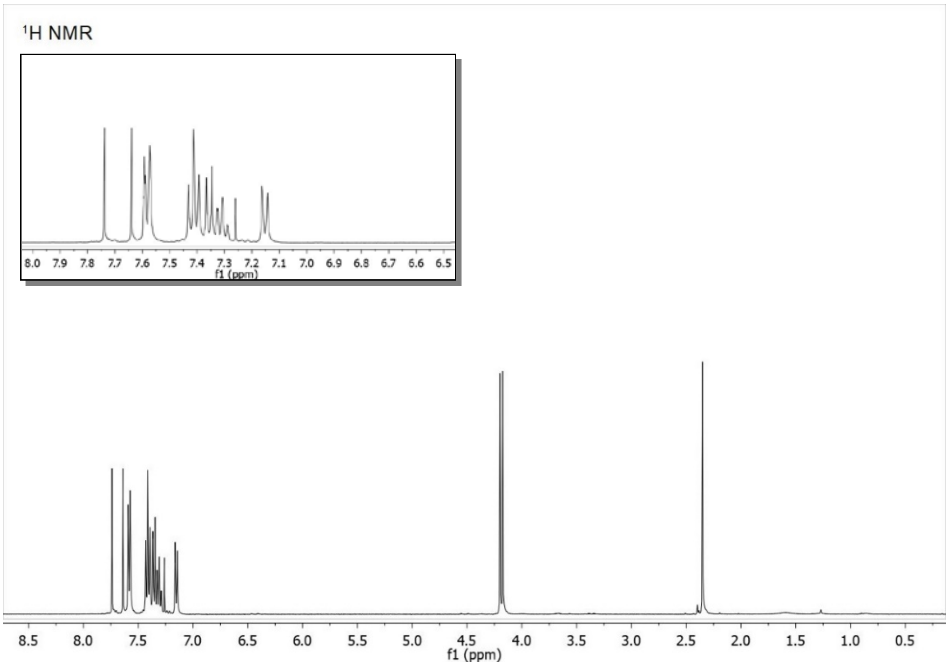

Figure S1a: ^{1}H NMR Spectrum of **4** ($CDCl_3$). Enlargement: aromatic region.

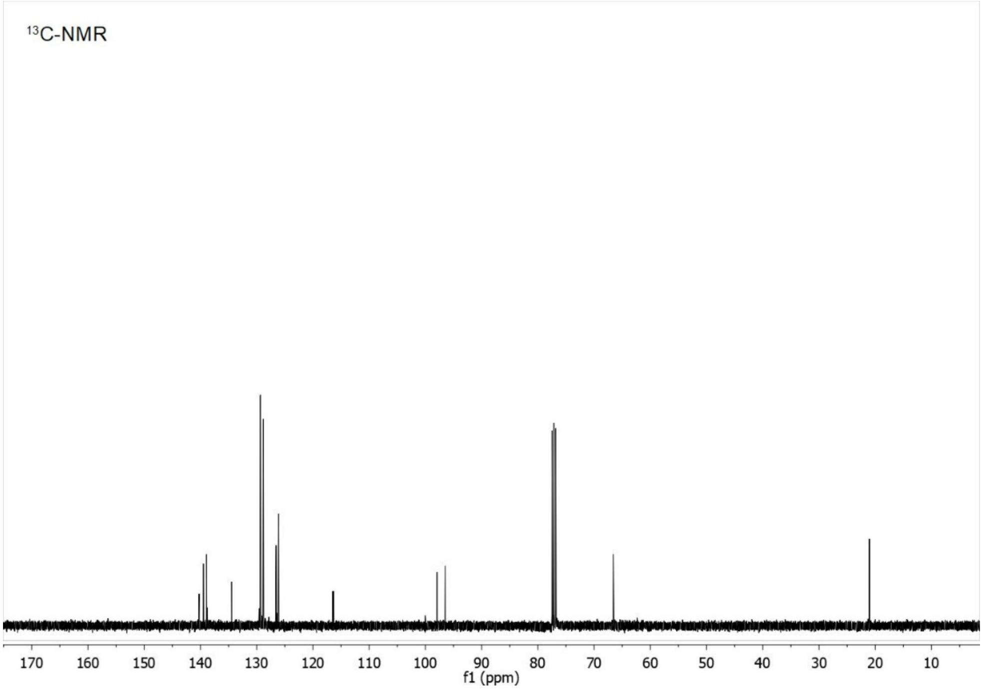

Figure S1b: ^{13}C NMR Spectrum of **4** ($CDCl_3$).

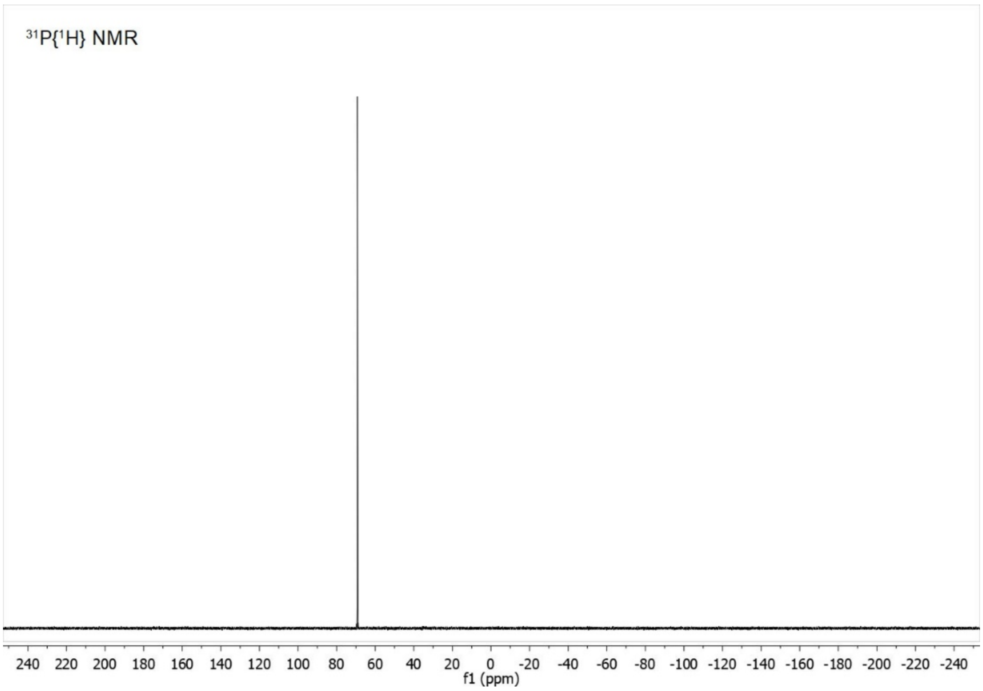

Figure S1c: ^{31}P{^{1}H} NMR Spectrum of **4** ($CDCl_3$).

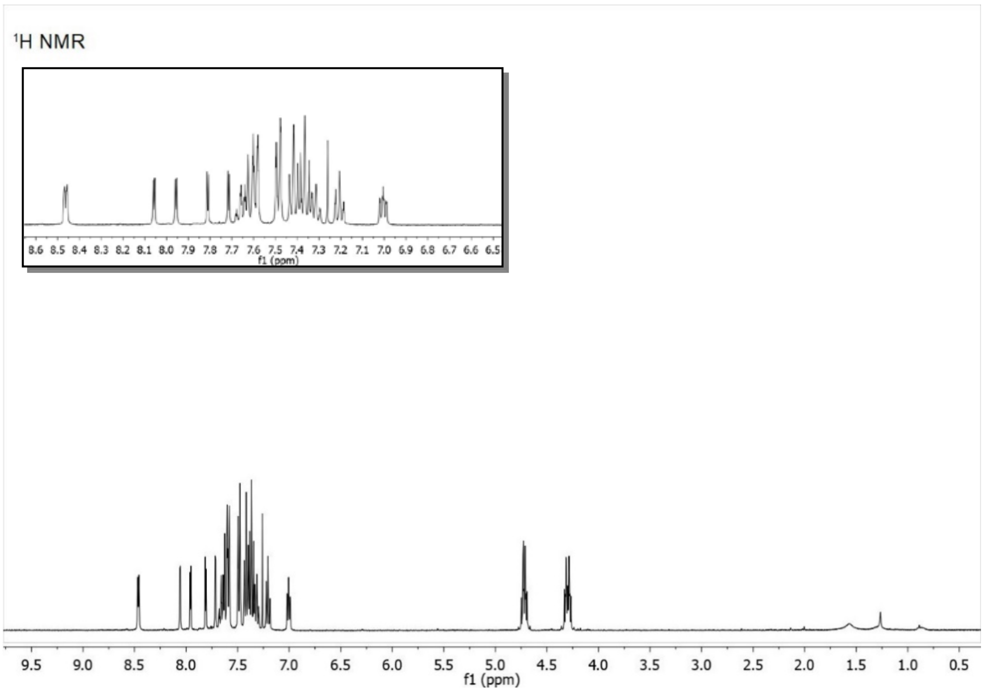

Figure S2a: ^{1}H NMR Spectrum of **5** ($CDCl_3$). Enlargement: aromatic region.

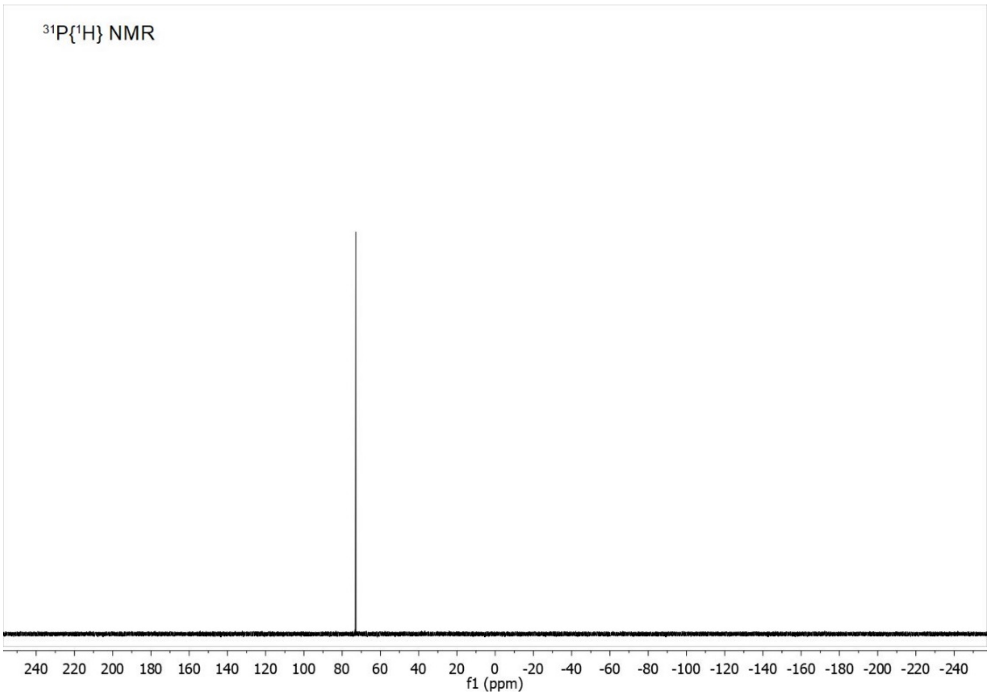

Figure S2b: $^{31}P\{^{1}H\}$ NMR Spectrum of **5** ($CDCl_3$).

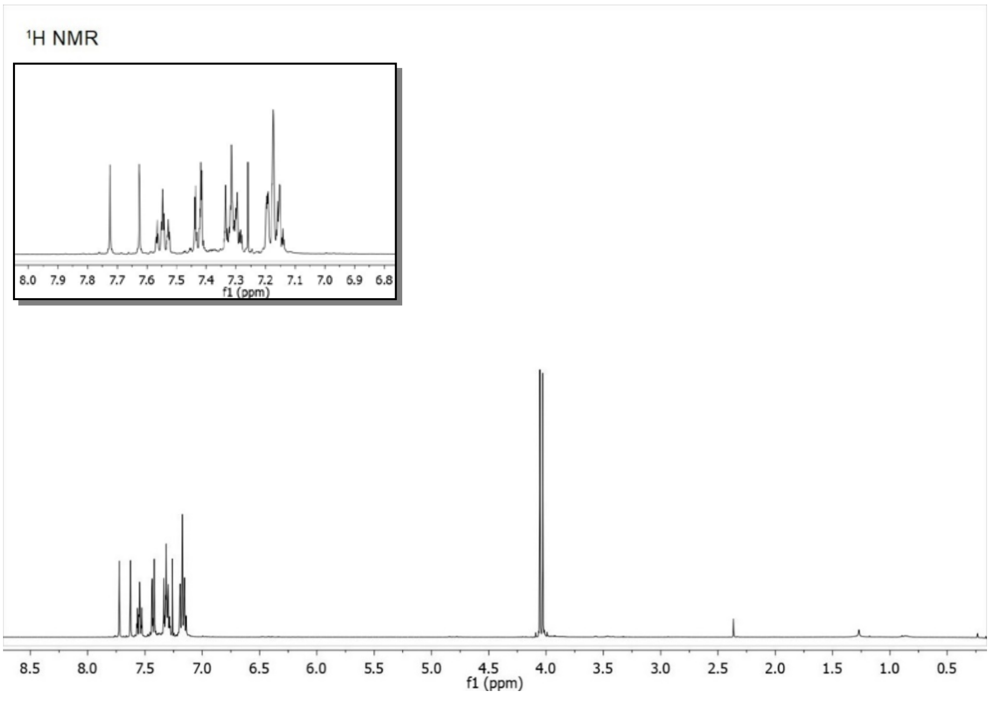

Figure S3a: ^{1}H NMR Spectrum of **6** ($CDCl_3$). Enlargement: aromatic region.

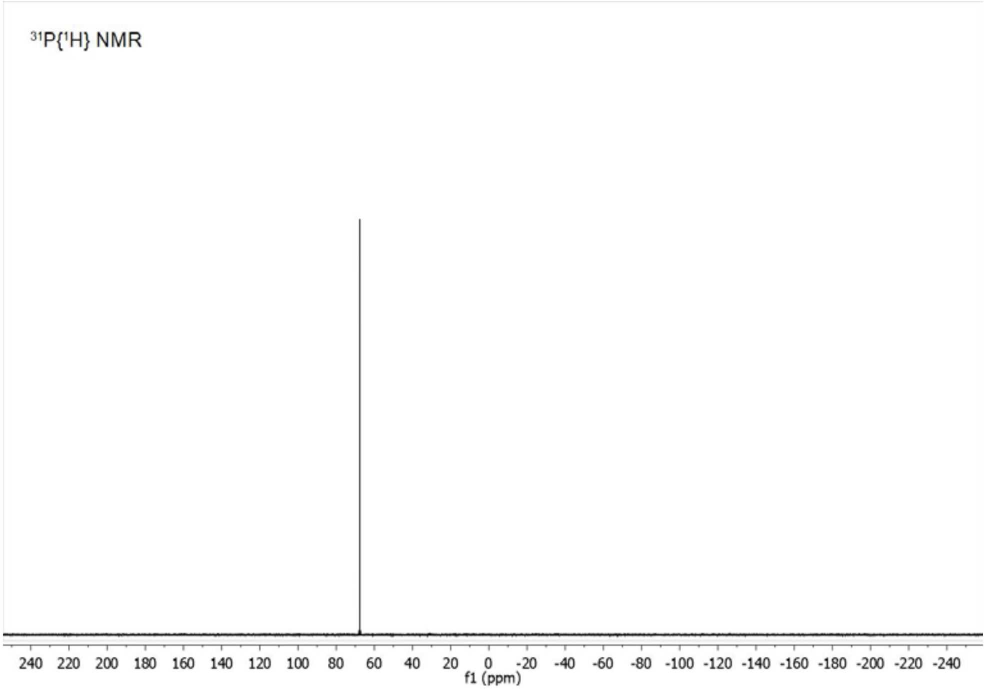

Figure S3b: $^{31}P\{^1H\}$ NMR Spectrum of **6** ($CDCl_3$).

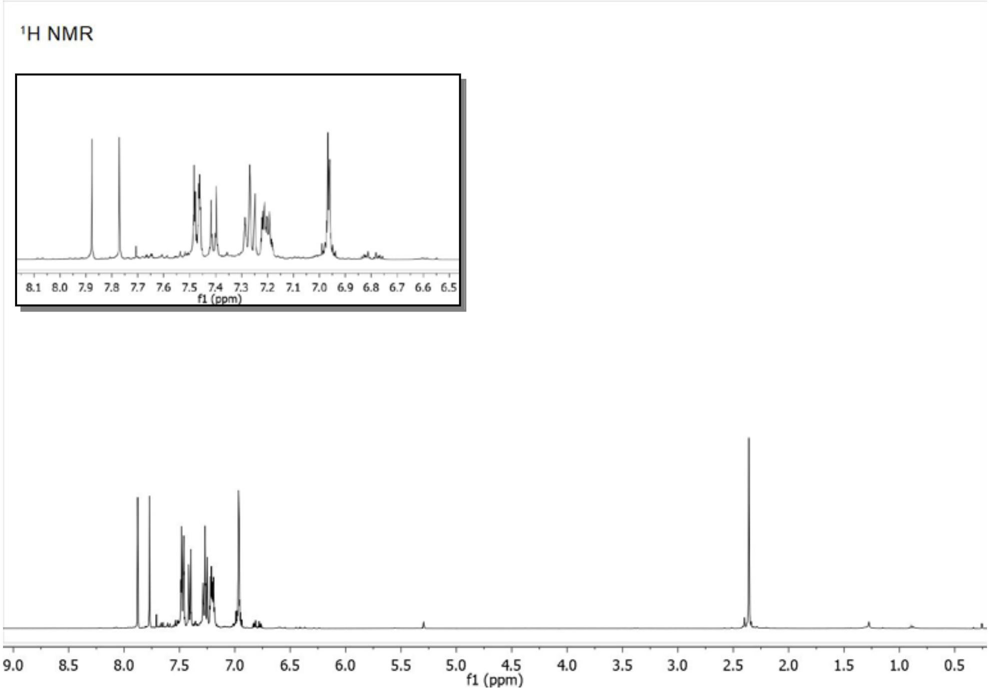

Figure S4a: ^1H NMR Spectrum of **7** (CD_2Cl_2). Enlargement: aromatic region.

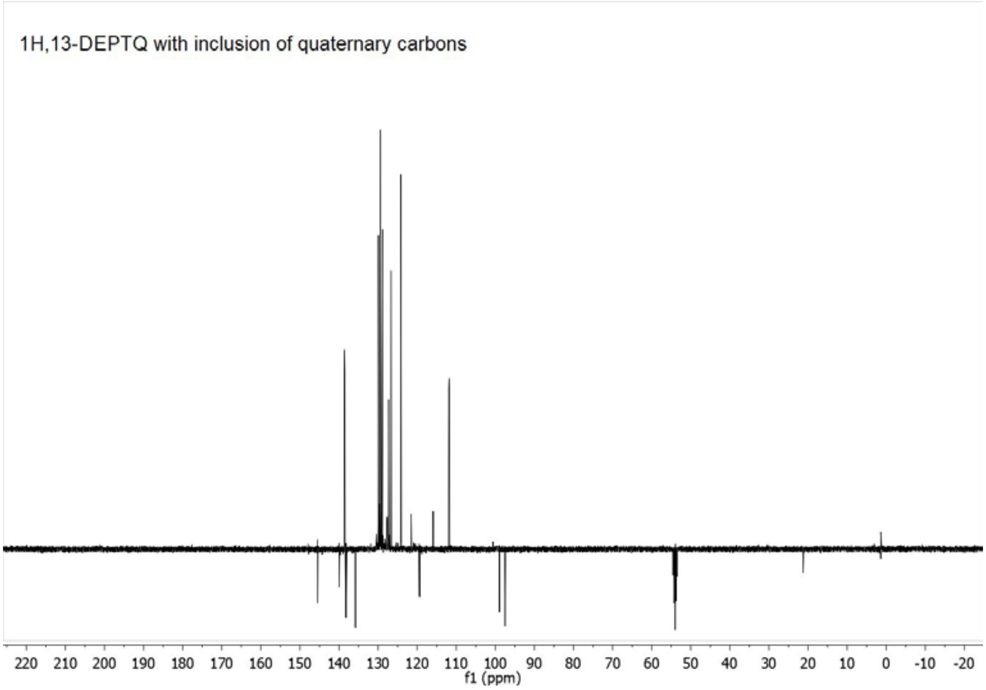

Figure S4b: ^{13}C NMR Spectrum of **7** (CD_2Cl_2).

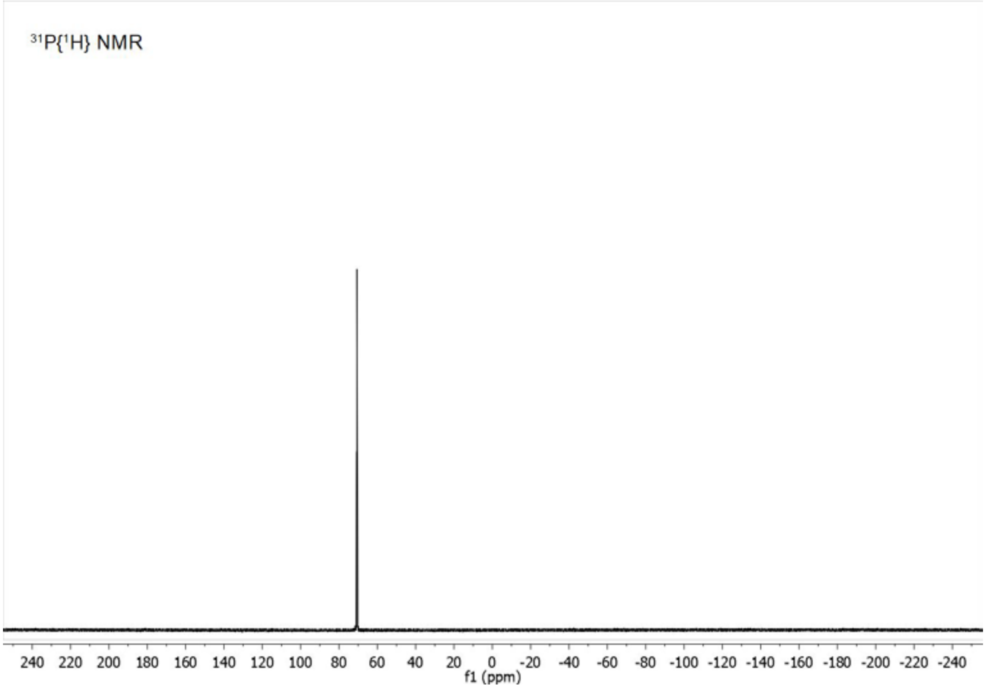

Figure S4c: $^{31}P\{^1H\}$ NMR Spectrum of **7** (CD_2Cl_2).

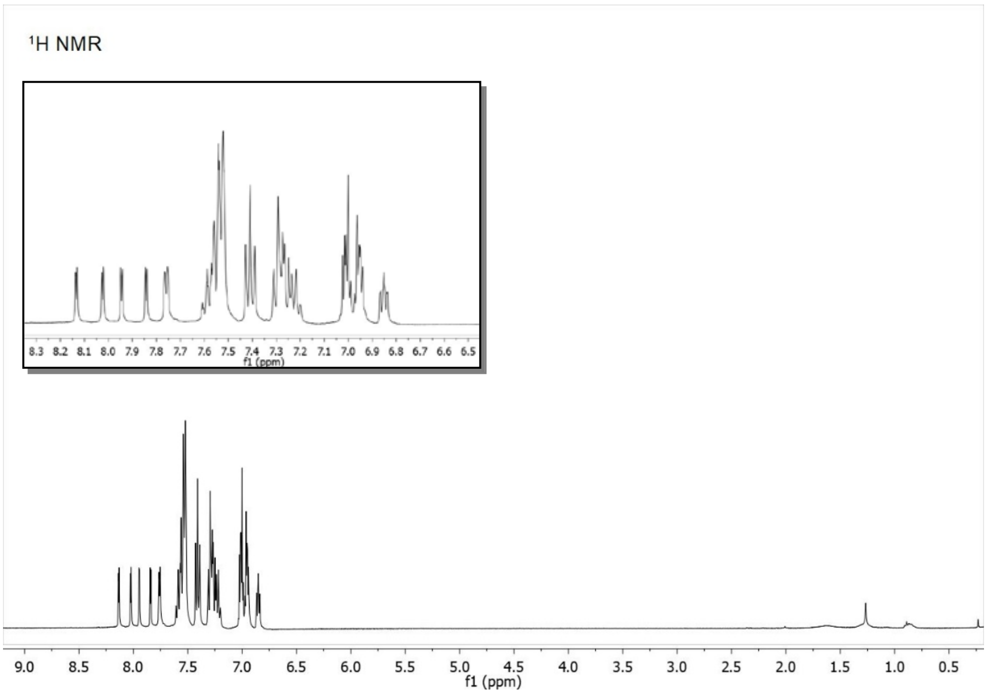

Figure S5a: ^{1}H NMR Spectrum of **8** ($CDCl_3$). Enlargement: aromatic region.

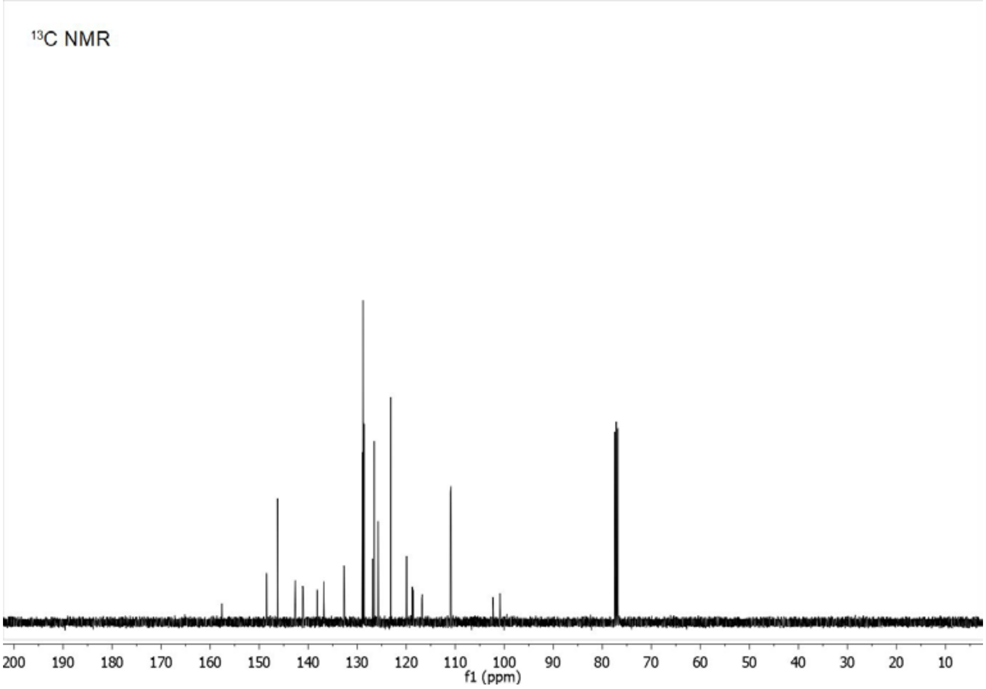

Figure S5b: ^{13}C NMR Spectrum of **8** ($CDCl_3$).

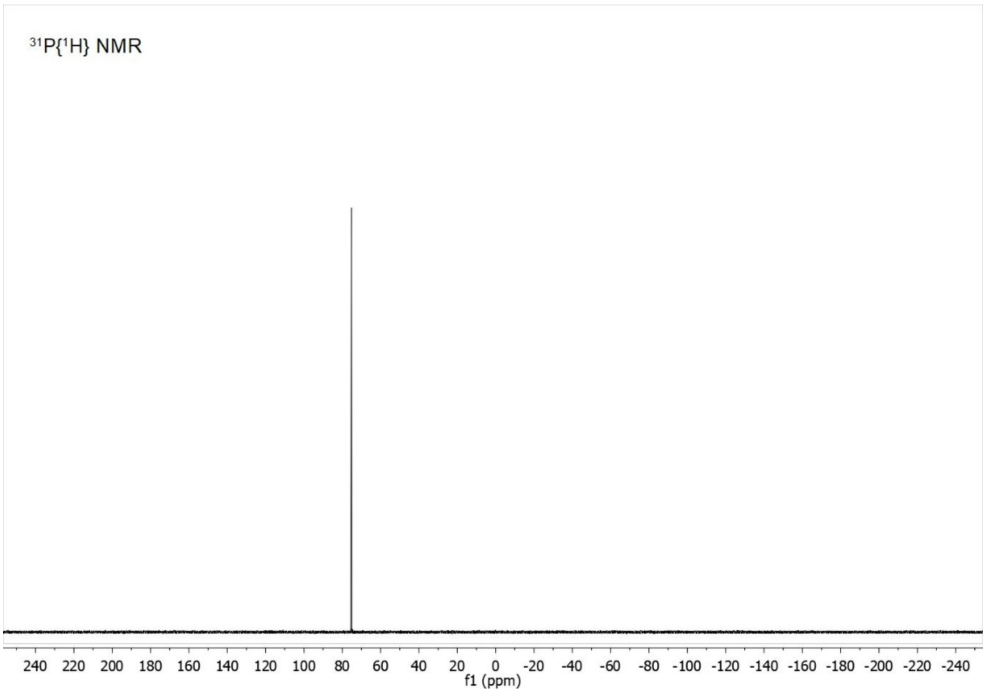

Figure S4c: $^{31}P\{^{1}H\}$ NMR Spectrum of **8** ($CDCl_3$).

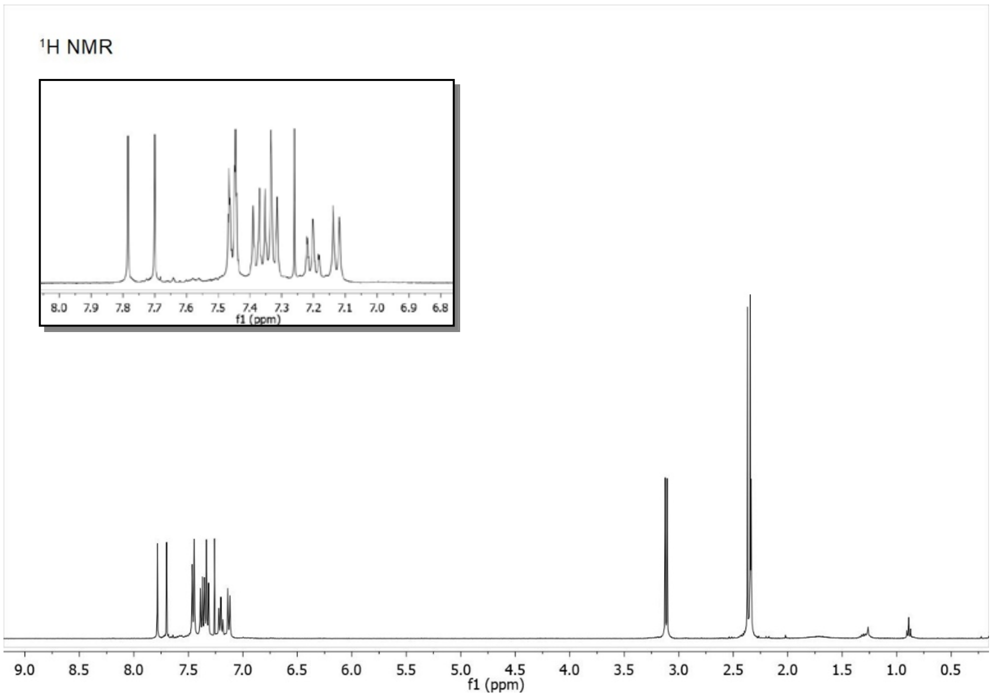

Figure S6a: ^{1}H NMR Spectrum of **9** ($CDCl_3$). Enlargement: aromatic region.

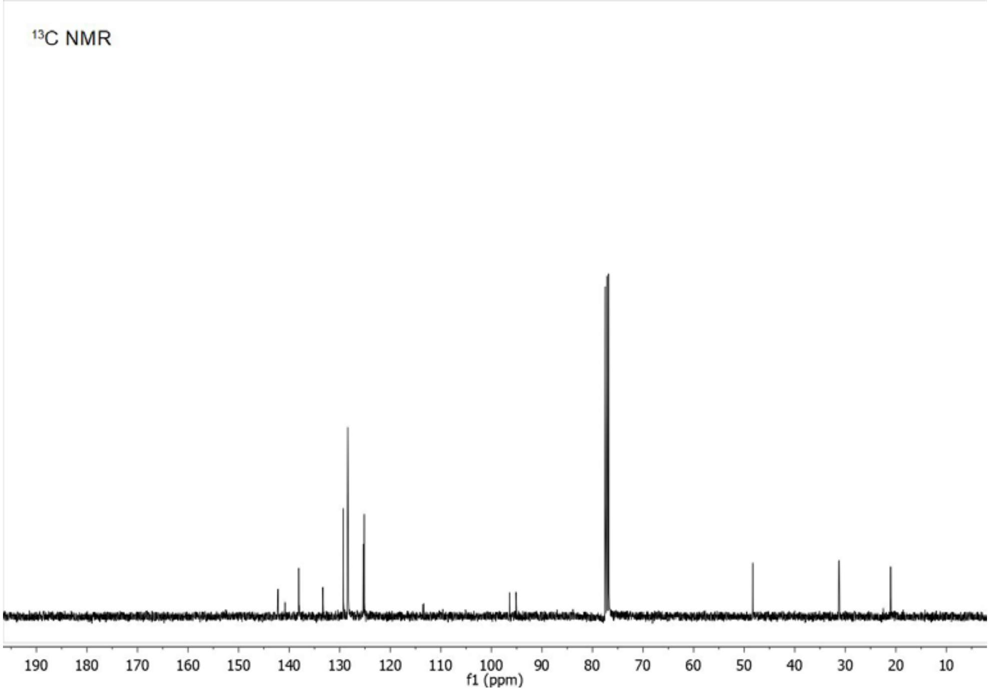

Figure S6b: ^{13}C NMR Spectrum of **9** ($CDCl_3$).

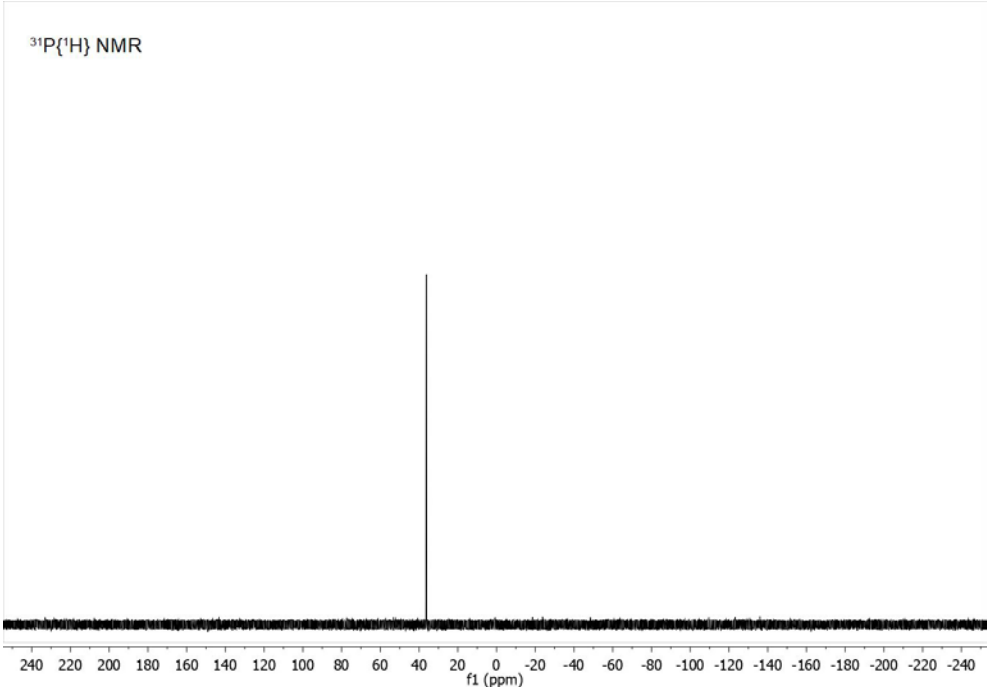

Figure S6c: $^{31}P\{^1H\}$ NMR Spectrum of **9** ($CDCl_3$).

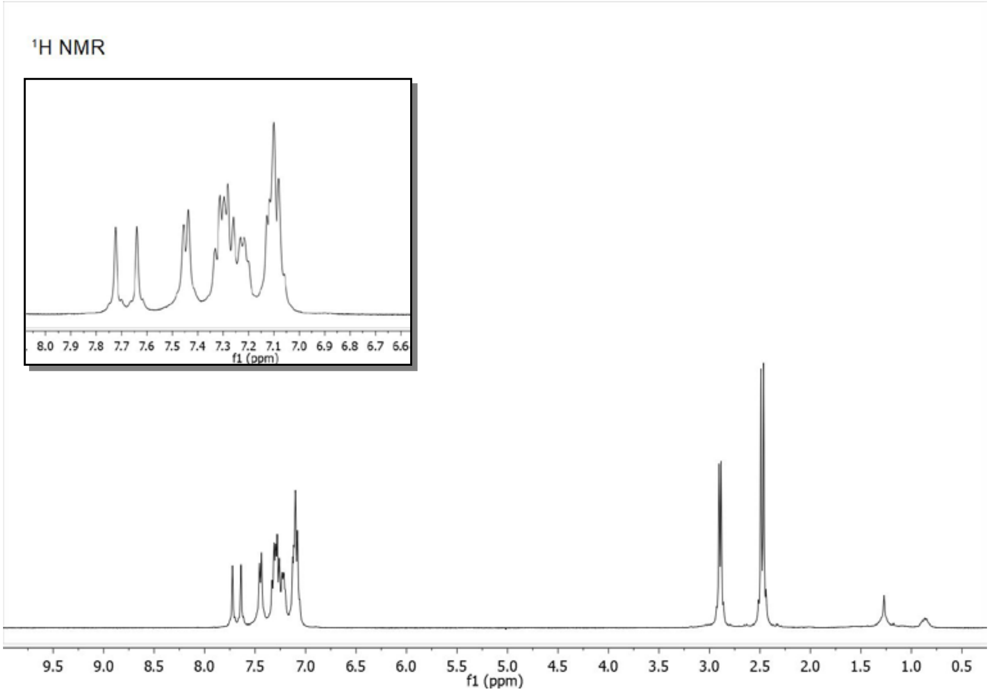

Figure S7a: ^{1}H NMR Spectrum of **10** ($CDCl_3$). Enlargement: aromatic region.

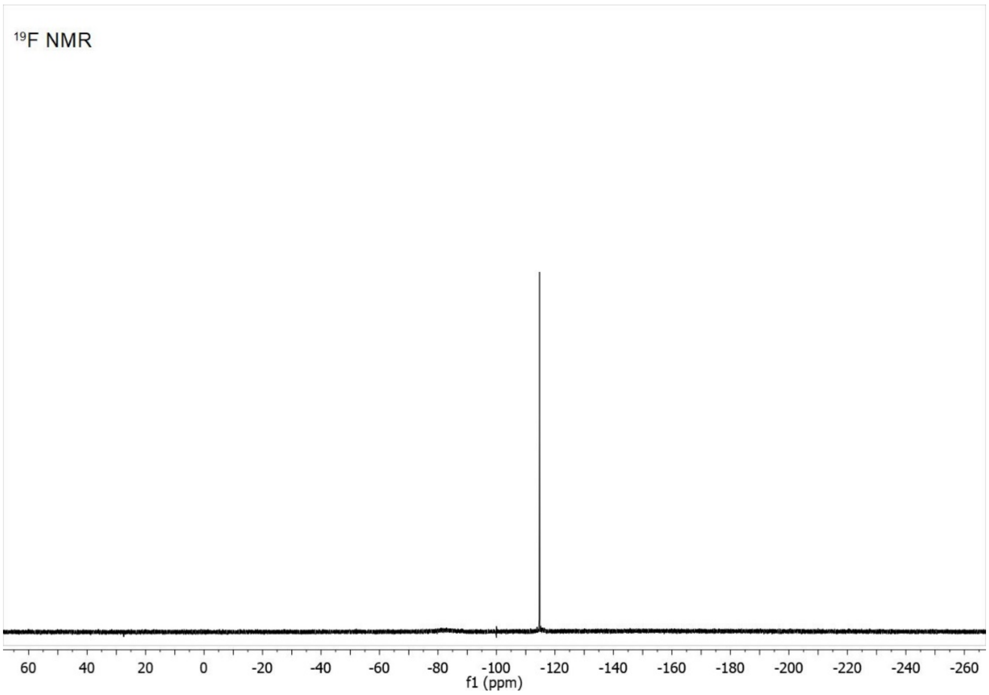

Figure S7b: ^{13}C NMR Spectrum of **10** ($CDCl_3$).

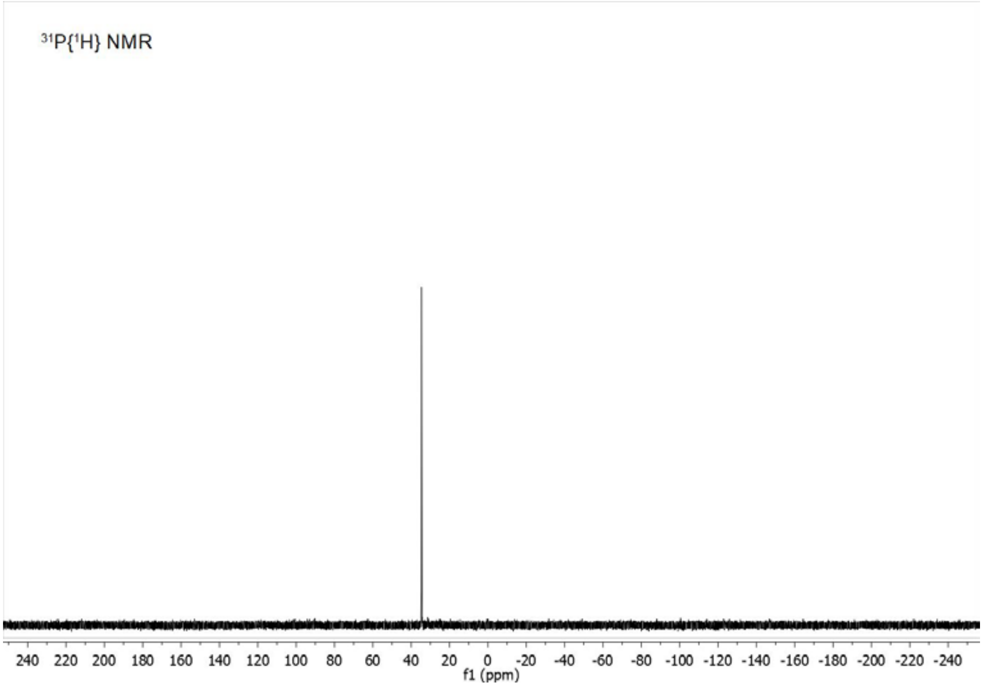

Figure S7c: $^{31}P\{^1H\}$ NMR Spectrum of **10** ($CDCl_3$).

Photophysical properties.

Compound 3

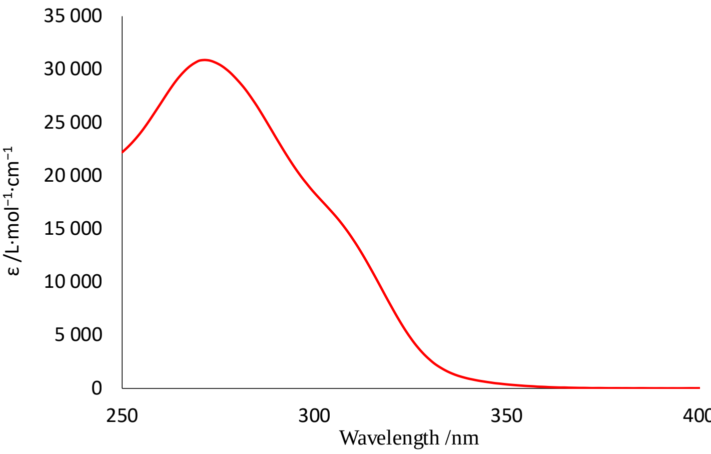

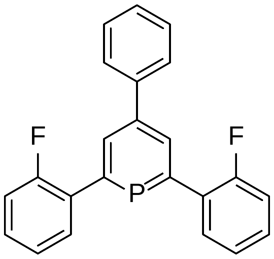

Figure S8: Absorption spectrum recorded in CH_2Cl_2 (c = 10^{-5} M) at room temperature.

Compound 4

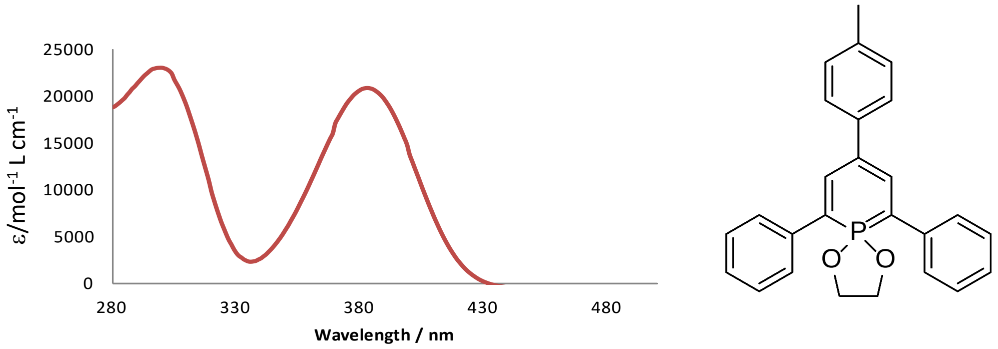

Figure S9: Absorption spectrum recorded in CH_2Cl_2 ($c = 10^{-5}$ M) at room temperature.

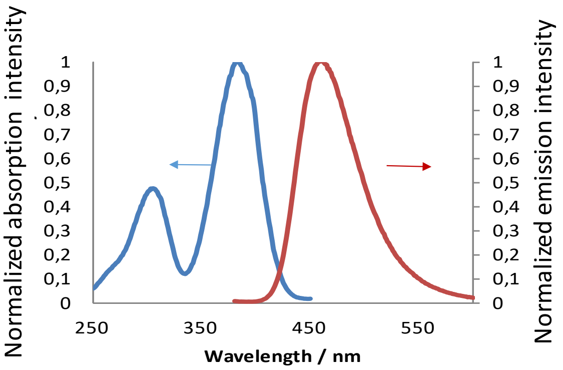

Figure S10: Emission and excitation spectra recorded in CH_2Cl_2 ($c = 10^{-5}$ M, excitation: 383 nm, emission: 457 nm) at room temperature.

Compound 5

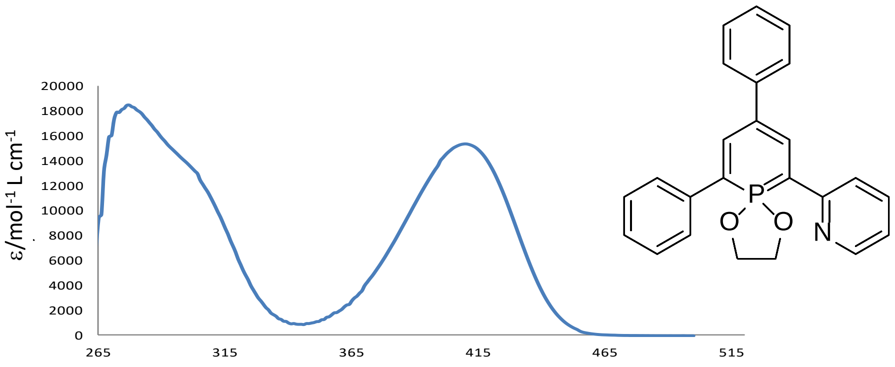

Figure S11: Absorption spectrum recorded in CH_2Cl_2 ($c = 10^{-5}$ M) at room temperature.

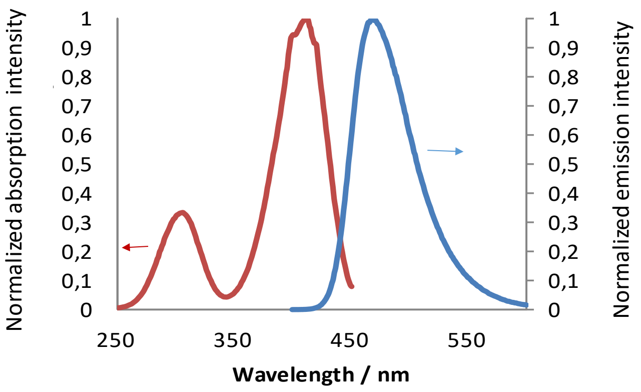

Figure S12: Emission and excitation spectra recorded in CH_2Cl_2 (c = 10^{-5} M, excitation: 410 nm, emission 469 nm) at room temperature.

<u>Compound 6</u>

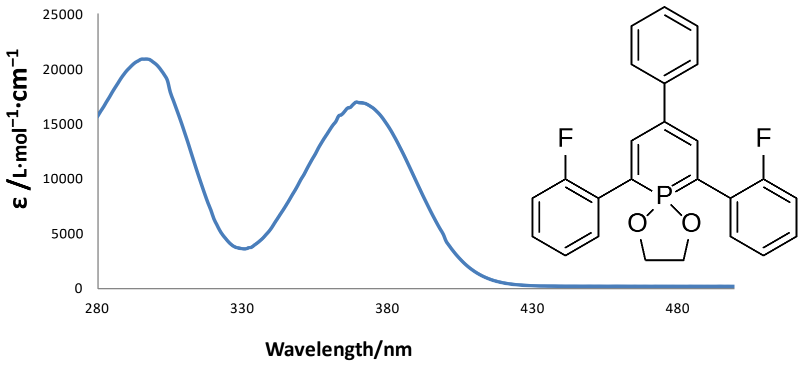

Figure S13: Absorption spectrum recorded in CH_2Cl_2 (c = 10^{-5} M) at room temperature.

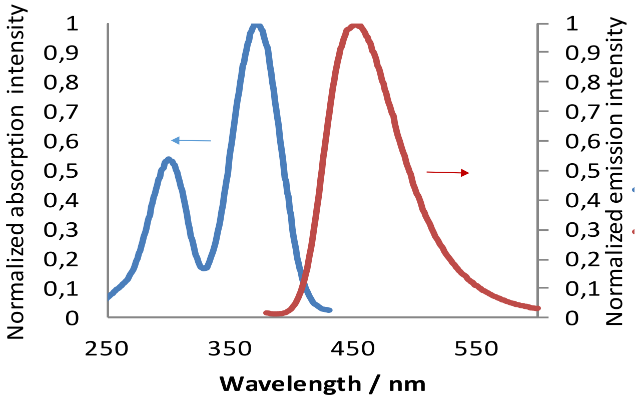

Figure S14: Emission and excitation spectra recorded in CH_2Cl_2 (c = 10^{-5} M, excitation: 369 nm, emission: 452 nm) at room temperature

Compound 7

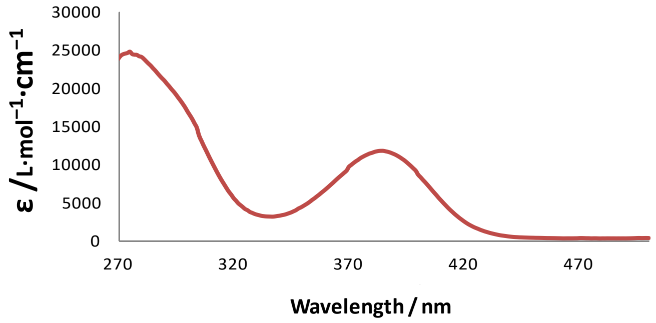

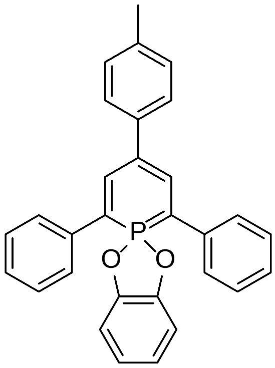

Figure S15: Absorption spectrum recorded in CH_2Cl_2 (c = 10^{-5} M) at room temperature.

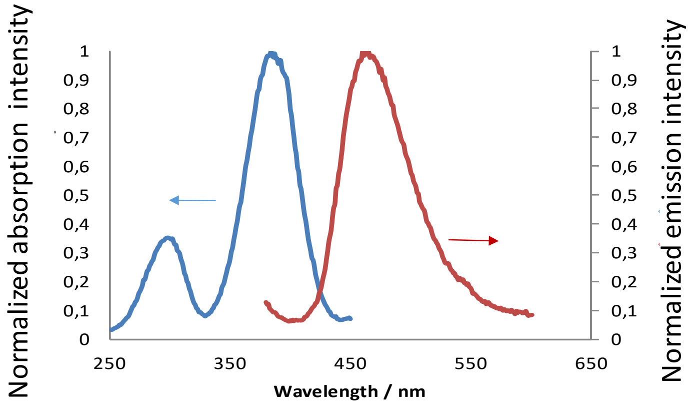

Figure S16: Emission and excitation spectra recorded in CH_2Cl_2 (c = 10^{-5} M, excitation: 384 nm, emission : 462 nm) at room temperature

Compound 8

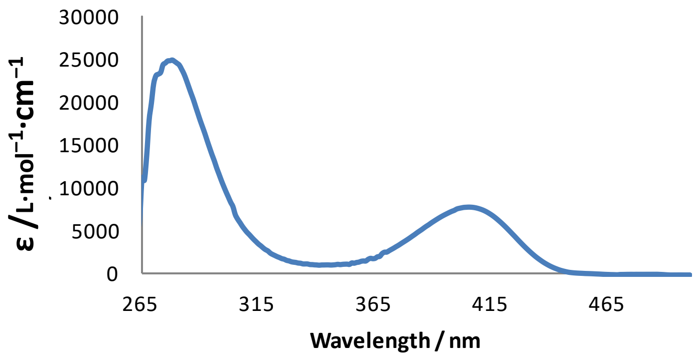

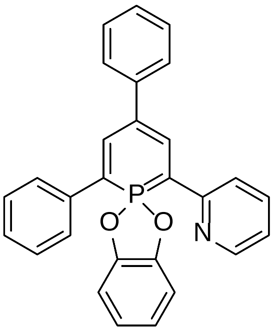

Figure S17: Absorption spectrum recorded in CH_2Cl_2 (c = 10^{-5} M) at room temperature.

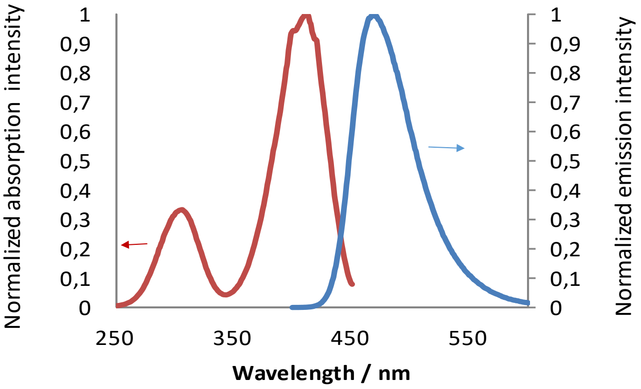

Figure S18: Emission and excitation spectra recorded in CH_2Cl_2 (c = 10^{-5} M, excitation: 405 nm, emission: 465 nm) at room temperature.

Compound 9

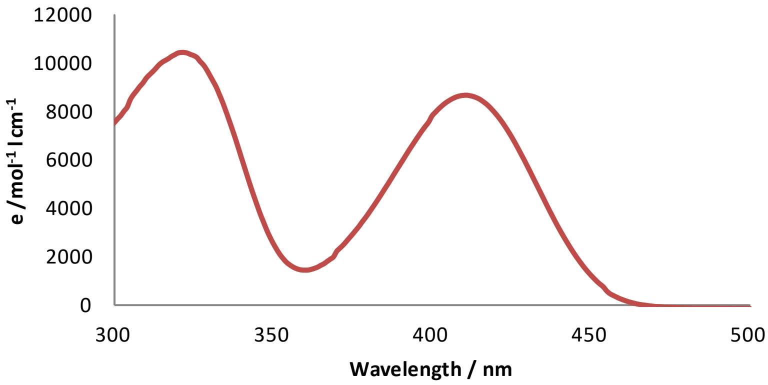

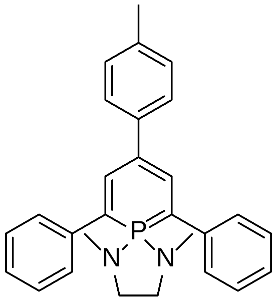

Figure S19: Absorption spectrum recorded in CH_2Cl_2 (c = 10^{-5} M) at room temperature.

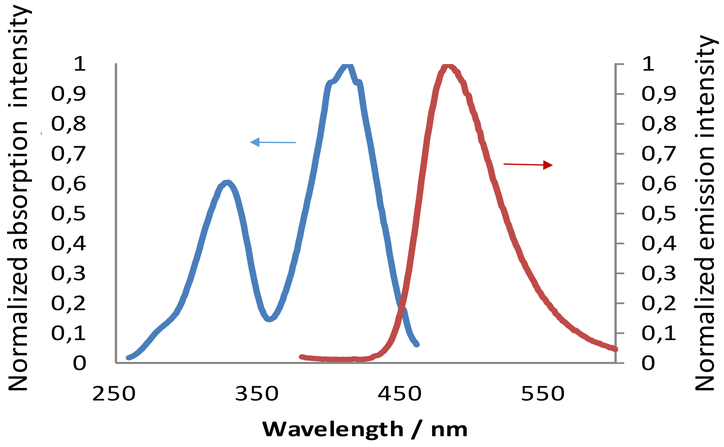

Figure S20: Emission and excitation spectra recorded in CH_2Cl_2 (c = 10^{-5} M, excitation: 411 nm, emission: 482 nm) at room temperature.

Compound 10

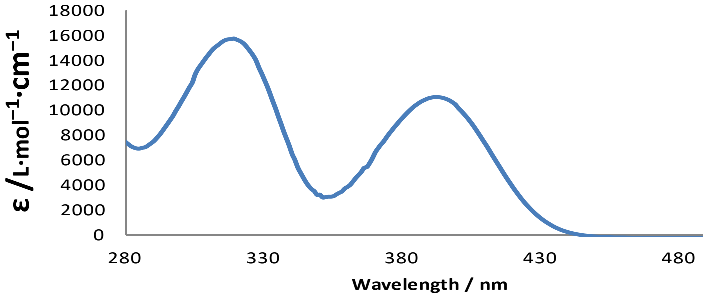

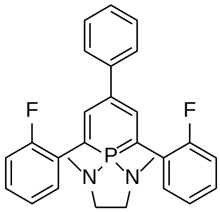

Figure S21: Absorption spectrum recorded in CH_2Cl_2 (c = 10^{-5} M) at room temperature.

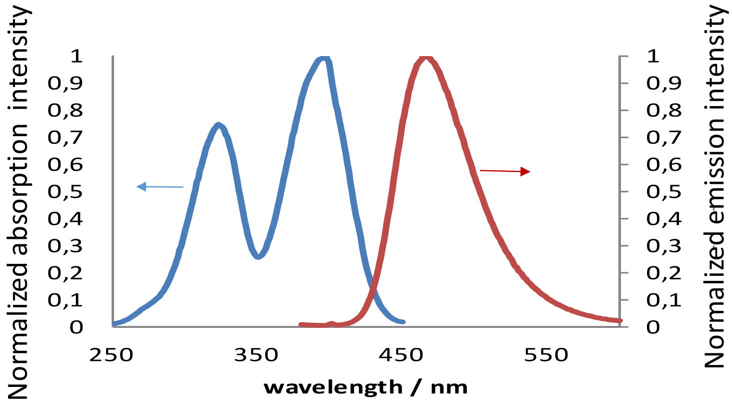

Figure S22: Emission and excitation spectra recorded in CH_2Cl_2 (c = 10^{-5} M, excitation: 402 nm, emission: 465) at room temperature.

Computational Details

Figure S23 The HOMO and LUMO orbitals of molecules **3**-**10** at the B3LYP/6-31+G level of theory

	**3**	**4**	**5**	**6**	**7**	**8**	**9**	**10**
**LUMO**	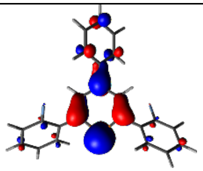 -2.04	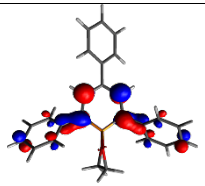 -1.19	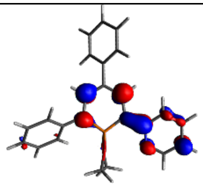 -1.51	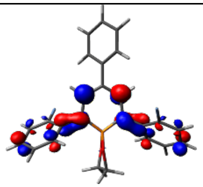 -1.31	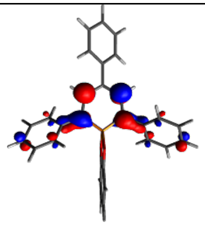 -1.40	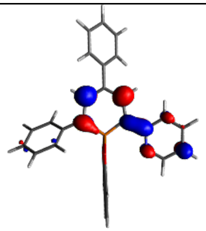 -1.68	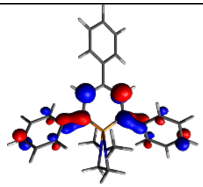 -1.09	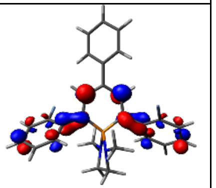 -1.16
**HOMO**	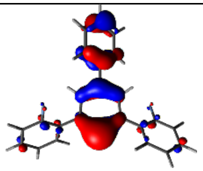 -6.27	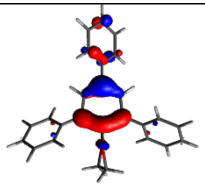 -5.18	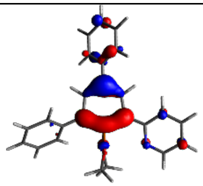 -5.17	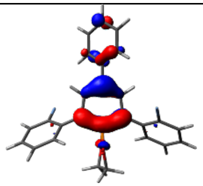 -5.27	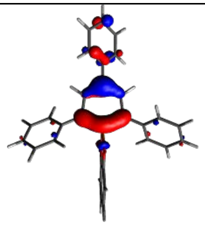 -5.44	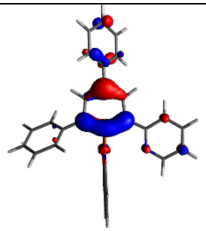 -5.41	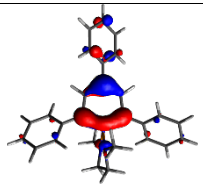 -4.83	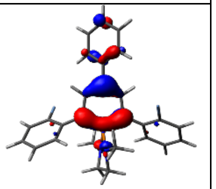 -4.91
**Δ**	4.23	3.99	3.66	3.96	4.04	3.73	3.74	3.75

Table S1a Relative energies of the rotational isomers of **3** at different levels of theory in kcal/mol

	**B3LYP/ 6-31+G***	**B3LYP/ cc-pVTZ**	**M06-2X/ 6-31+G***	**M06-2X/ cc-pVTZ**	**wB97xd/ 6-31+G***	**wB97xd/ cc-pVTZ**
**3_a**	0.00	0.00	0.00	0.00	0.00	0.00
**3_b**	0.37	0.32	0.42	0.45	0.39	0.33
**3_c**	0.53	0.55	0.73	0.79	0.62	0.64
**3_d**	0.79	0.79	0.82	0.89	0.75	0.73
**3_e**	1.13	1.05	1.10	1.11	1.03	0.91
**3_f**	1.23	1.21	1.37	1.44	1.18	1.12
**3_g**	1.24	1.22	1.33	1.45	1.26	1.18
**3_h**	1.88	1.92	1.78	1.93	1.99	1.98
**3_i**	2.13	2.05	2.06	2.12	2.01	1.92
**3_j**	2.18	2.12	2.11	2.16	2.15	2.06

Table S1b Rotational barriers characterized by the relative energies of the stationery points for the aryl rings of **3** at the B3LYP/6-31+G* level of theory in kcal/mol (Bold border connects the minimum and the transition state toward which the geometry of the excited state minima changed)

	ω		θ		ϕ(≈ω)	
	Dihedral angle (°)	Relative energy (kcal/mol)	Dihedral angle (°)	Relative energy (kcal/mol)	Dihedral angle (°)	Relative energy (kcal/mol)
	56.0	0.8	40.8	0.0	56.0	0.8
TS_a	87.9	1.3	91.1	2.5	87.9	1.3
	131.2	0.0	138.2	0.5	131.2	0.0
TS_b	181.6	3.7	178.5	3.1	181.6	3.7
	230.8	0.4	220.8	0.0	230.8	0.4
TS_c	275.4	1.5	271.1	2.5	275.4	1.5
	304.9	1.2	318.2	0.5	304.9	1.2
TS_d	360.2	4.9	358.5	3.1	360.2	4.9

Dihedral angles in the TD-DFT optimised excited state geometry

154.6	209.5	154.6

Table S2 NICS(0) and NICS(1) values for the phosphinine ring in molecules **3**-**10** at the B3LYP/6-31+G level of theory. The two NICS(1) values refer to gauge points placed above and below the ring. The small difference in the non-symmetrical cases show that the rotation of the substituents has a minor impact on the magnetic shielding.

	**3**	**4**	**5**	**6**	**7**	**8**	**9**	**10**
NICS(0)	-6.1	-6.7	-5.1	-6.1	-6.6	-5.4	-5.1	-4.9
NICS(1)	-8.0/-8.0	-6.4/-6.4	-5.5/-5.2	-6.0/-6.0	-6.4/-6.4	-5.4-5.5	-5.2/-5.2	-5.2/-5.2

Table S3a Relative energies, and TD-DFT results of the rotational isomers of **4** at the B3LYP/6-31+G* level of theory in kcal/mol

	**Relative energy**	**λ_a (nm)**	**Intensity**	**Transition**	**Coefficient**	**λ_e (nm)**	**Intensity**	**Transition**	**Coefficient**
**a**	0.00	354	0.3063	HOMO → LUMO	0.699	415	0.3408	HOMO ← LUMO	-0.702
**b**	0.40	355	0.3013	HOMO → LUMO	0.699				
**c**	0.42	349	0.2798	HOMO → LUMO	0.699				
**d**	0.48	349	0.2801	HOMO → LUMO	0.699				
**e**	0.74	349	0.2678	HOMO → LUMO	0.698				
**f**	0.97	349	0.2603	HOMO → LUMO	0.698				

Table S3b Rotational barriers characterized by the relative energies of the stationery points for the aryl rings of **4** at the B3LYP/6-31+G* level of theory in kcal/mol (Bold border connects the minimum and the transition state toward which the geometry of the excited state minima changed)

	ω		θ		ϕ(≈ω)	
	Dihedral angle (°)	Relative energy (kcal/mol)	Dihedral angle (°)	Relative energy (kcal/mol)	Dihedral angle (°)	Relative energy (kcal/mol)
	53.5	0.0	40.1	0.0	54.5	0.0
TS_a	92.0	0.9	91.4	2.3	92.3	0.9
	121.7	0.4	139.5	0.4	123.0	0.4
TS_b	177.2	4.3	178.9	2.6	177.2	4.3
	233.5	0.0	220.1	0.0	234.5	0.0
TS_c	272.0	0.9	271.4	2.3	272.3	0.9
	301.7	0.4	319.5	0.4	303.0	0.4
TS_d	357.2	4.3	358.9	2.6	357.2	4.3

Dihedral angles in the TD-DFT optimised excited state geometry

217.6	204.4	219.5

Table S4a Relative energies, and TD-DFT results of the rotational isomers of **5** at the B3LYP/6-31+G* level of theory in kcal/mol

	**Relative energy**	**λ_a (nm)**	**Intensity**	**Transition**	**Coefficient**	**λ_e (nm)**	**Intensity**	**Transition**	**Coefficient**
**a**	0.00	384	0.3674	HOMO → LUMO	0.701	444	0.343	HOMO ← LUMO	-0.703
**b**	0.01	384	0.3601	HOMO → LUMO	0.701	444	0.343	HOMO ← LUMO	-0.703
**c**	0.01	384	0.3601	HOMO → LUMO	0.701				
**d**	0.05	383	0.3556	HOMO → LUMO	0.700				
**e**	0.28	384	0.3607	HOMO → LUMO	0.701				
**f**	0.38	386	0.3684	HOMO → LUMO	0.701				
**g**	3.00	375	0.3098	HOMO → LUMO	0.701				
**h**	3.10	376	0.3088	HOMO → LUMO	0.701				
**i**	3.27	373	0.2896	HOMO → LUMO	0.701				
**j**	3.45	372	0.2865	HOMO → LUMO	0.700				
**k**	3.47	371	0.2964	HOMO → LUMO	0.701				
**l**	3.74	370	0.2965	HOMO → LUMO	0.701				
**m**	4.04	370	0.2818	HOMO → LUMO	0.700				

**n**	4.05	369	0.2862	HOMO → LUMO	0.700						

Table S4b1 Rotational barriers characterized by the relative energies of the stationery points for the aryl rings of **5_a** at the B3LYP/6-31+G* level of theory in kcal/mol (Bold border connects the minimum and the transition state toward which the geometry of the excited state minima changed)

	ω		θ		ϕ	
	Dihedral angle (°)	Relative energy (kcal/mol)	Dihedral angle (°)	Relative energy (kcal/mol)	Dihedral angle (°)	Relative energy (kcal/mol)
	-9.1	0.0	41.1	0.0	The molecule rotated into another isomer during the optimisation of the minima connecting to the investigated TS	
TS_a	89.4	6.1	90.2	2.1		
	144.4	3.4	138.9	0.0		
TS_b	171.4	3.8	179.9	2.6		
	211.2	3.0	221.1	0.0		
TS_c	269.5	6.0	270.2	2.1		
			318.9	0.0		
TS_d			359.9	2.6		

Dihedral angles in the TD-DFT optimised excited state geometry

-3.3	152.7	144.6

Table S4b2 Rotational barriers for the aryl rings of **5_b** characterized by the relative energies of the stationery points at the B3LYP/6-31+G* level of theory in kcal/mol (Bold border connects the minimum and the transition state toward which the geometry of the excited state minima changed)

	ω		θ		ϕ	
	Dihedral angle (°)	Relative energy (kcal/mol)	Dihedral angle (°)	Relative energy (kcal/mol)	Dihedral angle (°)	Relative energy (kcal/mol)
	-15.4	0.0	41.6	0.3	56.9	0.0
TS_a	88.9	6.6	89.4	2.3	89.7	0.7
	145.1	4.0	139.3	0.0	124.1	0.0
TS_b	168.5	4.2	181.0	2.7	179.7	3.9
	212.3	3.2	221.6	0.3	236.9	0.0
TS_c	268.7	6.0	269.4	2.3	269.7	0.7
			319.3	0.0	304.1	0.0
TS_d			361.0	2.7	359.7	3.9

Dihedral angles in the TD-DFT optimised excited state geometry

-8.0	152.5	143.6

Table S5a Relative energies, and TD-DFT results of the rotational isomers of **6** at the B3LYP/6-31+G* level of theory in kcal/mol

	**Relative energy**	**λ_a (nm)**	**Intensity**	**Transition**	**Coefficient**	**λ_e (nm)**	**Intensity**	**Transition**			**Coefficient**
**a**	0.00	360	0.2554	HOMO → LUMO	0.700	419	0.2829	HOMO	←	LUMO	0.703
**b**	0.24	358	0.2394	HOMO → LUMO	0.699						
**c**	0.35	357	0.2234	HOMO → LUMO	0.699						

**d**	0.39	351	0.2372	HOMO → LUMO	0.699						
**e**	0.41	351	0.2383	HOMO → LUMO	0.699						
**f**	0.62	352	0.2517	HOMO → LUMO	0.699						
**g**	0.66	354	0.2061	HOMO → LUMO	0.698						
**h**	0.99	355	0.2511	HOMO → LUMO	0.698						
**i**	1.02	350	0.2382	HOMO → LUMO	0.698						
**j**	1.04	354	0.2907	HOMO → LUMO	0.698						
**k**	1.12	356	0.2515	HOMO → LUMO	0.698						
**l**	1.60	354	0.2792	HOMO → LUMO	0.698						

Table S5b Rotational barriers characterized by the relative energies of the stationery points for the aryl rings of **6** at the B3LYP/6-31+G* level of theory in kcal/mol (Bold border connects the minimum and the transition state toward which the geometry of the excited state minima changed)

	ω		θ		ϕ(≈ω)	
	Dihedral angle (°)	Relative energy (kcal/mol)	Dihedral angle (°)	Relative energy (kcal/mol)	Dihedral angle (°)	Relative energy (kcal/mol)
	117.7	0.4	40.8	0.3	117.7	0.4
TS_a	176.3	5.8	89.0	2.2	176.3	5.8
	238.5	0.0	138.9	0.0	238.5	0.0
TS_b	277.9	0.8	180.5	2.8	277.9	0.8
	296.9	0.6	220.8	0.3	296.9	0.6
TS_c	359.8	5.2	269.0	2.2	359.8	5.2
			318.9	0.0		
TS_d			360.5	2.8		

Dihedral angles in the TD-DFT optimised excited state geometry

ω	θ	ϕ(≈ω)
226.0	154.5	226.0

Table S6a Relative energies, and TD-DFT results of the rotational isomers of **7** at the B3LYP/6-31+G* level of theory in kcal/mol

	**Relative energy**	**λ_a (nm)**	**Intensity**	**Transition**	**Coefficient**	**λ_e (nm)**	**Intensity**	**Transition**			**Coefficient**
**a**	0.00	349	0.2945	HOMO → LUMO	0.699	432	0.3078	HOMO	←	LUMO	0.702
**b**	0.23	348	0.2779	HOMO → LUMO	0.698						
**c**	0.23	348	0.2779	HOMO → LUMO	0.698						
**d**	0.39	349	0.2893	HOMO → LUMO	0.699						

Table S6b Rotational barriers characterized by the relative energies of the stationery points for the aryl rings of **7** at the B3LYP/6-31+G* level of theory in kcal/mol (Bold border connects the minimum and the transition state toward which the geometry of the excited state minima changed)

	ω		θ		ϕ(≈ω)	
	Dihedral angle (°)	Relative energy (kcal/mol)	Dihedral angle (°)	Relative energy (kcal/mol)	Dihedral angle (°)	Relative energy (kcal/mol)
	50.6	0.0	40.9	0.0	50.6	0.0
TS_a	90.4	0.9	91.5	2.2	90.4	0.9

	126.5	0.2	138.7	0.4	126.5	0.2
TS_b	178.6	2.9	179.0	2.7	178.9	2.9
	230.6	0.0	220.9	0.0	230.6	0.0
TS_c	270.4	0.9	271.5	2.2	270.4	0.9
	306.5	0.2	318.7	0.4	306.5	0.2
TS_d	358.6	2.9	359.0	2.7	358.9	2.9

Dihedral angles in the TD-DFT optimised excited state geometry

208.8	209.0	210.3

Table S7a Relative energies, and TD-DFT results of the rotational isomers of **8** at the B3LYP/6-31+G* level of theory in kcal/mol

	Relative energy	λ_a (nm)	Intensity	Transition	Coefficient	λ_e (nm)	Intensity	Transition			Coefficient
**a**	0.00	378	0.3435	HOMO → LUMO	0.701	438	0.3261	HOMO	←	LUMO	0.703
**b**	0.20	378	0.3381	HOMO → LUMO	0.700						
**c**	2.08	377	0.3085	HOMO → LUMO	0.700						
**d**	2.16	374	0.3008	HOMO → LUMO	0.700						

Table S7b Rotational barriers characterized by the relative energies of the stationery points for the aryl rings of **8** at the B3LYP/6-31+G* level of theory in kcal/mol (Bold border connects the minimum and the transition state toward which the geometry of the excited state minima changed)

	ω		θ		ϕ	
	Dihedral angle (°)	Relative energy (kcal/mol)	Dihedral angle (°)	Relative energy (kcal/mol)	Dihedral angle (°)	Relative energy (kcal/mol)
	5.6	0.0	41.7	0.0	53.5	0.0
TS_a	89.1	5.5	90.6	2.1	90.9	0.9
	166.7	2.1	137.8	0.2	125.8	0.2
TS_b	272.2	5.7	179.4	2.8	179.7	3.2
	365.6	0.0	221.7	0.0	233.5	0.0
TS_c			270.6	2.1	270.9	0.9
			317.8	0.2	305.8	0.2
TS_d			359.4	2.8	359.7	3.2

Dihedral angles in the TD-DFT optimised excited state geometry

3.6	207.5	210.4

Table S8a Relative energies, and TD-DFT results of the rotational isomers of **9** at the B3LYP/6-31+G* level of theory in kcal/mol

	Relative energy	λ_a (nm)	Intensity	Transition	Coefficient	λ_e (nm)	Intensity	Transition			Coefficient
**a**	0.00	381	0.3231	HOMO → LUMO	0.701	436	0.3455	HOMO	←	LUMO	0.703
**b**	0.54	383	0.3124	HOMO → LUMO	0.701						
**c**	1.22	380	0.2895	HOMO → LUMO	0.701						
**d**	1.26	380	0.2895	HOMO → LUMO	0.701						
**e**	1.28	381	0.2999	HOMO → LUMO	0.702						

**f**	1.77	380	0.2884	HOMO → LUMO	0.701						

Table S8b Rotational barriers characterized by the relative energies of the stationery points for the aryl rings of **9** at the B3LYP/6-31+G* level of theory in kcal/mol (Bold border connects the minimum and the transition state toward which the geometry of the excited state minima changed)

	ω		θ		ϕ(≈ω)	
	Dihedral angle (°)	Relative energy (kcal/mol)	Dihedral angle (°)	Relative energy (kcal/mol)	Dihedral angle (°)	Relative energy (kcal/mol)
	47.5	0.0	38.5	0.0	48.3	0.0
TS_a	97.2	1.9	91.5	2.8	98.4	1.9
	131.6	1.3	141.1	0.5	132.4	1.3
TS_b	177.2	3.4	178.4	2.4	177.3	3.4
	227.5	0.0	218.5	0.0	228.3	0.0
TS_c	277.2	1.9	271.5	2.8	278.4	1.9
	311.6	1.3	321.1	0.5	312.4	1.3
TS_d	357.2	3.4	358.4	2.4	357.3	3.4

Dihedral angles in the TD-DFT optimised excited state geometry

ω	θ	ϕ(≈ω)
213.9	205.1	215.4

Table S9a Relative energies, and TD-DFT results of the rotational isomers of **10** at the B3LYP/6-31+G* level of theory in kcal/mol

	**Relative energy**	**λ_a (nm)**	**Intensity**	**Transition**	**Coefficient**	**λ_e (nm)**	**Intensity**	**Transition**			**Coefficient**
**a**	0.00	383	0.2491	HOMO → LUMO	0.701	439	0.2955	HOMO	←	LUMO	0.704
**b**	0.10	379	0.2748	HOMO → LUMO	0.700						
**c**	0.24	380	0.235	HOMO → LUMO	0.701						
**d**	0.53	378	0.2381	HOMO → LUMO	0.700						
**e**	0.58	379	0.2455	HOMO → LUMO	0.700						
**f**	0.68	371	0.1729	HOMO → LUMO	0.689						
**g**	0.70	372	0.1799	HOMO → LUMO	0.689						
**h**	0.73	380	0.2702	HOMO → LUMO	0.700						
**i**	0.79	381	0.246	HOMO → LUMO	0.701						
**j**	0.89	381	0.2138	HOMO → LUMO	0.701						
**k**	1.00	364	0.0275	HOMO → LUMO	0.692						
**l**	1.00	378	0.2357	HOMO → LUMO	0.700						
**m**	1.04	363	0.124	HOMO → LUMO	0.668						
**n**	1.04	377	0.1911	HOMO → LUMO	0.700						
**o**	1.15	372	0.2052	HOMO → LUMO	0.700						
**p**	1.17	363	0.014	HOMO → LUMO	0.700						
**q**	1.20	370	0.1966	HOMO → LUMO	0.700						
**r**	1.20	364	0.0956	HOMO → LUMO	0.664						

Table S9b Rotational barriers characterized by the relative energies of the stationery points for the aryl rings of **10** at the B3LYP/6-31+G* level of theory in kcal/mol (Bold border connects the minimum and the transition state toward which the geometry of the excited state minima changed)

	ω		θ		ϕ(≈ω)	
	Dihedral angle (°)	Relative energy (kcal/mol)	Dihedral angle (°)	Relative energy (kcal/mol)	Dihedral angle (°)	Relative energy (kcal/mol)
	77.8	0.7	38.9	0.2	77.8	0.7
TS_a	106.9	1.2	89.5	2.6	106.9	1.2
	113.7	1.2	140.5	0.0	113.7	1.2
TS_b	177.3	5.1	180.3	2.5	177.3	5.1
	236.7	0.0	218.9	0.2	236.7	0.0
TS_c	276.4	1.1	269.5	2.6	276.4	1.1
	302.4	0.5	320.5	0.0	302.4	0.5
TS_d	361.0	6.8	360.3	2.5	361.0	6.8

Dihedral angles in the TD-DFT optimised excited state geometry

222.7	154.5	222.7

Optimised geometries:

3_a (B3LYP/6-31+G*)

P	0.0000000	0.0000000	1.8442920
C	-0.3055800	1.3261920	0.7265560
C	-0.2753040	1.1930990	-0.6633400
C	0.0000000	0.0000000	-1.3522600
C	0.2753040	-1.1930990	-0.6633400
C	0.3055800	-1.3261920	0.7265560
C	0.6385640	-2.6465320	1.3301180
C	1.6050750	-2.7693080	2.3470830
C	1.9107090	-4.0010310	2.9270760
C	1.2490300	-5.1569080	2.5042250
C	0.2806090	-5.0726190	1.5015670
C	0.0000000	-3.8332910	0.9417260
C	-0.6385640	2.6465320	1.3301180
C	-1.6050750	2.7693080	2.3470830
C	-1.9107090	4.0010310	2.9270760
C	-1.2490300	5.1569080	2.5042250

C	-0.2806090	5.0726190	1.5015670
C	0.0000000	3.8332910	0.9417260
F	0.9668340	3.7845000	-0.0179130
C	0.0000000	0.0000000	-2.8404810
C	0.5669530	1.0663310	-3.5617430
C	0.5681780	1.0654520	-4.9575960
C	0.0000000	0.0000000	-5.6617620
C	-0.5681780	-1.0654520	-4.9575960
C	-0.5669530	-1.0663310	-3.5617430
F	-0.9668340	-3.7845000	-0.0179130
H	1.0211340	1.8948910	-5.4949620
H	1.0332830	1.8887190	-3.0255810
H	-1.0332830	-1.8887190	-3.0255810
H	-1.0211340	-1.8948910	-5.4949620
H	0.0000000	0.0000000	-6.7487250
H	0.5159560	-2.0687610	-1.2611560
H	-0.5159560	2.0687610	-1.2611560
H	-2.1322910	1.8757810	2.6692180
H	-2.6685120	4.0569510	3.7034650
H	-1.4821610	6.1204610	2.9488300
H	0.2625150	5.9456240	1.1530660
H	-0.2625150	-5.9456240	1.1530660
H	1.4821610	-6.1204610	2.9488300
H	2.6685120	-4.0569510	3.7034650
H	2.1322910	-1.8757810	2.6692180

3_a (B3LYP/6-31+G*, excited state minima)

P	0.0000000	0.0000000	1.9287300
C	0.0000000	1.3890500	0.7716590
C	-0.0317090	1.2281940	-0.6338120
C	0.0000000	0.0000000	-1.3359310

C	0.0317090	-1.2281940	-0.6338120
C	0.0000000	-1.3890500	0.7716590
C	0.0378920	-2.7207520	1.3599310
C	0.5674180	-2.9353290	2.6690330
C	0.6277470	-4.1909380	3.2534360
C	0.1423930	-5.3170160	2.5694650
C	-0.4113810	-5.1587860	1.2947690
C	-0.4574520	-3.8979510	0.7274000
C	-0.0378920	2.7207520	1.3599310
C	-0.5674180	2.9353290	2.6690330
C	-0.6277470	4.1909380	3.2534360
C	-0.1423930	5.3170160	2.5694650
C	0.4113810	5.1587860	1.2947690
C	0.4574520	3.8979510	0.7274000
F	1.0607530	3.8049480	-0.4873400
C	0.0000000	0.0000000	-2.8095400
C	0.5674690	1.0666850	-3.5446930
C	0.5661520	1.0654130	-4.9380780
C	0.0000000	0.0000000	-5.6468710
C	-0.5661520	-1.0654130	-4.9380780
C	-0.5674690	-1.0666850	-3.5446930
F	-1.0607530	-3.8049480	-0.4873400
H	1.0208960	1.8953040	-5.4737310
H	1.0449110	1.8886460	-3.0187040
H	-1.0449110	-1.8886460	-3.0187040
H	-1.0208960	-1.8953040	-5.4737310
H	0.0000000	0.0000000	-6.7336620
H	0.1505530	-2.1223940	-1.2330940
H	-0.1505530	2.1223940	-1.2330940
H	-0.9640190	2.0794410	3.2053590
H	-1.0582690	4.3006780	4.2447950

H	-0.1834380	6.3024390	3.0241610
H	0.8230120	5.9967590	0.7409880
H	-0.8230120	-5.9967590	0.7409880
H	0.1834380	-6.3024390	3.0241610
H	1.0582690	-4.3006780	4.2447950
H	0.9640190	-2.0794410	3.2053590

3_a (B3LYP/cc-pVTZ)

P	0.0000000	0.0000000	1.8354520
C	-0.0007090	1.3573680	0.7234020
C	0.0000000	1.2184290	-0.6587430
C	0.0000000	0.0000000	-1.3438700
C	0.0000000	-1.2184290	-0.6587430
C	0.0007090	-1.3573680	0.7234020
C	0.0262580	-2.7133720	1.3268180
C	0.9216070	-3.0416460	2.3532170
C	0.9409000	-4.3026590	2.9322450
C	0.0534610	-5.2801950	2.4990340
C	-0.8519470	-4.9886090	1.4870760
C	-0.8482570	-3.7257470	0.9251540
C	-0.0262580	2.7133720	1.3268180
C	-0.9216070	3.0416460	2.3532170
C	-0.9409000	4.3026590	2.9322450
C	-0.0534610	5.2801950	2.4990340
C	0.8519470	4.9886090	1.4870760
C	0.8482570	3.7257470	0.9251540
F	1.7589810	3.4691920	-0.0419190
C	0.0000000	0.0000000	-2.8273420
C	0.7783710	0.9147450	-3.5458970
C	0.7800890	0.9127860	-4.9341010
C	0.0000000	0.0000000	-5.6345520

C	-0.7800890	-0.9127860	-4.9341010
C	-0.7783710	-0.9147450	-3.5458970
F	-1.7589810	-3.4691920	-0.0419190
H	1.3980270	1.6215760	-5.4692310
H	1.4061930	1.6158640	-3.0127240
H	-1.4061930	-1.6158640	-3.0127240
H	-1.3980270	-1.6215760	-5.4692310
H	0.0000000	0.0000000	-6.7162460
H	0.0355720	-2.1206920	-1.2554550
H	-0.0355720	2.1206920	-1.2554550
H	-1.6219170	2.2873920	2.6846480
H	-1.6519820	4.5215660	3.7166600
H	-0.0627220	6.2659510	2.9433400
H	1.5642290	5.7189960	1.1303350
H	-1.5642290	-5.7189960	1.1303350
H	0.0627220	-6.2659510	2.9433400
H	1.6519820	-4.5215660	3.7166600
H	1.6219170	-2.2873920	2.6846480

3_a (M06-2X/6-31+G*)

P	0.0000000	0.0000000	1.8568260
C	0.0000000	1.3488210	0.7441250
C	-0.0068050	1.2197510	-0.6428760
C	0.0000000	0.0000000	-1.3277170
C	0.0068050	-1.2197510	-0.6428760
C	0.0000000	-1.3488210	0.7441250
C	0.0169630	-2.7109880	1.3399160
C	0.8821610	-3.0509630	2.3896600
C	0.8850740	-4.3250950	2.9498380
C	0.0117450	-5.2999630	2.4702020
C	-0.8651480	-4.9936630	1.4327510

C	-0.8443390	-3.7174700	0.8944490
C	-0.0169630	2.7109880	1.3399160
C	-0.8821610	3.0509630	2.3896600
C	-0.8850740	4.3250950	2.9498380
C	-0.0117450	5.2999630	2.4702020
C	0.8651480	4.9936630	1.4327510
C	0.8443390	3.7174700	0.8944490
F	1.7221220	3.4415170	-0.0946570
C	0.0000000	0.0000000	-2.8134110
C	0.7860070	0.9140600	-3.5267090
C	0.7869300	0.9128940	-4.9189860
C	0.0000000	0.0000000	-5.6196410
C	-0.7869300	-0.9128940	-4.9189860
C	-0.7860070	-0.9140600	-3.5267090
F	-1.7221220	-3.4415170	-0.0946570
H	1.4093550	1.6216670	-5.4576710
H	1.4188660	1.6129370	-2.9847870
H	-1.4188660	-1.6129370	-2.9847870
H	-1.4093550	-1.6216670	-5.4576710
H	0.0000000	0.0000000	-6.7057110
H	0.0508480	-2.1291880	-1.2390020
H	-0.0508480	2.1291880	-1.2390020
H	-1.5746870	2.2972270	2.7547450
H	-1.5737720	4.5568080	3.7561840
H	-0.0101550	6.2970130	2.8998690
H	1.5681980	5.7193760	1.0373270
H	-1.5681980	-5.7193760	1.0373270
H	0.0101550	-6.2970130	2.8998690
H	1.5737720	-4.5568080	3.7561840
H	1.5746870	-2.2972270	2.7547450

3_a (M06-2X/cc-pVTZ)

P	0.0000000	0.0000000	1.8487040
C	0.0000000	1.3453660	0.7407110
C	-0.0044910	1.2143730	-0.6396350
C	0.0000000	0.0000000	-1.3216680
C	0.0044910	-1.2143730	-0.6396350
C	0.0000000	-1.3453660	0.7407110
C	0.0165450	-2.7035470	1.3363100
C	0.8739880	-3.0379190	2.3859590
C	0.8789340	-4.3058650	2.9443470
C	0.0152090	-5.2790360	2.4630850
C	-0.8537660	-4.9778450	1.4258020
C	-0.8362600	-3.7081820	0.8867070
C	-0.0165450	2.7035470	1.3363100
C	-0.8739880	3.0379190	2.3859590
C	-0.8789340	4.3058650	2.9443470
C	-0.0152090	5.2790360	2.4630850
C	0.8537660	4.9778450	1.4258020
C	0.8362600	3.7081820	0.8867070
F	1.7062900	3.4364320	-0.1020110
C	0.0000000	0.0000000	-2.8031940
C	0.7662320	0.9236900	-3.5138860
C	0.7680110	0.9218040	-4.8997350
C	0.0000000	0.0000000	-5.5971380
C	-0.7680110	-0.9218040	-4.8997350
C	-0.7662320	-0.9236900	-3.5138860
F	-1.7062900	-3.4364320	-0.1020110
H	1.3755650	1.6377130	-5.4365060
H	1.3827020	1.6310810	-2.9736380
H	-1.3827020	-1.6310810	-2.9736380
H	-1.3755650	-1.6377130	-5.4365060

H	0.0000000	0.0000000	-6.6784870
H	0.0445260	-2.1195350	-1.2341930
H	-0.0445260	2.1195350	-1.2341930
H	-1.5571360	2.2829350	2.7525930
H	-1.5612310	4.5343360	3.7507770
H	-0.0147980	6.2714600	2.8916990
H	1.5495980	5.7036240	1.0293450
H	-1.5495980	-5.7036240	1.0293450
H	0.0147980	-6.2714600	2.8916990
H	1.5612310	-4.5343360	3.7507770
H	1.5571360	-2.2829350	2.7525930

3_a (wB97xd/6-31+G*)

P	0.0000000	0.0000000	1.8365520
C	0.0000000	1.3486370	0.7283250
C	-0.0056740	1.2191510	-0.6579120
C	0.0000000	0.0000000	-1.3420520
C	0.0056740	-1.2191510	-0.6579120
C	0.0000000	-1.3486370	0.7283250
C	0.0193610	-2.7120160	1.3250900
C	0.9617130	-3.0762950	2.2963540
C	0.9731270	-4.3479910	2.8611110
C	0.0306930	-5.2940510	2.4645680
C	-0.9228870	-4.9633900	1.5058420
C	-0.9088540	-3.6902200	0.9605480
C	-0.0193610	2.7120160	1.3250900
C	-0.9617130	3.0762950	2.2963540
C	-0.9731270	4.3479910	2.8611110
C	-0.0306930	5.2940510	2.4645680
C	0.9228870	4.9633900	1.5058420
C	0.9088540	3.6902200	0.9605480

F	1.8587280	3.3893550	0.0458010
C	0.0000000	0.0000000	-2.8287740
C	0.8282360	0.8745690	-3.5427630
C	0.8289330	0.8737030	-4.9347000
C	0.0000000	0.0000000	-5.6357600
C	-0.8289330	-0.8737030	-4.9347000
C	-0.8282360	-0.8745690	-3.5427630
F	-1.8587280	-3.3893550	0.0458010
H	1.4844660	1.5522680	-5.4731430
H	1.4932600	1.5439040	-3.0028630
H	-1.4932600	-1.5439040	-3.0028630
H	-1.4844660	-1.5522680	-5.4731430
H	0.0000000	0.0000000	-6.7220260
H	0.0440630	-2.1290970	-1.2529930
H	-0.0440630	2.1290970	-1.2529930
H	-1.7050430	2.3437150	2.5980560
H	-1.7209290	4.5998330	3.6064690
H	-0.0331600	6.2885770	2.9001750
H	1.6771100	5.6718380	1.1799410
H	-1.6771100	-5.6718380	1.1799410
H	0.0331600	-6.2885770	2.9001750
H	1.7209290	-4.5998330	3.6064690
H	1.7050430	-2.3437150	2.5980560

3_a (wB97xd/cc-pVTZ)

P	0.0000000	0.0000000	1.8283960
C	0.0000000	1.3453890	0.7246890
C	-0.0043760	1.2133060	-0.6542000
C	0.0000000	0.0000000	-1.3349650
C	0.0043760	-1.2133060	-0.6542000
C	0.0000000	-1.3453890	0.7246890

C	0.0187670	-2.7038840	1.3219050
C	0.9442330	-3.0575890	2.3032550
C	0.9566550	-4.3213260	2.8682980
C	0.0316300	-5.2694430	2.4618260
C	-0.9049040	-4.9492780	1.4931400
C	-0.8945520	-3.6837570	0.9444140
C	-0.0187670	2.7038840	1.3219050
C	-0.9442330	3.0575890	2.3032550
C	-0.9566550	4.3213260	2.8682980
C	-0.0316300	5.2694430	2.4618260
C	0.9049040	4.9492780	1.4931400
C	0.8945520	3.6837570	0.9444140
F	1.8256730	3.3938670	0.0216620
C	0.0000000	0.0000000	-2.8174030
C	0.8070890	0.8857420	-3.5288260
C	0.8083530	0.8843140	-4.9136230
C	0.0000000	0.0000000	-5.6112340
C	-0.8083530	-0.8843140	-4.9136230
C	-0.8070890	-0.8857420	-3.5288260
F	-1.8256730	-3.3938670	0.0216620
H	1.4484760	1.5713620	-5.4502870
H	1.4548590	1.5655370	-2.9907180
H	-1.4548590	-1.5655370	-2.9907180
H	-1.4484760	-1.5713620	-5.4502870
H	0.0000000	0.0000000	-6.6926970
H	0.0392820	-2.1186160	-1.2482390
H	-0.0392820	2.1186160	-1.2482390
H	-1.6731450	2.3209940	2.6136190
H	-1.6919530	4.5657230	3.6215960
H	-0.0346330	6.2583960	2.8984530
H	1.6460230	5.6612020	1.1595420

H	-1.6460230	-5.6612020	1.1595420
H	0.0346330	-6.2583960	2.8984530
H	1.6919530	-4.5657230	3.6215960
H	1.6731450	-2.3209940	2.6136190

3_b (B3LYP/6-31+G*)

P	0.0249520	-1.8467990	-0.0337670
C	1.3742250	-0.7168410	-0.0986980
C	1.2236870	0.6706280	-0.1559690
C	-0.0079630	1.3446090	-0.2044040
C	-1.2249710	0.6427950	-0.1924810
C	-1.3475030	-0.7471090	-0.1317140
C	-2.7021400	-1.3656070	-0.1463730
C	-3.0132060	-2.4375280	-1.0051820
C	-4.2758270	-3.0313430	-1.0145590
C	-5.2733590	-2.5672150	-0.1530230
C	-4.9995900	-1.5096390	0.7168040
C	-3.7345630	-0.9373120	0.7011670
C	2.7415410	-1.3073160	-0.0622210
C	3.1095620	-2.3596520	-0.9224670
C	4.3833430	-2.9283560	-0.8870910
C	5.3339430	-2.4580210	0.0227950
C	5.0023190	-1.4198670	0.8958570
C	3.7275880	-0.8720310	0.8355070
F	3.4306430	0.1209170	1.7182760
C	-0.0270350	2.8310970	-0.2786850
C	0.8739820	3.5278720	-1.1036120
C	0.8525510	4.9219620	-1.1710260
C	-0.0683200	5.6494290	-0.4117910
C	-0.9684030	4.9699910	0.4139770
C	-0.9491870	3.5758010	0.4787820

F	-3.4963270	0.0768140	1.5796350
H	1.5515550	5.4393200	-1.8234430
H	1.5795930	2.9731820	-1.7166430
H	-1.6377810	3.0601480	1.1430530
H	-1.6824600	5.5257190	1.0165950
H	-0.0839360	6.7350520	-0.4628000
H	-2.1371990	1.2289540	-0.2710500
H	2.1247970	1.2789700	-0.1538920
H	2.3775730	-2.7201400	-1.6397800
H	4.6324860	-3.7338160	-1.5721950
H	6.3284820	-2.8941350	0.0570290
H	5.7088320	-1.0348440	1.6246740
H	-5.7440680	-1.1277710	1.4085310
H	-6.2598480	-3.0224860	-0.1544060
H	-4.4800570	-3.8511440	-1.6974940
H	-2.2451350	-2.7926860	-1.6864240

3_b (B3LYP/cc-pVTZ)

P	0.0239510	-1.8368200	-0.0358160
C	1.3703840	-0.7131680	-0.0998640
C	1.2178420	0.6665730	-0.1572350
C	-0.0076080	1.3372640	-0.2064370
C	-1.2189070	0.6396820	-0.1938840
C	-1.3444030	-0.7423710	-0.1331550
C	-2.6937790	-1.3605180	-0.1437740
C	-2.9977850	-2.4392680	-0.9844690
C	-4.2521760	-3.0327300	-0.9902490
C	-5.2477280	-2.5616750	-0.1430710
C	-4.9806550	-1.4973400	0.7082620
C	-3.7239470	-0.9220420	0.6917430
C	2.7316070	-1.3049400	-0.0602710

C	3.0880890	-2.3700940	-0.8975100
C	4.3523820	-2.9410050	-0.8585020
C	5.3046580	-2.4603180	0.0319470
C	4.9845970	-1.4089930	0.8810700
C	3.7191960	-0.8557670	0.8198130
F	3.4345120	0.1502220	1.6762480
C	-0.0261540	2.8192260	-0.2783070
C	0.8828970	3.5154450	-1.0826760
C	0.8613710	4.9020350	-1.1476780
C	-0.0662860	5.6237050	-0.4055290
C	-0.9739800	4.9458880	0.4000240
C	-0.9560570	3.5591940	0.4612140
F	-3.4923380	0.0972600	1.5504190
H	1.5667070	5.4189590	-1.7847870
H	1.5958650	2.9655450	-1.6820330
H	-1.6529920	3.0446320	1.1088750
H	-1.6941500	5.4978910	0.9892770
H	-0.0814430	6.7041830	-0.4542450
H	-2.1261040	1.2249770	-0.2690790
H	2.1138710	1.2735070	-0.1537450
H	2.3527420	-2.7403250	-1.5985760
H	4.5931350	-3.7570390	-1.5256160
H	6.2924390	-2.8986760	0.0689050
H	5.6943080	-1.0147500	1.5944740
H	-5.7254080	-1.1097740	1.3888310
H	-6.2284130	-3.0171560	-0.1417110
H	-4.4517740	-3.8584510	-1.6590060
H	-2.2289620	-2.8012610	-1.6530980

3_b (M06-2X/6-31+G*)

P	0.0396950	-1.8542510	-0.0563340

C	1.3694140	-0.7203720	-0.1204890
C	1.2183430	0.6633820	-0.1787060
C	-0.0132410	1.3255560	-0.2271880
C	-1.2207530	0.6191830	-0.2176450
C	-1.3267560	-0.7681130	-0.1535320
C	-2.6787310	-1.3864760	-0.1525710
C	-2.9974110	-2.4747020	-0.9771170
C	-4.2619110	-3.0561890	-0.9618780
C	-5.2469850	-2.5604180	-0.1093510
C	-4.9612970	-1.4852030	0.7282160
C	-3.6948860	-0.9254820	0.6889940
C	2.7393760	-1.2972460	-0.0704400
C	3.1224580	-2.3627140	-0.8970960
C	4.4008470	-2.9102590	-0.8367290
C	5.3346620	-2.4018880	0.0649190
C	4.9840940	-1.3484700	0.9054020
C	3.7051890	-0.8224810	0.8213130
F	3.3826910	0.1800880	1.6643870
C	-0.0455270	2.8099540	-0.2883730
C	0.8471810	3.5172440	-1.1030670
C	0.8118410	4.9082370	-1.1540430
C	-0.1146690	5.6142360	-0.3882940
C	-1.0067360	4.9199220	0.4274430
C	-0.9735180	3.5290060	0.4760280
F	-3.4373930	0.1002710	1.5289600
H	1.5049490	5.4414570	-1.7984630
H	1.5574310	2.9717000	-1.7193240
H	-1.6554880	2.9930800	1.1320050
H	-1.7257780	5.4626860	1.0342980
H	-0.1411120	6.6992870	-0.4268910
H	-2.1398980	1.1968740	-0.2953790

H	2.1182850	1.2755700	-0.1741760
H	2.3983270	-2.7518530	-1.6079510
H	4.6674140	-3.7311000	-1.4949310
H	6.3336680	-2.8234550	0.1191770
H	5.6773490	-0.9327960	1.6291900
H	-5.6952340	-1.0775980	1.4155990
H	-6.2360770	-3.0077910	-0.0920770
H	-4.4784840	-3.8926520	-1.6188430
H	-2.2343630	-2.8531070	-1.6521280

3_b (M06-2X/cc-pVTZ)

P	0.0419720	-1.8442560	-0.0554800
C	1.3672010	-0.7136130	-0.1159380
C	1.2127610	0.6634070	-0.1725440
C	-0.0143450	1.3210300	-0.2227580
C	-1.2159120	0.6163680	-0.2133970
C	-1.3228670	-0.7644420	-0.1502460
C	-2.6692470	-1.3859910	-0.1473550
C	-2.9736040	-2.4867660	-0.9503790
C	-4.2287130	-3.0730460	-0.9350240
C	-5.2187240	-2.5696100	-0.1038350
C	-4.9477580	-1.4813160	0.7109520
C	-3.6906920	-0.9144710	0.6731100
C	2.7330740	-1.2905410	-0.0664640
C	3.1076370	-2.3584600	-0.8836370
C	4.3791440	-2.9059930	-0.8269350
C	5.3143940	-2.3954590	0.0615960
C	4.9722200	-1.3397410	0.8921720
C	3.7002370	-0.8111860	0.8129430
F	3.3871660	0.1940090	1.6464760
C	-0.0484280	2.8012800	-0.2848170

C	0.8566280	3.5073510	-1.0763750
C	0.8189410	4.8917850	-1.1298670
C	-0.1208120	5.5932520	-0.3880200
C	-1.0245130	4.9008880	0.4055390
C	-0.9904660	3.5164410	0.4548210
F	-3.4499700	0.1260910	1.4897880
H	1.5213380	5.4239900	-1.7569310
H	1.5787150	2.9649260	-1.6727820
H	-1.6833300	2.9812890	1.0918660
H	-1.7543420	5.4406250	0.9935760
H	-0.1485160	6.6735290	-0.4279200
H	-2.1308830	1.1919550	-0.2892400
H	2.1073480	1.2753480	-0.1656300
H	2.3803490	-2.7499230	-1.5829600
H	4.6395210	-3.7286370	-1.4776860
H	6.3084440	-2.8171780	0.1132210
H	5.6674040	-0.9218350	1.6064490
H	-5.6871880	-1.0663620	1.3810830
H	-6.2009380	-3.0208350	-0.0860860
H	-4.4346030	-3.9191940	-1.5751720
H	-2.2046170	-2.8717600	-1.6072280

3_b (wB97xd/6-31+G*)

P	0.0274310	-1.8378250	-0.0632770
C	1.3633070	-0.7154740	-0.1223470
C	1.2186330	0.6682920	-0.1787180
C	-0.0084960	1.3365570	-0.2264010
C	-1.2194220	0.6379450	-0.2190990
C	-1.3333890	-0.7482040	-0.1571040
C	-2.6899550	-1.3604200	-0.1629210
C	-3.0348370	-2.3753200	-1.0654940

C	-4.3005150	-2.9536940	-1.0646760
C	-5.2592680	-2.5279850	-0.1481490
C	-4.9476520	-1.5260520	0.7665830
C	-3.6805640	-0.9672340	0.7401980
C	2.7326620	-1.2983510	-0.0769990
C	3.1366190	-2.2914780	-0.9793070
C	4.4126030	-2.8449650	-0.9316840
C	5.3220620	-2.4147980	0.0319250
C	4.9505490	-1.4342190	0.9473280
C	3.6742880	-0.9002810	0.8749940
F	3.3324320	0.0336400	1.7890920
C	-0.0297400	2.8221710	-0.2867400
C	0.8333030	3.5196970	-1.1401210
C	0.8109430	4.9105280	-1.1915690
C	-0.0734390	5.6273810	-0.3880220
C	-0.9358610	4.9430800	0.4662560
C	-0.9148570	3.5522240	0.5159970
F	-3.3986850	-0.0093590	1.6519970
H	1.4816780	5.4353550	-1.8659400
H	1.5113560	2.9673480	-1.7856820
H	-1.5749380	3.0263240	1.2011400
H	-1.6218610	5.4940870	1.1033330
H	-0.0905540	6.7127550	-0.4277050
H	-2.1353940	1.2202330	-0.2930620
H	2.1215820	1.2753240	-0.1732620
H	2.4310430	-2.6207380	-1.7370910
H	4.6961030	-3.6080950	-1.6497160
H	6.3190050	-2.8425530	0.0769800
H	5.6293440	-1.0832680	1.7174480
H	-5.6659730	-1.1772520	1.5009740
H	-6.2489480	-2.9743950	-0.1404150

H	-4.5377440	-3.7322790	-1.7828910
H	-2.2917370	-2.7003940	-1.7883370

3_b (wB97xd/cc-pVTZ)

P	0.0325300	-1.8295470	-0.0789120
C	1.3626940	-0.7084550	-0.1339390
C	1.2125140	0.6677360	-0.1896530
C	-0.0103620	1.3296870	-0.2373650
C	-1.2137220	0.6315250	-0.2297520
C	-1.3274180	-0.7475320	-0.1688380
C	-2.6782440	-1.3619220	-0.1639540
C	-3.0202180	-2.3855840	-1.0466820
C	-4.2771110	-2.9660260	-1.0338530
C	-5.2289480	-2.5336420	-0.1244750
C	-4.9198550	-1.5229810	0.7704800
C	-3.6617490	-0.9587250	0.7345010
C	2.7276430	-1.2898900	-0.0790600
C	3.1256700	-2.2995810	-0.9546310
C	4.3924260	-2.8550470	-0.8947680
C	5.2983680	-2.4101490	0.0546800
C	4.9330840	-1.4122710	0.9425880
C	3.6659440	-0.8730500	0.8605810
F	3.3312460	0.0765130	1.7461460
C	-0.0361720	2.8112370	-0.2904720
C	0.8362830	3.5150520	-1.1177650
C	0.8077940	4.8989510	-1.1620440
C	-0.0906660	5.6036960	-0.3759560
C	-0.9615930	4.9139530	0.4533430
C	-0.9357940	3.5301130	0.4946360
F	-3.3825940	0.0053880	1.6256090
H	1.4854630	5.4290940	-1.8174120

H	1.5273220	2.9718050	-1.7489550
H	-1.6048630	2.9988520	1.1589450
H	-1.6588310	5.4561960	1.0775510
H	-0.1125150	6.6843640	-0.4101480
H	-2.1267000	1.2106930	-0.2978820
H	2.1090960	1.2759490	-0.1812840
H	2.4202250	-2.6419310	-1.6999850
H	4.6719510	-3.6312010	-1.5929500
H	6.2884010	-2.8407600	0.1102590
H	5.6110650	-1.0492800	1.7015530
H	-5.6344240	-1.1691250	1.4995670
H	-6.2121510	-2.9826910	-0.1068090
H	-4.5133200	-3.7514200	-1.7376490
H	-2.2799310	-2.7170250	-1.7625290

3_c (B3LYP/6-31+G*)

P	0.0000000	0.0000000	1.8394780
C	0.0060850	-1.3604140	0.7215310
C	0.0139070	-1.2244270	-0.6683270
C	0.0000000	0.0000000	-1.3573600
C	-0.0139070	1.2244270	-0.6683270
C	-0.0060850	1.3604140	0.7215310
C	0.0002210	2.7218870	1.3268020
C	0.9289280	3.0771130	2.3239600
C	0.9348610	4.3457610	2.9051470
C	0.0000000	5.3035160	2.5032880
C	-0.9400580	4.9842960	1.5211510
C	-0.9199740	3.7142820	0.9595060
C	-0.0002210	-2.7218870	1.3268020
C	-0.9289280	-3.0771130	2.3239600
C	-0.9348610	-4.3457610	2.9051470

C	0.0000000	-5.3035160	2.5032880
C	0.9400580	-4.9842960	1.5211510
C	0.9199740	-3.7142820	0.9595060
F	1.8657710	-3.4283040	0.0222700
C	0.0000000	0.0000000	-2.8459590
C	-0.7906770	-0.9116650	-3.5684130
C	-0.7919010	-0.9109620	-4.9642010
C	0.0000000	0.0000000	-5.6687970
C	0.7919010	0.9109620	-4.9642010
C	0.7906770	0.9116650	-3.5684130
F	-1.8657710	3.4283040	0.0222700
H	-1.4191770	-1.6181760	-5.5010830
H	-1.4298640	-1.6092970	-3.0335300
H	1.4298640	1.6092970	-3.0335300
H	1.4191770	1.6181760	-5.5010830
H	0.0000000	0.0000000	-6.7556920
H	-0.0410040	2.1323670	-1.2659350
H	0.0410040	-2.1323670	-1.2659350
H	-1.6658510	-2.3398160	2.6299400
H	-1.6720680	-4.5856320	3.6661900
H	0.0016510	-6.2943200	2.9491600
H	1.6892560	-5.6968630	1.1902020
H	-1.6892560	5.6968630	1.1902020
H	-0.0016510	6.2943200	2.9491600
H	1.6720680	4.5856320	3.6661900
H	1.6658510	2.3398160	2.6299400

3_c (B3LYP/cc-pVTZ)

P	0.0000000	0.0000000	1.8296710
C	0.0085550	1.3573200	0.7179160
C	0.0000000	1.2187480	-0.6643020

C	0.0000000	0.0000000	-1.3490630
C	0.0000000	-1.2187480	-0.6643020
C	-0.0085550	-1.3573200	0.7179160
C	-0.0263960	-2.7126680	1.3243500
C	-0.9308240	-3.0445150	2.3415620
C	-0.9464890	-4.3037260	2.9247670
C	-0.0458160	-5.2751820	2.5053280
C	0.8689050	-4.9794600	1.5030050
C	0.8615940	-3.7184880	0.9363630
C	0.0263960	2.7126680	1.3243500
C	0.9308240	3.0445150	2.3415620
C	0.9464890	4.3037260	2.9247670
C	0.0458160	5.2751820	2.5053280
C	-0.8689050	4.9794600	1.5030050
C	-0.8615940	3.7184880	0.9363630
F	-1.7800390	3.4568510	-0.0203850
C	0.0000000	0.0000000	-2.8331270
C	0.7955610	0.8988340	-3.5524890
C	0.7976710	0.8970410	-4.9406930
C	0.0000000	0.0000000	-5.6414160
C	-0.7976710	-0.8970410	-4.9406930
C	-0.7955610	-0.8988340	-3.5524890
F	1.7800390	-3.4568510	-0.0203850
H	1.4295450	1.5936430	-5.4755520
H	1.4380100	1.5872330	-3.0202260
H	-1.4380100	-1.5872330	-3.0202260
H	-1.4295450	-1.5936430	-5.4755520
H	0.0000000	0.0000000	-6.7230660
H	0.0162200	-2.1212950	-1.2614710
H	-0.0162200	2.1212950	-1.2614710
H	1.6403520	2.2944700	2.6629470

H	1.6644310	4.5257470	3.7020370
H	0.0517150	6.2593000	2.9533610
H	-1.5916960	5.7048330	1.1573490
H	1.5916960	-5.7048330	1.1573490
H	-0.0517150	-6.2593000	2.9533610
H	-1.6644310	-4.5257470	3.7020370
H	-1.6403520	-2.2944700	2.6629470

3_c (M06-2X/6-31+G*)

P	0.0000000	0.0000000	1.8469770
C	0.0045160	1.3488310	0.7345600
C	0.0010500	1.2202780	-0.6525870
C	0.0000000	0.0000000	-1.3373140
C	-0.0010500	-1.2202780	-0.6525870
C	-0.0045160	-1.3488310	0.7345600
C	-0.0132690	-2.7094360	1.3354610
C	-0.8944710	-3.0529820	2.3705100
C	-0.8936150	-4.3237180	2.9384630
C	0.0000000	-5.2910390	2.4814380
C	0.8933050	-4.9803740	1.4595530
C	0.8690120	-3.7074460	0.9132200
C	0.0132690	2.7094360	1.3354610
C	0.8944710	3.0529820	2.3705100
C	0.8936150	4.3237180	2.9384630
C	0.0000000	5.2910390	2.4814380
C	-0.8933050	4.9803740	1.4595530
C	-0.8690120	3.7074460	0.9132200
F	-1.7629130	3.4259160	-0.0576460
C	0.0000000	0.0000000	-2.8238380
C	0.8133340	0.8884180	-3.5384600
C	0.8145920	0.8876250	-4.9306600

C	0.0000000	0.0000000	-5.6317350
C	-0.8145920	-0.8876250	-4.9306600
C	-0.8133340	-0.8884180	-3.5384600
F	1.7629130	-3.4259160	-0.0576460
H	1.4591720	1.5765990	-5.4689870
H	1.4686530	1.5669290	-2.9980640
H	-1.4686530	-1.5669290	-2.9980640
H	-1.4591720	-1.5765990	-5.4689870
H	0.0000000	0.0000000	-6.7177430
H	0.0123270	-2.1304290	-1.2492570
H	-0.0123270	2.1304290	-1.2492570
H	1.6012810	2.3043750	2.7186180
H	1.5945500	4.5582730	3.7333780
H	-0.0053480	6.2853920	2.9173100
H	-1.6123740	5.6999590	1.0820390
H	1.6123740	-5.6999590	1.0820390
H	0.0053480	-6.2853920	2.9173100
H	-1.5945500	-4.5582730	3.7333780
H	-1.6012810	-2.3043750	2.7186180

3_c (M06-2X/cc-pVTZ)

P	0.0000000	0.0000000	1.8376060
C	0.0047240	1.3454930	0.7300900
C	0.0007860	1.2150550	-0.6504870
C	0.0000000	0.0000000	-1.3321880
C	-0.0007860	-1.2150550	-0.6504870
C	-0.0047240	-1.3454930	0.7300900
C	-0.0127290	-2.7017610	1.3317160
C	-0.8848200	-3.0390160	2.3682530
C	-0.8844430	-4.3029720	2.9357750
C	0.0000000	-5.2686110	2.4773340

C	0.8840420	-4.9637300	1.4540720
C	0.8613710	-3.6979520	0.9054990
C	0.0127290	2.7017610	1.3317160
C	0.8848200	3.0390160	2.3682530
C	0.8844430	4.3029720	2.9357750
C	0.0000000	5.2686110	2.4773340
C	-0.8840420	4.9637300	1.4540720
C	-0.8613710	3.6979520	0.9054990
F	-1.7458270	3.4207190	-0.0666260
C	0.0000000	0.0000000	-2.8146580
C	0.7931980	0.8992790	-3.5266510
C	0.7954310	0.8975880	-4.9124870
C	0.0000000	0.0000000	-5.6103660
C	-0.7954310	-0.8975880	-4.9124870
C	-0.7931980	-0.8992790	-3.5266510
F	1.7458270	-3.4207190	-0.0666260
H	1.4249450	1.5945790	-5.4488830
H	1.4320400	1.5871670	-2.9881170
H	-1.4320400	-1.5871670	-2.9881170
H	-1.4249450	-1.5945790	-5.4488830
H	0.0000000	0.0000000	-6.6916620
H	0.0124930	-2.1205310	-1.2460040
H	-0.0124930	2.1205310	-1.2460040
H	1.5822870	2.2891320	2.7182290
H	1.5777650	4.5337400	3.7320900
H	-0.0054580	6.2577310	2.9135350
H	-1.5962810	5.6831800	1.0755340
H	1.5962810	-5.6831800	1.0755340
H	0.0054580	-6.2577310	2.9135350
H	-1.5777650	-4.5337400	3.7320900
H	-1.5822870	-2.2891320	2.7182290

3_c (wB97xd/6-31+G*)

P	0.0000000	0.0000000	1.8261310
C	0.0068490	1.3483850	0.7179540
C	0.0000000	1.2195450	-0.6683090
C	0.0000000	0.0000000	-1.3524330
C	0.0000000	-1.2195450	-0.6683090
C	-0.0068490	-1.3483850	0.7179540
C	-0.0257760	-2.7105890	1.3187480
C	-0.9940430	-3.0789790	2.2624590
C	-1.0107020	-4.3480180	2.8331200
C	-0.0473560	-5.2865460	2.4702720
C	0.9324730	-4.9508610	1.5402040
C	0.9237830	-3.6801350	0.9884160
C	0.0257760	2.7105890	1.3187480
C	0.9940430	3.0789790	2.2624590
C	1.0107020	4.3480180	2.8331200
C	0.0473560	5.2865460	2.4702720
C	-0.9324730	4.9508610	1.5402040
C	-0.9237830	3.6801350	0.9884160
F	-1.8984240	3.3725250	0.1044380
C	0.0000000	0.0000000	-2.8397310
C	0.8448200	0.8573330	-3.5548190
C	0.8455570	0.8571000	-4.9466950
C	0.0000000	0.0000000	-5.6480850
C	-0.8455570	-0.8571000	-4.9466950
C	-0.8448200	-0.8573330	-3.5548190
F	1.8984240	-3.3725250	0.1044380
H	1.5141210	1.5230450	-5.4848340
H	1.5237520	1.5132690	-3.0160490
H	-1.5237520	-1.5132690	-3.0160490

H	-1.5141210	-1.5230450	-5.4848340
H	0.0000000	0.0000000	-6.7343020
H	0.0153880	-2.1300420	-1.2637670
H	-0.0153880	2.1300420	-1.2637670
H	1.7519320	2.3511660	2.5385580
H	1.7775750	4.6033100	3.5576680
H	0.0533050	6.2789110	2.9107750
H	-1.7035350	5.6531790	1.2415300
H	1.7035350	-5.6531790	1.2415300
H	-0.0533050	-6.2789110	2.9107750
H	-1.7775750	-4.6033100	3.5576680
H	-1.7519320	-2.3511660	2.5385580

3_c (wB97xd/cc-pVTZ)

P	0.0000000	0.0000000	1.8185650
C	0.0072720	1.3453590	0.7151770
C	0.0000000	1.2137930	-0.6638180
C	0.0000000	0.0000000	-1.3443850
C	0.0000000	-1.2137930	-0.6638180
C	-0.0072720	-1.3453590	0.7151770
C	-0.0264650	-2.7023050	1.3171530
C	-0.9697910	-3.0544730	2.2818890
C	-0.9876950	-4.3148030	2.8543340
C	-0.0500490	-5.2606520	2.4722330
C	0.9044960	-4.9414520	1.5209970
C	0.8998130	-3.6790400	0.9644540
C	0.0264650	2.7023050	1.3171530
C	0.9697910	3.0544730	2.2818890
C	0.9876950	4.3148030	2.8543340
C	0.0500490	5.2606520	2.4722330
C	-0.9044960	4.9414520	1.5209970

C	-0.8998130	3.6790400	0.9644540
F	-1.8470840	3.3888420	0.0603170
C	0.0000000	0.0000000	-2.8274620
C	0.8234550	0.8693520	-3.5400270
C	0.8249210	0.8683070	-4.9247800
C	0.0000000	0.0000000	-5.6228070
C	-0.8249210	-0.8683070	-4.9247800
C	-0.8234550	-0.8693520	-3.5400270
F	1.8470840	-3.3888420	0.0603170
H	1.4779740	1.5433040	-5.4611300
H	1.4851710	1.5363710	-3.0032570
H	-1.4851710	-1.5363710	-3.0032570
H	-1.4779740	-1.5433040	-5.4611300
H	0.0000000	0.0000000	-6.7042220
H	0.0153560	-2.1194820	-1.2582340
H	-0.0153560	2.1194820	-1.2582340
H	1.7071590	2.3188880	2.5742360
H	1.7358230	4.5579400	3.5953040
H	0.0568060	6.2469520	2.9148010
H	-1.6557050	5.6515490	1.2065910
H	1.6557050	-5.6515490	1.2065910
H	-0.0568060	-6.2469520	2.9148010
H	-1.7358230	-4.5579400	3.5953040
H	-1.7071590	-2.3188880	2.5742360

3_d (B3LYP/6-31+G*)

P	0.2343350	-1.7357540	0.1241150
C	1.4586750	-0.4697170	0.0973980
C	1.1652830	0.8919580	0.0023240
C	-0.1264000	1.4361870	-0.1080580
C	-1.2611880	0.6109240	-0.1272760

C	-1.2375060	-0.7842140	-0.0398950
C	-2.5181060	-1.5423890	-0.0969530
C	-2.6774220	-2.6546890	-0.9461890
C	-3.8697770	-3.3775620	-0.9961930
C	-4.9473500	-3.0070630	-0.1873730
C	-4.8238790	-1.9129920	0.6716930
C	-3.6256540	-1.2119210	0.6975830
C	2.8859440	-0.8669900	0.2695460
C	3.6780800	-0.2909610	1.2813650
C	5.0175640	-0.6403350	1.4551980
C	5.6079970	-1.5851700	0.6108620
C	4.8532880	-2.1732800	-0.4056270
C	3.5207940	-1.8076390	-0.5520440
F	2.8214800	-2.3805290	-1.5688310
C	-0.2931680	2.9112890	-0.2152160
C	0.5672570	3.6818240	-1.0179200
C	0.4078620	5.0651740	-1.1164620
C	-0.6139630	5.7081090	-0.4120230
C	-1.4756990	4.9549610	0.3904570
C	-1.3178260	3.5713880	0.4870510
F	-3.5327910	-0.1641510	1.5650590
H	1.0776580	5.6394620	-1.7516790
H	1.3493950	3.1909300	-1.5912770
H	-1.9793430	2.9995250	1.1325300
H	-2.2685190	5.4453630	0.9497630
H	-0.7377260	6.7852990	-0.4881000
H	-2.2266290	1.0944560	-0.2546100
H	2.0015410	1.5888800	0.0218210
H	3.2162450	0.4299590	1.9506870
H	5.5954710	-0.1812640	2.2524530
H	6.6496950	-1.8656640	0.7399190

H	5.2785360	-2.9033310	-1.0872880
H	-5.6337310	-1.6001360	1.3234820
H	-5.8800440	-3.5632830	-0.2214100
H	-3.9570580	-4.2251030	-1.6701500
H	-1.8475310	-2.9380230	-1.5874080

3_d (B3LYP/cc-pVTZ)

P	0.2365570	-1.7227470	0.1179670
C	1.4565250	-0.4608530	0.0922360
C	1.1588290	0.8921340	-0.0031070
C	-0.1275570	1.4315330	-0.1104960
C	-1.2556830	0.6090410	-0.1268540
C	-1.2324890	-0.7786030	-0.0418090
C	-2.5064570	-1.5388120	-0.0951800
C	-2.6592720	-2.6502230	-0.9344340
C	-3.8421910	-3.3742360	-0.9818430
C	-4.9155390	-3.0060230	-0.1800890
C	-4.7979390	-1.9129260	0.6687490
C	-3.6098890	-1.2070240	0.6942640
C	2.8793480	-0.8560830	0.2634230
C	3.6765820	-0.2593180	1.2491890
C	5.0088030	-0.6061490	1.4215980
C	5.5873240	-1.5682200	0.6018520
C	4.8275580	-2.1769920	-0.3878050
C	3.5003020	-1.8165060	-0.5357390
F	2.7966070	-2.4110070	-1.5231630
C	-0.2966550	2.9018160	-0.2158460
C	0.5680260	3.6743420	-0.9995190
C	0.4058690	5.0497960	-1.0964780
C	-0.6221010	5.6841040	-0.4086340
C	-1.4879360	4.9300650	0.3750360

C	-1.3284960	3.5542200	0.4689610
F	-3.5221700	-0.1615940	1.5491860
H	1.0793460	5.6256560	-1.7173330
H	1.3569660	3.1906510	-1.5596140
H	-1.9954310	2.9811610	1.0988610
H	-2.2862750	5.4145310	0.9214260
H	-0.7475570	6.7559140	-0.4830490
H	-2.2173970	1.0904790	-0.2486150
H	1.9890980	1.5885960	0.0147060
H	3.2246950	0.4769310	1.8999940
H	5.5913200	-0.1309750	2.1987310
H	6.6243400	-1.8464180	0.7300860
H	5.2443540	-2.9221060	-1.0503870
H	-5.6064280	-1.6019630	1.3150300
H	-5.8413310	-3.5638100	-0.2119610
H	-3.9255760	-4.2216980	-1.6479840
H	-1.8302000	-2.9328470	-1.5684570

3_d (M06-2X/6-31+G*)

P	0.2618360	-1.7264630	0.1280520
C	1.4579440	-0.4497550	0.0984450
C	1.1531580	0.9053820	0.0023000
C	-0.1409610	1.4293400	-0.1073700
C	-1.2601710	0.5922450	-0.1263740
C	-1.2094760	-0.7979550	-0.0341630
C	-2.4796750	-1.5688230	-0.0912880
C	-2.6210140	-2.6929140	-0.9169410
C	-3.8076840	-3.4189020	-0.9644170
C	-4.8909890	-3.0357210	-0.1756480
C	-4.7818830	-1.9275540	0.6609040
C	-3.5893290	-1.2230370	0.6847590

C	2.8884940	-0.8262930	0.2643870
C	3.6963420	-0.1891250	1.2170600
C	5.0388670	-0.5199280	1.3740350
C	5.6109610	-1.5063890	0.5718510
C	4.8364970	-2.1571770	-0.3845930
C	3.5013160	-1.8088720	-0.5152550
F	2.7777530	-2.4389730	-1.4630040
C	-0.3289710	2.8991880	-0.2155290
C	0.5269770	3.6765320	-1.0063980
C	0.3468410	5.0535700	-1.1058270
C	-0.6913690	5.6761480	-0.4144170
C	-1.5487450	4.9122300	0.3762340
C	-1.3695290	3.5350200	0.4745410
F	-3.5020700	-0.1648830	1.5203000
H	1.0127930	5.6403840	-1.7319640
H	1.3225840	3.1938280	-1.5683650
H	-2.0262480	2.9467140	1.1108960
H	-2.3552760	5.3908950	0.9241470
H	-0.8319880	6.7502940	-0.4921460
H	-2.2346000	1.0588380	-0.2581130
H	1.9826120	1.6121920	0.0187880
H	3.2444900	0.5658340	1.8553610
H	5.6344470	-0.0130940	2.1267960
H	6.6571880	-1.7718240	0.6885580
H	5.2452420	-2.9234330	-1.0350780
H	-5.5981440	-1.6033990	1.2981570
H	-5.8203480	-3.5959400	-0.2077200
H	-3.8858610	-4.2795700	-1.6210830
H	-1.7811830	-2.9833200	-1.5424500

3_d (M06-2X/cc-pVTZ)

P	0.2759050	-1.7182300	0.1093330
C	1.4603890	-0.4374040	0.0815730
C	1.1443350	0.9088410	-0.0128280
C	-0.1479570	1.4210660	-0.1218600
C	-1.2558300	0.5791450	-0.1406580
C	-1.1985520	-0.8042070	-0.0477760
C	-2.4608170	-1.5813780	-0.0917520
C	-2.5951570	-2.7166890	-0.8924940
C	-3.7724050	-3.4465630	-0.9267790
C	-4.8528080	-3.0563430	-0.1492400
C	-4.7503570	-1.9371260	0.6625840
C	-3.5677780	-1.2268050	0.6749240
C	2.8893240	-0.8026450	0.2534110
C	3.6885770	-0.1440730	1.1901600
C	5.0258250	-0.4631300	1.3574910
C	5.6019570	-1.4589040	0.5815460
C	4.8365310	-2.1302250	-0.3586130
C	3.5049240	-1.7951020	-0.5016880
F	2.7916530	-2.4452570	-1.4343900
C	-0.3493180	2.8856890	-0.2211540
C	0.5141440	3.6800020	-0.9753740
C	0.3194090	5.0493780	-1.0646120
C	-0.7403900	5.6483060	-0.3983780
C	-1.6051200	4.8681590	0.3563490
C	-1.4121760	3.4986720	0.4433750
F	-3.4868770	-0.1577170	1.4870240
H	0.9915320	5.6491290	-1.6633710
H	1.3279030	3.2158370	-1.5174680
H	-2.0760630	2.8972080	1.0515260
H	-2.4288520	5.3287500	0.8847440
H	-0.8921310	6.7167610	-0.4678240

H	-2.2291260	1.0382220	-0.2687280
H	1.9644640	1.6193680	0.0074260
H	3.2331520	0.6193130	1.8075370
H	5.6147340	0.0603180	2.0972840
H	6.6446360	-1.7155190	0.7063940
H	5.2491390	-2.9049060	-0.9890990
H	-5.5649590	-1.6067170	1.2912650
H	-5.7751460	-3.6197240	-0.1709820
H	-3.8454860	-4.3165270	-1.5638330
H	-1.7558520	-3.0132880	-1.5078070

3_d (wB97xd/6-31+G*)

P	0.2381700	-1.7292890	0.1268980
C	1.4472760	-0.4697210	0.1128390
C	1.1597770	0.8883870	0.0229050
C	-0.1276570	1.4249280	-0.0985580
C	-1.2544830	0.6002550	-0.1333700
C	-1.2211980	-0.7901760	-0.0439470
C	-2.5026840	-1.5447000	-0.1096370
C	-2.6915390	-2.5881040	-1.0256890
C	-3.8877780	-3.2967090	-1.0866740
C	-4.9309020	-2.9774420	-0.2205230
C	-4.7732820	-1.9506620	0.7065040
C	-3.5727930	-1.2605660	0.7418760
C	2.8732850	-0.8683070	0.2829440
C	3.6283010	-0.3855350	1.3600400
C	4.9631060	-0.7385680	1.5309330
C	5.5778860	-1.5922790	0.6167510
C	4.8565160	-2.0852780	-0.4662360
C	3.5277560	-1.7172610	-0.6099560
F	2.8567500	-2.1902980	-1.6817820

C	-0.3018370	2.8978760	-0.2021480
C	0.5364070	3.6650170	-1.0202860
C	0.3644530	5.0427090	-1.1210730
C	-0.6486780	5.6768870	-0.4047060
C	-1.4871080	4.9234760	0.4146340
C	-1.3150800	3.5459510	0.5152180
F	-3.4393130	-0.2782890	1.6621910
H	1.0175660	5.6210940	-1.7684120
H	1.3128360	3.1758020	-1.6029510
H	-1.9599200	2.9679340	1.1722400
H	-2.2751350	5.4099260	0.9825950
H	-0.7849240	6.7513790	-0.4861650
H	-2.2228250	1.0774740	-0.2683180
H	1.9962620	1.5853310	0.0559250
H	3.1418120	0.2685580	2.0787750
H	5.5189940	-0.3527420	2.3798000
H	6.6186090	-1.8747300	0.7434590
H	5.3053080	-2.7450690	-1.2013010
H	-5.5601010	-1.6811110	1.4032050
H	-5.8682710	-3.5238310	-0.2624910
H	-4.0046760	-4.0937210	-1.8143590
H	-1.8834860	-2.8307360	-1.7102150

3_d (wB97xd/cc-pVTZ)

P	0.2453090	-1.7194060	0.1150230
C	1.4473490	-0.4600500	0.1050600
C	1.1523530	0.8894510	0.0182540
C	-0.1311330	1.4193930	-0.0977790
C	-1.2497710	0.5947040	-0.1283670
C	-1.2137640	-0.7888570	-0.0451060
C	-2.4887580	-1.5464100	-0.1062980

C	-2.6763290	-2.5828820	-1.0196080
C	-3.8650840	-3.2904020	-1.0790170
C	-4.9007090	-2.9773810	-0.2134660
C	-4.7434680	-1.9580040	0.7112810
C	-3.5512810	-1.2650070	0.7469340
C	2.8708030	-0.8521360	0.2748240
C	3.6295200	-0.3372150	1.3255170
C	4.9588990	-0.6808810	1.4978930
C	5.5653610	-1.5570260	0.6109700
C	4.8408530	-2.0822130	-0.4451010
C	3.5152860	-1.7255300	-0.5934960
F	2.8431890	-2.2306950	-1.6369190
C	-0.3114390	2.8873020	-0.2004750
C	0.5342720	3.6592280	-0.9947120
C	0.3530480	5.0281040	-1.0996240
C	-0.6764810	5.6503710	-0.4105330
C	-1.5213200	4.8934100	0.3864840
C	-1.3408790	3.5245870	0.4903500
F	-3.4177690	-0.2910950	1.6612870
H	1.0119040	5.6100040	-1.7297570
H	1.3259450	3.1795530	-1.5552820
H	-1.9932030	2.9431510	1.1286330
H	-2.3231780	5.3709260	0.9331580
H	-0.8204200	6.7186490	-0.4963400
H	-2.2163090	1.0677640	-0.2540430
H	1.9814730	1.5881130	0.0494600
H	3.1499020	0.3361590	2.0236050
H	5.5172650	-0.2697740	2.3269280
H	6.6030920	-1.8318020	0.7388980
H	5.2836570	-2.7612510	-1.1594670
H	-5.5255640	-1.6931980	1.4082180

H	-5.8330830	-3.5230230	-0.2546810
H	-3.9820620	-4.0819210	-1.8055460
H	-1.8718020	-2.8214940	-1.7023460

3_e (B3LYP/6-31+G*)

P	0.2193910	-1.7423830	-0.1519020
C	-1.2417740	-0.7623250	-0.2053430
C	-1.2473940	0.6351570	-0.1710350
C	-0.1017090	1.4426150	-0.1019400
C	1.1844890	0.8751860	-0.0651790
C	1.4596490	-0.4934920	-0.0865290
C	2.8888840	-0.9204440	-0.0803280
C	3.7917490	-0.4234230	-1.0395760
C	5.1347980	-0.8012060	-1.0516150
C	5.6165840	-1.6959370	-0.0917030
C	4.7501680	-2.2050580	0.8771660
C	3.4170930	-1.8127830	0.8617940
C	-2.5344780	-1.4956610	-0.3067670
C	-2.7254400	-2.5263080	-1.2473270
C	-3.9288860	-3.2265950	-1.3379730
C	-4.9861720	-2.9150420	-0.4791390
C	-4.8313690	-1.9024600	0.4700390
C	-3.6228960	-1.2217180	0.5345000
F	-3.4997440	-0.2550870	1.4876080
C	-0.2530000	2.9227250	-0.0628380
C	-1.2700800	3.5209350	0.7032920
C	-1.4146420	4.9086260	0.7436240
C	-0.5466120	5.7291000	0.0176960
C	0.4683630	5.1487790	-0.7482940
C	0.6137120	3.7610710	-0.7871040
F	2.6066230	-2.3070710	1.8350120

H	-2.2016180	5.3489440	1.3507630
H	-1.9353960	2.8952650	1.2923990
H	1.3917120	3.3224150	-1.4065950
H	1.1438480	5.7764720	-1.3242280
H	-0.6595530	6.8096880	0.0487890
H	2.0295990	1.5555250	0.0248580
H	-2.2086400	1.1389640	-0.2349170
H	-1.9111930	-2.7634660	-1.9261960
H	-4.0406010	-4.0102870	-2.0819760
H	-5.9271910	-3.4542720	-0.5436300
H	-5.6247700	-1.6379400	1.1622150
H	5.0888210	-2.8933300	1.6453160
H	6.6602390	-1.9980080	-0.0937720
H	5.8003600	-0.4032700	-1.8125800
H	3.4153820	0.2580220	-1.7980360

3_e (B3LYP/cc-pVTZ)

P	0.2251960	-1.7286260	-0.1505870
C	-1.2349150	-0.7587700	-0.2051580
C	-1.2420410	0.6310370	-0.1701160
C	-0.1046050	1.4382100	-0.1026430
C	1.1772300	0.8780250	-0.0667180
C	1.4586010	-0.4816800	-0.0855790
C	2.8837270	-0.9053890	-0.0777300
C	3.7887900	-0.3922400	-1.0157940
C	5.1245040	-0.7675930	-1.0254900
C	5.5972480	-1.6743640	-0.0839880
C	4.7287370	-2.1990390	0.8634090
C	3.4006300	-1.8121340	0.8481730
C	-2.5193220	-1.4969450	-0.3042840
C	-2.6976190	-2.5345540	-1.2286810

C	-3.8897200	-3.2392760	-1.3173940
C	-4.9473960	-2.9257970	-0.4726550
C	-4.8048110	-1.9064330	0.4598810
C	-3.6079980	-1.2180280	0.5252250
F	-3.4966350	-0.2460980	1.4600760
C	-0.2613180	2.9130360	-0.0624260
C	-1.2901410	3.5031070	0.6813860
C	-1.4393360	4.8825120	0.7236940
C	-0.5653220	5.7041630	0.0214150
C	0.4605720	5.1331270	-0.7226560
C	0.6116020	3.7537250	-0.7625630
F	2.5885260	-2.3232440	1.7971940
H	-2.2357350	5.3162110	1.3137860
H	-1.9627340	2.8763630	1.2512100
H	1.4003020	3.3229990	-1.3646100
H	1.1412510	5.7625580	-1.2804810
H	-0.6822940	6.7790000	0.0537060
H	2.0155410	1.5593040	0.0204350
H	-2.2000770	1.1308040	-0.2302840
H	-1.8807370	-2.7746730	-1.8951690
H	-3.9925200	-4.0288890	-2.0487380
H	-5.8801290	-3.4691530	-0.5358160
H	-5.6004260	-1.6400080	1.1410840
H	5.0610180	-2.8981150	1.6176100
H	6.6361940	-1.9740590	-0.0844100
H	5.7924680	-0.3574670	-1.7702940
H	3.4203630	0.3004060	-1.7604900

3_e (M06-2X/6-31+G*)

P	0.2576290	-1.7325730	-0.1470520
C	-1.2080720	-0.7839160	-0.2082650

C	-1.2481110	0.6092090	-0.1795230
C	-0.1228510	1.4348700	-0.1044430
C	1.1689490	0.8952970	-0.0621980
C	1.4623750	-0.4657270	-0.0835380
C	2.8959300	-0.8657470	-0.0764230
C	3.8001860	-0.3140610	-0.9951440
C	5.1450310	-0.6719120	-1.0003910
C	5.6209650	-1.5996460	-0.0744290
C	4.7498350	-2.1627490	0.8540010
C	3.4148750	-1.7898910	0.8324120
C	-2.4872400	-1.5357850	-0.3079220
C	-2.6584970	-2.5853240	-1.2214300
C	-3.8540080	-3.2936040	-1.3029040
C	-4.9157560	-2.9679240	-0.4608700
C	-4.7769600	-1.9341240	0.4621480
C	-3.5763900	-1.2454570	0.5180660
F	-3.4588790	-0.2611540	1.4358030
C	-0.3051760	2.9087980	-0.0618930
C	-1.3404380	3.4737350	0.6944380
C	-1.5158610	4.8541190	0.7388050
C	-0.6595650	5.6934090	0.0278140
C	0.3738590	5.1422150	-0.7281140
C	0.5495650	3.7617390	-0.7716200
F	2.5959910	-2.3315210	1.7555950
H	-2.3178780	5.2753270	1.3382250
H	-1.9952540	2.8263560	1.2727790
H	1.3420390	3.3384570	-1.3839790
H	1.0396790	5.7875790	-1.2939620
H	-0.7967060	6.7701910	0.0624200
H	2.0031830	1.5902140	0.0318170
H	-2.2214630	1.0911590	-0.2507950

H	-1.8353660	-2.8310200	-1.8870690
H	-3.9571280	-4.0948690	-2.0276800
H	-5.8518210	-3.5147990	-0.5189680
H	-5.5759990	-1.6564460	1.1417230
H	5.0839180	-2.8796860	1.5967240
H	6.6680970	-1.8863660	-0.0722020
H	5.8185250	-0.2334960	-1.7300720
H	3.4218710	0.3949330	-1.7272260

3_e (M06-2X/cc-pVTZ)

P	0.2608360	-1.7216720	-0.1425510
C	-1.2031340	-0.7803170	-0.2051540
C	-1.2432030	0.6063740	-0.1767990
C	-0.1243170	1.4304810	-0.1021810
C	1.1629120	0.8960550	-0.0590960
C	1.4612930	-0.4576210	-0.0779680
C	2.8921830	-0.8529220	-0.0696880
C	3.8002550	-0.2669330	-0.9538550
C	5.1400730	-0.6178080	-0.9592110
C	5.6083110	-1.5725710	-0.0673190
C	4.7337020	-2.1701150	0.8257490
C	3.4022390	-1.8055040	0.8061800
C	-2.4758810	-1.5358110	-0.3023250
C	-2.6312880	-2.6013420	-1.1908190
C	-3.8169050	-3.3134160	-1.2720290
C	-4.8855090	-2.9756970	-0.4546000
C	-4.7626280	-1.9262930	0.4428720
C	-3.5718310	-1.2320810	0.5007790
F	-3.4733980	-0.2333460	1.3958370
C	-0.3098710	2.8999630	-0.0594490
C	-1.3565260	3.4587380	0.6746520

C	-1.5346020	4.8322650	0.7196710
C	-0.6708250	5.6714500	0.0302870
C	0.3733840	5.1270940	-0.7037950
C	0.5529730	3.7535000	-0.7464470
F	2.5813080	-2.3850530	1.6947270
H	-2.3454840	5.2487670	1.3017460
H	-2.0189710	2.8103770	1.2341910
H	1.3554260	3.3350210	-1.3402380
H	1.0454540	5.7731580	-1.2523060
H	-0.8107690	6.7431700	0.0646200
H	1.9909480	1.5914390	0.0335630
H	-2.2123260	1.0861960	-0.2472630
H	-1.8010580	-2.8578830	-1.8356180
H	-3.9069300	-4.1277230	-1.9768510
H	-5.8146240	-3.5252920	-0.5121300
H	-5.5677240	-1.6380070	1.1036240
H	5.0611910	-2.9103690	1.5417760
H	6.6521200	-1.8541330	-0.0651930
H	5.8159340	-0.1527100	-1.6628860
H	3.4282520	0.4637160	-1.6605750

3_e (wB97xd/6-31+G*)

P	0.2063010	-1.7331010	-0.1767370
C	-1.2360260	-0.7526740	-0.2214290
C	-1.2399470	0.6403790	-0.1830810
C	-0.0938390	1.4347960	-0.1061240
C	1.1836100	0.8641960	-0.0642090
C	1.4433960	-0.5027590	-0.0950060
C	2.8689630	-0.9368150	-0.0855930
C	3.7630660	-0.4645190	-1.0561080
C	5.1008830	-0.8458830	-1.0642580

C	5.5794450	-1.7198700	-0.0898410
C	4.7184800	-2.2032970	0.8905110
C	3.3903290	-1.8062010	0.8734900
C	-2.5353230	-1.4727070	-0.3200660
C	-2.7674700	-2.4363940	-1.3106630
C	-3.9777170	-3.1180540	-1.3957750
C	-4.9928480	-2.8512430	-0.4799700
C	-4.7930190	-1.9032500	0.5197220
C	-3.5787860	-1.2392100	0.5789740
F	-3.4045350	-0.3376130	1.5717480
C	-0.2373220	2.9141490	-0.0623700
C	-1.2027670	3.5096450	0.7585340
C	-1.3418280	4.8939320	0.8019000
C	-0.5191710	5.7057840	0.0235390
C	0.4448460	5.1238700	-0.7972600
C	0.5849050	3.7394360	-0.8388600
F	2.5843310	-2.2699540	1.8508840
H	-2.0899290	5.3387840	1.4519180
H	-1.8332860	2.8838580	1.3849650
H	1.3241230	3.2931320	-1.4993730
H	1.0848390	5.7486210	-1.4139650
H	-0.6290140	6.7859230	0.0569850
H	2.0338920	1.5384180	0.0298090
H	-2.1999160	1.1482000	-0.2466900
H	-1.9813580	-2.6383150	-2.0329040
H	-4.1267370	-3.8552950	-2.1785540
H	-5.9407520	-3.3772940	-0.5400820
H	-5.5570970	-1.6764830	1.2558230
H	5.0584450	-2.8776860	1.6693730
H	6.6213790	-2.0254070	-0.0888210
H	5.7667960	-0.4667870	-1.8333340

H	3.3847710	0.2050870	-1.8241170

3_e (wB97xd/cc-pVTZ)

P	0.2113080	-1.7209270	-0.1779310
C	-1.2300530	-0.7492500	-0.2193490
C	-1.2351170	0.6366360	-0.1762760
C	-0.0964290	1.4302720	-0.1032510
C	1.1767640	0.8661990	-0.0646970
C	1.4427170	-0.4923280	-0.0940540
C	2.8651470	-0.9225840	-0.0822010
C	3.7644340	-0.4242500	-1.0249300
C	5.0960170	-0.8009020	-1.0302540
C	5.5640330	-1.6955420	-0.0801970
C	4.6983020	-2.2045380	0.8723240
C	3.3739330	-1.8144590	0.8553750
C	-2.5221760	-1.4732930	-0.3172310
C	-2.7427120	-2.4432110	-1.2944350
C	-3.9432140	-3.1275020	-1.3800260
C	-4.9594520	-2.8577970	-0.4775690
C	-4.7707180	-1.9037300	0.5085780
C	-3.5664560	-1.2337810	0.5709630
F	-3.4033590	-0.3275290	1.5476880
C	-0.2442440	2.9048310	-0.0588260
C	-1.2180940	3.4941700	0.7450900
C	-1.3613710	4.8708140	0.7882050
C	-0.5359790	5.6819520	0.0249120
C	0.4357410	5.1069170	-0.7793020
C	0.5814760	3.7301780	-0.8192170
F	2.5655980	-2.3045500	1.8038430
H	-2.1160640	5.3107370	1.4259160
H	-1.8532800	2.8677820	1.3576600

H	1.3288410	3.2892240	-1.4659820
H	1.0779710	5.7319060	-1.3848750
H	-0.6499310	6.7568550	0.0576440
H	2.0204090	1.5420820	0.0244060
H	-2.1921360	1.1411010	-0.2324050
H	-1.9536520	-2.6487930	-2.0053620
H	-4.0837130	-3.8698380	-2.1528570
H	-5.9007680	-3.3858380	-0.5386630
H	-5.5371080	-1.6737160	1.2346850
H	5.0304110	-2.8967520	1.6325540
H	6.6022090	-1.9970710	-0.0769400
H	5.7663270	-0.4018250	-1.7783610
H	3.3954040	0.2639560	-1.7740220

3_f (B3LYP/6-31+G*)

P	-0.2091480	-1.7374970	0.1319260
C	1.2514790	-0.7643410	0.0014720
C	1.2569420	0.6312990	-0.0810100
C	0.1099380	1.4398360	-0.0948720
C	-1.1762460	0.8761570	-0.0157730
C	-1.4503370	-0.4894960	0.0781330
C	-2.8763820	-0.9123380	0.1899990
C	-3.7101950	-0.3804970	1.1919920
C	-5.0489330	-0.7553060	1.3102720
C	-5.5958510	-1.6815580	0.4174030
C	-4.7988800	-2.2258210	-0.5911700
C	-3.4682680	-1.8357910	-0.6814760
C	2.5444520	-1.5044240	-0.0095330
C	2.7517670	-2.6040260	-0.8647020
C	3.9547770	-3.3109490	-0.8757290
C	4.9941010	-2.9364460	-0.0200370

C	4.8217330	-1.8550200	0.8465590
C	3.6140850	-1.1694670	0.8334690
F	3.4713320	-0.1359640	1.7092340
C	0.2554270	2.9168110	-0.2107690
C	1.2009980	3.4836180	-1.0843010
C	1.3369900	4.8684610	-1.1937780
C	0.5310590	5.7178010	-0.4305560
C	-0.4125330	5.1691710	0.4424510
C	-0.5489480	3.7842570	0.5502210
F	-2.7262520	-2.3657330	-1.6909060
H	2.0680000	5.2837350	-1.8829850
H	1.8163590	2.8350570	-1.7021940
H	-1.2685380	3.3707390	1.2519680
H	-1.0381650	5.8197950	1.0484190
H	0.6372200	6.7961990	-0.5151350
H	-2.0249620	1.5557310	-0.0716040
H	2.2208250	1.1318810	-0.1306870
H	1.9512170	-2.8907160	-1.5408670
H	4.0798700	-4.1493530	-1.5551420
H	5.9346380	-3.4803920	-0.0227860
H	5.6005250	-1.5404460	1.5343890
H	-5.1899470	-2.9399360	-1.3092000
H	-6.6366840	-1.9813410	0.5026290
H	-5.6603080	-0.3304970	2.1015500
H	-3.2816170	0.3256800	1.8982270

3_f (B3LYP/cc-pVTZ)

P	-0.2146940	-1.7231830	0.1316320
C	1.2449450	-0.7596520	0.0063310
C	1.2515380	0.6285700	-0.0732990
C	0.1124060	1.4361130	-0.0907660

C	-1.1693210	0.8790780	-0.0150950
C	-1.4495520	-0.4776430	0.0773190
C	-2.8715320	-0.8976340	0.1863330
C	-3.7112870	-0.3460250	1.1624200
C	-5.0428370	-0.7188280	1.2772190
C	-5.5769540	-1.6617360	0.4065670
C	-4.7741100	-2.2253180	-0.5757130
C	-3.4485790	-1.8400770	-0.6653720
C	2.5295290	-1.5045360	-0.0050260
C	2.7213000	-2.6091230	-0.8453230
C	3.9127270	-3.3205490	-0.8585850
C	4.9553290	-2.9459910	-0.0199950
C	4.7982770	-1.8596120	0.8309590
C	3.6024860	-1.1659670	0.8224820
F	3.4752410	-0.1276180	1.6789960
C	0.2625790	2.9078530	-0.2078830
C	1.2268940	3.4675830	-1.0540320
C	1.3663510	4.8441160	-1.1664060
C	0.5463200	5.6936440	-0.4331090
C	-0.4153730	5.1532870	0.4126270
C	-0.5564590	3.7766880	0.5224690
F	-2.7013850	-2.3902870	-1.6461120
H	2.1121100	5.2534870	-1.8348530
H	1.8553380	2.8190750	-1.6491860
H	-1.2927470	3.3703910	1.2026500
H	-1.0528890	5.8049400	0.9953360
H	0.6554860	6.7662770	-0.5198180
H	-2.0111040	1.5593960	-0.0707580
H	2.2117220	1.1257790	-0.1194070
H	1.9159860	-2.8968910	-1.5070960
H	4.0265460	-4.1631690	-1.5263600

H	5.8874910	-3.4939980	-0.0245360
H	5.5818100	-1.5443140	1.5052940
H	-5.1559580	-2.9539320	-1.2766060
H	-6.6130850	-1.9597180	0.4894360
H	-5.6595330	-0.2787780	2.0486540
H	-3.2935280	0.3748920	1.8520910

3_f (M06-2X/6-31+G*)

P	-0.2297070	-1.7257630	0.1397780
C	1.2273750	-0.7700410	0.0195490
C	1.2555070	0.6218430	-0.0575660
C	0.1204430	1.4380170	-0.0775950
C	-1.1668720	0.8894630	-0.0061120
C	-1.4470340	-0.4709550	0.0862900
C	-2.8740250	-0.8818020	0.1877520
C	-3.7264600	-0.3061120	1.1402420
C	-5.0642790	-0.6764300	1.2402470
C	-5.5851990	-1.6406670	0.3779480
C	-4.7659730	-2.2284300	-0.5817460
C	-3.4368870	-1.8422300	-0.6546440
C	2.5117020	-1.5203030	-0.0009450
C	2.7032350	-2.6176960	-0.8519450
C	3.9022740	-3.3244720	-0.8719840
C	4.9466110	-2.9485260	-0.0291820
C	4.7862030	-1.8674860	0.8341740
C	3.5822590	-1.1815990	0.8303190
F	3.4420880	-0.1521470	1.6919010
C	0.2798790	2.9102670	-0.2021770
C	1.2380970	3.4536940	-1.0677090
C	1.3857130	4.8325010	-1.1905720
C	0.5775170	5.6930860	-0.4495830

C	-0.3792890	5.1640310	0.4153620
C	-0.5264930	3.7849510	0.5373310
F	-2.6690590	-2.4096730	-1.6067450
H	2.1268340	5.2349810	-1.8750350
H	1.8538520	2.7905450	-1.6697320
H	-1.2568360	3.3806370	1.2336450
H	-1.0071970	5.8259210	1.0048460
H	0.6921650	6.7688060	-0.5457680
H	-2.0114940	1.5753800	-0.0687600
H	2.2280550	1.1090720	-0.1029900
H	1.8934460	-2.9023900	-1.5185310
H	4.0208590	-4.1639890	-1.5496540
H	5.8852340	-3.4939820	-0.0398020
H	5.5703290	-1.5499190	1.5137720
H	-5.1362170	-2.9742360	-1.2773030
H	-6.6271710	-1.9370290	0.4496880
H	-5.6968740	-0.2186220	1.9942840
H	-3.3124480	0.4310200	1.8235400

3_f (M06-2X/cc-pVTZ)

P	-0.2350100	-1.7159010	0.1324040
C	1.2216910	-0.7688380	0.0139560
C	1.2515180	0.6163340	-0.0671810
C	0.1236000	1.4318910	-0.0929090
C	-1.1596410	0.8893120	-0.0258590
C	-1.4471390	-0.4631300	0.0699760
C	-2.8725520	-0.8646940	0.1743110
C	-3.7316560	-0.2327950	1.0755830
C	-5.0657960	-0.5894940	1.1808170
C	-5.5779790	-1.5972130	0.3756240
C	-4.7526600	-2.2418130	-0.5315700

C	-3.4259000	-1.8693110	-0.6125330
C	2.4990490	-1.5236660	0.0021080
C	2.6770030	-2.6377990	-0.8198110
C	3.8664880	-3.3482350	-0.8333830
C	4.9150490	-2.9594600	-0.0127750
C	4.7683250	-1.8615700	0.8210190
C	3.5742600	-1.1700180	0.8122690
F	3.4492490	-0.1219470	1.6434630
C	0.2880000	2.9001610	-0.2112820
C	1.2714560	3.4428190	-1.0384210
C	1.4254840	4.8153670	-1.1506800
C	0.5994530	5.6714320	-0.4365760
C	-0.3824160	5.1439690	0.3900210
C	-0.5370400	3.7712140	0.5002790
F	-2.6544990	-2.4945600	-1.5155100
H	2.1869160	5.2171650	-1.8053250
H	1.9031270	2.7830990	-1.6188330
H	-1.2879760	3.3678380	1.1672300
H	-1.0244970	5.8028480	0.9588430
H	0.7201800	6.7425800	-0.5235350
H	-1.9970820	1.5763630	-0.0920070
H	2.2202990	1.1004510	-0.1100970
H	1.8622400	-2.9333100	-1.4677900
H	3.9743060	-4.2011980	-1.4882510
H	5.8467340	-3.5076790	-0.0182460
H	5.5568110	-1.5326840	1.4829310
H	-5.1148770	-3.0239850	-1.1833410
H	-6.6176160	-1.8836030	0.4518410
H	-5.7026110	-0.0866590	1.8948100
H	-3.3251230	0.5390640	1.7162310

3_f (wB97xd/6-31+G*)

P	-0.1938460	-1.7250580	0.1463560
C	1.2461650	-0.7507360	0.0146260
C	1.2495350	0.6404920	-0.0676490
C	0.1012530	1.4360720	-0.0811530
C	-1.1763170	0.8679480	-0.0043200
C	-1.4329160	-0.4960410	0.0916800
C	-2.8540030	-0.9325360	0.1950220
C	-3.6746350	-0.4640450	1.2296030
C	-5.0049330	-0.8562410	1.3398130
C	-5.5490130	-1.7359790	0.4057020
C	-4.7620680	-2.2146080	-0.6371550
C	-3.4397310	-1.8071920	-0.7208630
C	2.5445440	-1.4792310	-0.0139740
C	2.7869640	-2.4927860	-0.9505800
C	3.9966950	-3.1797930	-0.9843200
C	4.9991960	-2.8680780	-0.0688870
C	4.7874180	-1.8711060	0.8796260
C	3.5741560	-1.2021480	0.8876230
F	3.3851710	-0.2510720	1.8288080
C	0.2369290	2.9123890	-0.1997200
C	1.1354040	3.4751980	-1.1142150
C	1.2624310	4.8566730	-1.2283820
C	0.4924240	5.6992050	-0.4292550
C	-0.4051170	5.1505620	0.4845100
C	-0.5316000	3.7688150	0.5978850
F	-2.7050540	-2.2677740	-1.7553830
H	1.9594980	5.2745650	-1.9492250
H	1.7233770	2.8262670	-1.7579690
H	-1.2178790	3.3500540	1.3295200
H	-1.0042950	5.7992080	1.1172580

H	0.5918720	6.7770680	-0.5188830
H	-2.0316810	1.5402020	-0.0579750
H	2.2124310	1.1442780	-0.1210910
H	2.0095240	-2.7295140	-1.6716810
H	4.1553120	-3.9549260	-1.7275970
H	5.9466520	-3.3980940	-0.0884820
H	5.5417430	-1.6089030	1.6139580
H	-5.1548280	-2.8936900	-1.3865660
H	-6.5852050	-2.0503770	0.4859770
H	-5.6129730	-0.4812910	2.1573020
H	-3.2436080	0.2096230	1.9655880

3_f (wB97xd/cc-pVTZ)

P	-0.2044530	-1.7108370	0.1515410
C	1.2369720	-0.7485760	0.0244970
C	1.2446130	0.6356840	-0.0555060
C	0.1058950	1.4333780	-0.0692760
C	-1.1687770	0.8749490	0.0077350
C	-1.4347660	-0.4802220	0.0990980
C	-2.8535580	-0.9118990	0.1941540
C	-3.6855450	-0.4206510	1.1996630
C	-5.0099360	-0.8097590	1.3013790
C	-5.5375760	-1.7085050	0.3870070
C	-4.7398350	-2.2087330	-0.6274030
C	-3.4215510	-1.8064980	-0.7058660
C	2.5261140	-1.4845900	-0.0073080
C	2.7514490	-2.5018840	-0.9337820
C	3.9497350	-3.1943010	-0.9737400
C	4.9570900	-2.8848080	-0.0742900
C	4.7620280	-1.8843620	0.8637220
C	3.5605880	-1.2061090	0.8800670

F	3.3889390	-0.2516010	1.8064600
C	0.2504480	2.9040690	-0.1939910
C	1.1636690	3.4520420	-1.0925980
C	1.2989580	4.8244760	-1.2167100
C	0.5239700	5.6736060	-0.4424220
C	-0.3874160	5.1407720	0.4558690
C	-0.5238500	3.7678570	0.5778460
F	-2.6792840	-2.2888810	-1.7115750
H	2.0071280	5.2304840	-1.9262150
H	1.7577810	2.7974380	-1.7163800
H	-1.2239080	3.3613030	1.2959300
H	-0.9910660	5.7952910	1.0698110
H	0.6304970	6.7452900	-0.5397900
H	-2.0154700	1.5510990	-0.0422870
H	2.2054900	1.1334990	-0.1091800
H	1.9682250	-2.7378590	-1.6417500
H	4.0957420	-3.9718530	-1.7100400
H	5.8967000	-3.4189260	-0.0991560
H	5.5219050	-1.6229370	1.5860070
H	-5.1198700	-2.9037280	-1.3622400
H	-6.5699630	-2.0206340	0.4613030
H	-5.6270770	-0.4170770	2.0971020
H	-3.2685210	0.2699110	1.9209340

3_g (B3LYP/6-31+G*)

P	-0.1941920	-1.7411400	-0.1526830
C	-1.4467750	-0.5030030	-0.1179360
C	-1.1828280	0.8676270	-0.0970970
C	0.0988010	1.4460340	-0.0968000
C	1.2533870	0.6490790	-0.1281920
C	1.2590330	-0.7483390	-0.1686540

C	2.5596870	-1.4738510	-0.2205100
C	2.7982640	-2.4855460	-1.1705510
C	4.0081890	-3.1790210	-1.2189130
C	5.0231120	-2.8792930	-0.3063860
C	4.8196370	-1.8859080	0.6537600
C	3.6058580	-1.2113630	0.6758720
C	-2.8721060	-0.9400450	-0.1686940
C	-3.7455860	-0.4377050	-1.1522020
C	-5.0845960	-0.8250020	-1.2137670
C	-5.5919330	-1.7347610	-0.2814980
C	-4.7549960	-2.2493980	0.7101380
C	-3.4252670	-1.8473680	0.7444050
F	-2.6440830	-2.3474530	1.7387280
C	0.2305220	2.9284010	-0.0621300
C	-0.5916060	3.7058570	0.7735450
C	-0.4675200	5.0957300	0.8095940
C	0.4799200	5.7391970	0.0085200
C	1.3029570	4.9796170	-0.8277330
C	1.1801240	3.5895410	-0.8616890
F	3.4324140	-0.2654760	1.6400770
H	-1.1059290	5.6750660	1.4719330
H	-1.3139430	3.2160070	1.4213650
H	1.8104980	3.0126520	-1.5333590
H	2.0378910	5.4696620	-1.4614650
H	0.5765400	6.8214500	0.0361930
H	2.2130930	1.1597200	-0.1094490
H	-2.0362580	1.5435690	-0.0868130
H	-3.3494940	0.2563780	-1.8888020
H	-5.7270250	-0.4224100	-1.9919560
H	-6.6326380	-2.0441480	-0.3225960
H	-5.1140260	-2.9495220	1.4580690

H	5.5787210	-1.6317350	1.3870830
H	5.9686380	-3.4136390	-0.3372020
H	4.1578050	-3.9484450	-1.9711240
H	2.0163640	-2.7140150	-1.8893730

3_g (B3LYP/cc-pVTZ)

P	-0.2001140	-1.7269390	-0.1478030
C	-1.4459650	-0.4908850	-0.1161150
C	-1.1755370	0.8706900	-0.0971100
C	0.1019150	1.4415150	-0.0963970
C	1.2484210	0.6451550	-0.1254330
C	1.2526500	-0.7448480	-0.1652480
C	2.5441320	-1.4765560	-0.2167250
C	2.7624580	-2.5033820	-1.1444920
C	3.9593760	-3.2039530	-1.1929860
C	4.9809730	-2.8970140	-0.3026980
C	4.7976510	-1.8884960	0.6344440
C	3.5970070	-1.2036440	0.6594480
C	-2.8670900	-0.9251680	-0.1650190
C	-3.7419640	-0.4132060	-1.1319680
C	-5.0734350	-0.7991650	-1.1912100
C	-5.5722690	-1.7153400	-0.2724960
C	-4.7339720	-2.2389510	0.7023160
C	-3.4094440	-1.8414810	0.7365960
F	-2.6271780	-2.3519890	1.7109660
C	0.2384380	2.9188140	-0.0620630
C	-0.5885580	3.6976280	0.7555980
C	-0.4592480	5.0793320	0.7923500
C	0.4970770	5.7145520	0.0088730
C	1.3243300	4.9547390	-0.8099930
C	1.1977830	3.5726400	-0.8434600

F	3.4441790	-0.2428870	1.5980510
H	-1.1017400	5.6597110	1.4411770
H	-1.3199660	3.2144950	1.3893750
H	1.8333690	2.9955370	-1.5013870
H	2.0666400	5.4391660	-1.4304100
H	0.5971920	6.7912110	0.0365190
H	2.2043770	1.1522370	-0.1057110
H	-2.0216950	1.5479290	-0.0873360
H	-3.3532460	0.2876650	-1.8584210
H	-5.7176140	-0.3895890	-1.9569600
H	-6.6080890	-2.0231570	-0.3118810
H	-5.0871960	-2.9453690	1.4399940
H	5.5641770	-1.6277400	1.3503310
H	5.9170060	-3.4375100	-0.3336230
H	4.0938310	-3.9854730	-1.9278870
H	1.9732740	-2.7389870	-1.8451560

3_g (M06-2X/6-31+G*)

P	-0.2123110	-1.7281920	-0.1437630
C	-1.4412940	-0.4836180	-0.1146890
C	-1.1720040	0.8817680	-0.0978650
C	0.1106810	1.4449170	-0.0930470
C	1.2536370	0.6402200	-0.1190930
C	1.2368970	-0.7534860	-0.1608430
C	2.5277070	-1.4910270	-0.2187180
C	2.7460960	-2.5112080	-1.1553070
C	3.9477970	-3.2119230	-1.2038470
C	4.9680300	-2.9078220	-0.3045540
C	4.7815160	-1.9032400	0.6421650
C	3.5756830	-1.2210720	0.6646760
C	-2.8663380	-0.9108080	-0.1662630

C	-3.7429800	-0.3865510	-1.1265650
C	-5.0783510	-0.7746390	-1.1833690
C	-5.5718940	-1.7049410	-0.2691720
C	-4.7281250	-2.2402510	0.7000960
C	-3.4021040	-1.8376900	0.7298100
F	-2.6094560	-2.3514690	1.6913190
C	0.2548310	2.9237650	-0.0598540
C	-0.5681360	3.7015490	0.7653300
C	-0.4351440	5.0870230	0.7991140
C	0.5219080	5.7189070	0.0070370
C	1.3459710	4.9550750	-0.8183170
C	1.2136470	3.5693110	-0.8506000
F	3.4098570	-0.2667400	1.6042750
H	-1.0745050	5.6737040	1.4524780
H	-1.2988430	3.2140120	1.4057840
H	1.8437360	2.9817280	-1.5134320
H	2.0896170	5.4391140	-1.4449260
H	0.6259700	6.7996220	0.0332700
H	2.2218830	1.1376900	-0.0958810
H	-2.0219000	1.5640500	-0.0932790
H	-3.3509420	0.3251820	-1.8485900
H	-5.7306990	-0.3568780	-1.9437320
H	-6.6117160	-2.0148040	-0.3073100
H	-5.0770250	-2.9578870	1.4352500
H	5.5467110	-1.6440080	1.3666730
H	5.9082350	-3.4497740	-0.3357250
H	4.0873650	-3.9908290	-1.9466460
H	1.9549850	-2.7397160	-1.8645830

3_g (M06-2X/cc-pVTZ)

P	-0.2187030	-1.7186090	-0.1414790

C	-1.4416670	-0.4751000	-0.1084650
C	-1.1655870	0.8826300	-0.0899750
C	0.1133130	1.4389900	-0.0885270
C	1.2487610	0.6342330	-0.1163820
C	1.2301190	-0.7530740	-0.1586570
C	2.5138520	-1.4954290	-0.2148570
C	2.7151110	-2.5332700	-1.1266800
C	3.9073120	-3.2373380	-1.1751570
C	4.9354260	-2.9194530	-0.3000470
C	4.7658880	-1.8976980	0.6215720
C	3.5695440	-1.2103430	0.6459970
C	-2.8650460	-0.8947560	-0.1580940
C	-3.7469180	-0.3329650	-1.0834840
C	-5.0785960	-0.7099430	-1.1393050
C	-5.5646270	-1.6660960	-0.2584040
C	-4.7159550	-2.2393110	0.6748470
C	-3.3920230	-1.8492240	0.7054120
F	-2.5963600	-2.4048600	1.6317700
C	0.2635390	2.9132710	-0.0564940
C	-0.5666760	3.6958330	0.7464790
C	-0.4255540	5.0741380	0.7795130
C	0.5457420	5.6949510	0.0073070
C	1.3764030	4.9270850	-0.7966760
C	1.2372710	3.5484430	-0.8268020
F	3.4226040	-0.2391780	1.5619120
H	-1.0703480	5.6645130	1.4164190
H	-1.3097520	3.2164910	1.3705100
H	1.8737110	2.9571470	-1.4722460
H	2.1313340	5.4031860	-1.4075990
H	0.6558220	6.7704110	0.0324230
H	2.2133580	1.1283110	-0.0938390

H	-2.0086420	1.5661310	-0.0814450
H	-3.3609820	0.3995460	-1.7806560
H	-5.7343580	-0.2631270	-1.8731810
H	-6.6021790	-1.9675220	-0.2957020
H	-5.0582640	-2.9794360	1.3840600
H	5.5381980	-1.6260430	1.3271020
H	5.8689110	-3.4637480	-0.3310570
H	4.0337820	-4.0298370	-1.8990310
H	1.9159630	-2.7739900	-1.8154490

3_g (wB97xd/6-31+G*)

P	-0.1850260	-1.7274040	-0.1687370
C	-1.4314370	-0.5047800	-0.1255480
C	-1.1805470	0.8634490	-0.0980770
C	0.0940960	1.4423650	-0.0918000
C	1.2474030	0.6546040	-0.1201870
C	1.2517890	-0.7382170	-0.1693750
C	2.5555350	-1.4557550	-0.2256570
C	2.8267970	-2.3956160	-1.2290120
C	4.0389010	-3.0771010	-1.2817270
C	5.0162300	-2.8332700	-0.3195590
C	4.7763900	-1.9095750	0.6940340
C	3.5607380	-1.2455430	0.7209750
C	-2.8528690	-0.9498270	-0.1767070
C	-3.7103370	-0.4905810	-1.1857770
C	-5.0418070	-0.8889980	-1.2490490
C	-5.5505440	-1.7666650	-0.2933740
C	-4.7265330	-2.2362070	0.7247670
C	-3.4040090	-1.8222070	0.7627420
F	-2.6341590	-2.2730810	1.7750390
C	0.2216120	2.9237810	-0.0583790

C	-0.5778670	3.6920250	0.7967810
C	-0.4575230	5.0784750	0.8284460
C	0.4643620	5.7211280	0.0046770
C	1.2649140	4.9668620	-0.8507210
C	1.1440460	3.5805770	-0.8819350
F	3.3446410	-0.3699230	1.7268420
H	-1.0800630	5.6575510	1.5046060
H	-1.2814980	3.1980420	1.4619190
H	1.7577760	3.0021140	-1.5675980
H	1.9813860	5.4591240	-1.5022800
H	0.5601440	6.8027800	0.0306130
H	2.2073650	1.1659560	-0.0957180
H	-2.0386850	1.5343360	-0.0894980
H	-3.3085670	0.1819550	-1.9391410
H	-5.6787430	-0.5202020	-2.0471290
H	-6.5873460	-2.0862410	-0.3371000
H	-5.0906170	-2.9132620	1.4903100
H	5.5107140	-1.7018340	1.4652330
H	5.9653700	-3.3594910	-0.3533430
H	4.2188720	-3.7965310	-2.0744950
H	2.0688980	-2.5797470	-1.9854680

3_g (wB97xd/cc-pVTZ)

P	-0.1953530	-1.7131530	-0.1702550
C	-1.4329670	-0.4892290	-0.1265450
C	-1.1727000	0.8699980	-0.1007700
C	0.0990370	1.4393820	-0.0943980
C	1.2428730	0.6492620	-0.1197450
C	1.2431100	-0.7364870	-0.1697830
C	2.5368940	-1.4632540	-0.2237240
C	2.7874070	-2.4206710	-1.2061330

C	3.9851730	-3.1129510	-1.2569180
C	4.9683210	-2.8634140	-0.3130190
C	4.7492440	-1.9219830	0.6788990
C	3.5480200	-1.2437320	0.7067490
C	-2.8521030	-0.9285280	-0.1719200
C	-3.7166200	-0.4470160	-1.1549830
C	-5.0422110	-0.8405540	-1.2131990
C	-5.5390910	-1.7348140	-0.2775890
C	-4.7085520	-2.2257250	0.7148460
C	-3.3896470	-1.8188380	0.7510060
F	-2.6162340	-2.2916940	1.7368990
C	0.2354900	2.9157840	-0.0604260
C	-0.5771030	3.6892360	0.7664810
C	-0.4447940	5.0672920	0.8017440
C	0.5016830	5.6978610	0.0090700
C	1.3142030	4.9395230	-0.8193600
C	1.1823050	3.5616150	-0.8533930
F	3.3534140	-0.3510510	1.6883540
H	-1.0777200	5.6504190	1.4567820
H	-1.3017700	3.2047420	1.4076670
H	1.8078300	2.9797700	-1.5173090
H	2.0500920	5.4228740	-1.4476440
H	0.6072680	6.7736770	0.0384560
H	2.2005050	1.1548900	-0.0924640
H	-2.0223930	1.5441570	-0.0915860
H	-3.3251410	0.2404530	-1.8932890
H	-5.6848540	-0.4546200	-1.9918720
H	-6.5722260	-2.0506840	-0.3177270
H	-5.0635980	-2.9171810	1.4653650
H	5.4902410	-1.7079650	1.4356450
H	5.9073730	-3.3980560	-0.3454140

H	4.1492970	-3.8460960	-2.0338350
H	2.0229350	-2.6109310	-1.9475790

3_h (B3LYP/6-31+G*)

P	0.0000000	1.6685540	0.0000000
C	1.3557520	0.5459650	-0.0317880
C	1.2211000	-0.8448410	-0.0357260
C	0.0000000	-1.5380310	0.0000000
C	-1.2211210	-0.8447420	0.0356790
C	-1.3557120	0.5460790	0.0317380
C	-2.7328560	1.1124040	0.1261580
C	-3.6049140	0.7187600	1.1591810
C	-4.8991130	1.2294390	1.2635740
C	-5.3614070	2.1572630	0.3257600
C	-4.5249640	2.5661200	-0.7144110
C	-3.2402570	2.0419970	-0.7907880
C	2.7329270	1.1122530	-0.1261920
C	3.6048360	0.7189060	-1.1594460
C	4.8990470	1.2295670	-1.2638160
C	5.3614820	2.1570580	-0.3257430
C	4.5251810	2.5656070	0.7146640
C	3.2404610	2.0415110	0.7910150
F	2.4608250	2.4405280	1.8316830
C	-0.0001020	-3.0263830	0.0000000
C	0.9160770	-3.7488190	0.7856870
C	0.9154330	-5.1447850	0.7866720
C	-0.0002100	-5.8492940	0.0000000
C	-0.9158000	-5.1447020	-0.7867760
C	-0.9163380	-3.7487360	-0.7857700
F	-2.4604970	2.4413400	-1.8312410
H	1.6259910	-5.6819110	1.4099850

H	1.6166130	-3.2131210	1.4210580
H	-1.6168330	-3.2129750	-1.4211320
H	-1.6263990	-5.6817640	-1.4100970
H	-0.0002520	-6.9361940	0.0000000
H	-2.1333840	-1.4376260	0.0779370
H	2.1333280	-1.4377760	-0.0780000
H	3.2415610	0.0120790	-1.9008230
H	5.5407820	0.9094820	-2.0799660
H	6.3667470	2.5625040	-0.4001940
H	4.8510560	3.2761590	1.4679100
H	-4.8507100	3.2769370	-1.4674620
H	-6.3666610	2.5627290	0.4002330
H	-5.5409470	0.9091090	2.0795490
H	-3.2417600	0.0116870	1.9003840

3_h (B3LYP/cc-pVTZ)

P	0.0000000	0.0000000	1.6525450
C	0.3760460	1.2996890	0.5361920
C	0.3316420	1.1697120	-0.8467290
C	0.0000000	0.0000000	-1.5359930
C	-0.3316420	-1.1697120	-0.8467290
C	-0.3760460	-1.2996890	0.5361920
C	-0.7006150	-2.6353930	1.1040060
C	0.0000000	-3.7784810	0.6978460
C	-0.2904240	-5.0347150	1.2103740
C	-1.3027920	-5.1836500	2.1512250
C	-2.0205800	-4.0724720	2.5722500
C	-1.7079640	-2.8305410	2.0492880
C	0.7006150	2.6353930	1.1040060
C	0.0000000	3.7784810	0.6978460
C	0.2904240	5.0347150	1.2103740

C	1.3027920	5.1836500	2.1512250
C	2.0205800	4.0724720	2.5722500
C	1.7079640	2.8305410	2.0492880
F	2.4403440	1.7743790	2.4625640
C	0.0000000	0.0000000	-3.0198110
C	1.0112500	0.6470080	-3.7392470
C	1.0123230	0.6454150	-5.1275670
C	0.0000000	0.0000000	-5.8281820
C	-1.0123230	-0.6454150	-5.1275670
C	-1.0112500	-0.6470080	-3.7392470
F	-2.4403440	-1.7743790	2.4625640
H	1.8103470	1.1428330	-5.6626970
H	1.8176520	1.1323770	-3.2062280
H	-1.8176520	-1.1323770	-3.2062280
H	-1.8103470	-1.1428330	-5.6626970
H	0.0000000	0.0000000	-6.9098460
H	-0.5654440	-2.0470320	-1.4387430
H	0.5654440	2.0470320	-1.4387430
H	-0.8000960	3.6637120	-0.0209550
H	-0.2774100	5.8937080	0.8805310
H	1.5347200	6.1584370	2.5577220
H	2.8210200	4.1495630	3.2942580
H	-2.8210200	-4.1495630	3.2942580
H	-1.5347200	-6.1584370	2.5577220
H	0.2774100	-5.8937080	0.8805310
H	0.8000960	-3.6637120	-0.0209550

3_h (M06-2X/6-31+G*)

P	-0.0000010	-1.6521050	-0.0000010
C	-1.3442450	-0.5339090	-0.0248890
C	-1.2168630	0.8538980	-0.0269170

C	0.0000010	1.5432500	0.0000000
C	1.2168630	0.8538970	0.0269160
C	1.3442440	-0.5339100	0.0248870
C	2.7187050	-1.0990650	0.1160480
C	3.6133370	-0.6573100	1.1012010
C	4.9032960	-1.1701360	1.1981950
C	5.3321120	-2.1479650	0.3015800
C	4.4694780	-2.6048810	-0.6911350
C	3.1895800	-2.0771490	-0.7615400
C	-2.7187060	-1.0990640	-0.1160490
C	-3.6133370	-0.6573110	-1.1012030
C	-4.9032960	-1.1701380	-1.1981960
C	-5.3321130	-2.1479650	-0.3015780
C	-4.4694790	-2.6048780	0.6911380
C	-3.1895810	-2.0771460	0.7615420
F	-2.3806790	-2.5174840	1.7462320
C	0.0000010	3.0294970	0.0000000
C	-0.9037920	3.7443540	0.7963750
C	-0.9032700	5.1367100	0.7972940
C	0.0000010	5.8378550	0.0000000
C	0.9032730	5.1367090	-0.7972950
C	0.9037940	3.7443540	-0.7963750
F	2.3806790	-2.5174900	-1.7462290
H	-1.6045360	5.6750550	1.4284840
H	-1.5942890	3.2035020	1.4387690
H	1.5942900	3.2035000	-1.4387690
H	1.6045380	5.6750560	-1.4284840
H	0.0000020	6.9238620	-0.0000010
H	2.1314600	1.4458080	0.0600460
H	-2.1314590	1.4458100	-0.0600470
H	-3.2695160	0.0897870	-1.8120340

H	-5.5691870	-0.8129360	-1.9774420
H	-6.3353170	-2.5568340	-0.3726520
H	-4.7687130	-3.3572370	1.4135740
H	4.7687120	-3.3572420	-1.4135700
H	6.3353160	-2.5568340	0.3726550
H	5.5691860	-0.8129320	1.9774400
H	3.2695160	0.0897890	1.8120310

3_h (M06-2X/cc-pVTZ)

P	0.0000000	1.6386900	-0.0000010
C	1.3414550	0.5253910	-0.0203600
C	1.2117870	-0.8558630	-0.0217890
C	0.0000000	-1.5420450	-0.0000010
C	-1.2117860	-0.8558630	0.0217880
C	-1.3414550	0.5253910	0.0203600
C	-2.7120550	1.0904200	0.1084590
C	-3.6160410	0.6275380	1.0669200
C	-4.8990080	1.1401180	1.1636640
C	-5.3125720	2.1382900	0.2928660
C	-4.4410030	2.6158990	-0.6728360
C	-3.1665250	2.0899270	-0.7448570
C	2.7120550	1.0904220	-0.1084600
C	3.6160390	0.6275440	-1.0669240
C	4.8990050	1.1401260	-1.1636680
C	5.3125700	2.1382940	-0.2928670
C	4.4410020	2.6158980	0.6728390
C	3.1665250	2.0899250	0.7448600
F	2.3501010	2.5539880	1.7033370
C	0.0000010	-3.0242300	0.0000000
C	0.9114100	-3.7365390	0.7793250
C	0.9100900	-5.1225150	0.7811280

C	0.0000020	-5.8204950	0.0000000
C	-0.9100880	-5.1225150	-0.7811280
C	-0.9114080	-3.7365400	-0.7793270
F	-2.3501010	2.5539960	-1.7033310
H	1.6169180	-5.6589030	1.3996080
H	1.6085780	-3.1976900	1.4078820
H	-1.6085760	-3.1976900	-1.4078830
H	-1.6169140	-5.6589050	-1.3996080
H	0.0000020	-6.9017880	0.0000000
H	-2.1220310	-1.4463980	0.0508230
H	2.1220320	-1.4463980	-0.0508230
H	3.2846520	-0.1368610	-1.7578400
H	5.5721010	0.7661670	-1.9221580
H	6.3108090	2.5471190	-0.3637280
H	4.7276570	3.3856380	1.3751130
H	-4.7276570	3.3856420	-1.3751070
H	-6.3108120	2.5471140	0.3637280
H	-5.5721050	0.7661560	1.9221510
H	-3.2846560	-0.1368690	1.7578340

3_h (wB97xd/6-31+G*)

P	0.0000000	0.0000000	1.6571020
C	0.3933440	1.2861330	0.5450360
C	0.3496320	1.1657300	-0.8420250
C	0.0000000	0.0000000	-1.5302540
C	-0.3496320	-1.1657300	-0.8420250
C	-0.3933440	-1.2861330	0.5450360
C	-0.7342530	-2.6212610	1.1131580
C	0.0000000	-3.7585090	0.7499270
C	-0.2981180	-5.0155630	1.2657330
C	-1.3495940	-5.1640610	2.1683580

C	-2.1022570	-4.0556960	2.5442590
C	-1.7825460	-2.8156250	2.0133190
C	0.7342530	2.6212610	1.1131580
C	0.0000000	3.7585090	0.7499270
C	0.2981180	5.0155630	1.2657330
C	1.3495940	5.1640610	2.1683580
C	2.1022570	4.0556960	2.5442590
C	1.7825460	2.8156250	2.0133190
F	2.5448230	1.7622410	2.3755520
C	0.0000000	0.0000000	-3.0174260
C	1.0510580	0.5872670	-3.7322680
C	1.0513920	0.5873180	-5.1244520
C	0.0000000	0.0000000	-5.8253670
C	-1.0513920	-0.5873180	-5.1244520
C	-1.0510580	-0.5872670	-3.7322680
F	-2.5448230	-1.7622410	2.3755520
H	1.8799400	1.0402790	-5.6615280
H	1.8869250	1.0269780	-3.1942290
H	-1.8869250	-1.0269780	-3.1942290
H	-1.8799400	-1.0402790	-5.6615280
H	0.0000000	0.0000000	-6.9116260
H	-0.5978220	-2.0464990	-1.4330780
H	0.5978220	2.0464990	-1.4330780
H	-0.8304150	3.6382040	0.0592030
H	-0.2949020	5.8753770	0.9695940
H	1.5858750	6.1396930	2.5821080
H	2.9322910	4.1353530	3.2383710
H	-2.9322910	-4.1353530	3.2383710
H	-1.5858750	-6.1396930	2.5821080
H	0.2949020	-5.8753770	0.9695940
H	0.8304150	-3.6382040	0.0592030

3_h (wB97xd/cc-pVTZ)

P	0.0000000	0.0000000	1.6378230
C	0.3860660	1.2856560	0.5308820
C	0.3431760	1.1618230	-0.8487800
C	0.0000000	0.0000000	-1.5333920
C	-0.3431760	-1.1618230	-0.8487800
C	-0.3860660	-1.2856560	0.5308820
C	-0.7150440	-2.6181530	1.1012740
C	0.0000000	-3.7513630	0.7132420
C	-0.2797520	-5.0018810	1.2353640
C	-1.2940620	-5.1490320	2.1688820
C	-2.0284400	-4.0457550	2.5680630
C	-1.7298580	-2.8087650	2.0323570
C	0.7150440	2.6181530	1.1012740
C	0.0000000	3.7513630	0.7132420
C	0.2797520	5.0018810	1.2353640
C	1.2940620	5.1490320	2.1688820
C	2.0284400	4.0457550	2.5680630
C	1.7298580	2.8087650	2.0323570
F	2.4726620	1.7634330	2.4197950
C	0.0000000	0.0000000	-3.0164490
C	1.0321040	0.6079910	-3.7285890
C	1.0327920	0.6072380	-5.1136900
C	0.0000000	0.0000000	-5.8109130
C	-1.0327920	-0.6072380	-5.1136900
C	-1.0321040	-0.6079910	-3.7285890
F	-2.4726620	-1.7634330	2.4197950
H	1.8477390	1.0758890	-5.6485010
H	1.8538860	1.0652810	-3.1933860
H	-1.8538860	-1.0652810	-3.1933860

H	-1.8477390	-1.0758890	-5.6485010
H	0.0000000	0.0000000	-6.8923940
H	-0.5866910	-2.0388830	-1.4387820
H	0.5866910	2.0388830	-1.4387820
H	-0.8022960	3.6331420	-0.0031240
H	-0.2988110	5.8585070	0.9196590
H	1.5146840	6.1202750	2.5891680
H	2.8306740	4.1245110	3.2875790
H	-2.8306740	-4.1245110	3.2875790
H	-1.5146840	-6.1202750	2.5891680
H	0.2988110	-5.8585070	0.9196590
H	0.8022960	-3.6331420	-0.0031240

3_i (B3LYP/6-31+G*)

P	-0.0094530	-1.6508000	-0.2647380
C	-1.3578140	-0.5222210	-0.1825650
C	-1.2133610	0.8629840	-0.0683280
C	0.0128540	1.5439880	0.0078690
C	1.2298770	0.8434300	-0.0355330
C	1.3541930	-0.5438130	-0.1504220
C	2.7293980	-1.1173590	-0.2223700
C	3.6528560	-0.6580700	-1.1807980
C	4.9463030	-1.1746710	-1.2631410
C	5.3556260	-2.1754410	-0.3770760
C	4.4674570	-2.6509460	0.5891150
C	3.1846810	-2.1192310	0.6447960
C	-2.7392490	-1.0706350	-0.3111520
C	-3.6214020	-0.5830000	-1.2942190
C	-4.9195610	-1.0768330	-1.4274950
C	-5.3755630	-2.0822090	-0.5698560
C	-4.5291590	-2.5851290	0.4197050

C	-3.2406110	-2.0758410	0.5263330
F	-2.4512820	-2.5685090	1.5173130
C	0.0226600	3.0255770	0.1493190
C	-0.8859290	3.6762470	1.0035640
C	-0.8752780	5.0657120	1.1375750
C	0.0430450	5.8358690	0.4184740
C	0.9514680	5.2033610	-0.4349480
C	0.9412960	3.8138550	-0.5672990
F	2.3539760	-2.5854620	1.6142260
H	-1.5794950	5.5456850	1.8125310
H	-1.5870960	3.0871850	1.5888140
H	1.6370700	3.3365120	-1.2525250
H	1.6647960	5.7926130	-1.0058160
H	0.0513220	6.9177180	0.5227720
H	2.1458100	1.4229800	0.0669580
H	-2.1221880	1.4620620	-0.0428630
H	-3.2632500	0.1856700	-1.9740120
H	-5.5695690	-0.6823480	-2.2035560
H	-6.3838430	-2.4750740	-0.6674800
H	-4.8498650	-3.3581520	1.1111050
H	4.7514250	-3.4193930	1.3013390
H	6.3599740	-2.5858910	-0.4345920
H	5.6290900	-0.8018020	-2.0215620
H	3.3306570	0.1062250	-1.8831780

3_i (B3LYP/cc-pVTZ)

P	-0.0120820	-1.6350190	-0.2597140
C	-1.3556590	-0.5103380	-0.1794820
C	-1.2067190	0.8666780	-0.0652610
C	0.0146560	1.5421330	0.0096580
C	1.2247760	0.8437590	-0.0338070

C	1.3499670	-0.5359940	-0.1464830
C	2.7187390	-1.1122250	-0.2174160
C	3.6516330	-0.6343790	-1.1468910
C	4.9360170	-1.1528530	-1.2277440
C	5.3275450	-2.1733520	-0.3689840
C	4.4301170	-2.6672660	0.5678550
C	3.1539020	-2.1361520	0.6248100
C	-2.7321280	-1.0582930	-0.3064540
C	-3.6215200	-0.5556780	-1.2650060
C	-4.9115600	-1.0495540	-1.3959550
C	-5.3527850	-2.0689260	-0.5601660
C	-4.4992740	-2.5864640	0.4045390
C	-3.2164580	-2.0797790	0.5115940
F	-2.4206890	-2.5884310	1.4753040
C	0.0267150	3.0191420	0.1501760
C	-0.8871310	3.6706290	0.9865310
C	-0.8733070	5.0524300	1.1202650
C	0.0523310	5.8153610	0.4179620
C	0.9654720	5.1831510	-0.4178820
C	0.9535790	3.8011870	-0.5487740
F	2.3148060	-2.6224520	1.5627170
H	-1.5818820	5.5329420	1.7817920
H	-1.5960950	3.0874580	1.5584340
H	1.6550560	3.3242930	-1.2199940
H	1.6850560	5.7676110	-0.9757800
H	0.0625050	6.8919860	0.5216000
H	2.1364980	1.4211940	0.0658990
H	-2.1095930	1.4657900	-0.0387900
H	-3.2758730	0.2256310	-1.9284360
H	-5.5678110	-0.6434820	-2.1532870
H	-6.3557130	-2.4615440	-0.6562870

H	-4.8090740	-3.3718440	1.0791800
H	4.7006710	-3.4526330	1.2591670
H	6.3257730	-2.5851430	-0.4256180
H	5.6266840	-0.7653220	-1.9639060
H	3.3445460	0.1469800	-1.8289570

3_i (M06-2X/6-31+G*)

P	-0.0282720	-1.6340220	-0.2453060
C	-1.3529670	-0.4953390	-0.1734290
C	-1.2032630	0.8854730	-0.0631860
C	0.0255230	1.5498850	0.0176130
C	1.2306500	0.8401730	-0.0178610
C	1.3345000	-0.5451740	-0.1320610
C	2.7009710	-1.1304820	-0.2087830
C	3.6467200	-0.6294110	-1.1148450
C	4.9297480	-1.1611310	-1.2001920
C	5.3003430	-2.2185790	-0.3703420
C	4.3863780	-2.7358480	0.5437100
C	3.1145440	-2.1875550	0.6039180
C	-2.7347230	-1.0336520	-0.3069530
C	-3.6200450	-0.5236960	-1.2670920
C	-4.9145040	-1.0175240	-1.4001330
C	-5.3567600	-2.0431500	-0.5652800
C	-4.5037330	-2.5668590	0.4021300
C	-3.2189610	-2.0574250	0.5090890
F	-2.4192980	-2.5635350	1.4684970
C	0.0521610	3.0294570	0.1537370
C	-0.8494510	3.6879580	0.9994440
C	-0.8209800	5.0742000	1.1284250
C	0.1088230	5.8263720	0.4122760
C	1.0103380	5.1819230	-0.4332040

C	0.9818630	3.7958250	-0.5609040
F	2.2563360	-2.6893370	1.5135290
H	-1.5208730	5.5660270	1.7979240
H	-1.5591020	3.1059330	1.5819370
H	1.6724540	3.3028950	-1.2408130
H	1.7331000	5.7600920	-1.0018140
H	0.1315080	6.9074180	0.5135170
H	2.1545470	1.4085320	0.0881900
H	-2.1082650	1.4926400	-0.0450040
H	-3.2659150	0.2623280	-1.9293260
H	-5.5738990	-0.6079200	-2.1588740
H	-6.3633380	-2.4374590	-0.6648750
H	-4.8137370	-3.3580540	1.0767460
H	4.6388820	-3.5517990	1.2128580
H	6.2977850	-2.6426720	-0.4329040
H	5.6358560	-0.7562340	-1.9182970
H	3.3486690	0.1818030	-1.7743890

3_i (M06-2X/cc-pVTZ)

P	-0.0203350	-1.6241770	-0.2421730
C	-1.3476210	-0.4963270	-0.1669560
C	-1.2005170	0.8784200	-0.0546960
C	0.0199590	1.5456600	0.0205390
C	1.2229590	0.8446910	-0.0192970
C	1.3349180	-0.5338120	-0.1311740
C	2.7003910	-1.1120220	-0.2059320
C	3.6512460	-0.5872730	-1.0838810
C	4.9318940	-1.1078110	-1.1660820
C	5.2964880	-2.1768610	-0.3596500
C	4.3777610	-2.7178210	0.5253630
C	3.1061550	-2.1828610	0.5835410

C	-2.7247390	-1.0365260	-0.2971780
C	-3.6289210	-0.4876310	-1.2090360
C	-4.9182950	-0.9768390	-1.3383680
C	-5.3386340	-2.0370230	-0.5474310
C	-4.4670390	-2.6007920	0.3701760
C	-3.1858510	-2.0970680	0.4755580
F	-2.3701870	-2.6479980	1.3866120
C	0.0393470	3.0213520	0.1561770
C	-0.8771190	3.6747060	0.9799240
C	-0.8561200	5.0546450	1.1085510
C	0.0798610	5.8066920	0.4129470
C	0.9959430	5.1681720	-0.4107590
C	0.9762410	3.7881330	-0.5366440
F	2.2442400	-2.7124740	1.4635970
H	-1.5674880	5.5425300	1.7610720
H	-1.5932630	3.0928140	1.5453910
H	1.6793120	3.2991790	-1.1986580
H	1.7240470	5.7466270	-0.9631430
H	0.0959880	6.8832530	0.5132750
H	2.1402300	1.4159950	0.0813890
H	-2.1035010	1.4802460	-0.0311070
H	-3.2937210	0.3275300	-1.8372400
H	-5.5914650	-0.5362480	-2.0602040
H	-6.3419580	-2.4277540	-0.6438270
H	-4.7580120	-3.4218140	1.0097200
H	4.6250590	-3.5445450	1.1759130
H	6.2929020	-2.5919350	-0.4187660
H	5.6415760	-0.6848970	-1.8631020
H	3.3590060	0.2350900	-1.7241450

3_i (wB97xd/6-31+G*)

P	-0.0207250	-1.6446420	-0.2642680
C	-1.3507040	-0.5178870	-0.1825470
C	-1.2061970	0.8623800	-0.0657920
C	0.0198910	1.5299510	0.0147940
C	1.2279820	0.8270990	-0.0224390
C	1.3375960	-0.5566860	-0.1415630
C	2.7079630	-1.1373720	-0.2198200
C	3.6002540	-0.7224350	-1.2175280
C	4.8859870	-1.2452950	-1.3103410
C	5.3121200	-2.2056030	-0.3945530
C	4.4520780	-2.6344280	0.6117050
C	3.1759950	-2.0964200	0.6791640
C	-2.7316190	-1.0627720	-0.3170720
C	-3.5706980	-0.6396300	-1.3565760
C	-4.8624580	-1.1366820	-1.4984050
C	-5.3480650	-2.0774060	-0.5919040
C	-4.5418730	-2.5121510	0.4556260
C	-3.2586370	-2.0003330	0.5716540
F	-2.5051460	-2.4204050	1.6086940
C	0.0394850	3.0110170	0.1469290
C	-0.8187540	3.6610000	1.0419650
C	-0.7994960	5.0474580	1.1653040
C	0.0768630	5.8079380	0.3937590
C	0.9350210	5.1719160	-0.5010460
C	0.9161480	3.7853720	-0.6227540
F	2.3706280	-2.5112880	1.6782390
H	-1.4657960	5.5338220	1.8721260
H	-1.4879090	3.0730400	1.6648030
H	1.5742610	3.2986600	-1.3381950
H	1.6155730	5.7574130	-1.1127770
H	0.0923780	6.8896580	0.4910560

H	2.1496500	1.3987580	0.0778330
H	-2.1135220	1.4650910	-0.0465810
H	-3.1844750	0.0823940	-2.0713120
H	-5.4857280	-0.7950780	-2.3190820
H	-6.3527460	-2.4754350	-0.6978500
H	-4.8904360	-3.2361430	1.1845300
H	4.7539390	-3.3730210	1.3467270
H	6.3118590	-2.6239600	-0.4612650
H	5.5515500	-0.9085210	-2.0991310
H	3.2596600	0.0140040	-1.9406090

3_i (wB97xd/cc-pVTZ)

P	-0.0381100	-1.6298110	-0.2557620
C	-1.3542840	-0.4951200	-0.1791060
C	-1.1952130	0.8763210	-0.0645200
C	0.0315550	1.5287700	0.0146010
C	1.2272550	0.8181630	-0.0217770
C	1.3276940	-0.5596120	-0.1360620
C	2.6880460	-1.1526770	-0.2140250
C	3.5923470	-0.7230370	-1.1850350
C	4.8656550	-1.2565480	-1.2794040
C	5.2684460	-2.2413640	-0.3907000
C	4.3969690	-2.6843800	0.5892650
C	3.1308060	-2.1380290	0.6611360
C	-2.7340950	-1.0316580	-0.3133830
C	-3.5688730	-0.6040170	-1.3452140
C	-4.8537850	-1.0978350	-1.4903780
C	-5.3377800	-2.0377180	-0.5935600
C	-4.5367210	-2.4746600	0.4473930
C	-3.2577270	-1.9690060	0.5693940
F	-2.5108170	-2.3922230	1.5969600

C	0.0664170	3.0052720	0.1455200
C	-0.7919300	3.6636310	1.0237970
C	-0.7549610	5.0423460	1.1492000
C	0.1376530	5.7879050	0.3948190
C	0.9947410	5.1444270	-0.4845070
C	0.9597420	3.7654930	-0.6068610
F	2.3166560	-2.5683270	1.6328700
H	-1.4212280	5.5352940	1.8441030
H	-1.4752930	3.0865130	1.6327360
H	1.6198840	3.2723810	-1.3085270
H	1.6879270	5.7192650	-1.0835720
H	0.1674030	6.8643070	0.4941640
H	2.1495900	1.3808240	0.0736550
H	-2.0922330	1.4862060	-0.0457180
H	-3.1845860	0.1189430	-2.0527780
H	-5.4738260	-0.7535610	-2.3060590
H	-6.3376200	-2.4341420	-0.7027950
H	-4.8848890	-3.1987180	1.1698030
H	4.6816880	-3.4421360	1.3048810
H	6.2591080	-2.6686480	-0.4591910
H	5.5409370	-0.9081960	-2.0480520
H	3.2704930	0.0337800	-1.8882350

3_j (B3LYP/6-31+G*)

P	0.0000000	1.6708190	0.0000000
C	-1.3557770	0.5483990	-0.0019950
C	-1.2218220	-0.8425720	-0.0074680
C	0.0000000	-1.5359730	0.0000000
C	1.2218390	-0.8425120	0.0074480
C	1.3557710	0.5484660	0.0019860
C	2.7350040	1.1163040	0.0387120

C	3.6406540	0.7436660	1.0501990
C	4.9375590	1.2555670	1.1014510
C	5.3684090	2.1631850	0.1294780
C	4.4980690	2.5510910	-0.8906520
C	3.2114180	2.0265440	-0.9135210
C	-2.7350270	1.1162270	-0.0387460
C	-3.6405070	0.7439420	-1.0505060
C	-4.9374160	1.2558390	-1.1017600
C	-5.3684280	2.1630760	-0.1295030
C	-4.4982530	2.5506150	0.8909080
C	-3.2115920	2.0260920	0.9137670
F	-2.3978830	2.4041390	1.9357060
C	0.0000000	-3.0242600	0.0000000
C	-0.8924720	-3.7474210	-0.8117880
C	-0.8920550	-5.1432770	-0.8129930
C	0.0001250	-5.8480280	0.0000000
C	0.8922700	-5.1432660	0.8128520
C	0.8926250	-3.7474090	0.8116970
F	2.3975520	2.4049840	-1.9351910
H	-1.5842790	-5.6802900	-1.4567000
H	-1.5744670	-3.2122200	-1.4673540
H	1.5745890	-3.2122020	1.4672900
H	1.5845150	-5.6802700	1.4565450
H	0.0001500	-6.9348960	-0.0001040
H	2.1348420	-1.4349060	-0.0233450
H	-2.1348120	-1.4349870	0.0232850
H	-3.3011880	0.0532810	-1.8180060
H	-5.6059930	0.9526910	-1.9026880
H	-6.3757000	2.5690680	-0.1620240
H	-4.7993680	3.2448650	1.6692240
H	4.7990400	3.2456490	-1.6687490

H	6.3756740	2.5691930	0.1620050
H	5.6062580	0.9521320	1.9021690
H	3.3014670	0.0527290	1.8175070

3_j (B3LYP/cc-pVTZ)

P	0.0000000	0.0000000	1.6552320
C	0.0000000	1.3527690	0.5387790
C	0.0073320	1.2158620	-0.8443680
C	0.0000000	0.0000000	-1.5338760
C	-0.0073320	-1.2158620	-0.8443680
C	0.0000000	-1.3527690	0.5387790
C	-0.0351550	-2.7268210	1.1061940
C	-1.0153310	-3.6435220	0.7042010
C	-1.0645210	-4.9329660	1.2139160
C	-0.1217520	-5.3461940	2.1480200
C	0.8664340	-4.4649980	2.5653850
C	0.8902430	-3.1833670	2.0450850
C	0.0351550	2.7268210	1.1061940
C	1.0153310	3.6435220	0.7042010
C	1.0645210	4.9329660	1.2139160
C	0.1217520	5.3461940	2.1480200
C	-0.8664340	4.4649980	2.5653850
C	-0.8902430	3.1833670	2.0450850
F	-1.8783670	2.3596850	2.4541880
C	0.0000000	0.0000000	-3.0175310
C	0.7955930	0.8982700	-3.7379560
C	0.7972880	0.8971890	-5.1261820
C	0.0000000	0.0000000	-5.8271670
C	-0.7972880	-0.8971890	-5.1261820
C	-0.7955930	-0.8982700	-3.7379560
F	1.8783670	-2.3596850	2.4541880

H	1.4290430	1.5939370	-5.6610300
H	1.4387340	1.5862310	-3.2059960
H	-1.4387340	-1.5862310	-3.2059960
H	-1.4290430	-1.5939370	-5.6610300
H	0.0000000	0.0000000	-6.9087890
H	0.0211630	-2.1236880	-1.4358120
H	-0.0211630	2.1236880	-1.4358120
H	1.7611030	3.3193690	-0.0090290
H	1.8410940	5.6107450	0.8873450
H	0.1528760	6.3486650	2.5521470
H	-1.6223940	4.7526110	3.2821190
H	1.6223940	-4.7526110	3.2821190
H	-0.1528760	-6.3486650	2.5521470
H	-1.8410940	-5.6107450	0.8873450
H	-1.7611030	-3.3193690	-0.0090290

3_j (M06-2X/6-31+G*)

P	0.0000020	-1.6555410	-0.0000010
C	1.3442260	-0.5371930	0.0093890
C	1.2171200	0.8508320	0.0066430
C	-0.0000010	1.5404060	0.0000000
C	-1.2171200	0.8508290	-0.0066420
C	-1.3442250	-0.5371960	-0.0093890
C	-2.7217130	-1.1010170	0.0245970
C	-3.6542700	-0.6563220	0.9726760
C	-4.9490870	-1.1635690	1.0175670
C	-5.3451120	-2.1396100	0.1041750
C	-4.4445400	-2.5999360	-0.8525950
C	-3.1607060	-2.0772360	-0.8714500
C	2.7217160	-1.1010130	-0.0245980
C	3.6542690	-0.6563220	-0.9726810

C	4.9490870	-1.1635680	-1.0175730
C	5.3451150	-2.1396030	-0.1041750
C	4.4445450	-2.5999250	0.8526000
C	3.1607110	-2.0772260	0.8714550
F	2.3163090	-2.5197160	1.8245260
C	-0.0000030	3.0265230	0.0000000
C	0.8984040	3.7422410	-0.8016430
C	0.8983940	5.1344630	-0.8025380
C	-0.0000060	5.8357540	-0.0000010
C	-0.8984040	5.1344620	0.8025360
C	-0.8984110	3.7422400	0.8016420
F	-2.3163020	-2.5197310	-1.8245180
H	1.5960050	5.6728130	-1.4377500
H	1.5848430	3.2018630	-1.4488000
H	-1.5848490	3.2018600	1.4488000
H	-1.5960160	5.6728100	1.4377480
H	-0.0000070	6.9217310	-0.0000020
H	-2.1317740	1.4418930	-0.0493930
H	2.1317730	1.4418970	0.0493940
H	3.3360920	0.0893620	-1.6970180
H	5.6447300	-0.8027750	-1.7686550
H	6.3524230	-2.5435070	-0.1334300
H	4.7167900	-3.3504770	1.5875800
H	-4.7167810	-3.3504930	-1.5875720
H	-6.3524200	-2.5435150	0.1334300
H	-5.6447320	-0.8027730	1.7686460
H	-3.3360960	0.0893660	1.6970100

3_j (M06-2X/cc-pVTZ)

P	0.0000000	0.0000000	1.6415850
C	-0.0064750	1.3415480	0.5282730

C	0.0000000	1.2119840	-0.8531660
C	0.0000000	0.0000000	-1.5395240
C	0.0000000	-1.2119840	-0.8531660
C	0.0064750	-1.3415480	0.5282730
C	-0.0286200	-2.7147920	1.0923790
C	-0.9521520	-3.6536070	0.6275430
C	-0.9995250	-4.9411710	1.1350890
C	-0.1126340	-5.3247380	2.1310820
C	0.8192550	-4.4182980	2.6111670
C	0.8424040	-3.1400970	2.0896170
C	0.0286200	2.7147920	1.0923790
C	0.9521520	3.6536070	0.6275430
C	0.9995250	4.9411710	1.1350890
C	0.1126340	5.3247380	2.1310820
C	-0.8192550	4.4182980	2.6111670
C	-0.8424040	3.1400970	2.0896170
F	-1.7704040	2.2903220	2.5549550
C	0.0000000	0.0000000	-3.0215460
C	0.7864070	0.9046890	-3.7346510
C	0.7881380	0.9038810	-5.1205120
C	0.0000000	0.0000000	-5.8186260
C	-0.7881380	-0.9038810	-5.1205120
C	-0.7864070	-0.9046890	-3.7346510
F	1.7704040	-2.2903220	2.5549550
H	1.4120820	1.6058720	-5.6569360
H	1.4209470	1.5967790	-3.1962230
H	-1.4209470	-1.5967790	-3.1962230
H	-1.4120820	-1.6058720	-5.6569360
H	0.0000000	0.0000000	-6.8999040
H	0.0345020	-2.1222510	-1.4432690
H	-0.0345020	2.1222510	-1.4432690

H	1.6559300	3.3459150	-0.1351410
H	1.7318450	5.6414890	0.7587740
H	0.1440410	6.3267200	2.5356080
H	-1.5334050	4.6802860	3.3788170
H	1.5334050	-4.6802860	3.3788170
H	-0.1440410	-6.3267200	2.5356080
H	-1.7318450	-5.6414890	0.7587740
H	-1.6559300	-3.3459150	-0.1351410

3_j (wB97xd/6-31+G*)

P	0.0000000	0.0000000	1.6641780
C	0.0000000	1.3446090	0.5518120
C	-0.0013380	1.2169160	-0.8355850
C	0.0000000	0.0000000	-1.5238440
C	0.0013380	-1.2169160	-0.8355850
C	0.0000000	-1.3446090	0.5518120
C	-0.0502440	-2.7230710	1.1161160
C	-1.0770080	-3.6026180	0.7465730
C	-1.1479700	-4.8964940	1.2520530
C	-0.1809180	-5.3443940	2.1500020
C	0.8546810	-4.4977120	2.5331030
C	0.8997940	-3.2134450	2.0127490
C	0.0502440	2.7230710	1.1161160
C	1.0770080	3.6026180	0.7465730
C	1.1479700	4.8964940	1.2520530
C	0.1809180	5.3443940	2.1500020
C	-0.8546810	4.4977120	2.5331030
C	-0.8997940	3.2134450	2.0127490
F	-1.9288630	2.4221890	2.3813590
C	0.0000000	0.0000000	-3.0108550
C	0.8365570	0.8648810	-3.7269230

C	0.8371310	0.8651930	-5.1188200
C	0.0000000	0.0000000	-5.8204140
C	-0.8371310	-0.8651930	-5.1188200
C	-0.8365570	-0.8648810	-3.7269230
F	1.9288630	-2.4221890	2.3813590
H	1.4992770	1.5375090	-5.6570040
H	1.5096710	1.5268840	-3.1881960
H	-1.5096710	-1.5268840	-3.1881960
H	-1.4992770	-1.5375090	-5.6570040
H	0.0000000	0.0000000	-6.9066010
H	0.0357040	-2.1318400	-1.4256480
H	-0.0357040	2.1318400	-1.4256480
H	1.8398410	3.2455920	0.0597140
H	1.9600470	5.5510400	0.9509150
H	0.2301600	6.3505290	2.5552400
H	-1.6286140	4.8159770	3.2235680
H	1.6286140	-4.8159770	3.2235680
H	-0.2301600	-6.3505290	2.5552400
H	-1.9600470	-5.5510400	0.9509150
H	-1.8398410	-3.2455920	0.0597140

3_j (wB97xd/cc-pVTZ)

P	0.0000000	0.0000000	1.6499300
C	-0.0009750	1.3419760	0.5424480
C	0.0000000	1.2111040	-0.8375390
C	0.0000000	0.0000000	-1.5222270
C	0.0000000	-1.2111040	-0.8375390
C	0.0009750	-1.3419760	0.5424480
C	-0.0491160	-2.7160330	1.1064800
C	-1.0482580	-3.6047390	0.7090800
C	-1.1210580	-4.8910320	1.2136200

C	-0.1826020	-5.3235590	2.1377380
C	0.8257010	-4.4684940	2.5478530
C	0.8759130	-3.1893440	2.0301790
C	0.0491160	2.7160330	1.1064800
C	1.0482580	3.6047390	0.7090800
C	1.1210580	4.8910320	1.2136200
C	0.1826020	5.3235590	2.1377380
C	-0.8257010	4.4684940	2.5478530
C	-0.8759130	3.1893440	2.0301790
F	-1.8769650	2.3926500	2.4265570
C	0.0000000	0.0000000	-3.0049710
C	0.8175940	0.8743910	-3.7184750
C	0.8187630	0.8739960	-5.1032490
C	0.0000000	0.0000000	-5.8014510
C	-0.8187630	-0.8739960	-5.1032490
C	-0.8175940	-0.8743910	-3.7184750
F	1.8769650	-2.3926500	2.4265570
H	1.4671940	1.5534430	-5.6396210
H	1.4753990	1.5454500	-3.1818380
H	-1.4753990	-1.5454500	-3.1818380
H	-1.4671940	-1.5534430	-5.6396210
H	0.0000000	0.0000000	-6.8828390
H	0.0298050	-2.1213350	-1.4266370
H	-0.0298050	2.1213350	-1.4266370
H	1.7895340	3.2607560	-0.0001100
H	1.9118780	5.5530600	0.8903700
H	0.2340190	6.3243710	2.5432440
H	-1.5783900	4.7753680	3.2596530
H	1.5783900	-4.7753680	3.2596530
H	-0.2340190	-6.3243710	2.5432440
H	-1.9118780	-5.5530600	0.8903700

H	-1.7895340	-3.2607560	-0.0001100

3_ω_TS_a (B3LYP/6-31+G*)

P	0.1904110	-1.7797100	0.1245720
C	1.4315330	-0.5371710	0.1607730
C	1.1772800	0.8308310	0.0865310
C	-0.1013860	1.4036820	-0.0425240
C	-1.2513000	0.6008890	-0.1068270
C	-1.2611010	-0.7957390	-0.0505440
C	-2.5560070	-1.5236240	-0.1564800
C	-2.7194090	-2.6092700	-1.0387100
C	-3.9255240	-3.3041720	-1.1348220
C	-5.0130460	-2.9316160	-0.3403900
C	-4.8860160	-1.8635900	0.5504000
C	-3.6740580	-1.1898320	0.6218430
C	2.8470280	-0.9943650	0.3289210
C	3.4418350	-1.0960200	1.5976630
C	4.7642960	-1.5183160	1.7500280
C	5.5244400	-1.8536040	0.6258840
C	4.9604990	-1.7651490	-0.6485350
C	3.6423660	-1.3406500	-0.7673920
F	3.1099420	-1.2549370	-2.0165460
C	-0.2381630	2.8835610	-0.1152210
C	0.6607610	3.6577170	-0.8709840
C	0.5292940	5.0457660	-0.9385230
C	-0.5021050	5.6897750	-0.2490990
C	-1.4018130	4.9330550	0.5069900
C	-1.2721120	3.5448170	0.5723630
F	-3.5784580	-0.1676070	1.5188050
H	1.2287720	5.6233010	-1.5377510
H	1.4512690	3.1667660	-1.4325830

H	-1.9633510	2.9695600	1.1827140
H	-2.2024650	5.4240930	1.0544510
H	-0.6038380	6.7707090	-0.3008690
H	-2.2033890	1.1075140	-0.2445190
H	2.0287680	1.5058630	0.1546700
H	2.8467030	-0.8397610	2.4702830
H	5.1974750	-1.5877410	2.7439250
H	6.5533370	-2.1848800	0.7366410
H	5.5208290	-2.0176360	-1.5434250
H	-5.7039000	-1.5504270	1.1919330
H	-5.9564290	-3.4660340	-0.4102240
H	-4.0156820	-4.1312510	-1.8333640
H	-1.8813720	-2.8933780	-1.6688790

3_ω_TS_b (B3LYP/6-31+G*)

P	0.0840750	-1.8316990	0.0075950
C	-1.3414460	-0.7722260	-0.0087340
C	-1.2127040	0.6231480	0.0264480
C	-0.0047050	1.3413180	0.1030600
C	1.2393180	0.6990380	0.1500500
C	1.4067300	-0.6867910	0.1204300
C	2.7777030	-1.2641260	0.1972060
C	3.0949320	-2.2976080	1.0995420
C	4.3746610	-2.8499770	1.1651420
C	5.3832350	-2.3810450	0.3192250
C	5.1039060	-1.3600320	-0.5916520
C	3.8216380	-0.8288300	-0.6321220
C	-2.6827400	-1.4382770	-0.0892890
C	-2.7794250	-2.8514150	-0.1103580
C	-3.9883300	-3.5351570	-0.1837920
C	-5.1923490	-2.8278390	-0.2404340

C	-5.1537880	-1.4368060	-0.2205400
C	-3.9287850	-0.7824630	-0.1465080
F	-4.0099430	0.5809880	-0.1306970
C	-0.0531350	2.8294430	0.1395860
C	-1.0186580	3.5053650	0.9071610
C	-1.0589660	4.9001960	0.9411160
C	-0.1369000	5.6495920	0.2050220
C	0.8267890	4.9912640	-0.5641650
C	0.8692640	3.5963800	-0.5952660
F	3.5781280	0.1498170	-1.5486300
H	-1.8079090	5.4012950	1.5492890
H	-1.7279550	2.9348730	1.5008550
H	1.6076620	3.0964020	-1.2166700
H	1.5426720	5.5636310	-1.1488230
H	-0.1694960	6.7357760	0.2299760
H	2.1259260	1.3199240	0.2497750
H	-2.1101550	1.2215990	-0.0291250
H	-1.8662090	-3.4349560	-0.0670530
H	-3.9869910	-4.6214340	-0.1965080
H	-6.1446190	-3.3472650	-0.2985340
H	-6.0565500	-0.8352620	-0.2616310
H	5.8572830	-0.9758550	-1.2723730
H	6.3830180	-2.8038950	0.3649830
H	4.5835330	-3.6407210	1.8801870
H	2.3175480	-2.6547730	1.7689010

3_ω_TS_c (B3LYP/6-31+G*)

P	0.1960870	-1.7796280	-0.1493640
C	1.4430640	-0.5422510	-0.1403830
C	1.1911530	0.8282350	-0.1374620
C	-0.0904950	1.4086060	-0.1611340

C	-1.2457590	0.6114530	-0.1896130
C	-1.2588990	-0.7863760	-0.1938060
C	-2.5613520	-1.5062610	-0.2523960
C	-2.7802200	-2.5594710	-1.1615950
C	-3.9923860	-3.2485260	-1.2125300
C	-5.0307540	-2.9026400	-0.3437760
C	-4.8482120	-1.8670920	0.5751320
C	-3.6316810	-1.1981300	0.6002860
C	2.8647880	-1.0107630	-0.1256510
C	3.6068560	-1.1398480	-1.3117300
C	4.9329360	-1.5775430	-1.2976530
C	5.5485190	-1.9008350	-0.0849100
C	4.8368680	-1.7837720	1.1107700
C	3.5189310	-1.3444820	1.0641620
F	2.8429100	-1.2308710	2.2380300
C	-0.2252300	2.8904980	-0.1595040
C	0.6338360	3.6976390	-0.9271290
C	0.5044740	5.0874500	-0.9243350
C	-0.4855160	5.7006980	-0.1513290
C	-1.3450450	4.9112810	0.6179360
C	-1.2172200	3.5213750	0.6131800
F	-3.4815390	-0.2084010	1.5251150
H	1.1730780	5.6908570	-1.5332770
H	1.3921780	3.2323660	-1.5515540
H	-1.8749250	2.9201790	1.2353330
H	-2.1120360	5.3777860	1.2310570
H	-0.5856350	6.7830000	-0.1479420
H	-2.2021120	1.1253760	-0.2468450
H	2.0481940	1.4978440	-0.0887870
H	3.1235070	-0.8947370	-2.2539700
H	5.4813050	-1.6687550	-2.2310990

H	6.5792200	-2.2442570	-0.0657440
H	5.2828710	-2.0249390	2.0706650
H	-5.6262270	-1.5756160	1.2739010
H	-5.9784600	-3.4328920	-0.3772920
H	-4.1257370	-4.0503330	-1.9333020
H	-1.9808230	-2.8234740	-1.8481700

3_ω_TS_d (B3LYP/6-31+G*)

P	-0.2002730	-1.7032560	0.0153990
C	-1.4739040	-0.4622520	-0.0119970
C	-1.1533440	0.9045990	0.0175610
C	0.1286950	1.4672290	0.0940300
C	1.2814470	0.6695410	0.1432500
C	1.2603710	-0.7232330	0.1217920
C	2.5434840	-1.4761070	0.2031410
C	2.7178430	-2.5432400	1.1054960
C	3.9138610	-3.2584130	1.1772730
C	4.9798780	-2.9257040	0.3374740
C	4.8415190	-1.8766920	-0.5740440
C	3.6401210	-1.1821980	-0.6204400
C	-2.9197980	-0.8541110	-0.0922590
C	-3.9616340	0.1048980	-0.1137860
C	-5.3094710	-0.2364940	-0.1834530
C	-5.6959620	-1.5781830	-0.2356990
C	-4.7101920	-2.5608430	-0.2165640
C	-3.3735660	-2.1860900	-0.1471350
F	-2.4763300	-3.2067890	-0.1318300
C	0.2691890	2.9496780	0.1282200
C	-0.5753860	3.7405880	0.9276980
C	-0.4393110	5.1297350	0.9586210
C	0.5438730	5.7576290	0.1888070

C	1.3909110	4.9838580	-0.6097530
C	1.2562090	3.5946550	-0.6384960
F	3.5329710	-0.1787390	-1.5372910
H	-1.0957530	5.7205490	1.5926980
H	-1.3245120	3.2616180	1.5532410
H	1.9058280	3.0048210	-1.2799370
H	2.1541760	5.4621780	-1.2184370
H	0.6499350	6.8391240	0.2122080
H	2.2407680	1.1725280	0.2369560
H	-1.9587340	1.6275820	-0.0448870
H	-3.7216760	1.1598370	-0.0750260
H	-6.0574330	0.5513590	-0.1970010
H	-6.7446260	-1.8560730	-0.2904010
H	-4.9491800	-3.6189990	-0.2548400
H	5.6422440	-1.5942400	-1.2505330
H	5.9151590	-3.4763550	0.3878570
H	4.0127290	-4.0707400	1.8918110
H	1.8964060	-2.7977150	1.7689750

3_θ_TS_a (B3LYP/6-31+G*)

P	0.0000000	0.0000000	1.8357090
C	-0.2950970	-1.3305870	0.7215540
C	-0.2700440	-1.1959930	-0.6705170
C	0.0000000	0.0000000	-1.3501780
C	0.2700440	1.1959930	-0.6705170
C	0.2950970	1.3305870	0.7215540
C	0.5695690	2.6629730	1.3277120
C	-0.2581250	3.1981420	2.3336040
C	0.0000000	4.4400160	2.9151320
C	1.1070120	5.1878080	2.5051450
C	1.9534860	4.6856800	1.5143930

C	1.6695730	3.4478960	0.9524930
C	-0.5695690	-2.6629730	1.3277120
C	0.2581250	-3.1981420	2.3336040
C	0.0000000	-4.4400160	2.9151320
C	-1.1070120	-5.1878080	2.5051450
C	-1.9534860	-4.6856800	1.5143930
C	-1.6695730	-3.4478960	0.9524930
F	-2.5293550	-2.9766900	0.0069680
C	0.0000000	0.0000000	-2.8486630
C	1.1733180	-0.2881550	-3.5622140
C	1.1732830	-0.2884360	-4.9598300
C	0.0000000	0.0000000	-5.6622190
C	-1.1732830	0.2884360	-4.9598300
C	-1.1733180	0.2881550	-3.5622140
F	2.5293550	2.9766900	0.0069680
H	2.0906870	-0.5127700	-5.4983800
H	2.0883730	-0.5101770	-3.0185250
H	-2.0883730	0.5101770	-3.0185250
H	-2.0906870	0.5127700	-5.4983800
H	0.0000000	0.0000000	-6.7492680
H	0.4599300	2.0793120	-1.2765050
H	-0.4599300	-2.0793120	-1.2765050
H	1.1276910	-2.6265400	2.6455840
H	0.6662130	-4.8237700	3.6826470
H	-1.3144170	-6.1566500	2.9510320
H	-2.8291580	-5.2312660	1.1765990
H	2.8291580	5.2312660	1.1765990
H	1.3144170	6.1566500	2.9510320
H	-0.6662130	4.8237700	3.6826470
H	-1.1276910	2.6265400	2.6455840

3_θ_TS_b (B3LYP/6-31+G*)

P	0.0000000	0.0000000	1.8638490
C	-0.4010430	-1.2907790	0.7384340
C	-0.3659240	-1.1595880	-0.6504760
C	0.0000000	0.0000000	-1.3605270
C	0.3659240	1.1595880	-0.6504760
C	0.4010430	1.2907790	0.7384340
C	0.7849750	2.6009520	1.3365510
C	0.0000000	3.2101650	2.3345020
C	0.3587930	4.4291200	2.9111980
C	1.5274820	5.0782960	2.5044530
C	2.3337670	4.5008790	1.5212720
C	1.9490730	3.2883910	0.9639680
C	-0.7849750	-2.6009520	1.3365510
C	0.0000000	-3.2101650	2.3345020
C	-0.3587930	-4.4291200	2.9111980
C	-1.5274820	-5.0782960	2.5044530
C	-2.3337670	-4.5008790	1.5212720
C	-1.9490730	-3.2883910	0.9639680
F	-2.7705480	-2.7427750	0.0235890
C	0.0000000	0.0000000	-2.8594870
C	-0.3303280	-1.1534110	-3.6011380
C	-0.3305290	-1.1551600	-4.9958770
C	0.0000000	0.0000000	-5.7067740
C	0.3305290	1.1551600	-4.9958770
C	0.3303280	1.1534110	-3.6011380
F	2.7705480	2.7427750	0.0235890
H	-0.5914040	-2.0677870	-5.5258090
H	-0.5942520	-2.0771710	-3.0999770
H	0.5942520	2.0771710	-3.0999770
H	0.5914040	2.0677870	-5.5258090

H	0.0000000	0.0000000	-6.7935170
H	0.6357700	2.0435570	-1.2160270
H	-0.6357700	-2.0435570	-1.2160270
H	0.9163670	-2.7156950	2.6444170
H	0.2769500	-4.8720340	3.6726680
H	-1.8138830	-6.0283430	2.9470220
H	-3.2546410	-4.9682420	1.1863390
H	3.2546410	4.9682420	1.1863390
H	1.8138830	6.0283430	2.9470220
H	-0.2769500	4.8720340	3.6726680
H	-0.9163670	2.7156950	2.6444170

4_a (B3LYP/6-31+G*)

C	1.1986560	1.2153540	0.0360640
C	-0.1892260	1.3972340	0.0549210
P	-1.2073320	0.0024270	0.0000000
C	-0.1945370	-1.3962450	-0.0553380
C	1.1940060	-1.2197630	-0.0366730
C	1.8968410	-0.0035320	-0.0003250
C	3.3842330	-0.0063860	-0.0001980
O	-2.3478810	0.0435950	-1.2072360
C	-3.6813110	0.2722120	-0.7197900
C	-3.6817480	-0.2600900	0.7203830
O	-2.3476750	-0.0346120	1.2074830
C	4.1145760	0.8837060	0.8101410
C	5.5107880	0.8853300	0.8069930
C	6.2166420	-0.0119450	0.0003830
C	5.5076130	-0.9064190	-0.8065060
C	4.1113770	-0.8993080	-0.8102220
C	-0.7913260	-2.7612990	-0.0261060
C	-0.7807550	2.7645560	0.0258230

C	-0.3496240	3.6965650	-0.9373200
C	-0.8684540	4.9921070	-0.9677570
C	-1.8420700	5.3834980	-0.0438270
C	-2.2843000	4.4678700	0.9148370
C	-1.7576920	3.1740220	0.9531960
C	-1.7693520	-3.1673590	-0.9538320
C	-2.3009910	-4.4591450	-0.9151970
C	-1.8627490	-5.3760840	0.0440440
C	-0.8880160	-4.9881310	0.9682440
C	-0.3641740	-3.6946110	0.9375480
H	1.7894670	2.1301170	0.0427520
H	1.7812810	-2.1368000	-0.0433630
H	3.5822850	1.5652490	1.4691750
H	6.0477290	1.5808260	1.4479030
H	7.3036870	-0.0140850	0.0006200
H	6.0420060	-1.6040540	-1.4472140
H	3.5767320	-1.5788090	-1.4694560
H	0.3876790	3.3922400	-1.6757310
H	-0.5191050	5.6930080	-1.7221250
H	-2.2489990	6.3911600	-0.0694020
H	-3.0309130	4.7648370	1.6477680
H	-2.0826330	2.4811060	1.7237270
H	-2.0912090	-2.4735360	-1.7248500
H	-3.0483980	-4.7535260	-1.6483600
H	-2.2736030	-6.3821480	0.0698380
H	-0.5417140	-5.6901050	1.7230170
H	0.3740050	-3.3928220	1.6761220
H	-4.3781770	-0.2640630	-1.3693720
H	-3.8930690	1.3464940	-0.7586330
H	-3.8958560	-1.3339030	0.7591870
H	-4.3772170	0.2777240	1.3701860

4_a (B3LYP/6-31+G*, excited state minima)

C	1.2142770	1.2448260	0.0453230
C	-0.1787560	1.4440930	0.0814750
P	-1.1817040	0.0033190	0.0006610
C	-0.1863830	-1.4426340	-0.0819430
C	1.2076740	-1.2507970	-0.0456960
C	1.8973120	-0.0047970	-0.0000770
C	3.3618750	-0.0086580	0.0001280
O	-2.2944060	0.0491530	-1.2113720
C	-3.6351100	0.2713870	-0.7186330
C	-3.6344860	-0.2555710	0.7246710
O	-2.2923340	-0.0368290	1.2149240
C	4.1069010	1.0732530	0.5395480
C	5.4977400	1.0646650	0.5449350
C	6.2024020	-0.0161570	0.0006230
C	5.4922360	-1.0932560	-0.5439110
C	4.1013660	-1.0945060	-0.5389940
C	-0.7983160	-2.7710930	-0.0452810
C	-0.7837280	2.7756820	0.0440190
C	-0.1349420	3.8311190	-0.6581180
C	-0.6765860	5.1110820	-0.7008960
C	-1.8791060	5.4060890	-0.0418440
C	-2.5266370	4.3838420	0.6693450
C	-1.9955240	3.0986670	0.7147070
C	-2.0115930	-3.0874990	-0.7164470
C	-2.5491490	-4.3700320	-0.6721190
C	-1.9068760	-5.3960650	0.0384050
C	-0.7030140	-5.1075750	0.6978920
C	-0.1549780	-3.8303000	0.6562000
H	1.8157560	2.1460160	0.0505500

H	1.8043520	-2.1551820	-0.0511230
H	3.5871000	1.9076810	0.9978860
H	6.0367690	1.9017310	0.9809680
H	7.2890970	-0.0190300	0.0008200
H	6.0269890	-1.9331760	-0.9797180
H	3.5773250	-1.9262100	-0.9974580
H	0.7773750	3.6221560	-1.2097390
H	-0.1619980	5.8869510	-1.2636660
H	-2.2961490	6.4088240	-0.0730010
H	-3.4443360	4.5989710	1.2128380
H	-2.4829990	2.3477780	1.3275100
H	-2.4952220	-2.3337510	-1.3288090
H	-3.4677950	-4.5801440	-1.2159750
H	-2.3289290	-6.3967270	0.0687010
H	-0.1924110	-5.8864320	1.2601660
H	0.7582830	-3.6263250	1.2081320
H	-4.3200500	-0.2799690	-1.3679400
H	-3.8522710	1.3422060	-0.7693450
H	-3.8542110	-1.3258380	0.7757100
H	-4.3167990	0.2974950	1.3752810

4_b (B3LYP/6-31+G*)

C	-1.2281760	1.1843500	-0.0583950
C	0.1551020	1.4004130	-0.0829090
P	1.2073750	0.0330640	-0.0006050
C	0.2304350	-1.3889980	0.0820130
C	-1.1624090	-1.2487810	0.0570300
C	-1.8953030	-0.0510480	-0.0007750
C	-3.3826620	-0.0911380	-0.0005610
O	2.3462420	0.1178890	1.2059970
C	3.6748080	0.3685310	0.7164850

C	3.6877450	-0.1797980	-0.7177240
O	2.3490310	0.0082880	-1.2074700
C	-4.1365510	0.8289230	0.7521990
C	-5.5323770	0.7953030	0.7492620
C	-6.2139860	-0.1680930	0.0002950
C	-5.4814490	-1.0930190	-0.7493010
C	-4.0859670	-1.0507390	-0.7531290
C	0.8619340	-2.7386670	0.1153080
C	0.7119080	2.7824330	-0.1150310
C	0.2764240	3.7354720	0.8251440
C	0.7596660	5.0448370	0.8000030
C	1.7014570	5.4297560	-0.1589220
C	2.1482790	4.4934430	-1.0951850
C	1.6572680	3.1852860	-1.0773530
C	1.8226590	-3.0902790	1.0824440
C	2.3849260	-4.3694470	1.1013260
C	1.9954890	-5.3273770	0.1612440
C	1.0390840	-4.9934880	-0.8023750
C	0.4845140	-3.7126580	-0.8284450
H	-1.8419270	2.0809290	-0.1304660
H	-1.7264280	-2.1774950	0.1288310
H	-3.6223040	1.5638160	1.3666280
H	-6.0879410	1.5158660	1.3450230
H	-7.3006010	-0.1977940	0.0005760
H	-5.9975870	-1.8425800	-1.3447390
H	-3.5327410	-1.7564440	-1.3678590
H	-0.4361070	3.4367880	1.5897420
H	0.4076240	5.7620420	1.5376130
H	2.0804240	6.4484190	-0.1775420
H	2.8703210	4.7848180	-1.8544790
H	1.9857250	2.4741760	-1.8293600

H	2.1073450	-2.3633370	1.8372190
H	3.1177640	-4.6218760	1.8642140
H	2.4299290	-6.3236440	0.1805570
H	0.7310620	-5.7279140	-1.5427300
H	-0.2395340	-3.4522160	-1.5961850
H	4.3823860	-0.1459120	1.3721430
H	3.8646610	1.4472930	0.7435550
H	3.9275850	-1.2485610	-0.7443580
H	4.3706320	0.3669410	-1.3734040

4_c (B3LYP/6-31+G*)

C	1.2049470	1.2131310	-0.0587360
C	-0.1821920	1.4011410	-0.0523350
P	-1.2083210	0.0134590	-0.0112120
C	-0.2044080	-1.3924750	-0.0594340
C	1.1852390	-1.2230510	-0.0681530
C	1.8958790	-0.0107750	-0.0713660
C	3.3828000	-0.0230460	-0.1089760
O	-2.4253210	-0.0095420	-1.1455870
C	-3.7310920	0.2155530	-0.5830700
C	-3.6255270	-0.2244270	0.8839410
O	-2.2689880	0.0546370	1.2638680
C	4.1380500	0.8790170	0.6643740
C	5.5336210	0.8719240	0.6276310
C	6.2143350	-0.0466530	-0.1767120
C	5.4806820	-0.9531750	-0.9471830
C	4.0847570	-0.9373350	-0.9171730
C	-0.8055860	-2.7531950	0.0299350
C	-0.7677620	2.7721240	-0.0311200
C	-1.6782220	3.2006190	-1.0147660
C	-2.1997620	4.4966830	-0.9921420

C	-1.8174470	5.3940000	0.0089640
C	-0.9095630	4.9831850	0.9894250
C	-0.3957890	3.6849790	0.9725100
C	-1.8055100	-3.1859900	-0.8618260
C	-2.3398780	-4.4737920	-0.7690730
C	-1.8834050	-5.3604590	0.2099790
C	-0.8873980	-4.9459650	1.0992430
C	-0.3602760	-3.6561330	1.0142000
H	1.7992400	2.1246370	-0.1018050
H	1.7672680	-2.1435210	-0.0655780
H	3.6257910	1.5784470	1.3205960
H	6.0901830	1.5776100	1.2400400
H	7.3010360	-0.0554080	-0.2030780
H	5.9952720	-1.6671280	-1.5860990
H	3.5301600	-1.6264440	-1.5492610
H	-1.9525840	2.5201700	-1.8161750
H	-2.8938080	4.8103230	-1.7684700
H	-2.2197250	6.4037660	0.0230630
H	-0.6073240	5.6711660	1.7753630
H	0.2915520	3.3639350	1.7510100
H	-2.1435850	-2.5162000	-1.6468460
H	-3.1044660	-4.7890590	-1.4753410
H	-2.2966990	-6.3635540	0.2779300
H	-0.5265460	-5.6239770	1.8689590
H	0.3950200	-3.3333390	1.7261010
H	-4.4526390	-0.3754020	-1.1532080
H	-3.9747260	1.2802360	-0.6701150
H	-3.8154860	-1.2971170	1.0027460
H	-4.2876080	0.3429010	1.5432690

4_d (B3LYP/6-31+G*)

C	1.1216880	-1.2784720	-0.0881590
C	-0.2748510	-1.3762870	-0.0873050
P	-1.2076610	0.0768810	-0.0130680
C	-0.1129180	1.4117690	-0.0234970
C	1.2629040	1.1527680	-0.0351930
C	1.8918970	-0.1028710	-0.0694810
C	3.3767190	-0.1871330	-0.1082430
O	-2.2680170	0.1548260	1.2605770
C	-3.6328720	-0.0712690	0.8756450
C	-3.7201090	0.3879560	-0.5866430
O	-2.4216980	0.1252320	-1.1496470
C	4.0713950	-1.1528290	0.6445870
C	5.4644470	-1.2363420	0.6057920
C	6.2036520	-0.3475710	-0.1801880
C	5.5308030	0.6212830	-0.9300050
C	4.1368730	0.6962180	-0.8980370
C	-0.6263560	2.8085080	0.0605020
C	-0.9446280	-2.7077870	-0.0588530
C	-0.5772580	-3.6564690	0.9143000
C	-1.1658060	-4.9220300	0.9447740
C	-2.1468710	-5.2649020	0.0096640
C	-2.5265950	-4.3319930	-0.9592100
C	-1.9307020	-3.0686200	-0.9967490
C	-0.1829800	3.6665530	1.0834290
C	-0.6295120	4.9873950	1.1571070
C	-1.5401800	5.4770250	0.2161780
C	-1.9926220	4.6349730	-0.8034960
C	-1.5381760	3.3161200	-0.8834140
H	1.6557630	-2.2254800	-0.1500480
H	1.9033530	2.0332890	-0.0116310
H	3.5145490	-1.8308740	1.2866820

H	5.9737700	-1.9896360	1.2024180
H	7.2885440	-0.4094830	-0.2081320
H	6.0909850	1.3134580	-1.5544060
H	3.6284110	1.4335760	-1.5143140
H	0.1667280	-3.3877800	1.6598030
H	-0.8646650	-5.6369450	1.7067390
H	-2.6078780	-6.2490410	0.0343410
H	-3.2786720	-4.5923970	-1.7003670
H	-2.2094830	-2.3622470	-1.7728530
H	0.5068500	3.2855730	1.8321650
H	-0.2725150	5.6314980	1.9571320
H	-1.8904980	6.5043660	0.2750690
H	-2.6893740	5.0092940	-1.5499810
H	-1.8658890	2.6797730	-1.7010780
H	-4.2753010	0.5121510	1.5403040
H	-3.8616520	-1.1376860	0.9823120
H	-3.9278850	1.4610520	-0.6623470
H	-4.4601490	-0.1720830	-1.1642470

4_e (B3LYP/6-31+G*)

C	1.1986680	1.2160080	-0.0010550
C	-0.1892610	1.3949700	-0.0164320
P	-1.2098240	0.0014470	0.0000000
C	-0.1926070	-1.3945310	0.0164340
C	1.1957470	-1.2188970	0.0008060
C	1.8989180	-0.0022880	-0.0001900
C	3.3863790	-0.0040990	-0.0002040
O	-2.3508600	-0.0824380	-1.2048070
C	-3.6883700	0.1778610	-0.7484680
C	-3.6889090	-0.1690920	0.7480380
O	-2.3508450	0.0881550	1.2045300

C	4.1162070	0.9058380	-0.7884990
C	5.5124560	0.9084600	-0.7848340
C	6.2186930	-0.0075340	0.0000000
C	5.5100630	-0.9218660	0.7848430
C	4.1139140	-0.9158620	0.7882760
C	-0.7879480	-2.7612930	-0.0059840
C	-0.7813080	2.7631680	0.0062020
C	-1.6702090	3.1960030	-0.9947730
C	-2.2000930	4.4887820	-0.9723910
C	-1.8465730	5.3782840	0.0460660
C	-0.9586980	4.9633610	1.0431100
C	-0.4369360	3.6683020	1.0263430
C	-1.6774140	-3.1920130	0.9953810
C	-2.2106100	-4.4834290	0.9730610
C	-1.8598780	-5.3736850	-0.0457090
C	-0.9714770	-4.9608670	-1.0431500
C	-0.4464460	-3.6671330	-1.0264680
H	1.7869350	2.1324250	0.0164810
H	1.7818190	-2.1367170	-0.0166510
H	3.5835010	1.6028920	-1.4307170
H	6.0491270	1.6196750	-1.4085180
H	7.3057060	-0.0089280	0.0001720
H	6.0450430	-1.6343090	1.4085800
H	3.5793010	-1.6115530	1.4303950
H	-1.9190930	2.5228420	-1.8108640
H	-2.8774630	4.8060850	-1.7618620
H	-2.2551330	6.3855190	0.0605930
H	-0.6780580	5.6458250	1.8417500
H	0.2357980	3.3441390	1.8162150
H	-1.9241820	-2.5183190	1.8116810
H	-2.8883950	-4.7991150	1.7628250

H	-2.2710230	-6.3798690	-0.0601920
H	-0.6930300	-5.6439010	-1.8420690
H	0.2266080	-3.3445420	-1.8167170
H	-4.3719680	-0.4484280	-1.3274470
H	-3.9230700	1.2346070	-0.9179430
H	-3.9262000	-1.2252630	0.9174770
H	-4.3710770	0.4588260	1.3269300

4_f (B3LYP/6-31+G*)

C	-1.2017200	1.2153280	0.0206990
C	0.1862690	1.3944500	0.0419790
P	1.2072490	0.0024790	0.0000000
C	0.1919950	-1.3936840	-0.0423900
C	-1.1967170	-1.2202320	-0.0210290
C	-1.9007470	-0.0038850	-0.0001390
C	-3.3881720	-0.0069940	0.0000000
O	2.3485640	-0.0906920	1.2042250
C	3.6847550	0.1792030	0.7489310
C	3.6857760	-0.1644170	-0.7478140
O	2.3487550	0.1004250	-1.2036730
C	-4.1191340	0.9121520	-0.7763330
C	-5.5152880	0.9136050	-0.7729090
C	-6.2207140	-0.0128840	0.0003110
C	-5.5112840	-0.9364510	0.7733460
C	-4.1151280	-0.9291980	0.7764470
C	0.7908880	-2.7589850	-0.0832600
C	0.7795520	2.7622050	0.0827700
C	1.6365260	3.1611020	1.1248330
C	2.1674470	4.4531470	1.1607020
C	1.8472830	5.3751830	0.1603390
C	0.9920650	4.9937700	-0.8777640

C	0.4691330	3.6996210	-0.9186640
C	1.6492570	-3.1543730	-1.1254970
C	2.1857620	-4.4441090	-1.1612470
C	1.8698930	-5.3673180	-0.1605890
C	1.0133980	-4.9893810	0.8777270
C	0.4849250	-3.6974900	0.9185330
H	-1.7908570	2.1300800	0.0679370
H	-1.7821530	-2.1373470	-0.0683380
H	-3.5869930	1.6179390	-1.4093880
H	-6.0526880	1.6322740	-1.3873210
H	-7.3077630	-0.0151670	0.0004260
H	-6.0455150	-1.6573690	1.3878810
H	-3.5799350	-1.6328080	1.4093690
H	1.8608100	2.4604600	1.9246310
H	2.8194790	4.7440570	1.9810520
H	2.2564570	6.3818070	0.1912660
H	0.7377920	5.7018700	-1.6628140
H	-0.1778340	3.4013060	-1.7396660
H	1.8703770	-2.4528660	-1.9254240
H	2.8388570	-4.7323390	-1.9816980
H	2.2834580	-6.3721490	-0.1914010
H	0.7625180	-5.6983770	1.6630600
H	-0.1629450	-3.4017650	1.7397630
H	4.3722920	-0.4442040	1.3263500
H	3.9124200	1.2372020	0.9206340
H	3.9176900	-1.2215030	-0.9194380
H	4.3711200	0.4616710	-1.3249310

4_ω_TS_a (B3LYP/6-31+G*)

C	-0.5365840	-1.6334310	-0.0145450
C	0.7781370	-1.1647520	-0.0027600

P	1.0684140	0.5346640	-0.0168080
C	-0.4620390	1.3347990	-0.0522380
C	-1.6228310	0.5480380	-0.0494720
C	-1.7081950	-0.8531980	-0.0389170
C	-3.0394340	-1.5162340	-0.0629350
O	2.1190640	1.0401900	-1.2032580
C	3.4301990	1.3437210	-0.6946160
C	3.2093580	1.7722490	0.7631830
O	2.0737130	1.0149580	1.2121690
C	-3.2981080	-2.6599740	0.7164100
C	-4.5455960	-3.2863300	0.6909390
C	-5.5747550	-2.7790400	-0.1075310
C	-5.3374600	-1.6409810	-0.8835930
C	-4.0857190	-1.0225650	-0.8654120
C	-0.5459470	2.8200300	0.0178190
C	1.9443050	-2.1045030	0.0045750
C	2.5287940	-2.5317440	-1.1998430
C	3.6067900	-3.4215300	-1.1953360
C	4.1209320	-3.8953380	0.0151690
C	3.5512330	-3.4742020	1.2203190
C	2.4742900	-2.5835150	1.2145070
C	0.1873460	3.6490940	-0.8526230
C	0.0780540	5.0401160	-0.7796200
C	-0.7704210	5.6350220	0.1579190
C	-1.5074840	4.8241550	1.0263620
C	-1.3910070	3.4347500	0.9615970
H	-0.6504880	-2.7163980	-0.0322440
H	-2.5597960	1.1030180	-0.0483220
H	-2.5200230	-3.0486710	1.3687940
H	-4.7170960	-4.1654870	1.3077910
H	-6.5474220	-3.2640490	-0.1252070

H	-6.1246230	-1.2399250	-1.5179020
H	-3.9083050	-0.1592860	-1.5020270
H	2.1276750	-2.1649400	-2.1411520
H	4.0418910	-3.7463540	-2.1374340
H	4.9582780	-4.5887510	0.0192680
H	3.9444310	-3.8387040	2.1663040
H	2.0347950	-2.2523470	2.1517740
H	0.8238860	3.1958280	-1.6065790
H	0.6469650	5.6595370	-1.4691980
H	-0.8581340	6.7172330	0.2104470
H	-2.1670110	5.2737890	1.7647850
H	-1.9470780	2.8135690	1.6589210
H	3.8588340	2.1381940	-1.3111620
H	4.0545270	0.4458490	-0.7647790
H	2.9789260	2.8405170	0.8450750
H	4.0581820	1.5284040	1.4075340

4_ω_TS_b (B3LYP/6-31+G*)

C	1.6741180	0.3190160	-0.0459980
C	0.6584120	1.2958050	-0.0247380
P	-0.9745900	0.7065810	-0.0169240
C	-0.9368410	-1.0249560	-0.0406700
C	0.2886140	-1.6847020	-0.0315790
C	1.5570740	-1.0753720	-0.0414660
C	2.7816640	-1.9205870	-0.0580850
O	-1.9948230	1.2934480	-1.1911150
C	-3.1442590	1.9910060	-0.6878190
C	-3.2256490	1.6583810	0.8169530
O	-1.8881230	1.3265040	1.2198250
C	3.9030190	-1.5952450	0.7283920
C	5.0538680	-2.3856420	0.7095900

C	5.1101920	-3.5316960	-0.0886380
C	4.0030490	-3.8738530	-0.8702720
C	2.8570460	-3.0763050	-0.8582250
C	-2.2180260	-1.7885750	0.0209500
C	0.9842570	2.7509690	-0.0151740
C	-0.0156680	3.7441490	-0.0577390
C	0.2895330	5.1053180	-0.0554360
C	1.6163620	5.5348710	-0.0084250
C	2.6268400	4.5717000	0.0408060
C	2.3182660	3.2119240	0.0395960
C	-3.1919230	-1.6843550	-0.9897860
C	-4.3720580	-2.4300290	-0.9256360
C	-4.6018340	-3.2979480	0.1457230
C	-3.6404460	-3.4150380	1.1539160
C	-2.4642630	-2.6641950	1.0944610
H	2.6904150	0.6947270	-0.0940310
H	0.2466280	-2.7726850	-0.0131620
H	3.8623310	-0.7279100	1.3829480
H	5.9026440	-2.1126910	1.3323500
H	6.0039960	-4.1502120	-0.1005930
H	4.0350270	-4.7576910	-1.5031780
H	2.0178450	-3.3389770	-1.4977090
H	-1.0588020	3.4612740	-0.0885270
H	-0.5195330	5.8312170	-0.0901400
H	1.8581400	6.5942710	-0.0068010
H	3.6697670	4.8766580	0.0830510
H	3.1439410	2.5114390	0.0861270
H	-3.0063840	-1.0345690	-1.8400900
H	-5.1062720	-2.3432440	-1.7232190
H	-5.5182420	-3.8807820	0.1921570
H	-3.8082330	-4.0860830	1.9928200

H	-1.7281320	-2.7451200	1.8901810
H	-4.0239350	1.6422260	-1.2357660
H	-3.0112800	3.0630560	-0.8707820
H	-3.8787130	0.7997470	1.0060540
H	-3.5568750	2.5091920	1.4182890

4_θ_TS_a (B3LYP/6-31+G*)

C	-1.1891000	-1.2265870	0.0503010
C	0.2029290	-1.3951090	0.0729240
P	1.2058320	0.0092420	-0.0003140
C	0.1825310	1.3988040	-0.0725610
C	-1.2069770	1.2094200	-0.0500210
C	-1.8904090	-0.0136260	0.0000000
C	-3.3896490	-0.0246070	-0.0001040
O	2.3482100	-0.0342430	-1.2076770
C	3.6827480	-0.2497570	-0.7190990
C	3.6808940	0.2952800	0.7164580
O	2.3484880	0.0685890	1.2059150
C	-4.1107520	0.0483560	1.2028330
C	-5.5086330	0.0384320	1.2046520
C	-6.2114040	-0.0450750	-0.0005700
C	-5.5071280	-0.1184790	-1.2055130
C	-4.1092640	-0.1080480	-1.2032590
C	0.7659990	2.7695620	-0.0777950
C	0.8067240	-2.7571330	0.0786860
C	0.3854940	-3.7155830	-0.8627970
C	0.9152660	-5.0070080	-0.8622740
C	1.8903180	-5.3685260	0.0722560
C	2.3224580	-4.4270330	1.0102280
C	1.7846820	-3.1372580	1.0176350
C	1.7412340	3.1632950	-1.0139710

C	2.2596010	4.4609750	-1.0063700
C	1.8104660	5.3969860	-0.0709010
C	0.8380860	5.0218330	0.8610340
C	0.3277180	3.7226360	0.8613850
H	-1.7779300	-2.1422830	0.0931380
H	-1.8094010	2.1162250	-0.0928910
H	-3.5673730	0.1111660	2.1428200
H	-6.0478080	0.0955450	2.1473500
H	-7.2985910	-0.0531920	-0.0006330
H	-6.0450960	-0.1831610	-2.1484100
H	-3.5647340	-0.1627710	-2.1430880
H	-0.3534460	-3.4349570	-1.6089050
H	0.5731450	-5.7283690	-1.6005350
H	2.3057970	-6.3730370	0.0710170
H	3.0697880	-4.7005640	1.7515340
H	2.1018080	-2.4241510	1.7727350
H	2.0714600	2.4541430	-1.7671480
H	3.0051130	4.7447820	-1.7456450
H	2.2108170	6.4076170	-0.0695530
H	0.4829240	5.7387130	1.5975040
H	-0.4090620	3.4319410	1.6057820
H	4.3763780	0.2849370	-1.3735600
H	3.9017100	-1.3229800	-0.7482390
H	3.8910870	1.3702590	0.7457520
H	4.3793700	-0.2337670	1.3703720

4_θ_TS_b (B3LYP/6-31+G*)

C	-1.3315900	1.0480480	-0.0447170
C	0.0204570	1.4084900	-0.0649230
P	1.2147720	0.1651070	-0.0054670
C	0.3837350	-1.3458170	0.0662990

C	-1.0141430	-1.3507410	0.0465190
C	-1.8854220	-0.2453620	-0.0003650
C	-3.3684400	-0.4432560	-0.0020480
O	2.3461000	0.3535290	1.1941820
C	3.6470190	0.7150750	0.6976050
C	3.6942870	0.1765080	-0.7390140
O	2.3420660	0.2678800	-1.2202580
C	-4.2677490	0.6445620	-0.0263590
C	-5.6509160	0.4613470	-0.0279800
C	-6.2005770	-0.8219800	-0.0052240
C	-5.3332960	-1.9159070	0.0188890
C	-3.9506430	-1.7291450	0.0202480
C	1.1485050	-2.6258810	0.0717150
C	0.4240620	2.8436330	-0.0616480
C	-0.1051050	3.7193960	0.9051250
C	0.2330150	5.0738600	0.9111880
C	1.1199970	5.5827950	-0.0421610
C	1.6593110	4.7242170	-1.0039420
C	1.3138040	3.3702190	-1.0169950
C	2.1260200	-2.9044790	1.0456650
C	2.8159430	-4.1197300	1.0403170
C	2.5404960	-5.0849680	0.0679350
C	1.5690020	-4.8233810	-0.9028950
C	0.8864290	-3.6053710	-0.9045540
H	-2.0093260	1.8953470	-0.0839440
H	-1.4479020	-2.3450730	0.0896700
H	-3.8993950	1.6643660	-0.0448390
H	-6.3004310	1.3335010	-0.0472480
H	-7.2777650	-0.9658500	-0.0064040
H	-5.7306640	-2.9281520	0.0367540
H	-3.3275690	-2.6165270	0.0391910

H	-0.7750580	3.3260810	1.6655210
H	-0.1892660	5.7296160	1.6688350
H	1.3860420	6.6367470	-0.0363650
H	2.3401960	5.1111110	-1.7585800
H	1.7130010	2.7183530	-1.7884820
H	2.3240980	-2.1738230	1.8244540
H	3.5596220	-4.3177970	1.8087320
H	3.0747750	-6.0316410	0.0677790
H	1.3485910	-5.5640680	-1.6678410
H	0.1505470	-3.3981250	-1.6773570
H	4.3984030	0.2569370	1.3461740
H	3.7476920	1.8056470	0.7294850
H	4.0112280	-0.8718070	-0.7708380
H	4.3310740	0.7744970	-1.3961670

5_a (B3LYP/6-31+G*)

C	-1.2498950	1.2105730	-0.0434710
C	0.1390820	1.4358590	-0.0225040
P	1.2051190	0.0631070	-0.0657080
C	0.2051660	-1.3597530	-0.0410980
C	-1.1796230	-1.2231430	-0.0338520
C	-1.9141790	-0.0179900	-0.0530860
C	-3.4005720	-0.0615010	-0.0907730
O	2.3306760	0.0833120	1.1348200
C	3.6757880	-0.1008900	0.6608540
C	3.6550290	0.2360130	-0.8424160
O	2.3137070	-0.0385040	-1.2839010
C	-4.1760950	0.8191860	0.6871410
C	-5.5711930	0.7836280	0.6464560
C	-6.2303880	-0.1435440	-0.1658880
C	-5.4762400	-1.0307620	-0.9390940

C	-4.0810290	-0.9866620	-0.9048020
C	0.8326430	-2.7083600	0.0666770
C	0.7160860	2.7912160	0.0276040
C	-0.0489100	3.9428850	0.3147710
C	0.5668040	5.1889800	0.3278790
C	1.9373940	5.2806480	0.0702720
C	2.6230000	4.0928790	-0.1813680
N	2.0447240	2.8880480	-0.2059820
C	0.4377120	-3.5819170	1.0976350
C	0.9823760	-4.8628910	1.2041730
C	1.9455770	-5.2968260	0.2886600
C	2.3521620	-4.4387170	-0.7367800
C	1.8012080	-3.1595500	-0.8497550
H	-1.8782530	2.0961370	-0.0888830
H	-1.7467720	-2.1524570	-0.0050000
H	-3.6795380	1.5213700	1.3526300
H	-6.1441210	1.4722610	1.2632160
H	-7.3165430	-0.1751290	-0.1947960
H	-5.9744800	-1.7521070	-1.5826240
H	-3.5101280	-1.6619940	-1.5372820
H	-1.1068470	3.8664700	0.5392540
H	-0.0166670	6.0800960	0.5463470
H	2.4579010	6.2335540	0.0724310
H	3.6950470	4.1082580	-0.3765740
H	-0.2922210	-3.2427650	1.8282040
H	0.6609190	-5.5181880	2.0101710
H	2.3726630	-6.2929570	0.3725140
H	3.0914520	-4.7696740	-1.4625420
H	2.1013030	-2.5109590	-1.6667710
H	4.3269370	0.5697650	1.2276460
H	3.9645980	-1.1416070	0.8441450

H	3.8669940	1.2911720	-1.0243690
H	4.3314120	-0.3987940	-1.4209930

5_a (B3LYP/6-31+G*, excited state minima)

C	0.9157060	-1.4908230	0.0528420
C	-0.4743520	-1.3832680	0.0037550
P	-1.1530520	0.2572500	-0.0333250
C	0.1636780	1.4143540	-0.0947730
C	1.4897930	0.9273440	-0.1105870
C	1.8774120	-0.4239920	-0.0342300
C	3.3018250	-0.7656330	-0.0463830
O	-2.1437300	0.6349540	1.2041770
C	-3.5389430	0.6385220	0.8375510
C	-3.6120210	0.5971170	-0.7132510
O	-2.2619190	0.4695150	-1.2136790
C	3.7868070	-1.9447350	0.5768120
C	5.1432660	-2.2563190	0.5808850
C	6.0657540	-1.4147060	-0.0507210
C	5.6107170	-0.2500790	-0.6832460
C	4.2580070	0.0709800	-0.6783760
C	-0.1169920	2.8522020	-0.0146950
C	-1.3931130	-2.5098720	-0.0111490
C	-0.9637730	-3.8581670	-0.1461920
C	-1.8960440	-4.8811560	-0.1222150
C	-3.2632530	-4.5683050	0.0321250
C	-3.6033120	-3.2145550	0.1367710
N	-2.7290680	-2.2107880	0.1183320
C	0.7733400	3.7123240	0.6782440
C	0.5429110	5.0826380	0.7555170
C	-0.5813250	5.6520220	0.1444570
C	-1.4716520	4.8215810	-0.5467600

C	-1.2465980	3.4496710	-0.6281290
H	1.3155320	-2.4970020	0.0950720
H	2.2704490	1.6781820	-0.1549100
H	3.0973490	-2.5974900	1.1019710
H	5.4841880	-3.1578550	1.0833800
H	7.1235660	-1.6634940	-0.0538990
H	6.3151680	0.4041480	-1.1903980
H	3.9254170	0.9596770	-1.2048130
H	0.0862820	-4.0935940	-0.2884400
H	-1.5734920	-5.9138810	-0.2308160
H	-4.0285400	-5.3374410	0.0608940
H	-4.6511510	-2.9278010	0.2433280
H	1.6294500	3.2884390	1.1949110
H	1.2385860	5.7103960	1.3075080
H	-0.7580670	6.7226390	0.2032690
H	-2.3398750	5.2496050	-1.0426530
H	-1.9195560	2.8363600	-1.2170510
H	-4.0104080	-0.2388530	1.2805250
H	-3.9656120	1.5573680	1.2485730
H	-4.1825290	-0.2629100	-1.0648130
H	-4.0193380	1.5216390	-1.1335780

5_b (B3LYP/6-31+G*)

C	1.2244850	-1.2308320	-0.0137560
C	-0.1681750	-1.4240590	-0.0137700
P	-1.2037100	-0.0287570	0.0509320
C	-0.1761300	1.3688230	-0.0509530
C	1.2059680	1.2031320	-0.0517330
C	1.9154640	-0.0163010	-0.0220770
C	3.4028180	-0.0072150	-0.0112650
O	-2.2274090	0.1439550	1.3295570

C	-3.5937500	-0.1555730	1.0047340
C	-3.7251780	0.0707770	-0.5159410
O	-2.4069960	-0.0806240	-1.0729660
C	4.1298990	-0.8943620	0.8050430
C	5.5262500	-0.8938310	0.8098870
C	6.2349470	0.0033490	0.0058650
C	5.5288650	0.8961640	-0.8056700
C	4.1327990	0.8872160	-0.8170310
C	-0.7782680	2.7327800	-0.0428670
C	-0.7743130	-2.7664210	-0.0657190
C	-0.0544170	-3.9165840	-0.4561210
C	-0.6903160	-5.1526770	-0.4625060
C	-2.0369780	-5.2334990	-0.0971320
C	-2.6807160	-4.0459240	0.2487870
N	-2.0819530	-2.8507390	0.2692940
C	-0.3803540	3.6764070	0.9217320
C	-0.9078840	4.9691390	0.9224560
C	-1.8552420	5.3438280	-0.0347920
C	-2.2639810	4.4152390	-0.9961770
C	-1.7295880	3.1243450	-1.0029970
H	1.8332110	-2.1314380	-0.0207780
H	1.7914640	2.1212730	-0.0675600
H	3.5943020	-1.5736490	1.4639280
H	6.0608230	-1.5864870	1.4558850
H	7.3218970	0.0079220	0.0126500
H	6.0659600	1.5943230	-1.4435310
H	3.6008740	1.5663530	-1.4788670
H	0.9808280	-3.8429810	-0.7704490
H	-0.1428670	-6.0433560	-0.7612340
H	-2.5723660	-6.1781270	-0.0904230
H	-3.7340010	-4.0532030	0.5282110

H	0.3374850	3.3840940	1.6838160
H	-0.5854250	5.6808900	1.6786320
H	-2.2685650	6.3492460	-0.0327680
H	-2.9904850	4.6997830	-1.7537950
H	-2.0285930	2.4186320	-1.7729520
H	-4.2272220	0.5238400	1.5810040
H	-3.7946990	-1.1934710	1.2770720
H	-4.0792960	1.0808980	-0.7482910
H	-4.3760010	-0.6681840	-0.9902800

5_b (B3LYP/6-31+G*, excited state minima)

C	0.9158320	-1.4907230	0.0529540
C	-0.4742330	-1.3833050	0.0037650
P	-1.1530840	0.2571640	-0.0331970
C	0.1635470	1.4143860	-0.0947930
C	1.4896980	0.9274830	-0.1105810
C	1.8774290	-0.4238210	-0.0341390
C	3.3018780	-0.7653340	-0.0462580
O	-2.1436170	0.6347260	1.2044440
C	-3.5388580	0.6381440	0.8380510
C	-3.6121600	0.5969500	-0.7127640
O	-2.2621380	0.4692450	-1.2133820
C	3.7869790	-1.9443070	0.5770920
C	5.1434600	-2.2557750	0.5811700
C	6.0658630	-1.4141700	-0.0505820
C	5.6107110	-0.2496690	-0.6832460
C	4.2579710	0.0712770	-0.6783780
C	-0.1172760	2.8522000	-0.0148510
C	-1.3928760	-2.5099880	-0.0113310
C	-0.9634230	-3.8581720	-0.1471330
C	-1.8955500	-4.8812950	-0.1231210

C	-3.2627210	-4.5686930	0.0319730
C	-3.6029280	-3.2150120	0.1371580
N	-2.7288340	-2.2111260	0.1187000
C	0.7729350	3.7124650	0.6780820
C	0.5423730	5.0827580	0.7552540
C	-0.5818790	5.6519960	0.1440850
C	-1.4720840	4.8214220	-0.5471310
C	-1.2468980	3.4495290	-0.6284010
H	1.3157410	-2.4968640	0.0953660
H	2.2702980	1.6783770	-0.1549720
H	3.0975880	-2.5970420	1.1023650
H	5.4844770	-3.1572110	1.0837800
H	7.1236940	-1.6628770	-0.0537560
H	6.3150900	0.4045530	-1.1905050
H	3.9252930	0.9598740	-1.2049310
H	0.0865860	-4.0933840	-0.2900910
H	-1.5729120	-5.9139290	-0.2323440
H	-4.0278890	-5.3379410	0.0608620
H	-4.6507720	-2.9284430	0.2441700
H	1.6290460	3.2886870	1.1948370
H	1.2379550	5.7106200	1.3072430
H	-0.7587320	6.7225990	0.2028190
H	-2.3403090	5.2493330	-1.0431170
H	-1.9197430	2.8361250	-1.2173590
H	-3.9656280	1.5568610	1.2492550
H	-4.0101340	-0.2393700	1.2809570
H	-4.0194450	1.5215740	-1.1328990
H	-4.1828090	-0.2629610	-1.0643840

5_c (B3LYP/6-31+G*)

C	1.2255050	-1.2298080	-0.0138140

C	-0.1670170	-1.4242490	-0.0138430
P	-1.2037070	-0.0298440	0.0509620
C	-0.1772580	1.3686350	-0.0508590
C	1.2049440	1.2041340	-0.0515630
C	1.9154710	-0.0147330	-0.0219920
C	3.4028150	-0.0043830	-0.0111780
O	-2.2275040	0.1423850	1.3297160
C	-3.5935550	-0.1585610	1.0048250
C	-3.7251970	0.0689040	-0.5155750
O	-2.4070550	-0.0826710	-1.0728020
C	4.1306090	-0.8910660	0.8050250
C	5.5269450	-0.8894310	0.8098430
C	6.2349050	0.0084700	0.0059550
C	5.5281240	0.9008310	-0.8054440
C	4.1320530	0.8907130	-0.8168450
C	-0.7805640	2.7320660	-0.0429040
C	-0.7719560	-2.7671370	-0.0658750
C	-0.0511360	-3.9166140	-0.4566040
C	-0.6859390	-5.1532730	-0.4629680
C	-2.0324290	-5.2353410	-0.0972360
C	-2.6771240	-4.0483880	0.2490280
N	-2.0794150	-2.8526760	0.2695190
C	-0.3837750	3.6760180	0.9218470
C	-0.9124280	4.9682910	0.9224330
C	-1.8598400	5.3421660	-0.0350830
C	-2.2675080	4.4132240	-0.9965800
C	-1.7319700	3.1228000	-1.0032670
H	1.8349680	-2.1299150	-0.0209610
H	1.7896830	2.1227600	-0.0673140
H	3.5955260	-1.5708450	1.4638180
H	6.0621000	-1.5817910	1.4556690

H	7.3218620	0.0139000	0.0127390
H	6.0646190	1.5995810	-1.4431540
H	3.5995890	1.5694860	-1.4786210
H	0.9839550	-3.8420560	-0.7712140
H	-0.1377810	-6.0434270	-0.7619630
H	-2.5669680	-6.1804500	-0.0905250
H	-3.7303170	-4.0566310	0.5287670
H	0.3341010	3.3843170	1.6841290
H	-0.5907960	5.6803080	1.6787110
H	-2.2740570	6.3472170	-0.0331590
H	-2.9940740	4.6971180	-1.7543830
H	-2.0301760	2.4168220	-1.7732960
H	-4.2277890	0.5196550	1.5816650
H	-3.7932680	-1.1969130	1.2763330
H	-4.0788800	1.0793550	-0.7471500
H	-4.3763530	-0.6694230	-0.9904380

5_d (B3LYP/6-31+G*)

C	-1.2679210	1.1881640	0.0162520
C	0.1165180	1.4354380	-0.0121500
P	1.2052790	0.0831180	0.0681660
C	0.2301990	-1.3562630	0.0637160
C	-1.1572510	-1.2438510	0.0466300
C	-1.9118410	-0.0509530	0.0490360
C	-3.3973630	-0.1149890	0.0947550
O	2.3076520	0.0178600	1.2946590
C	3.6456030	0.3160420	0.8581890
C	3.6833440	-0.0460800	-0.6383220
O	2.3392430	0.1121250	-1.1247470
C	-4.1393170	0.7625640	0.9079180
C	-5.5342820	0.7104180	0.9442500

C	-6.2260680	-0.2297330	0.1754730
C	-5.5048790	-1.1135380	-0.6326350
C	-4.1107580	-1.0530750	-0.6751730
C	0.8812610	-2.6976900	0.0153820
C	0.6688220	2.7974230	-0.1261130
C	-0.1078390	3.9089340	-0.5198890
C	0.4798440	5.1673100	-0.5815940
C	1.8346160	5.3082380	-0.2685510
C	2.5352990	4.1550130	0.0829450
N	1.9835390	2.9396930	0.1568510
C	1.8149860	-3.1143680	0.9827880
C	2.3860710	-4.3882920	0.9232220
C	2.0349490	-5.2746460	-0.0987320
C	1.1071240	-4.8749770	-1.0649100
C	0.5421250	-3.5994170	-1.0109030
H	-1.9112700	2.0641330	-0.0021930
H	-1.7076010	-2.1830260	0.0768930
H	-3.6151990	1.4749670	1.5404550
H	-6.0803890	1.3966710	1.5873920
H	-7.3116820	-0.2745510	0.2070440
H	-6.0290500	-1.8452870	-1.2430000
H	-3.5681660	-1.7272900	-1.3333860
H	-1.1502050	3.7890460	-0.7935750
H	-0.1117880	6.0284820	-0.8826160
H	2.3334170	6.2719920	-0.3058740
H	3.5972520	4.2088080	0.3215610
H	2.0719260	-2.4430930	1.7961170
H	3.0971340	-4.6930830	1.6875800
H	2.4774960	-6.2666810	-0.1407310
H	0.8291270	-5.5529830	-1.8682870
H	-0.1606700	-3.2860690	-1.7786920

H	4.3322950	-0.2929220	1.4521170
H	3.8317580	1.3790250	1.0229510
H	3.9873760	-1.0859350	-0.8009130
H	4.3296710	0.6232740	-1.2120920

5_e (B3LYP/6-31+G*)

C	1.2404540	1.2190660	-0.0116360
C	-0.1506960	1.4294480	-0.0196430
P	-1.2016920	0.0443290	-0.0481170
C	-0.1884260	-1.3627330	0.0689920
C	1.1951480	-1.2145310	0.0607810
C	1.9176990	-0.0023470	0.0179070
C	3.4050140	-0.0294540	0.0155750
O	-2.2386920	-0.1362450	-1.3148900
C	-3.6014560	0.1666220	-0.9783800
C	-3.7166980	-0.0469470	0.5451550
O	-2.3936700	0.1168180	1.0869470
C	4.1527250	0.8735800	0.7948590
C	5.5487740	0.8541300	0.7855640
C	6.2366030	-0.0785340	0.0039410
C	5.5102600	-0.9880120	-0.7700980
C	4.1143100	-0.9600540	-0.7668880
C	-0.8086560	-2.7181110	0.1207820
C	-0.7424740	2.7789680	-0.0325060
C	-0.0013750	3.9458150	0.2556860
C	-0.6291250	5.1850660	0.2097280
C	-1.9884140	5.2549450	-0.1080170
C	-2.6513500	4.0537410	-0.3566260
N	-2.0610160	2.8548190	-0.3244510
C	-0.4618800	-3.6914560	-0.8336040
C	-1.0049180	-4.9767150	-0.7792320

C	-1.9168250	-5.3136480	0.2253230
C	-2.2751900	-4.3549740	1.1771740
C	-1.7255930	-3.0714140	1.1279630
H	1.8599760	2.1104450	-0.0669320
H	1.7706400	-2.1362970	0.1335480
H	3.6331640	1.5804830	1.4372870
H	6.0997920	1.5598590	1.4029250
H	7.3234580	-0.0976320	-0.0003770
H	6.0311320	-1.7144240	-1.3895460
H	3.5654030	-1.6536410	-1.3991690
H	1.0466110	3.8858280	0.5272980
H	-0.0643780	6.0879590	0.4290370
H	-2.5177170	6.2019130	-0.1534090
H	-3.7140200	4.0524820	-0.5981100
H	0.2285210	-3.4281550	-1.6308180
H	-0.7220040	-5.7123790	-1.5283260
H	-2.3415900	-6.3134220	0.2670870
H	-2.9737470	-4.6100940	1.9706910
H	-1.9860070	-2.3409800	1.8886800
H	-4.2412470	-0.5174220	-1.5420880
H	-3.8047170	1.2021900	-1.2575400
H	-4.0625020	-1.0571330	0.7896590
H	-4.3668420	0.6924650	1.0197560

5_f (B3LYP/6-31+G*)

C	-1.1641170	-1.2982800	-0.0088100
C	0.2373670	-1.4152580	-0.0214980
P	1.1963990	0.0368740	-0.0127020
C	0.0914420	1.3751370	0.0710430
C	-1.2793170	1.1335820	0.0609060
C	-1.9197480	-0.1238900	0.0261930

C	-3.4057130	-0.1954740	0.0375770
O	2.2522740	0.2651060	-1.2518090
C	3.6257520	0.0699500	-0.8872060
C	3.6963820	0.1745800	0.6575870
O	2.3533390	0.0670170	1.1568540
C	-4.0839180	-1.1475710	0.8219300
C	-5.4782040	-1.2222050	0.8249760
C	-6.2342620	-0.3371360	0.0512040
C	-5.5776750	0.6201970	-0.7275700
C	-4.1831630	0.6863680	-0.7368260
C	0.6190270	2.7695240	0.0925300
C	0.9267320	-2.7162760	-0.0626500
C	0.2579240	-3.9550480	0.0504820
C	0.9888330	-5.1358770	-0.0021570
C	2.3765760	-5.0799030	-0.1607470
C	2.9594730	-3.8171390	-0.2555750
N	2.2712550	-2.6721530	-0.2104070
C	0.1848970	3.7005320	-0.8688820
C	0.6398150	5.0203180	-0.8490780
C	1.5499330	5.4364550	0.1269130
C	1.9947520	4.5211580	1.0849820
C	1.5331190	3.2025670	1.0707750
H	-1.7260180	-2.2269750	-0.0593040
H	-1.9155090	2.0150940	0.1246580
H	-3.5122010	-1.8183190	1.4587230
H	-5.9747790	-1.9638890	1.4463990
H	-7.3198660	-0.3911330	0.0566750
H	-6.1519390	1.3106610	-1.3409580
H	-3.6882770	1.4162920	-1.3728390
H	-0.8169370	-3.9983990	0.1845290
H	0.4795530	-6.0926880	0.0838890

H	2.9845920	-5.9783110	-0.2059250
H	4.0380100	-3.7175520	-0.3761960
H	-0.5031530	3.3772390	-1.6458500
H	0.2902520	5.7210680	-1.6035100
H	1.9070250	6.4630890	0.1415880
H	2.6928820	4.8370510	1.8567250
H	1.8604360	2.5059950	1.8370780
H	4.2095390	0.8523740	-1.3797090
H	3.9389690	-0.9152150	-1.2382090
H	4.0988310	1.1386310	0.9854200
H	4.2834920	-0.6378860	1.0932430

5_g (B3LYP/6-31+G*)

C	1.6521100	0.4139840	0.0157760
C	0.5706110	1.3114050	0.0496060
P	-1.0299080	0.6640280	0.0039800
C	-0.9069340	-1.0614620	-0.0407030
C	0.3584550	-1.6487360	-0.0209800
C	1.6003130	-0.9840280	-0.0019870
C	2.8606110	-1.7751620	-0.0085060
O	-1.9903510	1.2775470	-1.2053650
C	-3.0859540	2.0750310	-0.7280860
C	-3.3242710	1.6183050	0.7196470
O	-2.0320610	1.2188970	1.2092470
C	3.9670830	-1.3878010	0.7705060
C	5.1514440	-2.1269500	0.7622900
C	5.2582320	-3.2818750	-0.0178680
C	4.1674890	-3.6836760	-0.7940380
C	2.9874860	-2.9373480	-0.7926570
C	-2.1416630	-1.8970590	-0.0115290
C	0.8345500	2.7727100	0.0249530

C	-0.0345270	3.7129770	0.6157440
C	0.2755000	5.0690960	0.5467840
C	1.4513110	5.4676160	-0.0908300
C	2.2698630	4.4667020	-0.6191980
N	1.9787950	3.1639020	-0.5743470
C	-3.1464290	-1.7696620	-0.9892010
C	-4.2855620	-2.5783610	-0.9546920
C	-4.4423060	-3.5357350	0.0513950
C	-3.4493800	-3.6767710	1.0255020
C	-2.3156000	-2.8622990	0.9977390
H	2.6278410	0.8910620	-0.0236060
H	0.3709140	-2.7376830	-0.0169060
H	3.8906840	-0.5090780	1.4059030
H	5.9884260	-1.8054540	1.3778110
H	6.1786870	-3.8601280	-0.0215880
H	4.2385940	-4.5737620	-1.4149630
H	2.1621090	-3.2459260	-1.4295690
H	-0.9116010	3.3781170	1.1584630
H	-0.3847910	5.8033520	1.0024550
H	1.7344550	6.5133600	-0.1670060
H	3.2075160	4.7233400	-1.1105460
H	-3.0166540	-1.0519020	-1.7940650
H	-5.0442460	-2.4701410	-1.7263550
H	-5.3265220	-4.1676080	0.0743820
H	-3.5608960	-4.4156660	1.8153600
H	-1.5578520	-2.9620980	1.7706940
H	-3.9494690	1.8925210	-1.3734590
H	-2.8028970	3.1319920	-0.7862120
H	-4.0043130	0.7610000	0.7685830
H	-3.6982200	2.4200120	1.3623080

5_h (B3LYP/6-31+G*)

C	-1.6652490	0.3372830	-0.0570370
C	-0.6248480	1.2823020	-0.0919620
P	1.0020760	0.7079870	-0.0143890
C	0.9553440	-1.0190150	0.0695860
C	-0.2818650	-1.6642970	0.0452890
C	-1.5515940	-1.0560810	-0.0069070
C	-2.7774070	-1.9003830	0.0039180
O	1.9212670	1.3856160	1.1922010
C	2.9819630	2.2263190	0.7106600
C	3.2583380	1.7533670	-0.7249000
O	1.9916990	1.2850290	-1.2202650
C	-3.9097090	-1.5274100	0.7523010
C	-5.0621550	-2.3154160	0.7595130
C	-5.1104680	-3.5042340	0.0257060
C	-3.9931740	-3.8917700	-0.7193280
C	-2.8447710	-3.0976970	-0.7328740
C	2.2269660	-1.7978840	0.1066090
C	-0.9571960	2.7294110	-0.1180690
C	-0.1209250	3.6951550	-0.7148680
C	-0.4971030	5.0359740	-0.6901620
C	-1.7052680	5.3937830	-0.0900860
C	-2.4864390	4.3686630	0.4483030
N	-2.1319850	3.0808950	0.4466640
C	3.1802160	-1.6203110	1.1267950
C	4.3531500	-2.3797750	1.1497920
C	4.5959900	-3.3357670	0.1594800
C	3.6555460	-3.5254230	-0.8574020
C	2.4878430	-2.7605150	-0.8862610
H	-2.6622180	0.7679200	-0.0917200
H	-0.2464790	-2.7505310	0.1140510

H	-3.8782710	-0.6209470	1.3515070
H	-5.9201320	-2.0047940	1.3512480
H	-6.0062400	-4.1200340	0.0341420
H	-4.0188690	-4.8088210	-1.3035030
H	-1.9970420	-3.3986920	-1.3437220
H	0.7839550	3.3894560	-1.2280710
H	0.1379510	5.7889960	-1.1510230
H	-2.0401000	6.4261380	-0.0493400
H	-3.4463470	4.5926920	0.9118910
H	2.9844720	-0.9020250	1.9176750
H	5.0710330	-2.2341420	1.9536230
H	5.5064440	-3.9292520	0.1818020
H	3.8343400	-4.2636540	-1.6354560
H	1.7706770	-2.8969490	-1.6916750
H	3.8458090	2.0996320	1.3687700
H	2.6460990	3.2688670	0.7435980
H	3.9783680	0.9281710	-0.7495280
H	3.6024780	2.5590770	-1.3791600

5_i (B3LYP/6-31+G*)

C	-1.6558650	0.3463600	-0.0936120
C	-0.6093880	1.2849530	-0.0987690
P	1.0139090	0.6974070	-0.0112880
C	0.9559120	-1.0303550	-0.0323200
C	-0.2865880	-1.6658070	-0.0413580
C	-1.5521060	-1.0487860	-0.0735420
C	-2.7829090	-1.8853200	-0.0953420
O	1.8886240	1.2654400	1.2797210
C	2.9280180	2.1854010	0.9172330
C	3.2718480	1.8559000	-0.5435570
O	2.0478300	1.3725840	-1.1283940

C	-3.9201830	-1.5223870	0.6507020
C	-5.0770850	-2.3034380	0.6295250
C	-5.1250500	-3.4755130	-0.1309310
C	-4.0030890	-3.8528580	-0.8740120
C	-2.8500000	-3.0653580	-0.8596000
C	2.2191810	-1.8203810	0.0429970
C	-0.9360220	2.7338680	-0.0759340
C	-0.0929820	3.7170320	-0.6338110
C	-0.4667740	5.0572270	-0.5683480
C	-1.6790220	5.3982470	0.0330780
C	-2.4662910	4.3578750	0.5319540
N	-2.1142380	3.0701350	0.4915000
C	2.4304520	-2.7218670	1.1016730
C	3.5912590	-3.4957070	1.1643240
C	4.5718740	-3.3760630	0.1750850
C	4.3769750	-2.4817130	-0.8811380
C	3.2111770	-1.7142550	-0.9489820
H	-2.6497390	0.7841290	-0.1267380
H	-0.2570700	-2.7541790	-0.0158530
H	-3.8896080	-0.6294600	1.2700030
H	-5.9389420	-2.0008070	1.2197640
H	-6.0244650	-4.0858870	-0.1447800
H	-4.0283960	-4.7562790	-1.4790490
H	-1.9989330	-3.3568930	-1.4703210
H	0.8156730	3.4261590	-1.1484970
H	0.1735950	5.8232820	-0.9994310
H	-2.0122510	6.4294650	0.1043360
H	-3.4292840	4.5688560	0.9952770
H	1.6803760	-2.8042340	1.8840550
H	3.7326810	-4.1866560	1.9918700
H	5.4764810	-3.9767840	0.2248160

H	5.1262680	-2.3919850	-1.6642640
H	3.0499880	-1.0470440	-1.7914730
H	3.7737450	2.0300380	1.5923160
H	2.5507220	3.2082730	1.0296360
H	4.0319110	1.0703110	-0.6125120
H	3.5965830	2.7311180	-1.1128430

5_j (B3LYP/6-31+G*)

C	-1.6556180	0.3670580	-0.0507800
C	-0.5990330	1.2943570	-0.0545630
P	1.0184680	0.6872740	-0.0019690
C	0.9406520	-1.0388610	-0.0589750
C	-0.3082940	-1.6612040	-0.0630170
C	-1.5673850	-1.0291920	-0.0629230
C	-2.8072060	-1.8517430	-0.0899480
O	1.9172570	1.2233870	1.2864180
C	2.9727480	2.1252620	0.9247730
C	3.2880380	1.8147730	-0.5467980
O	2.0454840	1.3681040	-1.1214710
C	-3.9145160	-1.4743070	-0.8724720
C	-5.0803090	-2.2422450	-0.8938410
C	-5.1669870	-3.4162500	-0.1402640
C	-4.0748620	-3.8086560	0.6388710
C	-2.9137390	-3.0339100	0.6666680
C	2.1977480	-1.8428960	-0.0498300
C	-0.9066460	2.7457930	0.0194090
C	-0.0542670	3.7337200	-0.5156420
C	-0.4081600	5.0763170	-0.4053860
C	-1.6100590	5.4156010	0.2175380
C	-2.4082650	4.3713800	0.6903950
N	-2.0753730	3.0807360	0.6060040

C	2.4443620	-2.7579380	0.9893590
C	3.5978850	-3.5450420	0.9944970
C	4.5363340	-3.4250640	-0.0347130
C	4.3065820	-2.5168870	-1.0720350
C	3.1477980	-1.7359350	-1.0818030
H	-2.6441470	0.8167270	-0.0097060
H	-0.2904850	-2.7490790	-0.1097450
H	-3.8526630	-0.5803890	-1.4878880
H	-5.9182670	-1.9278880	-1.5116930
H	-6.0728640	-4.0168610	-0.1596060
H	-4.1305780	-4.7139780	1.2389720
H	-2.0870670	-3.3371440	1.3045460
H	0.8452690	3.4461210	-1.0480540
H	0.2393930	5.8463110	-0.8183690
H	-1.9275420	6.4487490	0.3240310
H	-3.3642500	4.5810090	1.1685730
H	1.7277730	-2.8399960	1.8025950
H	3.7669360	-4.2464060	1.8080000
H	5.4354290	-4.0360180	-0.0303670
H	5.0228070	-2.4264660	-1.8854090
H	2.9593750	-1.0559460	-1.9081840
H	3.8244100	1.9379740	1.5841640
H	2.6214470	3.1543510	1.0618300
H	4.0302010	1.0148410	-0.6414650
H	3.6226700	2.6931030	-1.1054270

5_k (B3LYP/6-31+G*)

C	1.6710770	0.3238810	-0.0712240
C	0.6436670	1.2829900	-0.0746180
P	-0.9905990	0.7319290	-0.0135940
C	-0.9708900	-0.9981360	-0.0697650

C	0.2584570	-1.6586890	-0.0764220
C	1.5375780	-1.0688560	-0.0797810
C	2.7498900	-1.9316500	-0.1038560
O	-2.0540940	1.3806850	-1.1184520
C	-3.0956870	2.1777510	-0.5269380
C	-3.1478020	1.7697690	0.9543760
O	-1.8112920	1.3630560	1.2829820
C	3.8857660	-1.6084250	0.6623080
C	5.0251530	-2.4147530	0.6397870
C	5.0563030	-3.5731220	-0.1422530
C	3.9353520	-3.9110240	-0.9056570
C	2.8000420	-3.0982440	-0.8898650
C	-2.2486900	-1.7629350	0.0141720
C	0.9980180	2.7251290	-0.0519750
C	0.2266550	3.7034430	-0.7102210
C	0.6200030	5.0380140	-0.6527800
C	1.7827340	5.3743540	0.0426160
C	2.5034240	4.3365180	0.6375180
N	2.1304010	3.0536850	0.6038850
C	-3.2815270	-1.5901750	-0.9266270
C	-4.4597020	-2.3367120	-0.8416780
C	-4.6297270	-3.2753110	0.1799660
C	-3.6104400	-3.4602070	1.1187440
C	-2.4364130	-2.7083790	1.0398670
H	2.6744680	0.7411400	-0.0864890
H	0.2067050	-2.7465300	-0.0718580
H	3.8674050	-0.7272570	1.2987190
H	5.8862360	-2.1428170	1.2458990
H	5.9419520	-4.2032590	-0.1571580
H	3.9479690	-4.8033300	-1.5273800
H	1.9502440	-3.3586760	-1.5161800

H	-0.6388730	3.4104250	-1.2942090
H	0.0363080	5.8021720	-1.1606930
H	2.1296250	6.4011610	0.1128240
H	3.4273560	4.5447240	1.1754740
H	-3.1446350	-0.8860940	-1.7420600
H	-5.2398390	-2.1946810	-1.5859830
H	-5.5447280	-3.8588800	0.2420870
H	-3.7316320	-4.1847540	1.9203770
H	-1.6565470	-2.8421330	1.7851640
H	-4.0284850	1.9659590	-1.0561390
H	-2.8420500	3.2370410	-0.6471290
H	-3.8312560	0.9295450	1.1203540
H	-3.4203970	2.6002070	1.6106950

5_I (B3LYP/6-31+G*)

C	1.6785570	0.2999100	-0.0323250
C	0.6669230	1.2763640	-0.0333580
P	-0.9762060	0.7491440	-0.0071860
C	-0.9819830	-0.9795900	-0.0945500
C	0.2357600	-1.6605360	-0.0948800
C	1.5240140	-1.0898720	-0.0690670
C	2.7229900	-1.9709260	-0.0971580
O	-2.0168220	1.4231250	-1.1180960
C	-3.0693060	2.2090820	-0.5316040
C	-3.1388710	1.7848540	0.9446120
O	-1.8059370	1.3763960	1.2857130
C	3.8539460	-1.6376430	-0.8658670
C	4.9811870	-2.4610620	-0.8887280
C	5.0043700	-3.6476460	-0.1503780
C	3.8880800	-3.9964750	0.6150730
C	2.7657410	-3.1666040	0.6443560

C	-2.2751950	-1.7239260	-0.0762390
C	1.0425240	2.7113000	0.0426360
C	0.2777770	3.7251090	-0.5682360
C	0.6900440	5.0508720	-0.4633360
C	1.8646190	5.3446660	0.2315620
C	2.5772400	4.2747420	0.7772400
N	2.1863000	2.9994610	0.6971160
C	-3.2525090	-1.5448610	-1.0731960
C	-4.4445140	-2.2735860	-1.0457460
C	-4.6839030	-3.1993950	-0.0261740
C	-3.7204410	-3.3899850	0.9686020
C	-2.5325600	-2.6561360	0.9462300
H	2.6871030	0.7013000	0.0243280
H	0.1672910	-2.7454900	-0.1594740
H	3.8408070	-0.7339740	-1.4699490
H	5.8384340	-2.1799680	-1.4960650
H	5.8800720	-4.2913480	-0.1708610
H	3.8948110	-4.9110470	1.2036120
H	1.9201180	-3.4378350	1.2717380
H	-0.5984330	3.4679310	-1.1530660
H	0.1110030	5.8418830	-0.9341300
H	2.2259360	6.3633360	0.3380280
H	3.5095750	4.4488370	1.3128070
H	-3.0618790	-0.8485650	-1.8844960
H	-5.1810550	-2.1275580	-1.8324500
H	-5.6096320	-3.7689430	-0.0089450
H	-3.8959990	-4.1049110	1.7688280
H	-1.7959800	-2.7934960	1.7337780
H	-3.9945380	1.9988150	-1.0745350
H	-2.8191600	3.2707180	-0.6379140
H	-3.8230690	0.9416880	1.0920440

H	-3.4212590	2.6071450	1.6069470

5_m (B3LYP/6-31+G*)

C	1.6744920	0.2955650	-0.0345700
C	0.6584330	1.2659820	-0.0667050
P	-0.9851320	0.7387500	-0.0191940
C	-0.9789550	-0.9899380	0.0305980
C	0.2394330	-1.6690780	0.0078820
C	1.5259070	-1.0953950	-0.0153760
C	2.7284650	-1.9719480	-0.0097210
O	-1.9997280	1.2976290	-1.2134990
C	-3.0383950	2.1719110	-0.7464050
C	-3.1443860	1.9349430	0.7707650
O	-1.8454140	1.4869010	1.1879880
C	3.8633270	-1.6434230	0.7555890
C	4.9940260	-2.4623060	0.7586370
C	5.0172040	-3.6385630	0.0036240
C	3.8969800	-3.9821800	-0.7583190
C	2.7705820	-3.1570870	-0.7678330
C	-2.2708050	-1.7368580	0.0815090
C	1.0324920	2.7031310	-0.0894590
C	0.2890350	3.6662130	-0.7995300
C	0.6981720	4.9971590	-0.7818260
C	1.8494550	5.3441040	-0.0728000
C	2.5433570	4.3196890	0.5750210
N	2.1546850	3.0410340	0.5789210
C	-2.6356230	-2.5947470	-0.9710510
C	-3.8247900	-3.3254360	-0.9217020
C	-4.6800690	-3.2041910	0.1775000
C	-4.3316000	-2.3530420	1.2299820
C	-3.1372500	-1.6286060	1.1840520

H	2.6827460	0.7010710	-0.0504140
H	0.1722430	-2.7550990	0.0540380
H	3.8505000	-0.7479760	1.3718290
H	5.8543700	-2.1858760	1.3637310
H	5.8959320	-4.2784600	0.0088140
H	3.9033430	-4.8889620	-1.3588170
H	1.9209790	-3.4237090	-1.3918950
H	-0.5666550	3.3629780	-1.3925730
H	0.1360100	5.7493910	-1.3302130
H	2.2080520	6.3684600	-0.0316160
H	3.4579360	4.5359650	1.1256300
H	-1.9819730	-2.6780620	-1.8355900
H	-4.0861920	-3.9845530	-1.7460400
H	-5.6064380	-3.7716900	0.2158020
H	-4.9821710	-2.2636940	2.0968330
H	-2.8548240	-0.9945850	2.0205790
H	-3.9607780	1.9182020	-1.2753750
H	-2.7623060	3.2073650	-0.9757310
H	-3.8826560	1.1612750	1.0075010
H	-3.3797150	2.8478500	1.3238830

5_n (B3LYP/6-31+G*)

C	-1.6664290	0.3491400	-0.0041110
C	-0.6207490	1.2873990	0.0271510
P	1.0064060	0.7082440	0.0165780
C	0.9464100	-1.0204590	-0.0070160
C	-0.2934580	-1.6595320	0.0106910
C	-1.5613450	-1.0459680	0.0058380
C	-2.7897990	-1.8854990	0.0050200
O	2.0198920	1.2445820	1.2216450
C	3.0935340	2.0812030	0.7660240

C	3.2093860	1.8409030	-0.7507180
O	1.9065780	1.4197810	-1.1831890
C	-3.9147830	-1.5291970	0.7723140
C	-5.0697760	-2.3134920	0.7698570
C	-5.1274840	-3.4835240	0.0073000
C	-4.0176800	-3.8549230	-0.7569240
C	-2.8673040	-3.0636720	-0.7614180
C	2.2131790	-1.8095290	0.0048910
C	-0.9467110	2.7360400	-0.0064460
C	-0.1758710	3.6980060	0.6755710
C	-0.5371340	5.0409220	0.6051650
C	-1.6693190	5.4018530	-0.1272960
C	-2.3940100	4.3790870	-0.7432860
N	-2.0510300	3.0883740	-0.6961430
C	2.4979900	-2.6817760	1.0703760
C	3.6634160	-3.4512820	1.0753910
C	4.5750030	-3.3555740	0.0196960
C	4.3059010	-2.4912440	-1.0451760
C	3.1349830	-1.7283440	-1.0544260
H	-2.6597930	0.7868790	-0.0620760
H	-0.2599470	-2.7479160	0.0331270
H	-3.8757030	-0.6386600	1.3945740
H	-5.9219340	-2.0151410	1.3761320
H	-6.0249580	-4.0968890	0.0079370
H	-4.0510190	-4.7565370	-1.3642250
H	-2.0269800	-3.3501260	-1.3890740
H	0.6638150	3.3876050	1.2874920
H	0.0471870	5.7925920	1.1306920
H	-1.9905450	6.4360570	-0.2098910
H	-3.2959330	4.6064470	-1.3100530
H	1.8011880	-2.7456880	1.9022220

H	3.8625710	-4.1204060	1.9089740
H	5.4832910	-3.9527800	0.0244080
H	5.0001320	-2.4217270	-1.8794070
H	2.9133490	-1.0858420	-1.9027770
H	4.0006140	1.7952860	1.3049920
H	2.8521280	3.1256230	0.9934600
H	3.9343060	1.0523970	-0.9791880
H	3.4698940	2.7484110	-1.3014580

5_a_ω_TS_a (B3LYP/6-31+G*)

C	0.9882480	-1.3993190	0.0074200
C	-0.4060310	-1.3452200	-0.0091690
P	-1.1965870	0.1852700	-0.0164320
C	0.0255080	1.4040360	0.0543210
C	1.3687360	1.0058040	0.0519180
C	1.8722290	-0.3052790	0.0326440
C	3.3406220	-0.5395340	0.0499900
O	-2.3717180	0.3257060	1.1545280
C	-3.7046220	0.2672890	0.6172690
C	-3.5835640	0.7782360	-0.8256450
O	-2.2733520	0.3811850	-1.2577010
C	3.9234600	-1.5594390	-0.7264790
C	5.3010170	-1.7856620	-0.7072720
C	6.1371990	-0.9893520	0.0805850
C	5.5764460	0.0321990	0.8525350
C	4.1972390	0.2493540	0.8414420
C	-0.3432310	2.8466710	0.0039460
C	-1.2179830	-2.6045080	0.0030660
C	-1.5704860	-3.2066530	1.2226770
C	-2.3006550	-4.3954050	1.2102810
C	-2.6629600	-4.9463360	-0.0193480

C	-2.2739790	-4.2742560	-1.1808480
N	-1.5729780	-3.1332730	-1.1830270
C	-1.2613510	3.4111920	0.9098320
C	-1.5756620	4.7717440	0.8571700
C	-0.9760810	5.5997390	-0.0956230
C	-0.0602860	5.0531750	-0.9997630
C	0.2469650	3.6922540	-0.9544790
H	1.4223570	-2.3978940	0.0266730
H	2.0940750	1.8180950	0.0590270
H	3.2928790	-2.1647220	-1.3729740
H	5.7229790	-2.5773760	-1.3220730
H	7.2103790	-1.1620200	0.0922930
H	6.2123880	0.6544780	1.4780020
H	3.7749020	1.0254510	1.4749750
H	-1.2682140	-2.7426780	2.1567420
H	-2.5790320	-4.8808300	2.1424920
H	-3.2302610	-5.8708230	-0.0822860
H	-2.5362070	-4.6694270	-2.1611610
H	-1.7079430	2.7832800	1.6753050
H	-2.2801370	5.1876250	1.5738510
H	-1.2178980	6.6589110	-0.1325210
H	0.4088760	5.6849000	-1.7502160
H	0.9400970	3.2703170	-1.6775750
H	-4.3506410	0.8935890	1.2381800
H	-4.0562250	-0.7705510	0.6522800
H	-3.6606510	1.8701950	-0.8790190
H	-4.3161740	0.3238160	-1.4977560

5_a_ω_TS_b (B3LYP/6-31+G*)

C	1.6834520	0.2041400	-0.0573250
C	0.7353100	1.2474600	-0.0450390

P	-0.9362710	0.8086440	-0.0374430
C	-1.0393230	-0.9212240	-0.0474160
C	0.1329490	-1.6727090	-0.0405050
C	1.4510600	-1.1729230	-0.0564270
C	2.5987020	-2.1205630	-0.0764570
O	-1.9025520	1.4792490	-1.2144830
C	-2.9829580	2.2802130	-0.7109790
C	-3.0912380	1.9609680	0.7939920
O	-1.7841850	1.5231100	1.1952610
C	3.7611810	-1.8701760	0.6769020
C	4.8390010	-2.7570370	0.6577320
C	4.7800750	-3.9251200	-0.1081410
C	3.6317710	-4.1911950	-0.8590310
C	2.5583640	-3.2980780	-0.8465190
C	-2.3735570	-1.5850270	0.0335470
C	1.2128170	2.6540910	-0.0173290
C	0.3581650	3.7655660	-0.1627630
C	0.8850030	5.0526990	-0.1242880
C	2.2586720	5.2234880	0.0540650
C	3.0350740	4.0703810	0.1833430
N	2.5402320	2.8312250	0.1529260
C	-3.3632350	-1.3862010	-0.9472860
C	-4.5955560	-2.0399080	-0.8636270
C	-4.8635660	-2.9093340	0.1975530
C	-3.8876170	-3.1199430	1.1760710
C	-2.6590480	-2.4605690	1.0976660
H	2.7127420	0.5492460	-0.0816050
H	0.0021740	-2.7536150	-0.0158880
H	3.8121290	-0.9810760	1.3001790
H	5.7225410	-2.5399400	1.2535360
H	5.6177890	-4.6177940	-0.1203120

H	3.5749180	-5.0899680	-1.4687880
H	1.6873170	-3.5044150	-1.4637510
H	-0.7048680	3.6306180	-0.3125600
H	0.2284290	5.9121990	-0.2367470
H	2.7140380	6.2086390	0.0902580
H	4.1131230	4.1437660	0.3214520
H	-3.1504330	-0.7378020	-1.7922560
H	-5.3413930	-1.8808570	-1.6389890
H	-5.8207030	-3.4209230	0.2589340
H	-4.0843940	-3.7927100	2.0072650
H	-1.9113500	-2.6135720	1.8716770
H	-3.8919530	2.0138610	-1.2573520
H	-2.7534030	3.3359020	-0.8955260
H	-3.8124290	1.1594160	0.9862080
H	-3.3509130	2.8379330	1.3927570

5_a_ω_TS_c (B3LYP/6-31+G*)

C	-0.8726150	-1.4770760	-0.0304820
C	0.5131740	-1.3112550	-0.0141750
P	1.1790920	0.2762780	-0.0512720
C	-0.1365360	1.3963880	-0.0492910
C	-1.4430920	0.8907920	-0.0409220
C	-1.8405270	-0.4566060	-0.0458170
C	-3.2866970	-0.8030640	-0.0689860
O	2.2791420	0.5797750	-1.2566680
C	3.6373100	0.5908940	-0.7873760
C	3.5477580	1.0233820	0.6826480
O	2.3009430	0.4870030	1.1557380
C	-3.7931740	-1.8743250	0.6916330
C	-5.1501560	-2.2014620	0.6676790
C	-6.0427130	-1.4580650	-0.1099860

C	-5.5583720	-0.3879230	-0.8675320
C	-4.1990120	-0.0696850	-0.8514090
C	0.1135470	2.8626120	0.0357840
C	1.4247220	-2.5002580	0.0134450
C	1.8526070	-3.0379600	1.2389180
C	2.6755420	-4.1641590	1.2388490
C	3.0524810	-4.7189360	0.0151950
C	2.5855660	-4.1120190	-1.1532420
N	1.7958050	-3.0302930	-1.1671640
C	0.9784150	3.5253860	-0.8560640
C	1.1801130	4.9051390	-0.7664060
C	0.5175290	5.6555540	0.2086860
C	-0.3479490	5.0115450	1.0979950
C	-0.5417650	3.6315350	1.0165670
H	-1.2251000	-2.5071330	-0.0523390
H	-2.2313100	1.6419850	-0.0233800
H	-3.1199580	-2.4427840	1.3287060
H	-5.5126350	-3.0314030	1.2699330
H	-7.1001100	-1.7096420	-0.1258800
H	-6.2378390	0.1936370	-1.4863300
H	-3.8346310	0.7431500	-1.4746200
H	1.5374670	-2.5719720	2.1675610
H	3.0139270	-4.5994660	2.1759650
H	3.6899730	-5.5971620	-0.0377140
H	2.8568040	-4.5115120	-2.1293170
H	1.4711400	2.9569780	-1.6390750
H	1.8454440	5.3964510	-1.4726640
H	0.6715640	6.7296720	0.2736730
H	-0.8660050	5.5823260	1.8649620
H	-1.1963580	3.1355040	1.7285480
H	4.2046430	1.2931370	-1.4038070

H	4.0606660	-0.4145930	-0.8943490
H	3.5261690	2.1141930	0.7850690
H	4.3515110	0.6096760	1.2974450

5_a_θ_TS_a (B3LYP/6-31+G*)

C	-1.2360320	1.2260990	0.0341030
C	0.1575890	1.4341060	0.0077650
P	1.2053490	0.0491260	0.0695130
C	0.1914410	-1.3632820	0.0553650
C	-1.1942970	-1.2098420	0.0447040
C	-1.9069350	0.0049270	0.0556530
C	-3.4054050	-0.0195970	0.0971110
O	2.3112840	-0.0529860	1.2918880
C	3.6555770	0.2080190	0.8523550
C	3.6780500	-0.1436900	-0.6472630
O	2.3369120	0.0505690	-1.1272190
C	-4.1578430	-0.0445050	-1.0885680
C	-5.5552140	-0.0683100	-1.0527110
C	-6.2255310	-0.0677480	0.1737470
C	-5.4897110	-0.0436480	1.3618750
C	-4.0925140	-0.0205100	1.3220340
C	0.8019330	-2.7211240	-0.0254440
C	0.7487250	2.7811300	-0.0782500
C	-0.0013360	3.9273360	-0.4219960
C	0.6239910	5.1679820	-0.4646330
C	1.9896010	5.2588670	-0.1812290
C	2.6609990	4.0755480	0.1241530
N	2.0733220	2.8761850	0.1785770
C	1.7687590	-3.1665410	0.8958890
C	2.3032780	-4.4544710	0.8064550
C	1.8815680	-5.3282220	-0.1994450

C	0.9196630	-4.9007000	-1.1193380
C	0.3916850	-3.6111110	-1.0364140
H	-1.8597400	2.1168180	0.0486810
H	-1.7783250	-2.1296340	0.0476010
H	-3.6394540	-0.0441050	-2.0446790
H	-6.1190680	-0.0870820	-1.9824360
H	-7.3122170	-0.0862100	0.2034640
H	-6.0025070	-0.0433830	2.3208660
H	-3.5231960	-0.0028340	2.2484570
H	-1.0539410	3.8487560	-0.6699080
H	0.0523510	6.0549270	-0.7270690
H	2.5174450	6.2074650	-0.2051690
H	3.7291260	4.0901360	0.3400060
H	2.0803480	-2.5057660	1.6985640
H	3.0414780	-4.7798760	1.5358610
H	2.2957560	-6.3311720	-0.2647940
H	0.5861800	-5.5681360	-1.9103940
H	-0.3373180	-3.2779240	-1.7706450
H	4.3263220	-0.4254600	1.4390250
H	3.8743010	1.2635980	1.0242740
H	3.9563940	-1.1893050	-0.8190590
H	4.3387490	0.5140600	-1.2182380

5_a_θ_TS_b (B3LYP/6-31+G*)

C	1.2238720	1.2148990	-0.0318770
C	-0.1653960	1.4254630	-0.0068630
P	-1.2239650	0.0520430	-0.0863480
C	-0.2057190	-1.3539560	-0.0490700
C	1.1769860	-1.2038480	-0.0346090
C	1.9192830	0.0000000	-0.0559020
C	3.4142840	-0.0296290	-0.1030450

O	-2.3083460	-0.0588140	-1.3247740
C	-3.6575910	0.2175430	-0.9077480
C	-3.7053350	-0.1291580	0.5918740
O	-2.3711510	0.0691040	1.0925510
C	4.1866550	1.1519340	-0.1283590
C	5.5807090	1.1254930	-0.1669210
C	6.2706090	-0.0885760	-0.1834450
C	5.5314830	-1.2725360	-0.1634050
C	4.1368060	-1.2420800	-0.1253090
C	-0.8159530	-2.7122860	0.0432210
C	-0.7496240	2.7762530	0.0895380
C	-0.0101390	3.9030830	0.5094980
C	-0.6255570	5.1489560	0.5528860
C	-1.9724350	5.2611930	0.1975070
C	-2.6377270	4.0934340	-0.1744230
N	-2.0580820	2.8902070	-0.2319260
C	-1.7520430	-3.1786220	-0.8987710
C	-2.2870820	-4.4655880	-0.7985600
C	-1.8968740	-5.3157430	0.2398480
C	-0.9665110	-4.8662920	1.1813940
C	-0.4374540	-3.5777060	1.0869600
H	1.8130850	2.1242920	-0.0421150
H	1.7221740	-2.1425610	-0.0207880
H	3.7072220	2.1247710	-0.1224350
H	6.1280570	2.0651380	-0.1859700
H	7.3566660	-0.1108840	-0.2142830
H	6.0393940	-2.2340720	-0.1796040
H	3.6179470	-2.1940090	-0.1168530
H	1.0241450	3.8039690	0.8200640
H	-0.0624540	6.0220140	0.8736610
H	-2.4924820	6.2141150	0.2193510

H	-3.6930320	4.1246180	-0.4442730
H	-2.0386430	-2.5357980	-1.7251820
H	-3.0005910	-4.8088170	-1.5441170
H	-2.3115690	-6.3178710	0.3137470
H	-0.6588680	-5.5153240	1.9977760
H	0.2661730	-3.2258320	1.8371950
H	-4.3245990	-0.4112150	-1.5033560
H	-3.8624150	1.2748390	-1.0863730
H	-3.9846520	-1.1745250	0.7628680
H	-4.3750110	0.5297780	1.1506480

5_b_ω_TS_a (B3LYP/6-31+G*)

C	0.9265440	-1.4403150	-0.0579960
C	-0.4643730	-1.3251390	-0.0484980
P	-1.1884920	0.2379210	-0.0199490
C	0.0877820	1.4009980	-0.0221530
C	1.4122180	0.9455350	-0.0203590
C	1.8581490	-0.3863970	-0.0436670
C	3.3155360	-0.6821600	-0.0621380
O	-2.2550960	0.4695290	1.2341430
C	-3.6293360	0.4425670	0.8181370
C	-3.6177520	0.8856990	-0.6521210
O	-2.3578110	0.4374220	-1.1777900
C	3.8520870	-1.7508640	0.6813230
C	5.2194610	-2.0318140	0.6624650
C	6.0925130	-1.2433180	-0.0925200
C	5.5781920	-0.1749740	-0.8326990
C	4.2087820	0.0970070	-0.8219900
C	-0.2172350	2.8587070	0.0362150
C	-1.3302890	-2.5480480	-0.0415810
C	-1.6801200	-3.1579570	1.1750680

C	-2.4634090	-4.3125290	1.1590220
C	-2.8790750	-4.8226020	-0.0712000
C	-2.4877270	-4.1457550	-1.2293750
N	-1.7370370	-3.0371240	-1.2279390
C	0.3379390	3.6599490	1.0505500
C	0.0880200	5.0328700	1.0991890
C	-0.7340870	5.6339640	0.1413480
C	-1.2955120	4.8499200	-0.8702730
C	-1.0368090	3.4780050	-0.9255510
H	1.3161170	-2.4561630	-0.1046090
H	2.1713270	1.7260820	0.0045150
H	3.1934320	-2.3543890	1.3011450
H	5.6048780	-2.8611600	1.2511520
H	7.1578950	-1.4588160	-0.1043860
H	6.2425020	0.4414390	-1.4339680
H	3.8223700	0.9096420	-1.4320550
H	-1.3353290	-2.7259900	2.1098920
H	-2.7412370	-4.8030480	2.0887630
H	-3.4885440	-5.7196200	-0.1373300
H	-2.7902450	-4.5101160	-2.2100130
H	0.9594260	3.1950480	1.8116200
H	0.5288130	5.6316070	1.8926680
H	-0.9316320	6.7021820	0.1808920
H	-1.9237700	5.3091050	-1.6300380
H	-1.4455380	2.8830180	-1.7378200
H	-4.0173700	-0.5766240	0.9319360
H	-4.1937960	1.1231950	1.4608680
H	-4.4140790	0.4237760	-1.2413200
H	-3.6743060	1.9761850	-0.7450820

5_b_ω_TS_b (B3LYP/6-31+G*)

C	-1.6784130	0.2288450	-0.0184920
C	-0.7099580	1.2525050	-0.0021810
P	0.9540990	0.7829630	0.0375110
C	1.0214460	-0.9457000	-0.0346290
C	-0.1638150	-1.6764060	-0.0245430
C	-1.4724790	-1.1528010	-0.0154820
C	-2.6381790	-2.0780180	-0.0146230
O	1.8673680	1.3377020	1.3106320
C	2.9583680	2.1913570	0.9448380
C	3.1329400	2.0450970	-0.5830200
O	1.8734200	1.5772740	-1.0910880
C	-3.7812210	-1.8044780	0.7599620
C	-4.8771170	-2.6692710	0.7616330
C	-4.8557020	-3.8384850	-0.0041910
C	-3.7270030	-4.1276750	-0.7761750
C	-2.6359620	-3.2563360	-0.7846420
C	2.3461050	-1.6337890	-0.0234010
C	-1.1590780	2.6680500	-0.0426880
C	-0.2996610	3.7601790	0.1933600
C	-0.7986050	5.0578050	0.1360490
C	-2.1498290	5.2576540	-0.1505980
C	-2.9342360	4.1218980	-0.3606720
N	-2.4657390	2.8731120	-0.3125020
C	2.6931580	-2.4894950	1.0372170
C	3.9145090	-3.1668120	1.0418610
C	4.8198420	-2.9932310	-0.0092660
C	4.4891970	-2.1438810	-1.0689780
C	3.2631080	-1.4729960	-1.0777340
H	-2.7004520	0.5932090	-0.0658390
H	-0.0510510	-2.7596330	-0.0175800
H	-3.8021670	-0.9145320	1.3838220

H	-5.7448710	-2.4341670	1.3735810
H	-5.7070380	-4.5144420	-0.0001440
H	-3.6996340	-5.0276550	-1.3861900
H	-1.7811850	-3.4805490	-1.4181380
H	0.7443520	3.6023070	0.4304730
H	-0.1387360	5.9025980	0.3187690
H	-2.5829840	6.2518640	-0.2066430
H	-3.9963080	4.2182300	-0.5828290
H	1.9995190	-2.6136140	1.8648590
H	4.1613490	-3.8252120	1.8712010
H	5.7711910	-3.5191230	-0.0051870
H	5.1793360	-2.0144880	-1.8994340
H	2.9965200	-0.8411690	-1.9211200
H	2.7151550	3.2193320	1.2375410
H	3.8471560	1.8674580	1.4933960
H	3.3587060	2.9957750	-1.0734340
H	3.9090510	1.3136720	-0.8302500

5_b_ω_TS_c (B3LYP/6-31+G*)

C	-0.8866950	-1.4615470	0.0362940
C	0.4999490	-1.3068710	0.0278340
P	1.1778710	0.2762140	-0.0328450
C	-0.1281150	1.4040720	0.0311610
C	-1.4391630	0.9102660	0.0367150
C	-1.8473900	-0.4336730	0.0365110
C	-3.2952930	-0.7727260	0.0511180
O	2.1897010	0.6074490	-1.3023220
C	3.5778670	0.5378300	-0.9449290
C	3.6263420	0.9160440	0.5420200
O	2.3887840	0.4365060	1.0977470
C	-3.7978150	-1.8441330	-0.7120930

C	-5.1557000	-2.1683120	-0.6976350
C	-6.0525330	-1.4219970	0.0722680
C	-5.5719440	-0.3511870	0.8314180
C	-4.2118480	-0.0356420	0.8250630
C	0.1362180	2.8696800	-0.0037550
C	1.4006840	-2.5042060	0.0488660
C	1.8601760	-3.0290320	1.2682990
C	2.6712160	-4.1636620	1.2593770
C	3.0060330	-4.7393320	0.0329860
C	2.5105350	-4.1440100	-1.1294160
N	1.7309240	-3.0546060	-1.1348130
C	-0.4852800	3.6816110	-0.9701210
C	-0.2705150	5.0609720	-0.9949790
C	0.5815260	5.6590620	-0.0617330
C	1.2095170	4.8646200	0.9014720
C	0.9863160	3.4858440	0.9332200
H	-1.2478960	-2.4881760	0.0741330
H	-2.2203090	1.6691400	0.0358370
H	-3.1206710	-2.4130390	-1.3444750
H	-5.5153330	-2.9978510	-1.3021090
H	-7.1106360	-1.6710230	0.0801310
H	-6.2552240	0.2333770	1.4431890
H	-3.8512180	0.7788700	1.4484130
H	1.5769480	-2.5476120	2.1992580
H	3.0328290	-4.5897180	2.1920400
H	3.6330720	-5.6246440	-0.0263300
H	2.7488650	-4.5592840	-2.1075140
H	-1.1288540	3.2205070	-1.7148170
H	-0.7617540	5.6674590	-1.7520880
H	0.7516900	6.7324770	-0.0833900
H	1.8632590	5.3203920	1.6415610

H	1.4485510	2.8817980	1.7095590
H	3.9430590	-0.4808920	-1.1203730
H	4.1274360	1.2383880	-1.5788160
H	4.4478820	0.4335750	1.0780870
H	3.6813690	2.0016210	0.6809000

5_b_θ_TS_a (B3LYP/6-31+G*)

C	1.2183840	-1.2416260	0.0006980
C	-0.1783930	-1.4256820	0.0046220
P	-1.2019780	-0.0214140	0.0501910
C	-0.1664340	1.3690180	-0.0639990
C	1.2166680	1.1938810	-0.0592900
C	1.9098030	-0.0315370	-0.0197330
C	3.4089930	-0.0317390	-0.0125960
O	-2.2291810	0.1697930	1.3249250
C	-3.5972120	-0.1177260	0.9987250
C	-3.7220730	0.0983320	-0.5239460
O	-2.4047200	-0.0726310	-1.0760270
C	4.1353860	-0.0804530	-1.2136770
C	5.5333950	-0.0804070	-1.2084620
C	6.2306030	-0.0313110	0.0019270
C	5.5209520	0.0180630	1.2050690
C	4.1230030	0.0183800	1.1958480
C	-0.7593280	2.7365320	-0.0887110
C	-0.7934440	-2.7642350	-0.0131840
C	-0.0765730	-3.9320010	-0.3549570
C	-0.7220680	-5.1627600	-0.3341510
C	-2.0752660	-5.2226400	0.0108310
C	-2.7145080	-4.0202500	0.3105960
N	-2.1068540	-2.8294780	0.3039450
C	-0.3577690	3.6985780	0.8561280

C	-0.8758260	4.9947690	0.8271850
C	-1.8172990	5.3555330	-0.1411730
C	-2.2294110	4.4091280	-1.0835280
C	-1.7043860	3.1146160	-1.0605590
H	1.8269540	-2.1426130	0.0270830
H	1.8151450	2.1031760	-0.1065570
H	3.5961080	-0.1172700	-2.1573880
H	6.0768630	-0.1184530	-2.1496560
H	7.3178370	-0.0311840	0.0075410
H	6.0546630	0.0563240	2.1518200
H	3.5741210	0.0556440	2.1340120
H	0.9652230	-3.8770940	-0.6506720
H	-0.1763760	-6.0663230	-0.5951210
H	-2.6180980	-6.1626620	0.0377180
H	-3.7719500	-4.0111160	0.5740230
H	0.3559990	3.4180250	1.6264030
H	-0.5503810	5.7205250	1.5686680
H	-2.2231820	6.3637680	-0.1625420
H	-2.9510820	4.6823780	-1.8499090
H	-2.0058290	2.3946700	-1.8161190
H	-3.8101510	-1.1513470	1.2782650
H	-4.2255320	0.5726940	1.5676550
H	-4.3806090	-0.6364490	-0.9942740
H	-4.0640360	1.1107940	-0.7645960

5_b_θ_TS_b (B3LYP/6-31+G*)

C	1.1921130	-1.2463760	-0.0029260
C	-0.2024890	-1.4171600	0.0005490
P	-1.2206330	-0.0118700	0.0598920
C	-0.1655190	1.3600410	-0.0672000
C	1.2124420	1.1719580	-0.0698990

C	1.9214980	-0.0510450	-0.0238180
C	3.4173260	-0.0618630	-0.0088880
O	-2.2318150	0.1954460	1.3427050
C	-3.6051250	-0.0839430	1.0269290
C	-3.7395790	0.1474660	-0.4913210
O	-2.4312610	-0.0596320	-1.0557210
C	4.1577290	-1.2636950	0.0160770
C	5.5524540	-1.2748280	0.0304760
C	6.2752250	-0.0799830	0.0205830
C	5.5682160	1.1234020	-0.0022890
C	4.1728010	1.1305350	-0.0162430
C	-0.7416410	2.7356350	-0.0956030
C	-0.8263140	-2.7530340	-0.0272960
C	-0.1334650	-3.9131410	-0.4357790
C	-0.7840330	-5.1415890	-0.4168550
C	-2.1197680	-5.2039260	-0.0105610
C	-2.7387390	-4.0066900	0.3473790
N	-2.1247190	-2.8191180	0.3447810
C	-0.3443200	3.6890580	0.8594170
C	-0.8483550	4.9908600	0.8275480
C	-1.7709190	5.3647440	-0.1538990
C	-2.1793930	4.4262380	-1.1057390
C	-1.6688520	3.1259550	-1.0793390
H	1.7553070	-2.1719330	0.0251570
H	1.7830960	2.0936000	-0.1295460
H	3.6520280	-2.2230390	0.0271290
H	6.0743950	-2.2288120	0.0502180
H	7.3618630	-0.0870560	0.0319520
H	6.1022140	2.0708110	-0.0087830
H	3.6799380	2.0961510	-0.0311670
H	0.8913150	-3.8527730	-0.7855750

H	-0.2572950	-6.0401660	-0.7288150
H	-2.6662060	-6.1418160	0.0168530
H	-3.7836480	-3.9993440	0.6564730
H	0.3532570	3.3971420	1.6403220
H	-0.5273330	5.7103510	1.5769670
H	-2.1657170	6.3772940	-0.1776840
H	-2.8867800	4.7099400	-1.8815080
H	-1.9676650	2.4111390	-1.8409220
H	-3.8188110	-1.1195960	1.2983760
H	-4.2248190	0.6036590	1.6082760
H	-4.4231840	-0.5636020	-0.9621010
H	-4.0525770	1.1716860	-0.7211750

5_b_ϕ_TS_a (B3LYP/6-31+G*)

C	-1.4586480	0.9605520	0.0040900
C	-0.1309840	1.4276970	-0.0077870
P	1.1577680	0.2640160	0.0500240
C	0.4210550	-1.3055010	0.0134760
C	-0.9622910	-1.4245420	0.0066080
C	-1.8987590	-0.3651660	0.0166830
C	-3.3551510	-0.6656980	0.0384680
O	2.2471790	0.3264420	1.2845320
C	3.5298750	0.8297880	0.8761840
C	3.6312890	0.5401180	-0.6347470
O	2.2817510	0.4948190	-1.1308090
C	-4.2452090	0.0943370	0.8212290
C	-5.6144680	-0.1788960	0.8323700
C	-6.1312090	-1.2303280	0.0700510
C	-5.2609970	-2.0008080	-0.7066230
C	-3.8938930	-1.7186640	-0.7255960
C	1.3088430	-2.5118680	0.0001940

C	0.2012760	2.8614080	-0.0805230
C	-0.7368880	3.8506430	-0.4474020
C	-0.3484770	5.1851780	-0.4756860
C	0.9688880	5.5238570	-0.1543870
C	1.8375610	4.4831190	0.1728000
N	1.4784920	3.1962360	0.2138330
C	1.7293900	-3.1131100	1.1981750
C	2.5419320	-4.2498430	1.1797070
C	2.9543950	-4.7993310	-0.0378970
C	2.5481450	-4.2063620	-1.2366890
C	1.7337290	-3.0704360	-1.2169620
H	-2.2329150	1.7234740	-0.0107130
H	-1.3522910	-2.4413050	0.0198050
H	-3.8554470	0.8913330	1.4498190
H	-6.2764550	0.4221450	1.4515440
H	-7.1962380	-1.4474410	0.0823370
H	-5.6485190	-2.8170880	-1.3118580
H	-3.2379020	-2.3077540	-1.3618180
H	-1.7492370	3.5798790	-0.7262340
H	-1.0649920	5.9531890	-0.7568740
H	1.3142710	6.5532260	-0.1662900
H	2.8783650	4.6926390	0.4183870
H	1.4143200	-2.6823600	2.1447410
H	2.8525210	-4.7055940	2.1168230
H	3.5865720	-5.6837540	-0.0522340
H	2.8629460	-4.6278370	-2.1883560
H	1.4190410	-2.6084260	-2.1492470
H	3.5631090	1.9010710	1.0857450
H	4.2927250	0.3007260	1.4533260
H	4.1610940	1.3299620	-1.1737650
H	4.1052210	-0.4269120	-0.8365370

5_b_ϕ_TS_b (B3LYP/6-31+G*)

C	0.9063200	-1.4901480	-0.0009660
C	-0.4863630	-1.3432930	-0.0160840
P	-1.1690700	0.2599810	0.0472640
C	0.1500930	1.4057680	-0.0005470
C	1.4484970	0.8838410	-0.0001110
C	1.8535750	-0.4627340	0.0122740
C	3.3039680	-0.7951350	0.0372420
O	-2.1814490	0.5658950	1.3059730
C	-3.5699960	0.6127690	0.9488760
C	-3.6487200	0.6283650	-0.6050670
O	-2.3087880	0.5131990	-1.1081030
C	3.7977680	-1.8302470	0.8535470
C	5.1566330	-2.1510620	0.8719620
C	6.0614380	-1.4369570	0.0814050
C	5.5890930	-0.4021750	-0.7311060
C	4.2284220	-0.0899730	-0.7562490
C	-0.0920980	2.8792970	-0.0112610
C	-1.3968410	-2.4999420	-0.0944860
C	-0.9540930	-3.8050670	-0.4051180
C	-1.8699560	-4.8494720	-0.4404970
C	-3.2188230	-4.5901900	-0.1803630
C	-3.5759770	-3.2710810	0.0929260
N	-2.7068210	-2.2557170	0.1375930
C	0.9764950	3.8031910	-0.0438290
C	0.7600500	5.1802170	-0.0585690
C	-0.5363070	5.7004990	-0.0428240
C	-1.6095360	4.8097900	-0.0122640
C	-1.3896780	3.4320650	0.0031430
H	1.2924230	-2.5055910	-0.0153640

H	2.2573570	1.6061320	0.0202990
H	3.1128610	-2.3710220	1.5023520
H	5.5106000	-2.9512730	1.5178020
H	7.1201070	-1.6828130	0.0988220
H	6.2793560	0.1556730	-1.3596100
H	3.8733190	0.6936230	-1.4212350
H	0.0875430	-3.9998030	-0.6338200
H	-1.5353650	-5.8562410	-0.6784200
H	-3.9670490	-5.3767420	-0.1984380
H	-4.6166020	-3.0132760	0.2891130
H	2.0043570	3.4598140	-0.0589760
H	1.6167580	5.8494910	-0.0830610
H	-0.7042150	6.7740370	-0.0545880
H	-2.6305830	5.1839280	0.0000000
H	-2.2555230	2.7861680	0.0259160
H	-4.0606380	-0.2699430	1.3631170
H	-3.9908200	1.5191920	1.3952140
H	-4.2265680	-0.2174180	-0.9836940
H	-4.0653900	1.5636520	-0.9926000

6_a (B3LYP/6-31+G*)

C	1.2117970	1.1910150	-0.1255130
C	1.3855670	-0.1973060	-0.1598650
P	0.0000560	-1.2140460	-0.0000380
C	-1.3855250	-0.1974260	0.1599050
C	-1.2118850	1.1909080	0.1255480
C	-0.0000750	1.8895580	0.0000070
C	-0.0001430	3.3777580	0.0000020
O	0.1088660	-2.3549670	1.2025700
C	0.3060160	-3.6892390	0.7040410
C	-0.3057080	-3.6892000	-0.7043700

O	-0.1086950	-2.3548550	-1.2027650
C	0.9829060	4.1045100	0.6973720
C	0.9864180	5.5007700	0.6933740
C	-0.0003190	6.2074980	-0.0000530
C	-0.9869890	5.5006000	-0.6934770
C	-0.9833090	4.1043760	-0.6974250
C	-2.7374230	-0.8059280	0.2929080
C	2.7375220	-0.8056840	-0.2928240
C	3.0663720	-1.6954380	-1.3345540
C	4.3402150	-2.2564500	-1.4470580
C	5.3323250	-1.9359440	-0.5163000
C	5.0424380	-1.0553310	0.5282270
C	3.7645950	-0.5174710	0.6184540
C	-3.0661710	-1.6956850	1.3346660
C	-4.3399660	-2.2568000	1.4472100
C	-5.3321230	-1.9363950	0.5164670
C	-5.0423350	-1.0557860	-0.5280890
C	-3.7645360	-0.5178240	-0.6183590
H	2.1185090	1.7816380	-0.2392410
H	-2.1186480	1.7814460	0.2393120
H	1.7373980	3.5697480	1.2690760
H	1.7548610	6.0370160	1.2453710
H	-0.0004220	7.2945740	-0.0000950
H	-1.7554680	6.0367840	-1.2454840
H	-1.7377100	3.5694500	-1.2690930
H	2.3037160	-1.9286000	-2.0711450
H	4.5602770	-2.9312630	-2.2699390
H	6.3281020	-2.3624150	-0.6013770
H	5.7843990	-0.7842400	1.2730510
F	3.5108980	0.3256140	1.6556320
H	-2.3034740	-1.9287780	2.0712360

H	-4.5599550	-2.9316140	2.2701080
H	-6.3278620	-2.3629470	0.6015790
H	-5.7843370	-0.7847740	-1.2729010
F	-3.5109290	0.3252580	-1.6555590
H	-0.1946630	-4.3842820	1.3831980
H	1.3800850	-3.9053290	0.6830440
H	-1.3797560	-3.9053930	-0.6833980
H	0.1950380	-4.3841270	-1.3835950

6_a (B3LYP/6-31+G*, excited state minima)

C	1.2447460	1.1864900	-0.1357220
C	1.4351020	-0.1993160	-0.1711700
P	-0.0000610	-1.2025890	-0.0000070
C	-1.4351270	-0.1991810	0.1711580
C	-1.2446430	1.1866070	0.1357060
C	0.0000840	1.8721600	-0.0000100
C	0.0001590	3.3323680	-0.0000030
O	0.1209260	-2.3098930	1.2069210
C	0.3129800	-3.6518200	0.7007950
C	-0.3133600	-3.6517850	-0.7008130
O	-0.1211590	-2.3098780	-1.2069380
C	1.1456690	4.0727790	0.4002850
C	1.1404410	5.4623710	0.4056270
C	0.0003150	6.1687140	0.0000080
C	-1.1398880	5.4625000	-0.4056200
C	-1.1452690	4.0729090	-0.4002900
C	-2.7520060	-0.8337450	0.2730900
C	2.7519260	-0.8339980	-0.2730860
C	3.0249360	-1.9459450	-1.1246790
C	4.2835090	-2.5369390	-1.1814320
C	5.3426710	-2.0491750	-0.4030230

C	5.1174930	-0.9413410	0.4336840
C	3.8603590	-0.3706070	0.4782010
C	-3.0251030	-1.9456630	1.1246930
C	-4.2837250	-2.5365510	1.1814590
C	-5.3428520	-2.0487040	0.4030540
C	-5.1175860	-0.9408980	-0.4336660
C	-3.8604050	-0.3702690	-0.4781970
H	2.1326550	1.7876960	-0.2893040
H	-2.1324940	1.7878940	0.2892980
H	2.0270260	3.5479840	0.7528040
H	2.0256930	6.0005630	0.7337790
H	0.0003750	7.2553480	0.0000120
H	-2.0250800	6.0007930	-0.7337690
H	-2.0266820	3.5482160	-0.7528210
H	2.2368020	-2.3048430	-1.7780560
H	4.4474770	-3.3734630	-1.8567110
H	6.3262910	-2.5067340	-0.4483240
H	5.9037030	-0.5261660	1.0571680
F	3.6817380	0.6845730	1.3295010
H	-2.2369920	-2.3046260	1.7780620
H	-4.4477570	-3.3730570	1.8567450
H	-6.3265090	-2.5061820	0.4483640
H	-5.9037650	-0.5256640	-1.0571500
F	-3.6817000	0.6848840	-1.3295130
H	-0.1893730	-4.3370200	1.3878690
H	1.3858910	-3.8638770	0.6747830
H	-1.3862960	-3.8637210	-0.6748010
H	0.1889150	-4.3370400	-1.3878870

6_b (B3LYP/6-31+G*)

C	-1.2144360	1.1973800	0.0993310

C	-1.3883020	-0.1906830	0.1310350
P	-0.0000100	-1.2074410	0.0000040
C	1.3883030	-0.1907080	-0.1310230
C	1.2144620	1.1973500	-0.0993290
C	0.0000180	1.8963230	0.0000060
C	0.0000560	3.3841810	-0.0000160
O	-0.0898170	-2.3480130	-1.2042920
C	-0.2956450	-3.6821100	-0.7085630
C	0.2954700	-3.6821440	0.7085440
O	0.0897910	-2.3480260	1.2042840
C	-0.8483310	4.1123120	0.8550800
C	-0.8530250	5.5084390	0.8518600
C	0.0001100	6.2156880	0.0000140
C	0.8532040	5.5084480	-0.8518140
C	0.8484550	4.1122730	-0.8550530
C	2.7429850	-0.8034440	-0.2093620
C	-2.7429960	-0.8033850	0.2093510
C	-3.1094570	-1.6851760	1.2450990
C	-4.3837500	-2.2528760	1.3101160
C	-5.3374010	-1.9472680	0.3351270
C	-5.0087730	-1.0760130	-0.7057460
C	-3.7310960	-0.5313240	-0.7485520
C	3.1094490	-1.6851220	-1.2452030
C	4.3837310	-2.2528430	-1.3102460
C	5.3373540	-1.9473750	-0.3351840
C	5.0087190	-1.0762340	0.7057810
C	3.7310540	-0.5315130	0.7486060
H	-2.1275230	1.7874690	0.1457110
H	2.1275530	1.7874330	-0.1457310
H	-1.4939370	3.5789370	1.5485650
H	-1.5156840	6.0441410	1.5276010

H	0.0001170	7.3027310	0.0000410
H	1.5159150	6.0440540	-1.5275750
H	1.4940850	3.5789480	-1.5485560
H	-2.3759770	-1.9072320	2.0142260
H	-4.6342710	-2.9214560	2.1293630
H	-6.3334950	-2.3788720	0.3825670
H	-5.7202190	-0.8175480	-1.4840680
F	-3.4371870	0.2996460	-1.7833390
H	2.3759810	-1.9070710	-2.0143710
H	4.6342690	-2.9213320	-2.1295610
H	6.3334390	-2.3789980	-0.3826410
H	5.7201500	-0.8178810	1.4841540
F	3.4371270	0.2993510	1.7834690
H	0.2141850	-4.3775950	-1.3803870
H	-1.3701180	-3.8970580	-0.7030300
H	1.3699190	-3.8972170	0.7030130
H	-0.2144410	-4.3775740	1.3803640

6_c (B3LYP/6-31+G*)

C	-1.2138660	1.2014450	-0.1029450
C	-1.3854950	-0.1867000	-0.1349960
P	0.0003920	-1.2058390	-0.0000220
C	1.3856090	-0.1858020	0.1350070
C	1.2130960	1.2022280	0.1028980
C	-0.0006110	1.9016150	-0.0000430
C	-0.0010820	3.3895350	-0.0000800
O	0.1748150	-2.3469450	-1.1947670
C	-0.1245040	-3.6830410	-0.7573580
C	0.1271860	-3.6829390	0.7572720
O	-0.1732900	-2.3471050	1.1946770
C	-0.9681350	4.1159590	0.7196950

C	-0.9722530	5.5122140	0.7158840
C	-0.0019840	6.2193100	-0.0000790
C	0.9687550	5.5128330	-0.7160080
C	0.9655280	4.1165600	-0.7198120
C	2.7372660	-0.7936540	0.2790360
C	-2.7367730	-0.7953980	-0.2789310
C	-3.0770370	-1.6148940	-1.3720520
C	-4.3480200	-2.1796800	-1.4987220
C	-5.3239550	-1.9312730	-0.5293820
C	-5.0222450	-1.1185910	0.5655510
C	-3.7470220	-0.5761420	0.6678740
C	3.0781010	-1.6125380	1.3724360
C	4.3494220	-2.1765470	1.4991940
C	5.3251070	-1.9279640	0.5296490
C	5.0228250	-1.1158760	-0.5655670
C	3.7472870	-0.5741770	-0.6679560
H	-2.1237770	1.7902420	-0.2025760
H	2.1226270	1.7916210	0.2025120
H	-1.7088460	3.5808180	1.3088150
H	-1.7278940	6.0481630	1.2855400
H	-0.0023160	7.3064000	-0.0000880
H	1.7240740	6.0492290	-1.2856630
H	1.7066140	3.5819070	-1.3089050
H	-2.3275150	-1.7858390	-2.1398170
H	-4.5792200	-2.7996920	-2.3607280
H	-6.3174670	-2.3610010	-0.6239790
H	-5.7528950	-0.9038510	1.3393760
F	-3.4789250	0.2015880	1.7504810
H	2.3287530	-1.7836300	2.1403400
H	4.5810660	-2.7960990	2.3614120
H	6.3188700	-2.3571010	0.6243010

H	5.7532730	-0.9010250	-1.3395520
F	3.4786210	0.2029730	-1.7508330
H	0.5316740	-4.3713740	-1.2961070
H	-1.1705350	-3.9089350	-0.9935020
H	1.1734050	-3.9079410	0.9934310
H	-0.5284070	-4.3718370	1.2960130

6_d (B3LYP/6-31+G*)

C	1.2040670	1.2050700	-0.2137500
C	1.3983030	-0.1806360	-0.1867140
P	0.0169950	-1.2147330	-0.1246650
C	-1.3888770	-0.2170250	-0.1864560
C	-1.2322090	1.1731370	-0.2122220
C	-0.0233280	1.8878280	-0.2312010
C	-0.0451400	3.3749320	-0.2758010
O	-0.0091680	-2.2727230	1.1509170
C	0.2942730	-3.6255900	0.7753960
C	-0.1369830	-3.7416890	-0.6932680
O	0.0724480	-2.4340300	-1.2571330
C	0.8430470	4.0932110	-1.0977800
C	0.8244010	5.4889180	-1.1351930
C	-0.0911720	6.2036450	-0.3576680
C	-0.9832680	5.5053570	0.4612380
C	-0.9566010	4.1101220	0.5051280
C	-2.7393890	-0.8437960	-0.1842760
C	2.7637860	-0.7707920	-0.1175660
C	3.2369860	-1.6882470	-1.0762010
C	4.5207210	-2.2329130	-1.0011990
C	5.3773000	-1.8672950	0.0410700
C	4.9419100	-0.9592860	1.0085290
C	3.6570990	-0.4386160	0.9124100

C	-3.1571810	-1.7266110	-1.1987230
C	-4.4269740	-2.3077670	-1.1897250
C	-5.3239020	-2.0135130	-0.1587950
C	-4.9444400	-1.1383080	0.8611310
C	-3.6722840	-0.5801310	0.8286430
H	2.1098980	1.8080890	-0.2064900
H	-2.1540910	1.7498900	-0.2544170
H	1.5396530	3.5516270	-1.7331990
H	1.5187700	6.0178370	-1.7839260
H	-0.1088110	7.2901290	-0.3889300
H	-1.6940650	6.0478830	1.0802010
H	-1.6337330	3.5833070	1.1730270
H	2.5802850	-1.9576870	-1.8977250
H	4.8549340	-2.9308750	-1.7641570
H	6.3801850	-2.2810280	0.1024920
H	5.5754550	-0.6540980	1.8357150
F	3.2582840	0.4288170	1.8793010
H	-2.4699060	-1.9352190	-2.0137260
H	-4.7195540	-2.9771540	-1.9942480
H	-6.3161820	-2.4561660	-0.1475910
H	-5.6121530	-0.8870090	1.6795060
F	-3.3279300	0.2596040	1.8407400
H	-0.2641380	-4.2949970	1.4347620
H	1.3694050	-3.7980990	0.8994990
H	-1.1978000	-4.0007690	-0.7848670
H	0.4666210	-4.4564630	-1.2586200

6_e (B3LYP/6-31+G*)

C	1.2642290	1.1271330	-0.2333660
C	1.3712060	-0.2681940	-0.2124040
P	-0.0719960	-1.2127760	-0.1277440

C	-1.4119040	-0.1270080	-0.1573390
C	-1.1684140	1.2505580	-0.1860490
C	0.0828880	1.8866890	-0.2279530
C	0.1589550	3.3721690	-0.2741070
O	-0.1502550	-2.2733210	1.1434230
C	0.0759070	-3.6385850	0.7589170
C	-0.3772140	-3.7253710	-0.7052710
O	-0.1091130	-2.4272220	-1.2661620
C	1.1098110	4.0738690	0.4903370
C	1.1867530	5.4671760	0.4441070
C	0.3073070	6.1971240	-0.3607900
C	-0.6468160	5.5159090	-1.1218890
C	-0.7159770	4.1218970	-1.0821850
C	-2.7973210	-0.6698050	-0.0964520
C	2.6996340	-0.9403690	-0.2010950
C	3.0792400	-1.8811960	-1.1788420
C	4.3305360	-2.5007380	-1.1559920
C	5.2484200	-2.1901110	-0.1487470
C	4.9071680	-1.2601100	0.8355600
C	3.6528010	-0.6641270	0.7908850
C	-3.3133080	-1.5133040	-1.0987180
C	-4.6141010	-2.0177280	-1.0363210
C	-5.4435660	-1.6820840	0.0376080
C	-4.9655940	-0.8439580	1.0471710
C	-3.6644530	-0.3631160	0.9618350
H	2.2053090	1.6704800	-0.2896760
H	-2.0518360	1.8858090	-0.1619480
H	1.7784430	3.5228870	1.1471330
H	1.9271870	5.9838470	1.0503470
H	0.3642610	7.2821690	-0.3938880
H	-1.3320020	6.0695490	-1.7597040

H	-1.4425650	3.6056400	-1.7050020
H	2.3757590	-2.1083030	-1.9738320
H	4.5922220	-3.2143710	-1.9326370
H	6.2266080	-2.6627780	-0.1279630
H	5.5906910	-0.9947610	1.6362600
F	3.3467560	0.2261580	1.7724030
H	-2.6772890	-1.7536640	-1.9462080
H	-4.9828230	-2.6592060	-1.8322530
H	-6.4592980	-2.0644490	0.0906040
H	-5.5787760	-0.5632610	1.8980350
F	-3.2227290	0.4386230	1.9653620
H	-0.5098070	-4.2801360	1.4222350
H	1.1416800	-3.8686040	0.8698880
H	-1.4503590	-3.9315700	-0.7864900
H	0.1841120	-4.4666280	-1.2800940

6_f (B3LYP/6-31+G*)

C	1.4846690	0.8781350	-0.1912480
C	1.2839870	-0.5052250	-0.1658800
P	-0.3279960	-1.1248660	-0.0761830
C	-1.4014710	0.2305560	-0.0098460
C	-0.8584680	1.5211140	-0.0665490
C	0.4963250	1.8753480	-0.1660280
C	0.8839810	3.3099820	-0.2302270
O	-0.6539710	-2.1667900	1.1706430
C	-0.7492490	-3.5395250	0.7531900
C	-1.1642460	-3.4836340	-0.7241480
O	-0.5618120	-2.2835820	-1.2373330
C	2.0033970	3.7935880	0.4734830
C	2.3670210	5.1401360	0.4136930
C	1.6143810	6.0428640	-0.3433620

C	0.4971350	5.5802540	-1.0442350
C	0.1409970	4.2310590	-0.9923860
C	-2.8587200	0.0678980	0.2580480
C	2.4392600	-1.4456830	-0.1597820
C	2.6173460	-2.4392750	-1.1424960
C	3.7129300	-3.3049060	-1.1214140
C	4.6729170	-3.1975950	-0.1112000
C	4.5304290	-2.2220370	0.8777630
C	3.4275350	-1.3782060	0.8342250
C	-3.4406420	0.6596910	1.3965920
C	-4.8085220	0.5718070	1.6571830
C	-5.6439020	-0.1276690	0.7813360
C	-5.1025120	-0.7292740	-0.3561640
C	-3.7370890	-0.6168800	-0.5900880
H	2.5204700	1.2024920	-0.2673470
H	-1.5810760	2.3329800	0.0010360
H	2.5797210	3.1122140	1.0945210
H	3.2332050	5.4868860	0.9725060
H	1.8949830	7.0921560	-0.3865000
H	-0.0924990	6.2680960	-1.6458890
H	-0.7107430	3.8813530	-1.5707320
H	1.8834070	-2.5112620	-1.9387410
H	3.8224180	-4.0529220	-1.9020750
H	5.5314420	-3.8634150	-0.0921570
H	5.2526600	-2.1082380	1.6803910
F	3.3124660	-0.4470330	1.8198360
H	-2.7895780	1.1872010	2.0882520
H	-5.2184630	1.0408540	2.5473890
H	-6.7097930	-0.2049250	0.9774790
H	-5.7188960	-1.2689570	-1.0687410
F	-3.2455820	-1.1968610	-1.7215530

H	-1.4914910	-4.0344650	1.3848140
H	0.2266530	-4.0205180	0.8848290
H	-2.2503250	-3.4156540	-0.8459310
H	-0.7827910	-4.3280040	-1.3041280

6_g (B3LYP/6-31+G*)

C	1.2157760	1.2072230	0.0768500
C	1.3876990	-0.1807100	0.1053390
P	-0.0003410	-1.1999920	-0.0000580
C	-1.3877970	-0.1799060	-0.1053810
C	-1.2151010	1.2079210	-0.0768140
C	0.0005410	1.9077770	0.0000380
C	0.0009620	3.3956020	0.0000720
O	-0.1548980	-2.3406690	1.1974370
C	0.1364390	-3.6769070	0.7551510
C	-0.1387720	-3.6767300	-0.7555100
O	0.1535620	-2.3406620	-1.1976450
C	0.8620560	4.1234540	0.8425540
C	0.8670610	5.5196100	0.8392820
C	0.0017220	6.2270900	0.0000740
C	-0.8640220	5.5200540	-0.8391610
C	-0.8597750	4.1239340	-0.8424330
C	-2.7431150	-0.7913070	-0.1930420
C	2.7426640	-0.7928710	0.1929920
C	3.1264710	-1.6007570	1.2801540
C	4.3990720	-2.1709810	1.3578650
C	5.3320570	-1.9394780	0.3430520
C	4.9860620	-1.1391300	-0.7478390
C	3.7099330	-0.5912230	-0.8012350
C	-3.1276200	-1.5983810	-1.2805580
C	-4.4005530	-2.1678680	-1.3582740

C	-5.3331610	-1.9364140	-0.3431040
C	-4.9864760	-1.1368380	0.7481340
C	-3.7100360	-0.5896510	0.8015180
H	2.1310040	1.7954220	0.1091880
H	-2.1299980	1.7966380	-0.1091150
H	1.5177620	3.5898220	1.5262970
H	1.5397630	6.0551570	1.5051730
H	0.0019770	7.3141020	0.0000450
H	-1.5364040	6.0560220	-1.5050330
H	-1.5157480	3.5906040	-1.5261530
H	2.4100110	-1.7590500	2.0816470
H	4.6650560	-2.7822350	2.2160800
H	6.3267170	-2.3733830	0.3988120
H	5.6827430	-0.9382680	-1.5559480
F	3.3966970	0.1719080	-1.8807700
H	-2.4114420	-1.7566250	-2.0823140
H	-4.6670820	-2.7785070	-2.2167580
H	-6.3280700	-2.3697470	-0.3988600
H	-5.6828620	-0.9360290	1.5565110
F	-3.3961230	0.1727400	1.8813720
H	-0.5117940	-4.3647910	1.3040230
H	1.1858140	-3.9037230	0.9749840
H	-1.1883090	-3.9027600	-0.9753730
H	0.5089580	-4.3650350	-1.3044500

6_h (B3LYP/6-31+G*)

C	1.0044580	1.4376660	-0.0452150
C	1.4170730	0.0976540	-0.0429040
P	0.2069510	-1.1413700	-0.0489270
C	-1.3283130	-0.3549270	-0.1710620
C	-1.3934420	1.0397760	-0.1396750

C	-0.3093820	1.9309090	-0.0719040
C	-0.5499690	3.3983550	-0.0444020
O	0.1411220	-2.2548220	1.1711520
C	0.6672190	-3.5394100	0.8040200
C	0.4895360	-3.6287040	-0.7201020
O	0.5864290	-2.2753730	-1.2000300
C	0.2163520	4.2439760	0.7798870
C	-0.0015410	5.6229210	0.8028790
C	-1.0016160	6.1931760	0.0103080
C	-1.7762420	5.3674100	-0.8095210
C	-1.5489180	3.9902840	-0.8402950
C	-2.5629210	-1.1839610	-0.2497580
C	2.8695980	-0.2093330	-0.1674250
C	3.6208020	0.3323120	-1.2294380
C	4.9925440	0.1091740	-1.3531600
C	5.6607730	-0.6808830	-0.4130730
C	4.9490150	-1.2363380	0.6517230
C	3.5853250	-0.9881730	0.7492450
C	-2.8002790	-2.0679470	-1.3192380
C	-3.9606570	-2.8420220	-1.3893010
C	-4.9270730	-2.7453500	-0.3839950
C	-4.7261350	-1.8746880	0.6892180
C	-3.5584740	-1.1226070	0.7354290
H	1.8095250	2.1708690	-0.0510300
H	-2.3943490	1.4666490	-0.1576010
H	0.9730850	3.8123740	1.4303370
H	0.6020800	6.2505960	1.4543240
H	-1.1756780	7.2659500	0.0317820
H	-2.5520840	5.7972560	-1.4388420
H	-2.1383020	3.3680990	-1.5092960
H	3.0997060	0.9302020	-1.9720550

H	5.5357070	0.5437350	-2.1878320
H	6.7279930	-0.8637790	-0.5031520
H	5.4324930	-1.8429550	1.4114420
F	2.9243750	-1.5259160	1.8139290
H	-2.0616010	-2.1239510	-2.1144710
H	-4.1150670	-3.5078410	-2.2341580
H	-5.8357080	-3.3392030	-0.4333740
H	-5.4522110	-1.7742680	1.4901650
F	-3.3847610	-0.2947850	1.7984030
H	0.0958640	-4.3021220	1.3393270
H	1.7192730	-3.5920410	1.1015610
H	-0.4924890	-4.0321050	-0.9909950
H	1.2741530	-4.2146770	-1.2056640

6_i (B3LYP/6-31+G*)

C	1.5005830	0.8621750	-0.1542030
C	1.2824890	-0.5184440	-0.1294580
P	-0.3378610	-1.1174530	-0.0688700
C	-1.3947120	0.2505360	-0.0246860
C	-0.8365790	1.5349380	-0.0714870
C	0.5243560	1.8720750	-0.1479710
C	0.9351930	3.3004560	-0.2165470
O	-0.6895610	-2.1586460	1.1719210
C	-0.8077450	-3.5277310	0.7482130
C	-1.2120360	-3.4572570	-0.7312090
O	-0.5785440	-2.2696890	-1.2354200
C	1.9821920	3.7149480	-1.0611430
C	2.3754640	5.0532430	-1.1228940
C	1.7239810	6.0173540	-0.3487110
C	0.6786620	5.6242000	0.4919790
C	0.2944680	4.2838770	0.5605990

C	-2.8641840	0.0995720	0.1777440
C	2.4222960	-1.4764100	-0.0749120
C	2.6219770	-2.4708920	-1.0523380
C	3.6998510	-3.3565730	-0.9891070
C	4.6195730	-3.2680780	0.0596840
C	4.4539090	-2.2927730	1.0452840
C	3.3686470	-1.4290770	0.9600200
C	-3.4821440	0.6491760	1.3180760
C	-4.8601970	0.5699700	1.5220020
C	-5.6691450	-0.0779550	0.5838500
C	-5.0917140	-0.6368550	-0.5576550
C	-3.7165920	-0.5347740	-0.7340180
H	2.5427920	1.1750390	-0.1658260
H	-1.5545120	2.3534690	-0.0667800
H	2.4779390	2.9839020	-1.6951030
H	3.1846480	5.3443280	-1.7886460
H	2.0265710	7.0601530	-0.3993960
H	0.1686830	6.3607320	1.1086170
H	-0.4973810	3.9922760	1.2462060
H	1.9195090	-2.5281710	-1.8779170
H	3.8273930	-4.1053430	-1.7662970
H	5.4645930	-3.9492440	0.1120060
H	5.1441760	-2.1943870	1.8775190
F	3.2273000	-0.5012130	1.9436500
H	-2.8510270	1.1356040	2.0568130
H	-5.2990220	1.0052890	2.4155720
H	-6.7428970	-0.1478030	0.7348050
H	-5.6870170	-1.1353510	-1.3166380
F	-3.1890460	-1.0727380	-1.8687210
H	-1.5628220	-4.0115920	1.3731750
H	0.1584130	-4.0270480	0.8832770

H	-2.2958940	-3.3640080	-0.8589000
H	-0.8472410	-4.3080320	-1.3124480

6_j (B3LYP/6-31+G*)

C	-1.2166340	1.3053630	-0.0096720
C	-1.3941250	-0.0829550	-0.0143140
P	0.0013260	-1.1138810	-0.0003560
C	1.3942880	-0.0795940	0.0142290
C	1.2135490	1.3082720	0.0096010
C	-0.0023940	2.0097890	-0.0000750
C	-0.0041900	3.4965760	-0.0001570
O	0.2481370	-2.2450560	-1.1811990
C	-0.0836530	-3.5801810	-0.7641080
C	0.0934020	-3.5802480	0.7621430
O	-0.2424960	-2.2463320	1.1798360
C	-0.9127710	4.2238020	-0.7924520
C	-0.9187700	5.6200130	-0.7887420
C	-0.0075660	6.3284340	-0.0002440
C	0.9053590	5.6221990	0.7883470
C	0.9026920	4.2260470	0.7921560
C	2.7745470	-0.6258690	-0.1148830
C	-2.7730920	-0.6323560	0.1152680
C	-3.6026390	-0.2283850	1.1801600
C	-4.9160820	-0.6816380	1.3064580
C	-5.4432210	-1.5716550	0.3661160
C	-4.6499070	-1.9965140	-0.7012390
C	-3.3483450	-1.5203990	-0.8010010
C	3.6034900	-0.2202900	-1.1796270
C	4.9179800	-0.6706420	-1.3054240
C	5.4468020	-1.5592900	-0.3647280
C	4.6541100	-1.9857080	0.7024680

C	3.3514650	-1.5124500	0.8017540
H	-2.1333360	1.8926180	0.0148710
H	2.1288630	1.8976940	-0.0148280
H	-1.6050940	3.6893280	-1.4383570
H	-1.6285100	6.1549550	-1.4155420
H	-0.0088120	7.4154120	-0.0002500
H	1.6137550	6.1589860	1.4150890
H	1.5962390	3.6931830	1.4380740
H	-3.1890080	0.4465410	1.9244340
H	-5.5224990	-0.3476130	2.1438810
H	-6.4636600	-1.9332090	0.4582250
H	-5.0249940	-2.6756290	-1.4608900
F	-2.6076650	-1.9382640	-1.8675540
H	3.1885740	0.4535720	-1.9241510
H	5.5239150	-0.3354440	-2.1427260
H	6.4680670	-1.9186020	-0.4564560
H	5.0304690	-2.6638590	1.4623510
F	2.6113260	-1.9318510	1.8680680
H	0.5987520	-4.2680520	-1.2700050
H	-1.1146260	-3.7979980	-1.0605780
H	1.1250420	-3.7950340	1.0585300
H	-0.5868720	-4.2704290	1.2677570

6_k (B3LYP/6-31+G*)

C	0.9561810	1.4735660	-0.0301130
C	1.4172420	0.1492860	-0.0201480
P	0.2552740	-1.1338720	-0.0491150
C	-1.3053230	-0.4065880	-0.2041180
C	-1.4228010	0.9849430	-0.1712110
C	-0.3748980	1.9162590	-0.0802940
C	-0.6790530	3.3723570	-0.0497350

O	0.2158620	-2.2456680	1.1740120
C	0.8088050	-3.5047510	0.8208810
C	0.6529500	-3.6104100	-0.7043160
O	0.6953760	-2.2565130	-1.1902480
C	0.1058030	4.2998860	-0.7603090
C	-0.1792360	5.6661200	-0.7248380
C	-1.2657950	6.1416580	0.0148850
C	-2.0591200	5.2337870	0.7220950
C	-1.7661110	3.8689310	0.6942020
C	-2.5046490	-1.2787340	-0.3373470
C	2.8839650	-0.1061880	-0.0855540
C	3.6491280	0.4313050	-1.1394970
C	5.0315200	0.2572510	-1.2129550
C	5.6970820	-0.4777300	-0.2274110
C	4.9717980	-1.0278470	0.8309450
C	3.5970480	-0.8296650	0.8775700
C	-2.6680940	-2.1671940	-1.4171250
C	-3.7973490	-2.9804480	-1.5349680
C	-4.8062640	-2.9207260	-0.5693610
C	-4.6794470	-2.0463690	0.5121570
C	-3.5416020	-1.2544050	0.6057620
H	1.7324940	2.2348650	0.0269230
H	-2.4346230	1.3763770	-0.2563500
H	0.9353300	3.9449320	-1.3670770
H	0.4418360	6.3588370	-1.2880670
H	-1.4907900	7.2048330	0.0401330
H	-2.9019040	5.5892300	1.3103890
H	-2.3726800	3.1804530	1.2774000
H	3.1301370	0.9855570	-1.9166930
H	5.5854740	0.6866930	-2.0431990
H	6.7728260	-0.6219830	-0.2774620

H	5.4524390	-1.5923000	1.6242100
F	2.9226310	-1.3617410	1.9357060
H	-1.8972050	-2.1949540	-2.1825600
H	-3.8947480	-3.6482750	-2.3867010
H	-5.6908830	-3.5456790	-0.6563770
H	-5.4404690	-1.9732270	1.2830550
F	-3.4412970	-0.4213520	1.6754820
H	0.2702690	-4.2918160	1.3548960
H	1.8590260	-3.5038370	1.1300280
H	-0.3068280	-4.0587350	-0.9844450
H	1.4686030	-4.1633610	-1.1773620

6_I (B3LYP/6-31+G*)

C	-1.2161710	1.3037390	-0.0240430
C	-1.3929160	-0.0849420	-0.0358200
P	0.0009330	-1.1154040	-0.0004730
C	1.3930250	-0.0825790	0.0357130
C	1.2140040	1.3057710	0.0239510
C	-0.0016830	2.0076280	-0.0001150
C	-0.0029040	3.4954830	-0.0002260
O	0.2559070	-2.2476200	-1.1793500
C	-0.0821890	-3.5815530	-0.7647030
C	0.0892050	-3.5818920	0.7619140
O	-0.2518820	-2.2490170	1.1775130
C	-0.9191310	4.2233910	0.7820560
C	-0.9246850	5.6195830	0.7788290
C	-0.0053000	6.3274910	-0.0004630
C	0.9152450	5.6210230	-0.7796600
C	0.9120430	4.2247840	-0.7826490
C	2.7758690	-0.6338270	-0.0335380
C	-2.7748630	-0.6383340	0.0340540

C	-3.6334590	-0.2741510	1.0899890
C	-4.9498810	-0.7305070	1.1629260
C	-5.4501730	-1.5837820	0.1750450
C	-4.6274740	-1.9692420	-0.8849630
C	-3.3233840	-1.4911430	-0.9309240
C	3.6340880	-0.2691830	-1.0896070
C	4.9512160	-0.7236020	-1.1619320
C	5.4526150	-1.5753070	-0.1732560
C	4.6303230	-1.9611410	0.8869330
C	3.3254960	-1.4849850	0.9322770
H	-2.1326120	1.8907460	-0.0581300
H	2.1294510	1.8943140	0.0582960
H	-1.6194350	3.6891680	1.4194990
H	-1.6407720	6.1549310	1.3979230
H	-0.0061900	7.4145760	-0.0005650
H	1.6304310	6.1573650	-1.3989260
H	1.6132680	3.6917010	-1.4200380
H	-3.2399080	0.3715330	1.8702430
H	-5.5797180	-0.4279700	1.9950240
H	-6.4728820	-1.9472950	0.2246930
H	-4.9815690	-2.6188720	-1.6796540
F	-2.5533160	-1.8702930	-1.9900180
H	3.2396910	0.3752790	-1.8704460
H	5.5807530	-0.4207840	-1.9941540
H	6.4758830	-1.9373100	-0.2224250
H	4.9852770	-2.6095690	1.6822220
F	2.5558240	-1.8643960	1.9915410
H	0.5991020	-4.2719590	-1.2686310
H	-1.1130790	-3.7966930	-1.0647170
H	1.1205750	-3.7949590	1.0617800
H	-0.5905230	-4.2741790	1.2653710

6_ω_TS_a (B3LYP/6-31+G*)

C	-1.5382830	0.6690680	-0.0644320
C	-1.2536890	-0.7144620	-0.0241240
P	0.4328770	-1.1486030	-0.0770390
C	1.3734640	0.2988520	-0.1751220
C	0.7391720	1.5376390	-0.1688370
C	-0.6486540	1.7485590	-0.1269070
C	-1.1821640	3.1385790	-0.1513920
O	0.9025530	-2.2229870	-1.2576650
C	1.4976520	-3.4320940	-0.7631660
C	1.7951990	-3.1889070	0.7327770
O	0.8890840	-2.1585900	1.1553030
C	-2.3143090	3.4727880	-0.9173400
C	-2.8158820	4.7756910	-0.9342340
C	-2.1910580	5.7826040	-0.1930190
C	-1.0622360	5.4686870	0.5692380
C	-0.5678600	4.1630910	0.5929900
C	2.8596220	0.1936230	-0.2287300
C	-2.3153700	-1.7547540	0.0722420
C	-2.0026200	-3.1351350	0.0456600
C	-2.9550290	-4.1468690	0.1290150
C	-4.3092400	-3.8302700	0.2462070
C	-4.6737530	-2.4867070	0.2796020
C	-3.6990590	-1.5003170	0.1951550
C	3.5366460	-0.4045490	-1.3092660
C	4.9304380	-0.4821370	-1.3492430
C	5.6915270	0.0456590	-0.3019850
C	5.0534670	0.6503750	0.7829840
C	3.6647430	0.7086520	0.7976010
H	-2.5841740	0.9321580	-0.0157010

H	1.3857260	2.4102610	-0.2380890
H	-2.7942660	2.7083090	-1.5232130
H	-3.6901660	5.0060300	-1.5385990
H	-2.5789000	6.7979860	-0.2087050
H	-0.5716030	6.2389720	1.1597390
H	0.2903690	3.9290730	1.2182790
H	-0.9689200	-3.4339450	-0.0380840
H	-2.6326940	-5.1843510	0.1024540
H	-5.0654840	-4.6069010	0.3124450
H	-5.7093510	-2.1743250	0.3727770
F	-4.1793220	-0.2213760	0.2437440
H	2.9453930	-0.7952700	-2.1318300
H	5.4205930	-0.9408980	-2.2037340
H	6.7766220	-0.0048520	-0.3289180
H	5.6103010	1.0721220	1.6141140
F	3.0715240	1.2953780	1.8703130
H	2.4073790	-3.6267400	-1.3378700
H	0.7944410	-4.2578200	-0.9195230
H	2.8236650	-2.8467780	0.8907570
H	1.6086600	-4.0734030	1.3476320

6_ω_TS_b (B3LYP/6-31+G*)

C	-0.7684490	1.5708530	0.0280740
C	-1.3755650	0.3140020	0.0818870
P	-0.3930550	-1.1014220	-0.0011030
C	1.2480510	-0.5877290	-0.1570600
C	1.5280480	0.7847690	-0.1709500
C	0.6064470	1.8403000	-0.0951410
C	1.0842540	3.2484770	-0.1402130
O	-0.8169670	-2.2229140	-1.1474660
C	-1.4779440	-3.3737340	-0.5944690

C	-0.9601900	-3.4830860	0.8471050
O	-0.7092730	-2.1303430	1.2618180
C	0.3918840	4.2291960	-0.8750880
C	0.8359100	5.5525440	-0.9114900
C	1.9916870	5.9286450	-0.2212240
C	2.6940890	4.9659350	0.5095830
C	2.2430160	3.6453750	0.5536840
C	2.3399570	-1.5973760	-0.2202660
C	-2.8499140	0.1868190	0.2876930
C	-3.4261050	0.3613350	1.5589200
C	-4.8054470	0.2743910	1.7569760
C	-5.6492910	0.0015520	0.6760270
C	-5.1107230	-0.1778680	-0.5996610
C	-3.7330020	-0.0809730	-0.7651400
C	2.3828480	-2.6050770	-1.2042550
C	3.4157990	-3.5436630	-1.2503040
C	4.4480580	-3.4979980	-0.3091330
C	4.4396800	-2.5109180	0.6788050
C	3.3960530	-1.5942780	0.7037270
H	-1.4418560	2.4223670	0.1142470
H	2.5780320	1.0477280	-0.2811150
H	-0.4897200	3.9462070	-1.4451270
H	0.2842420	6.2879440	-1.4925500
H	2.3402640	6.9578280	-0.2521890
H	3.5894620	5.2454900	1.0599870
H	2.7817660	2.9165990	1.1542440
H	-2.7647810	0.5645410	2.3967550
H	-5.2187640	0.4165740	2.7516720
H	-6.7240640	-0.0682590	0.8207500
H	-5.7364850	-0.3805410	-1.4635100
F	-3.2356200	-0.2446930	-2.0191830

H	1.5936610	-2.6294890	-1.9491250
H	3.4203340	-4.3006200	-2.0300090
H	5.2588950	-4.2205640	-0.3428420
H	5.2209400	-2.4437390	1.4297510
F	3.4109940	-0.6552740	1.6887420
H	-1.2177520	-4.2411570	-1.2065520
H	-2.5616590	-3.2136300	-0.6292340
H	-0.0247500	-4.0515510	0.9024570
H	-1.6952960	-3.9176120	1.5295790

6_ω_TS_c (B3LYP/6-31+G*)

C	-1.4914670	0.9488510	-0.0536490
C	-1.4000950	-0.4636680	-0.0205730
P	0.2163630	-1.1125910	-0.0855430
C	1.3279870	0.2217130	-0.1857270
C	0.8687350	1.5284450	-0.1727180
C	-0.4851540	1.9126320	-0.1221570
C	-0.8440330	3.3567480	-0.1386990
O	0.5547580	-2.2031470	-1.2785590
C	1.0572310	-3.4665410	-0.8122330
C	1.3018680	-3.3211100	0.7127110
O	0.6229690	-2.1309620	1.1412380
C	-1.9240210	3.8335170	-0.9052120
C	-2.2640620	5.1879040	-0.9110050
C	-1.5239120	6.1046680	-0.1591590
C	-0.4426150	5.6492700	0.6008790
C	-0.1104820	4.2932790	0.6138310
C	2.7877910	-0.0781270	-0.2358910
C	-2.6583460	-1.2653000	0.0858470
C	-3.9350790	-0.6495120	0.1362940
C	-5.1259600	-1.3643990	0.2365200

C	-5.1168980	-2.7597150	0.2932260
C	-3.8886640	-3.4135700	0.2468690
C	-2.7227180	-2.6669690	0.1463320
C	3.3840960	-0.7595060	-1.3142610
C	4.7557240	-1.0200970	-1.3489840
C	5.5754570	-0.5994580	-0.2975820
C	5.0182530	0.0828180	0.7859330
C	3.6495750	0.3247300	0.7945530
H	-2.4918010	1.3577710	0.0100610
H	1.6203530	2.3127390	-0.2378720
H	-2.4860780	3.1394840	-1.5257030
H	-3.0999500	5.5290150	-1.5174240
H	-1.7848800	7.1598570	-0.1670310
H	0.1376350	6.3493610	1.1973960
H	0.7129630	3.9509400	1.2358560
H	-4.0155720	0.4287980	0.0993890
H	-6.0657010	-0.8199990	0.2713100
H	-6.0400400	-3.3262060	0.3722270
H	-3.8083640	-4.4955490	0.2867070
F	-1.5651100	-3.3924870	0.1035150
H	2.7496660	-1.0686010	-2.1393330
H	5.1844180	-1.5391830	-2.2020170
H	6.6442820	-0.7940870	-0.3196170
H	5.6221410	0.4254100	1.6206040
F	3.1353720	0.9853470	1.8654110
H	1.9857240	-3.6769880	-1.3507000
H	0.3176920	-4.2355810	-1.0426700
H	2.3663580	-3.2084550	0.9422150
H	0.8922720	-4.1623470	1.2771050

6_θ_TS_a (B3LYP/6-31+G*)

C	-1.2136610	-1.1979060	-0.1177210
C	-1.3873590	0.1925690	-0.1507520
P	0.0000000	1.2071610	0.0000000
C	1.3873530	0.1926240	0.1507500
C	1.2137080	-1.1978590	0.1177190
C	0.0000000	-1.8889220	0.0000000
C	0.0000000	-3.3883630	0.0000000
O	-0.1028620	2.3496920	1.2031320
C	-0.3028750	3.6833980	0.7055260
C	0.3026990	3.6834110	-0.7055570
O	0.1027720	2.3496870	-1.2031510
C	0.0962300	-4.1075990	-1.2022460
C	0.0961070	-5.5055110	-1.2040480
C	0.0001220	-6.2087880	0.0000000
C	-0.0958910	-5.5055150	1.2040440
C	-0.0960690	-4.1076030	1.2022420
C	2.7399160	0.8042060	0.2608080
C	-2.7399460	0.8041010	-0.2608050
C	-3.0847610	1.6876520	-1.3026110
C	-4.3582130	2.2533240	-1.3952930
C	-5.3336820	1.9435880	-0.4434950
C	-5.0273350	1.0700850	0.6022990
C	-3.7500060	0.5276820	0.6727690
C	3.0846890	1.6877770	1.3026120
C	4.3581200	2.2534980	1.3953020
C	5.3336080	1.9437920	0.4435130
C	5.0273030	1.0702720	-0.6022780
C	3.7499950	0.5278210	-0.6727560
H	-2.1205100	-1.7945040	-0.2011540
H	2.1205790	-1.7944230	0.2011560
H	0.1736470	-3.5633010	-2.1404990

H	0.1723350	-6.0440820	-2.1457260
H	0.0001440	-7.2960690	0.0000000
H	-0.1720980	-6.0440890	2.1457220
H	-0.1735070	-3.5633080	2.1404960
H	-2.3344370	1.9129360	-2.0542660
H	-4.5910290	2.9234420	-2.2185290
H	-6.3292430	2.3734710	-0.5128120
H	-5.7560110	0.8080300	1.3633100
F	-3.4788560	-0.3057440	1.7124930
H	2.3343510	1.9130360	2.0542590
H	4.5909040	2.9236290	2.2185360
H	6.3291520	2.3737140	0.5128360
H	5.7559950	0.8082410	-1.3632820
F	3.4788860	-0.3056230	-1.7124770
H	0.2002970	4.3790850	1.3822620
H	-1.3771390	3.8992600	0.6888610
H	1.3769480	3.8993430	-0.6888940
H	-0.2005200	4.3790590	-1.3822990

6_θ_TS_b (B3LYP/6-31+G*)

C	0.2583880	1.1829800	-1.1838720
C	0.3134650	1.3517560	0.2029870
P	0.0000000	0.0000000	1.2257860
C	-0.3134650	-1.3517560	0.2029870
C	-0.2583880	-1.1829800	-1.1838720
C	0.0000000	0.0000000	-1.9013160
C	0.0000000	0.0000000	-3.3982300
O	-1.1823970	0.2458840	2.3648310
C	-0.6639520	0.3854000	3.6992370
C	0.6639520	-0.3854000	3.6992370
O	1.1823970	-0.2458840	2.3648310

C	0.2653790	1.1678460	-4.1446400
C	0.2656100	1.1696690	-5.5398540
C	0.0000000	0.0000000	-6.2543050
C	-0.2656100	-1.1696690	-5.5398540
C	-0.2653790	-1.1678460	-4.1446400
C	-0.5864910	-2.6846130	0.8088590
C	0.5864910	2.6846130	0.8088590
C	1.6679590	2.9049240	1.6843220
C	1.9140440	4.1593020	2.2466520
C	1.0809200	5.2393930	1.9421020
C	0.0000000	5.0575310	1.0766560
C	-0.2240750	3.7968040	0.5369430
C	-1.6679590	-2.9049240	1.6843220
C	-1.9140440	-4.1593020	2.2466520
C	-1.0809200	-5.2393930	1.9421020
C	0.0000000	-5.0575310	1.0766560
C	0.2240750	-3.7968040	0.5369430
H	0.4513590	2.0908360	-1.7455200
H	-0.4513590	-2.0908360	-1.7455200
H	0.4789050	2.1061270	-3.6448750
H	0.4761730	2.0967940	-6.0679440
H	0.0000000	0.0000000	-7.3410800
H	-0.4761730	-2.0967940	-6.0679440
H	-0.4789050	-2.1061270	-3.6448750
H	2.3279140	2.0717020	1.9056340
H	2.7639120	4.2945230	2.9102200
H	1.2698300	6.2202450	2.3698340
H	-0.6720220	5.8703790	0.8189210
F	-1.2930540	3.6490650	-0.2902330
H	-2.3279140	-2.0717020	1.9056340
H	-2.7639120	-4.2945230	2.9102200

H	-1.2698300	-6.2202450	2.3698340
H	0.6720220	-5.8703790	0.8189210
F	1.2930540	-3.6490650	-0.2902330
H	-1.3967020	-0.0325320	4.3944150
H	-0.5186060	1.4500210	3.9139990
H	0.5186060	-1.4500210	3.9139990
H	1.3967020	0.0325320	4.3944150

7_a (B3LYP/6-31+G*)

C	1.8049860	-1.2173480	-0.0403660
C	0.4155520	-1.3996450	-0.0541260
P	-0.5873820	0.0000000	0.0000000
C	0.4155540	1.3996460	0.0540240
C	1.8049860	1.2173280	0.0403530
C	2.5038960	0.0000000	0.0000000
C	3.9921170	0.0000000	0.0000000
O	-1.7595580	-0.1147490	1.2110810
C	-3.0405980	-0.0673450	0.6942300
C	-3.0406670	0.0673380	-0.6939480
O	-1.7596790	0.1147090	-1.2109330
C	4.7192730	-0.8807350	-0.8223700
C	6.1155020	-0.8847200	-0.8192690
C	6.8223090	0.0000000	0.0001600
C	6.1154920	0.8846780	0.8195230
C	4.7192200	0.8807080	0.8225470
C	-0.1798620	2.7652870	0.0303440
C	-0.1798860	-2.7652820	-0.0305550
C	0.2814650	-3.7145830	0.9005170
C	-0.2415170	-5.0082680	0.9252220
C	-1.2486680	-5.3782200	0.0289600
C	-1.7216370	-4.4435520	-0.8954840

C	-1.1906640	-3.1519570	-0.9292860
C	-1.1907670	3.1520020	0.9289150
C	-1.7217210	4.4436000	0.8949990
C	-1.2486100	5.3782380	-0.0294030
C	-0.2413320	5.0082480	-0.9255060
C	0.2816320	3.7145580	-0.9006880
C	-4.2184030	-0.1370440	1.4180540
C	-5.4217260	-0.0676860	0.6960440
C	-5.4217940	0.0677520	-0.6955240
C	-4.2185420	0.1370750	-1.4176550
H	2.3940260	-2.1329100	-0.0513420
H	2.3940430	2.1328790	0.0513400
H	4.1854650	-1.5526050	-1.4900900
H	6.6514300	-1.5721950	-1.4694260
H	7.9092950	0.0000000	0.0001810
H	6.6513200	1.5721820	1.4697270
H	4.1854380	1.5526220	1.4902460
H	1.0434830	-3.4266590	1.6201790
H	0.1300840	-5.7238570	1.6546450
H	-1.6606450	-6.3838110	0.0519170
H	-2.4999700	-4.7209600	-1.6020510
H	-1.5460710	-2.4459550	-1.6741850
H	-1.5462880	2.4460340	1.6737920
H	-2.5001550	4.7210340	1.6014450
H	-1.6605740	6.3838320	-0.0524500
H	0.1303830	5.7238110	-1.6548970
H	1.0437460	3.4266080	-1.6202390
H	-4.2019000	-0.2451650	2.4978650
H	-6.3648810	-0.1209460	1.2323400
H	-6.3650010	0.1210400	-1.2317260
H	-4.2021440	0.2451980	-2.4974670

7_a (B3LYP/6-31+G*, excited state minima)

C	-1.7920760	-1.2184540	0.2560620
C	-0.3967400	-1.4337000	0.1401450
P	0.5968210	0.0000490	-0.0000200
C	-0.3968710	1.4337170	-0.1402040
C	-1.7921880	1.2183380	-0.2561160
C	-2.4775220	-0.0000920	-0.0000310
C	-3.9443420	-0.0001760	0.0000010
O	1.7434560	-0.2553580	-1.1979740
C	3.0255520	-0.1418400	-0.6833960
C	3.0255240	0.1420050	0.6834430
O	1.7434070	0.2555060	1.1979720
C	-4.6834890	-0.9154960	0.7923400
C	-6.0744980	-0.9089420	0.7972100
C	-6.7816980	-0.0003960	0.0000550
C	-6.0746700	0.9082510	-0.7971370
C	-4.6836620	0.9150180	-0.7923260
C	0.1786150	2.7736410	-0.0602170
C	0.1789210	-2.7735480	0.0601820
C	-0.6042730	-3.8555330	-0.4276320
C	-0.0951350	-5.1480700	-0.4878140
C	1.2145300	-5.4218630	-0.0697120
C	2.0049930	-4.3703570	0.4116060
C	1.5004790	-3.0754910	0.4816450
C	1.5000940	3.0758280	-0.4817610
C	2.0043910	4.3707780	-0.4117130
C	1.2137780	5.4221340	0.0696860
C	-0.0958180	5.1481060	0.4878460
C	-0.6047400	3.8554850	0.4276510
C	4.2037080	-0.2776080	-1.3983810

C	5.4055320	-0.1348500	-0.6868470
C	5.4055050	0.1350560	0.6869800
C	4.2036520	0.2777940	1.3984700
H	-2.3878200	-2.0992150	0.4656970
H	-2.3880100	2.0990530	-0.4657160
H	-4.1575290	-1.6096560	1.4395560
H	-6.6116580	-1.6114170	1.4291750
H	-7.8683830	-0.0004810	0.0000760
H	-6.6119640	1.6106350	-1.4290890
H	-4.1578400	1.6092380	-1.4395870
H	-1.6053630	-3.6632930	-0.8019220
H	-0.7190940	-5.9482930	-0.8794470
H	1.6095900	-6.4330040	-0.1170640
H	3.0201450	-4.5631400	0.7504590
H	2.1269640	-2.3012810	0.9111670
H	2.1266810	2.3017550	-0.9113690
H	3.0194860	4.5637460	-0.7506290
H	1.6086680	6.4333420	0.1170420
H	-0.7198970	5.9482130	0.8795280
H	-1.6057840	3.6630670	0.8019730
H	4.1859710	-0.4958330	-2.4612330
H	6.3487410	-0.2410340	-1.2148740
H	6.3486920	0.2412590	1.2150420
H	4.1858720	0.4960220	2.4613210

7_b (B3LYP/6-31+G*)

C	1.8045310	-1.2151110	-0.0562520
C	0.4152910	-1.4017620	-0.0445600
P	-0.5903890	-0.0032170	-0.0591250
C	0.4086090	1.3996890	-0.0767550
C	1.7990600	1.2209640	-0.0815970

C	2.5004070	0.0041880	-0.0828140
C	3.9883320	0.0057050	-0.1242890
O	-1.7386000	0.0043790	1.1733090
C	-3.0288150	0.0005290	0.6812600
C	-3.0587750	-0.0102750	-0.7129800
O	-1.7873760	-0.0152040	-1.2594060
C	4.6935860	-0.9005880	-0.9376640
C	6.0894980	-0.9039020	-0.9729390
C	6.8173160	0.0064610	-0.2016250
C	6.1322400	0.9165860	0.6084610
C	4.7367340	0.9121540	0.6495240
C	-0.1877320	2.7645010	-0.0194190
C	-0.1731680	-2.7655920	0.0744110
C	0.3154800	-3.6568420	1.0481320
C	-0.1982310	-4.9496410	1.1602630
C	-1.2232170	-5.3765890	0.3107720
C	-1.7238560	-4.4995080	-0.6545680
C	-1.2026180	-3.2093040	-0.7756290
C	0.2649800	3.6837640	0.9453960
C	-0.2538440	4.9780760	1.0034530
C	-1.2484660	5.3784640	0.1063910
C	-1.7133830	4.4736900	-0.8513190
C	-1.1867870	3.1816200	-0.9176640
C	-4.1911230	0.0062600	1.4326990
C	-5.4093370	0.0006950	0.7329970
C	-5.4396050	-0.0102410	-0.6646650
C	-4.2516590	-0.0160020	-1.4152650
H	2.3963150	-2.1287230	-0.0337450
H	2.3849680	2.1374400	-0.1258340
H	4.1419860	-1.5930210	-1.5687830
H	6.6083140	-1.6111860	-1.6156660

H	7.9038810	0.0071340	-0.2317160
H	6.6850840	1.6246930	1.2212480
H	4.2204650	1.6055700	1.3089790
H	1.0918900	-3.3238030	1.7320320
H	0.1949350	-5.6194840	1.9211370
H	-1.6278110	-6.3813320	0.4016470
H	-2.5168520	-4.8213910	-1.3250720
H	-1.5807970	-2.5485790	-1.5497260
H	1.0174700	3.3710520	1.6647910
H	0.1111500	5.6700680	1.7585320
H	-1.6571430	6.3845200	0.1543920
H	-2.4819990	4.7753920	-1.5585780
H	-1.5361300	2.4980460	-1.6853860
H	-4.1510740	0.0144900	2.5171160
H	-6.3407810	0.0049420	1.2918900
H	-6.3940120	-0.0143710	-1.1834030
H	-4.2589000	-0.0245340	-2.5007120

7_c (B3LYP/6-31+G*)

C	1.7989990	1.2210490	-0.0816030
C	0.4085330	1.3996930	-0.0767530
P	-0.5903870	-0.0032640	-0.0591250
C	0.4153720	-1.4017580	-0.0445620
C	1.8045970	-1.2150250	-0.0562620
C	2.5004080	0.0043130	-0.0828160
C	3.9883290	0.0058800	-0.1243240
O	-1.7873800	-0.0153050	-1.2593940
C	-3.0587770	-0.0103780	-0.7129580
C	-3.0288050	0.0004070	0.6812820
O	-1.7385890	0.0042370	1.1733230
C	4.7366960	0.9123660	0.6495260

C	6.1321770	0.9168550	0.6084820
C	6.8173120	0.0067470	-0.2016070
C	6.0895550	-0.9036590	-0.9728960
C	4.6936160	-0.9004000	-0.9376380
C	-0.1730140	-2.7656200	0.0743920
C	-0.1878800	2.7644750	-0.0194310
C	-1.1869500	3.1815380	-0.9176850
C	-1.7136160	4.4735790	-0.8513480
C	-1.2487580	5.3783810	0.1063640
C	-0.2541250	4.9780490	1.0034380
C	0.2647700	3.6837640	0.9453890
C	-1.2024840	-3.2093570	-0.7756100
C	-1.7236560	-4.4995880	-0.6545580
C	-1.2229330	-5.3766720	0.3107360
C	-0.1979310	-4.9496960	1.1601950
C	0.3157140	-3.6568710	1.0480740
C	-4.2516640	-0.0160860	-1.4152360
C	-5.4396060	-0.0103300	-0.6646300
C	-5.4093280	0.0005870	0.7330320
C	-4.1911110	0.0061360	1.4327270
H	2.3848460	2.1375620	-0.1258600
H	2.3964410	-2.1286000	-0.0337880
H	4.2203560	1.6057350	1.3089740
H	6.6850300	1.6249650	1.2212610
H	7.9038790	0.0074610	-0.2316840
H	6.6083580	-1.6109630	-1.6156050
H	4.1420860	-1.5929010	-1.5687470
H	-1.5362470	2.4979440	-1.6854100
H	-2.4822410	4.7752380	-1.5586160
H	-1.6574910	6.3844150	0.1543600
H	0.1108240	5.6700610	1.7585210

H	1.0172660	3.3710940	1.6647950
H	-1.5807320	-2.5486300	-1.5496710
H	-2.5166680	-4.8214900	-1.3250340
H	-1.6274770	-6.3814350	0.4016040
H	0.1952970	-5.6195390	1.9210360
H	1.0921340	-3.3238110	1.7319520
H	-4.2589100	-0.0246010	-2.5006840
H	-6.3940150	-0.0144460	-1.1833610
H	-6.3407690	0.0048320	1.2919320
H	-4.1510560	0.0143530	2.5171440

7_d (B3LYP/6-31+G*)

C	1.8053330	1.2165900	-0.0643430
C	0.4154830	1.3978690	-0.0841390
P	-0.5871500	0.0000000	0.0000000
C	0.4153720	-1.3978410	0.0841050
C	1.8052310	-1.2166840	0.0643150
C	2.5040100	0.0000000	0.0000000
C	3.9927160	-0.0001450	0.0000000
O	-1.7596440	-0.1299160	-1.2093360
C	-3.0407100	-0.0758390	-0.6931800
C	-3.0406870	0.0759830	0.6932380
O	-1.7596080	0.1301070	1.2093480
C	4.7199890	0.9332010	0.7616400
C	6.1161780	0.9369790	0.7588440
C	6.8228380	-0.0002810	0.0000000
C	6.1161140	-0.9374810	-0.7587660
C	4.7199120	-0.9335700	-0.7616040
C	-0.1809970	-2.7630870	0.1222420
C	-0.1807610	2.7631670	-0.1222910
C	-1.1637520	3.1216010	-1.0625430

C	-1.6941620	4.4136960	-1.0844470
C	-1.2481610	5.3764370	-0.1755010
C	-0.2687790	5.0346430	0.7617430
C	0.2534270	3.7407610	0.7922060
C	0.2530160	-3.7406770	-0.7923400
C	-0.2693140	-5.0345100	-0.7618880
C	-1.2486460	-5.3762480	0.1754290
C	-1.6944760	-4.4135060	1.0844580
C	-1.1639420	-3.1214620	1.0625640
C	-4.2185480	-0.1547860	-1.4159350
C	-5.4218490	-0.0764540	-0.6947970
C	-5.4218270	0.0765150	0.6949340
C	-4.2185050	0.1548880	1.4160320
H	2.3942290	2.1290530	-0.1413120
H	2.3940550	-2.1291970	0.1412560
H	4.1857690	1.6482320	1.3824360
H	6.6523530	1.6660860	1.3617240
H	7.9098090	-0.0003450	0.0000000
H	6.6522090	-1.6666580	-1.3616270
H	4.1856580	-1.6485780	-1.3823980
H	-1.4970360	2.3923240	-1.7949990
H	-2.4504070	4.6695670	-1.8224260
H	-1.6594920	6.3823460	-0.1962840
H	0.0814240	5.7726870	1.4792880
H	0.9937830	3.4744250	1.5421930
H	0.9933380	-3.4743740	-1.5423720
H	0.0807530	-5.7725570	-1.4794950
H	-1.6600730	-6.3821190	0.1962030
H	-2.4506810	-4.6693390	1.8224910
H	-1.4970940	-2.3921770	1.7950740
H	-4.2021980	-0.2763560	-2.4943220

H	-6.3650260	-0.1364330	-1.2303440
H	-6.3649880	0.1364610	1.2305140
H	-4.2021240	0.2764570	2.4944190

7_ω_TS_a (B3LYP/6-31+G*)

C	-1.7466770	-1.2995030	-0.0154080
C	-0.3522660	-1.3970710	-0.0065960
P	0.5787460	0.0472190	-0.0272810
C	-0.4939890	1.3952010	-0.0438180
C	-1.8728550	1.1339070	-0.0437600
C	-2.5084900	-0.1172950	-0.0371580
C	-3.9945410	-0.1937640	-0.0619800
O	1.7606690	0.0373060	-1.2376260
C	3.0372680	0.0759060	-0.7071670
C	3.0232450	0.1387700	0.6861330
O	1.7379230	0.1449810	1.1927590
C	-4.6882420	-1.1279850	0.7297180
C	-6.0820210	-1.2040330	0.7027660
C	-6.8208230	-0.3400840	-0.1105160
C	-6.1478680	0.5969620	-0.8994910
C	-4.7534430	0.6652190	-0.8786730
C	0.0231180	2.7901460	0.0264520
C	0.3384940	-2.7261490	0.0095930
C	0.6742120	-3.3739330	-1.1905800
C	1.2929590	-4.6265860	-1.1751850
C	1.5907710	-5.2474790	0.0411480
C	1.2679490	-4.6090600	1.2417630
C	0.6482120	-3.3566910	1.2258630
C	1.0445080	3.2480450	-0.8260100
C	1.5018400	4.5656280	-0.7514570
C	0.9440930	5.4565900	0.1688290

C	-0.0733000	5.0156630	1.0203420
C	-0.5229630	3.6960890	0.9550940
C	4.2221340	0.0606450	-1.4225800
C	5.4182040	0.1103470	-0.6869180
C	5.4040220	0.1730260	0.7097010
C	4.1937830	0.1877930	1.4230790
H	-2.2824460	-2.2473460	-0.0320550
H	-2.5106670	2.0162840	-0.0398650
H	-4.1310510	-1.7862140	1.3918650
H	-6.5918190	-1.9321480	1.3292900
H	-7.9061220	-0.3965300	-0.1294810
H	-6.7083260	1.2691050	-1.5449990
H	-4.2445940	1.3771590	-1.5239400
H	0.4450860	-2.8898650	-2.1363060
H	1.5424800	-5.1161370	-2.1133100
H	2.0727400	-6.2218290	0.0532380
H	1.4984500	-5.0844850	2.1919500
H	0.3994250	-2.8592290	2.1597740
H	1.4664050	2.5758380	-1.5672170
H	2.2898080	4.8973670	-1.4231010
H	1.2987940	6.4825780	0.2235290
H	-0.5102130	5.6962120	1.7471210
H	-1.2930000	3.3546850	1.6420410
H	4.2166680	0.0093280	-2.5067410
H	6.3666140	0.0990300	-1.2164610
H	6.3416680	0.2110460	1.2568580
H	4.1661580	0.2367420	2.5068630

7_ω_TS_b (B3LYP/6-31+G*)

C	1.8947390	1.0910860	-0.0434750
C	0.5263710	1.4245170	-0.0253150

P	-0.5784750	0.0908720	-0.0363910
C	0.3050690	-1.3902720	-0.0519410
C	1.7001450	-1.3375680	-0.0512270
C	2.4899950	-0.1761660	-0.0548080
C	3.9742020	-0.2935210	-0.0713350
O	-1.7609720	0.1789160	-1.2445280
C	-3.0369670	0.1560290	-0.7134430
C	-3.0235050	0.1086750	0.6813080
O	-1.7394120	0.1019240	1.1882940
C	4.7777380	0.5323470	0.7367100
C	6.1697500	0.4249450	0.7195840
C	6.7943670	-0.5198860	-0.0994120
C	6.0106040	-1.3523140	-0.9034740
C	4.6190600	-1.2371100	-0.8921620
C	-0.4112590	-2.6959850	0.0304010
C	0.0648540	2.8435210	0.0010560
C	0.9837270	3.9148200	0.0384460
C	0.5611710	5.2431400	0.0628970
C	-0.7994510	5.5592970	0.0542960
C	-1.7268530	4.5174380	0.0211570
C	-1.3023540	3.1885300	-0.0050060
C	-1.4168100	-3.0556440	-0.8847760
C	-2.0511460	-4.2972690	-0.7970310
C	-1.6889880	-5.2069470	0.1995600
C	-0.6882960	-4.8633480	1.1133360
C	-0.0614960	-3.6188510	1.0336750
C	-4.2223170	0.1828300	-1.4289100
C	-5.4182960	0.1567630	-0.6924070
C	-5.4045910	0.1084490	0.7051850
C	-4.1950970	0.0858540	1.4189270
H	2.5854730	1.9266840	-0.0745510

H	2.2107770	-2.2989150	-0.0372650
H	4.3054870	1.2475260	1.4059990
H	6.7661250	1.0715240	1.3589860
H	7.8777870	-0.6068340	-0.1104270
H	6.4831950	-2.0857640	-1.5524650
H	4.0252230	-1.8698960	-1.5471720
H	2.0514130	3.7283840	0.0500190
H	1.3060530	6.0347620	0.0900890
H	-1.1282240	6.5948470	0.0738000
H	-2.7926400	4.7318170	0.0148220
H	-2.0679870	2.4239340	-0.0324520
H	-1.6862760	-2.3700740	-1.6824230
H	-2.8232130	-4.5562810	-1.5173180
H	-2.1816630	-6.1736790	0.2644560
H	-0.4020800	-5.5601710	1.8973240
H	0.6975910	-3.3476340	1.7628410
H	-4.2160730	0.2247690	-2.5134690
H	-6.3667340	0.1755640	-1.2216320
H	-6.3426870	0.0889110	1.2524840
H	-4.1675560	0.0505600	2.5031960

7_θ_TS_a (B3LYP/6-31+G*)

C	1.8067720	-1.2178220	-0.0568050
C	0.4149250	-1.3998920	-0.0739730
P	-0.5855760	0.0000000	0.0000000
C	0.4150600	1.3997940	0.0738010
C	1.8068890	1.2175770	0.0566240
C	2.4979610	-0.0001550	0.0000000
C	3.9973700	-0.0002070	0.0000000
O	-1.7598670	-0.1278740	1.2096130
C	-3.0404230	-0.0747800	0.6934300

C	-3.0404640	0.0750170	-0.6933620
O	-1.7599360	0.1279450	-1.2096410
C	4.7165740	0.0875890	-1.2028200
C	6.1144200	0.0878120	-1.2044670
C	6.8175840	0.0000000	0.0001800
C	6.1142760	-0.0880310	1.2046650
C	4.7164430	-0.0879160	1.2028490
C	-0.1800770	2.7654870	0.0851890
C	-0.1803380	-2.7655340	-0.0853090
C	0.2800700	-3.7365640	0.8236940
C	-0.2421300	-5.0307240	0.8176290
C	-1.2477030	-5.3802750	-0.0885270
C	-1.7194540	-4.4245070	-0.9917700
C	-1.1890990	-3.1322280	-0.9947930
C	-1.1887820	3.1322310	0.9947140
C	-1.7190260	4.4245550	0.9917620
C	-1.2472180	5.3803240	0.0885510
C	-0.2416990	5.0307230	-0.8176470
C	0.2803890	3.7365180	-0.8237840
C	-4.2184320	-0.1522570	1.4162180
C	-5.4218470	-0.0750460	0.6951090
C	-5.4218880	0.0755580	-0.6948780
C	-4.2185120	0.1526340	-1.4160690
H	2.4020820	-2.1286930	-0.1051890
H	2.4023000	2.1283820	0.1050220
H	4.1728430	0.1537560	-2.1423540
H	6.6531150	0.1562120	-2.1465790
H	7.9047300	-0.0002190	0.0001400
H	6.6528300	-0.1561480	2.1468810
H	4.1726020	-0.1540220	2.1423240
H	1.0414800	-3.4652830	1.5504230

H	0.1290670	-5.7630530	1.5304910
H	-1.6590780	-6.3863870	-0.0897980
H	-2.4962570	-4.6858820	-1.7061200
H	-1.5432610	-2.4096380	-1.7240900
H	-1.5429840	2.4096380	1.7239870
H	-2.4957910	4.6859610	1.7061430
H	-1.6585060	6.3864710	0.0898780
H	0.1295450	5.7630490	-1.5304870
H	1.0417530	3.4652130	-1.5505540
H	-4.2020220	-0.2720880	2.4948140
H	-6.3650010	-0.1340830	1.2308350
H	-6.3650720	0.1347030	-1.2305380
H	-4.2021610	0.2724610	-2.4946670

7_θ_TS_b (B3LYP/6-31+G*)

C	1.7936040	-1.2093100	-0.0537420
C	0.4052540	-1.3916860	-0.0684900
P	-0.6037910	0.0000000	0.0000000
C	0.4052430	1.3917130	0.0684740
C	1.7935970	1.2093570	0.0537500
C	2.5112060	0.0000000	0.0000000
C	4.0085330	0.0000000	0.0000000
O	-1.7740440	-0.1200230	1.2104870
C	-3.0555520	-0.0702200	0.6937980
C	-3.0555760	0.0701850	-0.6936950
O	-1.7740860	0.1200310	-1.2104250
C	4.7545370	-1.1972720	-0.0314820
C	6.1497490	-1.1991220	-0.0315920
C	6.8639340	0.0000000	0.0000000
C	6.1497620	1.1991370	0.0316350
C	4.7545410	1.1972980	0.0315620

C	-0.1851010	2.7604610	0.0742480
C	-0.1850690	-2.7604410	-0.0743170
C	0.2556280	-3.7163790	0.8597990
C	-0.2627110	-5.0122860	0.8590000
C	-1.2445900	-5.3771140	-0.0668630
C	-1.6974450	-4.4355280	-0.9944470
C	-1.1710330	-3.1416060	-1.0023100
C	-1.1710690	3.1416500	1.0022270
C	-1.6974970	4.4355660	0.9943120
C	-1.2446550	5.3771170	0.0666870
C	-0.2627710	5.0122660	-0.8591610
C	0.2555870	3.7163660	-0.8599050
C	-4.2332130	-0.1432730	1.4173930
C	-5.4364360	-0.0707980	0.6955750
C	-5.4364590	0.0706810	-0.6954000
C	-4.2332610	0.1431960	-1.4172550
H	2.3531150	-2.1382010	-0.1015640
H	2.3530970	2.1382580	0.1015340
H	4.2548320	-2.1592540	-0.0571580
H	6.6780270	-2.1493180	-0.0567220
H	7.9506020	0.0000000	-0.0002520
H	6.6780740	2.1493050	0.0570160
H	4.2548420	2.1592780	0.0573830
H	0.9967560	-3.4312070	1.6021550
H	0.0920820	-5.7334070	1.5913320
H	-1.6530130	-6.3844100	-0.0641970
H	-2.4560120	-4.7094580	-1.7234850
H	-1.5098300	-2.4290380	-1.7487100
H	-1.5098570	2.4291090	1.7486580
H	-2.4560660	4.7095170	1.7233400
H	-1.6530920	6.3844080	0.0639790

H	0.0920120	5.7333630	-1.5915210
H	0.9967240	3.4311700	-1.6022430
H	-4.2167270	-0.2559690	2.4967260
H	-6.3796190	-0.1263770	1.2315540
H	-6.3796600	0.1262250	-1.2313510
H	-4.2168110	0.2558910	-2.4965880

8_a (B3LYP/6-31+G*)

C	-1.8160030	-1.2838060	-0.0188080
C	-0.4193130	-1.4432470	-0.0128650
P	0.5778200	-0.0243470	-0.0252120
C	-0.4720300	1.3537720	-0.0541540
C	-1.8516920	1.1500750	-0.0512940
C	-2.5321470	-0.0845150	-0.0382640
C	-4.0201290	-0.1065830	-0.0503660
O	1.7421550	0.0100540	-1.2363000
C	3.0160550	0.0246930	-0.6982440
C	2.9978900	0.0921850	0.6949450
O	1.7126670	0.1273020	1.1987370
C	-4.7413160	-0.9994840	0.7640530
C	-6.1372540	-1.0267910	0.7460250
C	-6.8495800	-0.1530470	-0.0803370
C	-6.1487690	0.7450610	-0.8902710
C	-4.7526890	0.7648850	-0.8777120
C	0.0925220	2.7323560	0.0114880
C	0.2471390	-2.7584250	-0.0029710
C	-0.4359280	-3.9889160	-0.0778020
C	0.2962200	-5.1719620	-0.0564910
C	1.6894860	-5.1179600	0.0347640
C	2.2845930	-3.8572140	0.0978980
N	1.5939640	-2.7146480	0.0812740

C	1.0829850	3.1760100	-0.8834080
C	1.5767280	4.4807290	-0.8129710
C	1.0881070	5.3711520	0.1463930
C	0.1026320	4.9436120	1.0409700
C	-0.3844480	3.6371710	0.9782220
C	4.2020800	-0.0135390	-1.4091110
C	5.3970830	0.0212870	-0.6695980
C	5.3789460	0.0884720	0.7265000
C	4.1658020	0.1226190	1.4354610
H	-2.4037990	-2.1978950	-0.0297980
H	-2.4591450	2.0536470	-0.0473010
H	-4.2030180	-1.6608580	1.4388440
H	-6.6689380	-1.7227080	1.3907190
H	-7.9363140	-0.1708120	-0.0919120
H	-6.6890960	1.4250450	-1.5446210
H	-4.2227240	1.4477350	-1.5373260
H	-1.5168630	-4.0273960	-0.1571470
H	-0.2178390	-6.1283140	-0.1137750
H	2.2950960	-6.0190190	0.0532710
H	3.3664090	-3.7543910	0.1651030
H	1.4530120	2.5025300	-1.6501610
H	2.3396410	4.8027420	-1.5174640
H	1.4722050	6.3866900	0.1982370
H	-0.2801210	5.6238410	1.7979520
H	-1.1300250	3.3042910	1.6957590
H	4.1991580	-0.0711310	-2.4930120
H	6.3470430	-0.0045060	-1.1961180
H	6.3151400	0.1155090	1.2770650
H	4.1347320	0.1723500	2.5191420

8_a (B3LYP/6-31+G*, excited state minima)

C	1.7682060	-1.3543950	-0.0735960
C	0.3716290	-1.4738010	-0.0509320
P	-0.5624100	0.0228170	0.0417910
C	0.5248570	1.3946070	0.0646910
C	1.9151030	1.1242870	0.1219200
C	2.5259600	-0.1452150	0.0451900
C	3.9884640	-0.2380770	0.0871640
O	-1.6603130	0.0060420	1.2977050
C	-2.9545210	0.0389870	0.7954690
C	-2.9801520	0.1665750	-0.5930490
O	-1.7063980	0.2345930	-1.1398200
C	4.6794220	-1.3116700	-0.5322260
C	6.0683430	-1.3903930	-0.5050380
C	6.8204100	-0.4110110	0.1539140
C	6.1612640	0.6542230	0.7816160
C	4.7741780	0.7427200	0.7455860
C	0.0141180	2.7621000	-0.0557440
C	-0.3651910	-2.7216020	-0.0826300
C	0.2555380	-4.0008230	-0.0905260
C	-0.5381800	-5.1361530	-0.1384690
C	-1.9372240	-5.0053290	-0.1754360
C	-2.4662980	-3.7067010	-0.1562280
N	-1.7270870	-2.6031770	-0.1136690
C	-1.2663210	3.1527610	0.4128640
C	-1.7122970	4.4652700	0.2919280
C	-0.9019060	5.4447850	-0.2950600
C	0.3669820	5.0826850	-0.7642910
C	0.8180020	3.7715960	-0.6497940
C	-4.1167930	-0.0127720	1.5430730
C	-5.3330270	0.0598940	0.8427460
C	-5.3580480	0.1820670	-0.5504090

C	-4.1683860	0.2380530	-1.2959330
H	2.3271480	-2.2818770	-0.1040600
H	2.5618630	1.9905720	0.1889230
H	4.1213180	-2.0657360	-1.0774030
H	6.5683220	-2.2163720	-1.0041610
H	7.9047090	-0.4773790	0.1808080
H	6.7335390	1.4133900	1.3084030
H	4.2844240	1.5579560	1.2679690
H	1.3356550	-4.0987610	-0.0507330
H	-0.0760850	-6.1206610	-0.1431460
H	-2.5915280	-5.8706010	-0.2148930
H	-3.5448900	-3.5519160	-0.1772480
H	-1.9054410	2.4347560	0.9134360
H	-2.6972340	4.7277060	0.6702550
H	-1.2522010	6.4694250	-0.3856570
H	1.0047600	5.8259060	-1.2371580
H	1.7869190	3.5104290	-1.0640350
H	-4.0794910	-0.1136420	2.6229660
H	-6.2662050	0.0171020	1.3971930
H	-6.3107050	0.2354030	-1.0696880
H	-4.1702250	0.3332010	-2.3768350

8_b (B3LYP/6-31+G*)

C	-1.8183260	-1.2809630	-0.0032990
C	-0.4219410	-1.4429640	-0.0250530
P	0.5779840	-0.0272120	0.0308830
C	-0.4691520	1.3522920	0.0707890
C	-1.8495520	1.1525070	0.0641230
C	-2.5321900	-0.0810490	0.0418960
C	-4.0201510	-0.1025620	0.0715490
O	1.7240580	0.1222200	-1.1820480

C	3.0047860	0.0879930	-0.6659830
C	3.0098450	0.0195990	0.7272650
O	1.7312380	0.0059170	1.2531890
C	-4.7178090	-1.0225520	0.8760100
C	-6.1137650	-1.0502420	0.8974060
C	-6.8492900	-0.1502630	0.1212240
C	-6.1719220	0.7746980	-0.6785040
C	-4.7761910	0.7948770	-0.7052580
C	0.0987380	2.7313320	0.0509000
C	0.2415420	-2.7568020	-0.1116970
C	-0.4463850	-3.9827560	-0.2124270
C	0.2839860	-5.1648310	-0.2855350
C	1.6801310	-5.1143740	-0.2608090
C	2.2796940	-3.8578580	-0.1643950
N	1.5909860	-2.7163670	-0.0917080
C	-0.3460910	3.6525260	-0.9155200
C	0.1415520	4.9600540	-0.9397760
C	1.0959410	5.3720140	-0.0051900
C	1.5526730	4.4653680	0.9547650
C	1.0580770	3.1594120	0.9862460
C	4.1793210	0.1194980	-1.3958080
C	5.3859290	0.0846540	-0.6757780
C	5.3911610	0.0156750	0.7203250
C	4.1894070	-0.0199120	1.4487670
H	-2.4084920	-2.1925590	-0.0473000
H	-2.4543170	2.0562380	0.1172870
H	-4.1600340	-1.7048240	1.5129760
H	-6.6268890	-1.7672170	1.5339930
H	-7.9359120	-0.1680690	0.1406810
H	-6.7308820	1.4757290	-1.2938420
H	-4.2653210	1.5001920	-1.3562480

H	-1.5300030	-4.0185000	-0.2380650
H	-0.2338920	-6.1176240	-0.3638660
H	2.2843960	-6.0147890	-0.3167030
H	3.3636590	-3.7576890	-0.1443120
H	-1.0673130	3.3311490	-1.6626620
H	-0.2160190	5.6533950	-1.6971870
H	1.4806110	6.3884450	-0.0262980
H	2.2908300	4.7757770	1.6901560
H	1.4022840	2.4723970	1.7528710
H	4.1582440	0.1702920	-2.4796870
H	6.3271350	0.1123590	-1.2176890
H	6.3362280	-0.0107080	1.2555540
H	4.1765670	-0.0785680	2.5325360

8_c (B3LYP/6-31+G*)

C	1.8929340	1.0683340	-0.0244080
C	0.5239820	1.3992870	-0.0075070
P	-0.6039170	0.0956890	-0.0284830
C	0.2611540	-1.3965550	-0.0546160
C	1.6573640	-1.3550450	-0.0505790
C	2.4684820	-0.2052560	-0.0442480
C	3.9505770	-0.3489950	-0.0595540
O	-1.7826790	0.2423330	-1.2345430
C	-3.0593680	0.2457750	-0.7055960
C	-3.0497740	0.1686230	0.6878930
O	-1.7671720	0.1117460	1.1957570
C	4.7685990	0.4882950	0.7214570
C	6.1582200	0.3557800	0.7071890
C	6.7662500	-0.6248060	-0.0819980
C	5.9679250	-1.4666770	-0.8611940
C	4.5785650	-1.3268520	-0.8529030

C	-0.4684670	-2.6949390	0.0157380
C	0.1174490	2.8306660	0.0093550
C	-1.2237260	3.2527890	0.1006530
C	-1.5160870	4.6138110	0.1085570
C	-0.4735640	5.5375800	0.0290510
C	0.8259260	5.0333850	-0.0529730
N	1.1189120	3.7313630	-0.0635440
C	-1.4944560	-3.0244170	-0.8881840
C	-2.1446510	-4.2585560	-0.8118940
C	-1.7779760	-5.1913600	0.1613640
C	-0.7570770	-4.8779770	1.0635980
C	-0.1147250	-3.6407810	0.9960250
C	-4.2423190	0.3217390	-1.4216400
C	-5.4398180	0.3159090	-0.6870380
C	-5.4300340	0.2381200	0.7092830
C	-4.2228580	0.1645360	1.4237040
H	2.5514660	1.9316990	-0.0451760
H	2.1544340	-2.3236450	-0.0416660
H	4.3095260	1.2372290	1.3618000
H	6.7660920	1.0132820	1.3242150
H	7.8480740	-0.7305140	-0.0906090
H	6.4271750	-2.2266110	-1.4890160
H	3.9749430	-1.9677400	-1.4909290
H	-2.0377160	2.5415070	0.1699030
H	-2.5493030	4.9446180	0.1781460
H	-0.6554310	6.6081570	0.0318330
H	1.6780380	5.7089770	-0.1146350
H	-1.7677970	-2.3218240	-1.6697450
H	-2.9323230	-4.4937660	-1.5234040
H	-2.2826520	-6.1524310	0.2172710
H	-0.4670570	-5.5928160	1.8297610

H	0.6596330	-3.3932830	1.7175000
H	-4.2330960	0.3844870	-2.5051730
H	-6.3863800	0.3730720	-1.2168500
H	-6.3692460	0.2340020	1.2549760
H	-4.1983400	0.1035950	2.5069420

8_d (B3LYP/6-31+G*)

C	1.8903090	1.0808730	-0.0522410
C	0.5189050	1.3971820	-0.0717250
P	-0.6012840	0.0896310	-0.0066440
C	0.2753730	-1.3926650	0.0805490
C	1.6718460	-1.3417710	0.0626250
C	2.4753450	-0.1878460	0.0032950
C	3.9587630	-0.3201050	0.0130560
O	-1.7701080	0.0339440	-1.2281060
C	-3.0514700	0.1158100	-0.7185630
C	-3.0551010	0.2695910	0.6682670
O	-1.7763650	0.2985280	1.1909170
C	4.7606850	0.5595830	0.7634010
C	6.1514570	0.4378440	0.7711890
C	6.7764950	-0.5730630	0.0353780
C	5.9940900	-1.4571450	-0.7125950
C	4.6038110	-1.3285500	-0.7262330
C	-0.4472730	-2.6964760	0.1149410
C	0.0955870	2.8221020	-0.1072190
C	-1.1973880	3.2297620	-0.4892620
C	-1.5166330	4.5849260	-0.4868910
C	-0.5434120	5.5142420	-0.1180420
C	0.7173980	5.0238350	0.2286700
N	1.0334190	3.7266200	0.2421300
C	-0.1364360	-3.6915770	-0.8299300

C	-0.7738420	-4.9329560	-0.8001670
C	-1.7465320	-5.2012870	0.1675160
C	-2.0701720	-4.2195170	1.1073060
C	-1.4251600	-2.9805160	1.0851110
C	-4.2276260	0.0672390	-1.4477530
C	-5.4321120	0.1765690	-0.7329740
C	-5.4355610	0.3310420	0.6568690
C	-4.2347690	0.3805880	1.3845160
H	2.5438780	1.9474000	-0.0954340
H	2.1756330	-2.3041510	0.1362750
H	4.2882380	1.3332870	1.3632600
H	6.7468300	1.1280230	1.3642330
H	7.8591100	-0.6702540	0.0443940
H	6.4666520	-2.2421510	-1.2983140
H	4.0124290	-2.0048230	-1.3387690
H	-1.9404040	2.5084100	-0.8083910
H	-2.5125640	4.9078240	-0.7796130
H	-0.7475080	6.5807530	-0.1031400
H	1.5157590	5.7066770	0.5160590
H	0.6003040	-3.4794280	-1.6004630
H	-0.5178310	-5.6867640	-1.5406890
H	-2.2469300	-6.1659920	0.1882800
H	-2.8195630	-4.4198880	1.8690490
H	-1.6637990	-2.2370980	1.8399690
H	-4.2079460	-0.0549530	-2.5259800
H	-6.3737450	0.1390610	-1.2731900
H	-6.3798480	0.4144520	1.1872640
H	-4.2210420	0.5027510	2.4628850

8_ω_TS_a (B3LYP/6-31+G*)

C	-1.7556170	-1.2814570	-0.0120760
C	-0.3631720	-1.3920160	-0.0085170
P	0.5880830	0.0375710	-0.0140620
C	-0.4707030	1.3958990	-0.0544760
C	-1.8521530	1.1520970	-0.0518730
C	-2.5034080	-0.0911790	-0.0339960
C	-3.9901710	-0.1507100	-0.0471800
O	1.7777970	-0.0192800	-1.2177050
C	3.0510810	0.0149340	-0.6782370
C	3.0262020	0.1142470	0.7130110
O	1.7383050	0.1603660	1.2073380
C	-4.6868190	-1.0705810	0.7586830
C	-6.0816360	-1.1315050	0.7437190
C	-6.8176860	-0.2664530	-0.0710660
C	-6.1414170	0.6567050	-0.8735850
C	-4.7461640	0.7099360	-0.8648390
C	0.0649500	2.7844640	0.0005350
C	0.3005830	-2.7362940	-0.0043300
C	0.5824790	-3.3923790	-1.2129330
C	1.1746850	-4.6548810	-1.1775410
C	1.4725490	-5.2208330	0.0621370
C	1.1620720	-4.4900310	1.2115770
N	0.5917150	-3.2787120	1.1920310
C	1.0889890	3.2195180	-0.8605840
C	1.5651480	4.5312050	-0.8008650
C	1.0236670	5.4387290	0.1129350
C	0.0037450	5.0204650	0.9728260
C	-0.4646830	3.7067840	0.9224110
C	4.2413430	-0.0392070	-1.3828910
C	5.4319860	0.0082560	-0.6381470
C	5.4069290	0.1058730	0.7564490

C	4.1914150	0.1601650	1.4587690
H	-2.3035670	-2.2223420	-0.0239550
H	-2.4788990	2.0424150	-0.0543960
H	-4.1307040	-1.7274290	1.4230720
H	-6.5940970	-1.8480050	1.3813710
H	-7.9037180	-0.3109800	-0.0801900
H	-6.7000350	1.3298420	-1.5196940
H	-4.2348640	1.4114600	-1.5195940
H	0.3386470	-2.9122430	-2.1557790
H	1.3998770	-5.1836900	-2.1002960
H	1.9348770	-6.2006980	0.1419050
H	1.3807840	-4.8935970	2.1988720
H	1.4985570	2.5342230	-1.5969400
H	2.3549770	4.8453090	-1.4787870
H	1.3930820	6.4600580	0.1563450
H	-0.4202760	5.7140390	1.6948930
H	-1.2358970	3.3825650	1.6162910
H	4.2442020	-0.1166090	-2.4655730
H	6.3845340	-0.0323550	-1.1588080
H	6.3405370	0.1408230	1.3107100
H	4.1547520	0.2354320	2.5406900

8_ω_TS_b (B3LYP/6-31+G*)

C	-1.7516970	-1.2865750	-0.0257310
C	-0.3587290	-1.3935600	-0.0135930
P	0.5891170	0.0377250	-0.0424660
C	-0.4727350	1.3943450	-0.0417880
C	-1.8535160	1.1470290	-0.0417430
C	-2.5020220	-0.0979930	-0.0436950
C	-3.9886360	-0.1589930	-0.0714520
O	1.7686150	0.0563220	-1.2485130

C	3.0450480	0.0569890	-0.7190640
C	3.0337500	0.0831760	0.6755730
O	1.7485490	0.0913810	1.1831310
C	-4.6940460	-1.0854530	0.7189780
C	-6.0885720	-1.1461940	0.6895770
C	-6.8161150	-0.2739150	-0.1251020
C	-6.1313810	0.6554830	-0.9130120
C	-4.7362900	0.7082980	-0.8896580
C	0.0580690	2.7838750	0.0388830
C	0.3085740	-2.7359550	0.0092730
C	0.5697350	-3.3763170	1.2310440
C	1.1660710	-4.6375510	1.2225120
C	1.4870280	-5.2184460	-0.0042330
C	1.1955250	-4.5028540	-1.1683070
N	0.6232110	-3.2925780	-1.1744970
C	1.0719190	3.2441750	-0.8212370
C	1.5405470	4.5571760	-0.7359330
C	1.0019520	5.4407720	0.2027150
C	-0.0078590	4.9972620	1.0618640
C	-0.4689200	3.6821790	0.9857820
C	4.2287670	0.0403960	-1.4364670
C	5.4261680	0.0525610	-0.7014020
C	5.4148470	0.0803440	0.6964610
C	4.2057150	0.0952380	1.4119590
H	-2.2969030	-2.2288270	-0.0480960
H	-2.4823980	2.0357910	-0.0311670
H	-4.1452220	-1.7499250	1.3819820
H	-6.6076680	-1.8687040	1.3150070
H	-7.9019660	-0.3185250	-0.1461870
H	-6.6830840	1.3334970	-1.5599160
H	-4.2180670	1.4138390	-1.5344200

H	0.3079260	-2.8851080	2.1634520
H	1.3764770	-5.1537510	2.1558430
H	1.9527320	-6.1981760	-0.0633530
H	1.4322640	-4.9187500	-2.1462870
H	1.4783440	2.5775700	-1.5758200
H	2.3220240	4.8913890	-1.4139070
H	1.3654410	6.4632300	0.2654890
H	-0.4301340	5.6721780	1.8024310
H	-1.2333660	3.3380490	1.6776500
H	4.2202350	0.0154650	-2.5214460
H	6.3737010	0.0398520	-1.2325200
H	6.3536750	0.0906260	1.2428700
H	4.1802660	0.1178800	2.4967230

8_θ_TS_a (B3LYP/6-31+G*)

C	1.8156200	-1.2875240	0.0079360
C	0.4164720	-1.4450000	-0.0061300
P	-0.5764710	-0.0240440	0.0288510
C	0.4730750	1.3535860	0.0667810
C	1.8547630	1.1476850	0.0622770
C	2.5259310	-0.0887090	0.0418380
C	4.0249700	-0.1137240	0.0613690
O	-1.7386860	0.0087200	1.2440980
C	-3.0138130	0.0247500	0.7105100
C	-3.0002680	0.0959930	-0.6826830
O	-1.7170900	0.1309660	-1.1906730
C	4.7603340	-0.0833500	-1.1345850
C	6.1580090	-0.1060330	-1.1180920
C	6.8444890	-0.1589520	0.0982240
C	6.1249730	-0.1891160	1.2960980
C	4.7273830	-0.1662200	1.2762010

C	-0.0887780	2.7339650	0.0257560
C	-0.2512060	-2.7581990	-0.0565770
C	0.4331550	-3.9904300	-0.0674920
C	-0.3001100	-5.1718380	-0.1174130
C	-1.6957770	-5.1152100	-0.1549470
C	-2.2916800	-3.8533570	-0.1396230
N	-1.6002410	-2.7121660	-0.0926890
C	-1.0792930	3.1658580	0.9265330
C	-1.5695820	4.4728160	0.8768150
C	-1.0773570	5.3779140	-0.0668220
C	-0.0913970	4.9626750	-0.9666150
C	0.3920010	3.6540980	-0.9246870
C	-4.1977250	-0.0150260	1.4249050
C	-5.3952420	0.0224910	0.6895420
C	-5.3816690	0.0937340	-0.7063800
C	-4.1707290	0.1291670	-1.4191020
H	2.4083350	-2.1989390	-0.0101090
H	2.4693470	2.0465540	0.0902850
H	4.2293990	-0.0429420	-2.0828880
H	6.7094060	-0.0821950	-2.0550360
H	7.9314450	-0.1760670	0.1125250
H	6.6506190	-0.2298830	2.2471430
H	4.1707390	-0.1882420	2.2101940
H	1.5164240	-4.0315590	-0.0370590
H	0.2152400	-6.1291870	-0.1265730
H	-2.3022980	-6.0150010	-0.1943110
H	-3.3750890	-3.7484340	-0.1661440
H	-1.4518950	2.4811260	1.6818170
H	-2.3326310	4.7850670	1.5855700
H	-1.4587340	6.3951900	-0.1022880
H	0.2947090	5.6544760	-1.7113460

H	1.1383350	3.3315250	-1.6461340
H	-4.1913280	-0.0757580	2.5086380
H	-6.3434700	-0.0042950	1.2191630
H	-6.3196270	0.1228560	-1.2538580
H	-4.1432580	0.1817840	-2.5027670

8_θ_TS_b (B3LYP/6-31+G*)

C	1.8038380	-1.2837620	0.0072120
C	0.4080300	-1.4412120	-0.0062250
P	-0.5931060	-0.0275310	0.0298870
C	0.4658120	1.3404170	0.0615700
C	1.8437560	1.1342630	0.0584590
C	2.5413890	-0.0942920	0.0422310
C	4.0384060	-0.1171950	0.0638460
O	-1.7478620	0.0137330	1.2483860
C	-3.0250830	0.0332570	0.7179330
C	-3.0150860	0.0997050	-0.6753200
O	-1.7324010	0.1280520	-1.1873280
C	4.7678790	-1.3252280	0.0601310
C	6.1625170	-1.3480260	0.0776280
C	6.8940550	-0.1588880	0.1007580
C	6.1973140	1.0505390	0.1069160
C	4.8020280	1.0691150	0.0893110
C	-0.0904760	2.7239170	0.0152920
C	-0.2595130	-2.7558300	-0.0562270
C	0.4196710	-3.9903670	-0.0749160
C	-0.3182730	-5.1690720	-0.1228880
C	-1.7137940	-5.1072840	-0.1510730
C	-2.3049550	-3.8433250	-0.1289650
N	-1.6084720	-2.7051850	-0.0835600
C	-1.0512340	3.1731960	0.9390480

C	-1.5362440	4.4820140	0.8848810
C	-1.0686930	5.3699790	-0.0872850
C	-0.1135090	4.9363810	-1.0112200
C	0.3648510	3.6259290	-0.9642600
C	-4.2069570	0.0004080	1.4358990
C	-5.4061130	0.0403420	0.7034740
C	-5.3960690	0.1069800	-0.6927450
C	-4.1871000	0.1353560	-1.4090030
H	2.3603440	-2.2130860	-0.0137280
H	2.4226260	2.0519460	0.0818520
H	4.2545860	-2.2804340	0.0446310
H	6.6770550	-2.3060850	0.0739610
H	7.9805190	-0.1749100	0.1148790
H	6.7389010	1.9933110	0.1266010
H	4.3171950	2.0388050	0.0975690
H	1.5029270	-4.0372000	-0.0521490
H	0.1934300	-6.1282550	-0.1379180
H	-2.3237780	-6.0047670	-0.1886080
H	-3.3880140	-3.7340580	-0.1484210
H	-1.4039170	2.5008140	1.7149140
H	-2.2754980	4.8092580	1.6118090
H	-1.4460230	6.3886200	-0.1262970
H	0.2516560	5.6147450	-1.7784900
H	1.0857050	3.2871680	-1.7041280
H	-4.1977360	-0.0565450	2.5197810
H	-6.3530090	0.0191200	1.2356600
H	-6.3354050	0.1381250	-1.2376800
H	-4.1622450	0.1845190	-2.4928640

8_ϕ_TS_a (B3LYP/6-31+G*)

C	1.8737550	1.2207960	-0.0136920

C	0.4830890	1.4353210	-0.0213160
P	-0.5639570	0.0537240	0.0018490
C	0.4368860	-1.3551630	0.0076290
C	1.8208570	-1.2142850	0.0060260
C	2.5468030	-0.0029200	0.0006020
C	4.0345170	-0.0342490	0.0113250
O	-1.7203440	-0.0136540	-1.2106240
C	-2.9969590	0.0156840	-0.6821900
C	-2.9895620	0.0178060	0.7128730
O	-1.7076870	-0.0111040	1.2276150
C	4.7751390	0.8494350	0.8183880
C	6.1712790	0.8260920	0.8227420
C	6.8642000	-0.0903280	0.0267750
C	6.1438420	-0.9798780	-0.7755440
C	4.7480220	-0.9490850	-0.7853680
C	-0.2068650	-2.7079530	0.0308970
C	-0.1345560	2.7730520	-0.0604080
C	0.5932290	3.9768880	-0.1449270
C	-0.0972100	5.1847040	-0.1724030
C	-1.4934850	5.1810350	-0.1188010
C	-2.1341690	3.9435260	-0.0413990
N	-1.4841470	2.7777820	-0.0121120
C	-0.5182050	-3.3332120	1.2492460
C	-1.0954550	-4.6054410	1.2698020
C	-1.3749930	-5.2689170	0.0718320
C	-1.0761530	-4.6530450	-1.1466630
C	-0.4989450	-3.3807220	-1.1667390
C	-4.1776160	0.0365610	-1.4025520
C	-5.3778650	0.0565050	-0.6713840
C	-5.3705940	0.0590560	0.7264400
C	-4.1628440	0.0415180	1.4451890

H	2.4953820	2.1120560	-0.0423440
H	2.3902330	-2.1420740	0.0357620
H	4.2506560	1.5435600	1.4707430
H	6.7177670	1.5159620	1.4615500
H	7.9509090	-0.1123070	0.0329420
H	6.6692760	-1.6931540	-1.4061690
H	4.2040450	-1.6272270	-1.4383770
H	1.6767220	3.9757320	-0.1936260
H	0.4514510	6.1211110	-0.2378500
H	-2.0673200	6.1026320	-0.1388860
H	-3.2203130	3.8796800	-0.0009540
H	-0.3047140	-2.8157390	2.1807790
H	-1.3274160	-5.0767110	2.2217030
H	-1.8247180	-6.2585280	0.0877400
H	-1.2936200	-5.1612050	-2.0828970
H	-0.2719310	-2.8995620	-2.1145370
H	-4.1660350	0.0372250	-2.4878170
H	-6.3236970	0.0696470	-1.2057650
H	-6.3108290	0.0741440	1.2705650
H	-4.1400830	0.0458270	2.5302710

8_ϕ_TS_b (B3LYP/6-31+G*)

C	-1.7399420	-1.3660740	-0.0019630
C	-0.3407700	-1.4379310	-0.0211330
P	0.5759550	0.0376840	0.0116640
C	-0.5376610	1.3791490	0.0068180
C	-1.9025590	1.0621890	0.0158560
C	-2.5130810	-0.2033830	0.0170540
C	-3.9987450	-0.3014890	0.0337930
O	1.7401660	0.1361200	-1.1922780
C	3.0130860	0.1254170	-0.6547140

C	2.9952490	0.1335240	0.7410740
O	1.7099760	0.1548060	1.2453420
C	-4.6562210	-1.2307000	0.8608200
C	-6.0491160	-1.3297830	0.8699030
C	-6.8211720	-0.4942310	0.0578110
C	-6.1838430	0.4377360	-0.7661490
C	-4.7906470	0.5291300	-0.7806680
C	-0.0711590	2.7992660	0.0091920
C	0.3949030	-2.7162360	-0.0798800
C	-0.2286500	-3.9691070	-0.2513730
C	0.5558830	-5.1175320	-0.2852860
C	1.9429610	-5.0078130	-0.1570620
C	2.4777550	-3.7282660	-0.0042660
N	1.7356130	-2.6187170	0.0353490
C	1.2954350	3.1460660	0.0219720
C	1.7186790	4.4759360	0.0209310
C	0.7916780	5.5180070	0.0067680
C	-0.5687420	5.2009870	-0.0071540
C	-0.9897760	3.8722660	-0.0065980
C	4.1990750	0.1218480	-1.3670030
C	5.3938390	0.1264190	-0.6267730
C	5.3764600	0.1339010	0.7712780
C	4.1638430	0.1373490	1.4818730
H	-2.2780400	-2.3097410	-0.0222020
H	-2.5884040	1.9013950	0.0497460
H	-4.0706760	-1.8630700	1.5239030
H	-6.5319140	-2.0517680	1.5242490
H	-7.9055660	-0.5679700	0.0674490
H	-6.7713410	1.0872070	-1.4107700
H	-4.3085980	1.2350610	-1.4528300
H	-1.3037760	-4.0512590	-0.3665040

H	0.0873090	-6.0897970	-0.4166070
H	2.5887520	-5.8804100	-0.1802830
H	3.5523710	-3.5812970	0.0911000
H	2.0626290	2.3843250	0.0336890
H	2.7845150	4.6898910	0.0314970
H	1.1199840	6.5539240	0.0061600
H	-1.3146040	5.9920850	-0.0190020
H	-2.0577510	3.6882500	-0.0194470
H	4.1950000	0.1147950	-2.4522570
H	6.3436960	0.1255160	-1.1540700
H	6.3130150	0.1389450	1.3218590
H	4.1330900	0.1424110	2.5667050

9_a (B3LYP/6-31+G*)

N	2.2946360	-0.1187340	-1.2139940
P	1.1013090	0.0000000	0.0000000
C	0.0392090	1.3896750	-0.1116750
C	-1.3482490	1.2122680	-0.0938370
C	-2.0526220	0.0000000	0.0000000
C	-1.3481620	-1.2123430	0.0938400
C	0.0393100	-1.3896690	0.1116490
C	0.6103060	-2.7659760	0.1024330
C	1.6924920	-3.1454150	0.9217370
C	2.1936190	-4.4494620	0.8989260
C	1.6249230	-5.4145070	0.0637200
C	0.5493200	-5.0570480	-0.7548700
C	0.0559680	-3.7516430	-0.7408540
N	2.2945770	0.1190020	1.2140250
C	3.6452270	0.3216990	0.6969270
C	3.6452430	-0.3215420	-0.6968030
C	0.6101450	2.7660080	-0.1024710

C	0.0558180	3.7516520	0.7408480
C	0.5491230	5.0570750	0.7548330
C	1.6246720	5.4145680	-0.0638150
C	2.1933610	4.4495420	-0.8990460
C	1.6922770	3.1454790	-0.9218280
H	2.1288330	2.4144880	-1.5946010
C	-3.5384910	-0.0001170	0.0000000
C	-4.2700670	0.8782960	-0.8235160
C	-5.6661650	0.8832140	-0.8196890
C	-6.3747480	-0.0001330	0.0000000
C	-5.6661950	-0.8834480	0.8196780
C	-4.2700420	-0.8785080	0.8235230
H	-0.7621910	-3.4828040	-1.4040340
C	1.9898080	-0.6513090	-2.5335530
C	1.9897050	0.6513340	2.5336610
H	-6.2013830	1.5704100	-1.4711330
H	-3.7380370	1.5489680	-1.4938280
H	-3.7380870	-1.5492080	1.4938690
H	-6.2013190	-1.5706880	1.4711450
H	-7.4618190	-0.0001170	0.0000000
H	-1.9394980	-2.1254280	0.1458210
H	-1.9396390	2.1253180	-0.1458150
H	3.8875280	-1.3950800	-0.6356190
H	4.3815770	0.1569750	-1.3557730
H	3.8876030	1.3952180	0.6357660
H	4.3814860	-0.1568800	1.3559390
H	-0.7623010	3.4827880	1.4040680
H	0.0983350	5.7933000	1.4163320
H	2.0123810	6.4300280	-0.0513450
H	3.0241550	4.7143980	-1.5493620
H	0.0985220	-5.7932860	-1.4163470

H	2.0126670	-6.4299520	0.0512270
H	3.0244530	-4.7142930	1.5492010
H	2.1290500	-2.4144240	1.5945060
H	2.6217380	0.1620380	3.2861970
H	2.1529080	1.7384740	2.5919600
H	0.9445880	0.4457300	2.7834070
H	0.9447790	-0.4454910	-2.7834930
H	2.6220700	-0.1623210	-3.2860930
H	2.1527610	-1.7384990	-2.5916020

9_a (B3LYP/6-31+G*, excited state minima)

N	-2.2506270	-0.1188610	1.2224650
P	-1.0807440	0.0011810	0.0001100
C	-0.0298870	1.4330600	0.1335740
C	1.3630350	1.2374130	0.1244380
C	2.0510780	-0.0023290	-0.0000150
C	1.3602500	-1.2405210	-0.1244750
C	-0.0331100	-1.4330070	-0.1335210
C	-0.6197490	-2.7750450	-0.1112970
C	-1.8806500	-3.0874380	-0.6918360
C	-2.3944870	-4.3801000	-0.6623150
C	-1.6810860	-5.4267040	-0.0570500
C	-0.4309620	-5.1448090	0.5127960
C	0.0928620	-3.8568170	0.4855090
N	-2.2505040	0.1236880	-1.2221120
C	-3.6061490	0.3197190	-0.6998260
C	-3.6067130	-0.3121770	0.7002930
C	-0.6133450	2.7764890	0.1111900
C	0.1016480	3.8563020	-0.4863210
C	-0.4190060	5.1455690	-0.5138470
C	-1.6681450	5.4307670	0.0565040

C	-2.3838140	4.3861540	0.6625310
C	-1.8731680	3.0922320	0.6922660
H	-2.4258170	2.3175700	1.2124360
C	3.5180380	-0.0039550	0.0000140
C	4.2611410	1.0334210	0.6198010
C	5.6529940	1.0274900	0.6239660
C	6.3600590	-0.0070740	0.0001190
C	5.6507690	-1.0400760	-0.6237920
C	4.2589050	-1.0429500	-0.6197350
H	1.0458490	-3.6658530	0.9705150
C	-1.9384990	-0.6845740	2.5294250
C	-1.9373500	0.6886870	-2.5291340
H	6.1900350	1.8310970	1.1215690
H	3.7399430	1.8311680	1.1385510
H	3.7360000	-1.8395400	-1.1385400
H	6.1860820	-1.8448560	-1.1213620
H	7.4468520	-0.0082660	0.0001590
H	1.9609260	-2.1392510	-0.1993610
H	1.9657500	2.1347830	0.1992540
H	-3.8622350	-1.3813630	0.6550620
H	-4.3302440	0.1859110	1.3586400
H	-3.8595280	1.3894110	-0.6545710
H	-4.3307370	-0.1769240	-1.3581020
H	1.0539230	3.6628140	-0.9717240
H	0.1509400	5.9374820	-0.9954600
H	-2.0694860	6.4402940	0.0371710
H	-3.3440320	4.5886070	1.1328560
H	0.1372590	-5.9383090	0.9938350
H	-2.0849120	-6.4352430	-0.0378930
H	-3.3554530	-4.5799960	-1.1322030
H	-2.4316920	-2.3111750	-1.2113480

H	-2.6064900	0.2526520	-3.2808710
H	-2.0458610	1.7829300	-2.5448680
H	-0.9097200	0.4354340	-2.8056930
H	-0.9103340	-0.4334200	2.8059110
H	-2.6066900	-0.2472290	3.2812450
H	-2.0492250	-1.7785960	2.5451000

9_b (B3LYP/6-31+G*)

N	2.2967220	-0.1389590	-1.2112520
P	1.1027720	0.0012670	0.0000000
C	0.0391760	1.3864620	-0.1420590
C	-1.3486220	1.2085760	-0.1166430
C	-2.0519130	-0.0026200	-0.0001560
C	-1.3456460	-1.2120860	0.1162740
C	0.0425940	-1.3865740	0.1416930
C	0.6150630	-2.7614550	0.1936540
C	1.6830660	-3.1119070	1.0438150
C	2.1829940	-4.4162050	1.0759140
C	1.6275810	-5.4102180	0.2661070
C	0.5665380	-5.0818040	-0.5830080
C	0.0747230	-3.7764460	-0.6234570
N	2.2957580	0.1443890	1.2120860
C	3.6462900	0.3389360	0.6915720
C	3.6475660	-0.3295880	-0.6901240
C	0.6081450	2.7628110	-0.1940520
C	0.0652220	3.7763360	0.6231570
C	0.5535440	5.0830050	0.5826720
C	1.6135440	5.4142770	-0.2666310
C	2.1714110	4.4217980	-1.0766360
C	1.6749720	3.1161660	-1.0444940
H	2.1030510	2.3628910	-1.6975920

C	-3.5385620	-0.0043930	0.0000000
C	-4.2718360	1.0022230	0.6583630
C	-5.6678440	1.0054800	0.6537800
C	-6.3752550	-0.0075770	0.0001230
C	-5.6656230	-1.0190690	-0.6536070
C	-4.2696370	-1.0126810	-0.6583340
H	-0.7316740	-3.5304280	-1.3095450
C	1.9925520	-0.7007100	-2.5187250
C	1.9893610	0.7054990	2.5193270
H	-6.2040330	1.7949580	1.1755860
H	-3.7408780	1.7799460	1.2016290
H	-3.7369980	-1.7892100	-1.2016560
H	-6.2000860	-1.8097830	-1.1753020
H	-7.4623390	-0.0088160	0.0001010
H	-1.9349180	-2.1200080	0.2362350
H	-1.9401240	2.1150630	-0.2365350
H	3.8917220	-1.4014480	-0.6091650
H	4.3834560	0.1379170	-1.3574600
H	3.8873940	1.4114940	0.6107260
H	4.3832280	-0.1264440	1.3592370
H	-0.7404550	3.5281320	1.3093070
H	0.1114640	5.8410190	1.2251890
H	1.9975230	6.4307660	-0.2972200
H	2.9899420	4.6659840	-1.7501480
H	0.1263830	-5.8410100	-1.2254390
H	2.0142940	-6.4256710	0.2966450
H	3.0023490	-4.6581560	1.7492310
H	2.1093030	-2.3574320	1.6967460
H	2.6209190	0.2334620	3.2832140
H	2.1523490	1.7937510	2.5536980
H	0.9441280	0.5048910	2.7726250

H	0.9468430	-0.5031250	-2.7724380
H	2.6230640	-0.2267000	-3.2822610
H	2.1587300	-1.7884730	-2.5532050

9_c (B3LYP/6-31+G*)

N	2.2344390	0.2044880	1.4029620
P	1.1012750	0.1358820	0.1285750
C	0.1988730	-1.3678460	0.0915760
C	-1.1996780	-1.3500710	0.0892920
C	-2.0353860	-0.2197410	0.0952380
C	-1.4697300	1.0642580	0.0410980
C	-0.1097230	1.3977090	0.0204510
C	0.2931390	2.8177540	-0.1764440
C	-0.3661170	3.6071540	-1.1405130
C	-0.0362070	4.9502840	-1.3287530
C	0.9763490	5.5423230	-0.5685640
C	1.6484740	4.7732220	0.3852210
C	1.3090950	3.4327330	0.5793160
N	2.3382030	0.3191070	-1.0224700
C	3.6843750	0.1415050	-0.4861260
C	3.5940190	0.5384330	0.9960540
C	0.9182110	-2.6681640	-0.0287390
C	0.4951790	-3.6102050	-0.9894630
C	1.1216640	-4.8507040	-1.1172650
C	2.2038850	-5.1821350	-0.2966410
C	2.6432240	-4.2590040	0.6557910
C	2.0083170	-3.0217500	0.7910180
H	2.3446460	-2.3271280	1.5534320
C	-3.5119250	-0.3795370	0.1519580
C	-4.1665240	-1.4052410	-0.5581330
C	-5.5530400	-1.5562960	-0.5025870

C	-6.3307180	-0.6766040	0.2563800
C	-5.7000560	0.3514040	0.9629090
C	-4.3117710	0.4930930	0.9157020
H	1.8238880	2.8637900	1.3465550
C	1.8557930	0.3804210	2.7927460
C	2.1267800	-0.0011330	-2.4255910
H	-6.0283550	-2.3557390	-1.0665980
H	-3.5838150	-2.0789910	-1.1814410
H	-3.8369570	1.2775270	1.4998660
H	-6.2888160	1.0386970	1.5663180
H	-7.4109690	-0.7908300	0.2966840
H	-2.1593000	1.9058290	-0.0063960
H	-1.6821240	-2.3259190	0.1234730
H	3.8004350	1.6132590	1.1292050
H	4.3205990	-0.0195830	1.6016670
H	4.0180360	-0.9038150	-0.5932270
H	4.3926340	0.7831340	-1.0257560
H	-0.3271330	-3.3547800	-1.6529290
H	0.7702090	-5.5547000	-1.8680550
H	2.6961230	-6.1460830	-0.3972980
H	3.4767590	-4.5068130	1.3092440
H	2.4328470	5.2207940	0.9914580
H	1.2377720	6.5868980	-0.7170310
H	-0.5647500	5.5317330	-2.0806310
H	-1.1359980	3.1533780	-1.7591340
H	2.7485760	0.6518260	-3.0507750
H	2.3745930	-1.0494620	-2.6547740
H	1.0801860	0.1731820	-2.6916150
H	0.8035070	0.1123850	2.9270510
H	2.4596830	-0.2722670	3.4382270
H	1.9910870	1.4191100	3.1348340

9_d (B3LYP/6-31+G*)

N	-2.3001950	0.2411690	1.1862930
P	-1.1057900	0.0000000	0.0001030
C	-0.0363290	1.3893230	-0.0694400
C	1.3511440	1.2132920	-0.0613480
C	2.0568870	0.0000000	0.0000000
C	1.3511330	-1.2132880	0.0613260
C	-0.0363480	-1.3893170	0.0693880
C	-0.6032110	-2.7660960	0.0508370
C	-0.0764950	-3.7379140	-0.8233040
C	-0.5717170	-5.0431250	-0.8407440
C	-1.6201230	-5.4124580	0.0067470
C	-2.1597800	-4.4605750	0.8762390
C	-1.6556150	-3.1586010	0.8993420
N	-2.3004960	-0.2410310	-1.1857720
C	-3.6630130	0.0597160	-0.7670390
C	-3.6628340	-0.0594800	0.7678890
C	-0.6032030	2.7661010	-0.0510510
C	-1.6555580	3.1584990	-0.8996670
C	-2.1597340	4.4604720	-0.8767440
C	-1.6201370	5.4124600	-0.0073290
C	-0.5717770	5.0432320	0.8402650
C	-0.0765440	3.7380240	0.8230050
H	0.7216740	3.4594420	1.5063330
C	3.5426820	0.0000000	0.0000000
C	4.2740290	0.8958860	-0.8045590
C	5.6701360	0.9006590	-0.8006970
C	6.3786210	0.0000000	0.0000000
C	5.6701740	-0.9006530	0.8007220
C	4.2740230	-0.8958950	0.8045900

H	-2.0667740	-2.4434010	1.6051650
C	-2.0070470	0.3191500	2.6046750
C	-2.0077360	-0.3194590	-2.6042060
H	6.2054390	1.6015230	-1.4373340
H	3.7417700	1.5806950	-1.4602180
H	3.7418320	-1.5807450	1.4602620
H	6.2054100	-1.6015440	1.4373780
H	7.4656950	0.0000000	0.0000000
H	1.9408680	-2.1285200	0.0908590
H	1.9408800	2.1285210	-0.0909390
H	-3.9671330	-1.0713720	1.0830770
H	-4.3608960	0.6568220	1.2203930
H	-3.9673080	1.0716300	-1.0821520
H	-4.3612330	-0.6565340	-1.2193800
H	-2.0666680	2.4432090	-1.6054270
H	-2.9679950	4.7347020	-1.5509690
H	-2.0094560	6.4272110	0.0079710
H	-0.1431950	5.7700880	1.5264150
H	-2.9680790	-4.7348880	1.5503820
H	-2.0094350	-6.4272100	-0.0086960
H	-0.1430890	-5.7698980	-1.5269520
H	0.7217590	-3.4592440	-1.5065530
H	-2.6078560	-1.1116030	-3.0708430
H	-2.2181380	0.6279540	-3.1274740
H	-0.9512710	-0.5632930	-2.7501000
H	-0.9505420	0.5629370	2.7503640
H	-2.6070360	1.1111540	3.0717190
H	-2.2173180	-0.6284220	3.1277100

9_e (B3LYP/6-31+G*)

N	2.3443740	0.2229240	-1.0287060

P	1.1055070	0.0733570	0.1251120
C	-0.0330820	1.4035300	0.0545200
C	-1.4102610	1.1483230	0.0645230
C	-2.0469350	-0.1033350	0.0909330
C	-1.2752010	-1.2780070	0.0592410
C	0.1195570	-1.3758270	0.0544570
C	0.7629880	-2.7085080	-0.1265730
C	1.8488280	-3.1457700	0.6580050
C	2.4140180	-4.4096590	0.4706670
C	1.9067860	-5.2771100	-0.5000730
C	0.8273420	-4.8625240	-1.2859740
C	0.2704380	-3.5953830	-1.1065160
N	2.2487980	0.0411070	1.3915570
C	3.6212070	0.3181050	0.9839190
C	3.6822240	-0.0425550	-0.5087740
C	0.4526450	2.8056130	-0.0719730
C	1.4778200	3.3325260	0.7359780
C	1.8976040	4.6577500	0.6042480
C	1.2988960	5.4991430	-0.3372320
C	0.2775250	4.9954870	-1.1477970
C	-0.1325900	3.6672320	-1.0214230
H	-0.9080790	3.2810270	-1.6778660
C	-3.5294740	-0.1902100	0.1485980
C	-4.3469330	0.7073380	-0.5663930
C	-5.7391480	0.6252700	-0.5087560
C	-6.3585310	-0.3670850	0.2570370
C	-5.5644870	-1.2706910	0.9686850
C	-4.1721400	-1.1787580	0.9196700
H	-0.5507230	-3.2764850	-1.7433410
C	2.1060130	-0.0305180	-2.4408690
C	1.8911530	0.1726540	2.7915830

H	-6.3417510	1.3308290	-1.0763630
H	-3.8858420	1.4653460	-1.1947730
H	-3.5727160	-1.8689510	1.5081080
H	-6.0295340	-2.0426410	1.5777150
H	-7.4426750	-0.4343890	0.2987260
H	-1.8136510	-2.2241250	0.0227830
H	-2.0485520	2.0302660	0.0874220
H	3.9586240	-1.1006760	-0.6473540
H	4.4206010	0.5748690	-1.0361500
H	3.8825760	1.3770630	1.1432150
H	4.3216780	-0.2921300	1.5695200
H	1.9347570	2.7064760	1.4952250
H	2.6867480	5.0370890	1.2495350
H	1.6228130	6.5318990	-0.4373270
H	-0.1948860	5.6345190	-1.8902710
H	0.4232800	-5.5225030	-2.0501200
H	2.3445010	-6.2620330	-0.6413800
H	3.2462500	-4.7218380	1.0977180
H	2.2372690	-2.4949460	1.4342020
H	2.4763570	-0.5294330	3.4014510
H	2.0691850	1.1896410	3.1765060
H	0.8308880	-0.0615390	2.9268590
H	1.0694780	0.2144920	-2.6902750
H	2.7605100	0.6092080	-3.0461050
H	2.2905210	-1.0817810	-2.7125710

9_f (B3LYP/6-31+G*)

N	2.3011390	-0.2688730	1.1794590
P	1.1060320	0.0001420	0.0000000
C	0.0369990	-1.3870450	-0.1014530
C	-1.3510200	-1.2120330	-0.0859480

C	-2.0572560	-0.0002700	0.0000000
C	-1.3513420	1.2116820	0.0859400
C	0.0366310	1.3870650	0.1014400
C	0.6038420	2.7630570	0.1463190
C	0.0857750	3.7687300	-0.6940800
C	0.5789890	5.0740950	-0.6543840
C	1.6176770	5.4102490	0.2184720
C	2.1495820	4.4247420	1.0546270
C	1.6470810	3.1223660	1.0204350
N	2.3012090	0.2694300	-1.1792080
C	3.6640010	-0.0409130	-0.7685350
C	3.6639240	0.0416620	0.7689200
C	0.6046000	-2.7628710	-0.1464250
C	1.6480060	-3.1217810	-1.0205060
C	2.1509040	-4.4240020	-1.0547860
C	1.6192450	-5.4097470	-0.2187560
C	0.5804070	-5.0739850	0.6540740
C	0.0868000	-3.7687720	0.6938650
H	-0.7034820	-3.5166070	1.3964240
C	-3.5437440	-0.0004790	0.0000000
C	-4.2757170	-0.9920130	0.6822870
C	-5.6717060	-0.9970910	0.6777350
C	-6.3803920	-0.0008710	0.0000000
C	-5.6719600	0.9955620	-0.6777280
C	-4.2759950	0.9908730	-0.6822730
H	2.0517930	2.3799310	1.7010920
C	2.0096430	-0.3985310	2.5942690
C	2.0098570	0.3991150	-2.5940440
H	-6.2069480	-1.7745600	1.2182450
H	-3.7439130	-1.7558790	1.2440320
H	-3.7443660	1.7548670	-1.2440080

H	-6.2074490	1.7728620	-1.2182340
H	-7.4674240	-0.0009920	0.0000000
H	-1.9403050	2.1223560	0.1852450
H	-1.9397420	-2.1228600	-0.1852830
H	3.9683120	1.0456130	1.1084740
H	4.3615570	-0.6856090	1.2042350
H	3.9685460	-1.0448250	-1.1080620
H	4.3615740	0.6864520	-1.2037880
H	2.0525500	-2.3791530	-1.7010560
H	2.9517480	-4.6716710	-1.7478580
H	2.0072910	-6.4246560	-0.2485090
H	0.1583610	-5.8274680	1.3151070
H	2.9503030	4.6727210	1.7477300
H	2.0054120	6.4252790	0.2481570
H	0.1567520	5.8273910	-1.3155070
H	-0.7043930	3.5162620	-1.3966580
H	2.6083530	1.2093890	-3.0308800
H	2.2232040	-0.5277830	-3.1515880
H	0.9527790	0.6449770	-2.7321250
H	0.9526420	-0.6447960	2.7322290
H	2.6083950	-1.2085320	3.0312570
H	2.2225520	0.5285000	3.1517610

9_ω_TS_a (B3LYP/6-31+G*)

N	-2.2923440	0.3675530	-1.0793170
P	-1.0790580	0.1887180	0.1003520
C	-0.2769800	-1.3629290	0.0168100
C	1.1143740	-1.4509970	0.0213150
C	2.0277640	-0.3796640	0.0735430
C	1.5498300	0.9400130	0.0868970

C	0.2151620	1.3691640	0.0884050
C	-0.0921390	2.8208730	-0.0467490
C	-1.0923970	3.4636140	0.7102560
C	-1.3429220	4.8305340	0.5659640
C	-0.5981420	5.5982560	-0.3330110
C	0.3987880	4.9786880	-1.0925950
C	0.6411090	3.6111230	-0.9570830
N	-2.2209370	0.4094120	1.3494370
C	-3.6097650	0.3580200	0.9049140
C	-3.5775260	0.8219630	-0.5585390
C	-1.1081780	-2.6062300	-0.0804690
C	-1.6658960	-3.2089520	1.0593620
C	-2.4227630	-4.3801710	0.9597580
C	-2.6355100	-4.9743740	-0.2869520
C	-2.0826220	-4.3906320	-1.4305650
C	-1.3307560	-3.2176850	-1.3264640
H	-0.9052350	-2.7650480	-2.2185860
C	3.4895950	-0.6435700	0.1027510
C	4.0604720	-1.6943040	-0.6427830
C	5.4337810	-1.9426250	-0.6141180
C	6.2829220	-1.1384670	0.1519620
C	5.7362270	-0.0874630	0.8938070
C	4.3606470	0.1509140	0.8743310
H	1.3987300	3.1392190	-1.5772670
C	-1.9769310	0.6775010	-2.4642400
C	-1.9287600	0.1477020	2.7468480
H	5.8430930	-2.7589120	-1.2051410
H	3.4234120	-2.3101380	-1.2728030
H	3.9512710	0.9509730	1.4861320
H	6.3805160	0.5420710	1.5035020
H	7.3531060	-1.3282840	0.1707370

H	2.2955060	1.7337610	0.0809560
H	1.5229120	-2.4607560	0.0050700
H	-3.6657510	1.9191030	-0.6251970
H	-4.3988590	0.3773010	-1.1350900
H	-4.0191200	-0.6619740	0.9874660
H	-4.2299270	1.0237610	1.5195300
H	-1.4864420	-2.7647740	2.0347120
H	-2.8381490	-4.8321110	1.8575090
H	-3.2217730	-5.8864950	-0.3662090
H	-2.2375380	-4.8474870	-2.4052250
H	0.9829070	5.5585140	-1.8035430
H	-0.7910020	6.6626530	-0.4403010
H	-2.1161340	5.2990250	1.1709200
H	-1.6638600	2.8872680	1.4302500
H	-2.3883900	0.9190010	3.3801280
H	-2.3046680	-0.8335640	3.0758380
H	-0.8471430	0.1752990	2.9109850
H	-0.9807440	0.2985340	-2.7109690
H	-2.7016450	0.1907520	-3.1296100
H	-1.9925420	1.7609830	-2.6600490

9_ω_TS_b (B3LYP/6-31+G*)

N	2.2749850	0.4628180	-1.1259720
P	1.0661690	0.2549270	0.0553900
C	-0.2666560	1.4007510	-0.0250450
C	-1.5779270	0.8906100	0.0078520
C	-2.0020070	-0.4427250	0.0477830
C	-1.0547270	-1.4837900	0.0565050
C	0.3293010	-1.3403650	0.0618430
C	1.1961430	-2.5509600	-0.0304360
C	2.2776930	-2.7803740	0.8425070

C	3.0505330	-3.9402730	0.7446490
C	2.7611730	-4.9054510	-0.2235650
C	1.6886580	-4.6967140	-1.0957190
C	0.9227860	-3.5331460	-1.0041110
N	2.2039290	0.4733970	1.3081960
C	3.5583760	0.7831580	0.8698650
C	3.6342980	0.3420870	-0.6028420
C	-0.0286390	2.8727990	-0.0907420
C	-1.0939250	3.7954690	-0.2142660
C	-0.8777540	5.1720050	-0.2600110
C	0.4150490	5.6975980	-0.1907430
C	1.4858030	4.8096010	-0.0816820
C	1.2667440	3.4319260	-0.0351630
H	2.1290900	2.7836360	0.0238820
C	-3.4533440	-0.7624350	0.0839000
C	-4.3854620	-0.0272290	-0.6745970
C	-5.7489070	-0.3246250	-0.6356270
C	-6.2208230	-1.3777330	0.1534320
C	-5.3100430	-2.1243640	0.9063560
C	-3.9484020	-1.8171340	0.8757830
H	0.1046920	-3.3716640	-1.7015020
C	2.0579560	0.0741410	-2.5114770
C	1.8196990	0.8404520	2.6608650
H	-6.4424090	0.2589200	-1.2369450
H	-4.0338780	0.7707710	-1.3240530
H	-3.2605950	-2.3879050	1.4948830
H	-5.6612740	-2.9415300	1.5323800
H	-7.2816550	-1.6134120	0.1803190
H	-1.4309810	-2.5058720	0.0522690
H	-2.3749400	1.6261420	0.0319020
H	3.9980310	-0.6949050	-0.6832000

H	4.3162020	0.9854630	-1.1742340
H	3.7725450	1.8600510	0.9765730
H	4.2925050	0.2416460	1.4818920
H	-2.1187750	3.4495430	-0.2827890
H	-1.7328190	5.8375190	-0.3542920
H	0.5819100	6.7708090	-0.2273130
H	2.5055030	5.1855990	-0.0355740
H	1.4519510	-5.4366350	-1.8567340
H	3.3606550	-5.8093740	-0.2956050
H	3.8735900	-4.0957130	1.4386000
H	2.4954750	-2.0519970	1.6167310
H	2.4516290	0.3152270	3.3897270
H	1.9123820	1.9240280	2.8337000
H	0.7801030	0.5527860	2.8427310
H	1.0019660	0.1967530	-2.7691180
H	2.6448610	0.7247390	-3.1719220
H	2.3457330	-0.9717200	-2.7006090

9_θ_TS_a (B3LYP/6-31+G*)

N	2.2956100	0.1398270	1.2119990
P	1.0999000	0.0001650	0.0001270
C	0.0406780	-1.3890560	0.1331370
C	-1.3499350	-1.2121010	0.1108930
C	-2.0454770	-0.0003940	0.0000000
C	-1.3503480	1.2115480	-0.1110630
C	0.0401950	1.3889800	-0.1332930
C	0.6098370	2.7650790	-0.1627170
C	1.6988450	3.1219960	-0.9835060
C	2.1978190	4.4269650	-0.9961140
C	1.6204330	5.4163660	-0.1961300
C	0.5383690	5.0817990	0.6236530

C	0.0473570	3.7757470	0.6452800
N	2.2961240	-0.1390180	-1.2112700
C	3.6463780	-0.3325430	-0.6907620
C	3.6459730	0.3339710	0.6919980
C	0.6107650	-2.7649730	0.1624050
C	0.0484310	-3.7757690	-0.6455400
C	0.5398490	-5.0816690	-0.6240970
C	1.6221900	-5.4159530	0.1954360
C	2.1994470	-4.4264180	0.9953460
C	1.7000700	-3.1215990	0.9829300
H	2.1437680	-2.3710400	1.6287570
C	-3.5446490	-0.0006080	0.0000000
C	-4.2669850	0.1406590	1.1965660
C	-5.6650120	0.1407250	1.1992080
C	-6.3689520	-0.0008800	0.0000000
C	-5.6650540	-0.1423580	-1.1990990
C	-4.2670280	-0.1420220	-1.1965390
H	-0.7761760	3.5262840	1.3093230
C	1.9905960	0.6980900	2.5204570
C	1.9918930	-0.6973390	-2.5198830
H	-6.2034600	0.2508750	2.1377320
H	-3.7231910	0.2486100	2.1322730
H	-3.7232650	-0.2498580	-2.1322770
H	-6.2035360	-0.2526100	-2.1375910
H	-7.4562510	-0.0009600	0.0001030
H	-1.9492860	2.1172450	-0.2027270
H	-1.9485550	-2.1180180	0.2024550
H	3.8881730	1.4064760	0.6125530
H	4.3828870	-0.1330210	1.3587350
H	3.8890310	-1.4049390	-0.6112350
H	4.3833270	0.1347840	-1.3572250

H	-0.7753020	-3.5265080	-1.3094070
H	0.0821000	-5.8370340	-1.2587640
H	2.0083810	-6.4319670	0.2107130
H	3.0358830	-4.6722990	1.6459390
H	0.0805220	5.8370620	1.2583690
H	2.0063100	6.4324970	-0.2115500
H	3.0340340	4.6730650	-1.6469070
H	2.1426330	2.3715250	-1.6293740
H	2.6229670	-0.2222230	-3.2823800
H	2.1565280	-1.7853070	-2.5571470
H	0.9463780	-0.4979320	-2.7730540
H	0.9449980	0.4984440	2.7731050
H	2.6214000	0.2231510	3.2832870
H	2.1549400	1.7860990	2.5577670

9_θ_TS_b (B3LYP/6-31+G*)

N	2.3093070	-0.1244370	-1.2136840
P	1.1177480	0.0000000	0.0000000
C	0.0500510	1.3813310	-0.1237630
C	-1.3364550	1.2044200	-0.1030600
C	-2.0586470	0.0000000	0.0000000
C	-1.3364290	-1.2044560	0.1030350
C	0.0500810	-1.3813380	0.1237430
C	0.6163910	-2.7602150	0.1430310
C	1.6750460	-3.1358770	0.9938370
C	2.1705360	-4.4422780	0.9966490
C	1.6196110	-5.4126680	0.1556620
C	0.5678050	-5.0588030	-0.6946780
C	0.0803710	-3.7510110	-0.7058040
N	2.3092390	0.1244890	1.2137900
C	3.6600140	0.3249160	0.6954160

C	3.6600670	-0.3247910	-0.6952410
C	0.6163300	2.7602210	-0.1430770
C	0.0802880	3.7510190	0.7057420
C	0.5676900	5.0588240	0.6945890
C	1.6194820	5.4127000	-0.1557630
C	2.1704280	4.4423080	-0.9967340
C	1.6749710	3.1358940	-0.9938940
H	2.0972130	2.4001060	-1.6705160
C	-3.5526690	0.0000000	0.0000000
C	-4.3020770	1.1954320	-0.0684780
C	-5.6972480	1.1971130	-0.0684680
C	-6.4129000	0.0000000	0.0000000
C	-5.6972230	-1.1972400	0.0684820
C	-4.3020510	-1.1955300	0.0684820
H	-0.7181920	-3.4838740	-1.3933210
C	2.0037730	-0.6652440	-2.5299190
C	2.0036090	0.6652900	2.5300050
H	-6.2252020	2.1465860	-0.1228670
H	-3.8019000	2.1560930	-0.1234020
H	-3.8018550	-2.1561810	0.1234040
H	-6.2251570	-2.1467240	0.1228870
H	-7.4996940	0.0000000	0.0000000
H	-1.8995020	-2.1287050	0.1913720
H	-1.8995470	2.1286560	-0.1914160
H	3.9022990	-1.3980270	-0.6290120
H	4.3963040	0.1506070	-1.3564410
H	3.9021930	1.3981650	0.6292000
H	4.3962430	-0.1504430	1.3566540
H	-0.7182660	3.4838760	1.3932660
H	0.1316290	5.7994360	1.3610370
H	2.0028000	6.4298680	-0.1632760

H	2.9824670	4.7047470	-1.6712080
H	0.1317610	-5.7994130	-1.3611380
H	2.0029540	-6.4298270	0.1631540
H	2.9825850	-4.7047090	1.6711140
H	2.0972710	-2.4000910	1.6704710
H	2.6359830	0.1814480	3.2856970
H	2.1663170	1.7528440	2.5810740
H	0.9586580	0.4605130	2.7810210
H	0.9588340	-0.4604900	-2.7810010
H	2.6361850	-0.1813850	-3.2855690
H	2.1665100	-1.7527930	-2.5809790

10_a (B3LYP/6-31+G*)

N	0.1914520	-2.3196440	1.2055030
P	0.0000540	-1.1270130	0.0001420
C	-1.3714900	-0.0619380	0.2121610
C	-1.2036140	1.3257090	0.1714970
C	-0.0001710	2.0293680	0.0000200
C	1.2033640	1.3258690	-0.1715080
C	1.3714180	-0.0617520	-0.2121590
C	2.7316260	-0.6485360	-0.3643120
C	3.0588970	-1.5743630	-1.3761140
C	4.3427890	-2.1080290	-1.5046050
C	5.3546260	-1.7255270	-0.6199210
C	5.0703860	-0.8106600	0.3962650
C	3.7816980	-0.3028140	0.5022300
N	-0.1911750	-2.3199920	-1.2048330
C	-0.3644330	-3.6696060	-0.6741560
C	0.3655080	-3.6692640	0.6750800
C	-2.7316380	-0.6489110	0.3641030
C	-3.0589070	-1.5749150	1.3757470

C	-4.3427450	-2.1087680	1.5040140
C	-5.3545290	-1.7262880	0.6192590
C	-5.0702830	-0.8112650	-0.3967850
C	-3.7816510	-0.3032340	-0.5025330
F	-3.5398470	0.5687460	-1.5226180
C	-0.0002630	3.5162650	0.0000280
C	-1.0323950	4.2457880	-0.6212900
C	-1.0363290	5.6419220	-0.6163150
C	-0.0004230	6.3499170	0.0000390
C	1.0355620	5.6420360	0.6163900
C	1.0317860	4.2459010	0.6213540
F	3.5399040	0.5690410	1.5224220
C	0.8123270	-2.0147800	2.4865190
C	-0.8119680	-2.0157460	-2.4860210
H	-1.8448230	6.1775270	-1.1088110
H	-1.8285080	3.7123630	-1.1348970
H	1.8279580	3.7125620	1.1349580
H	1.8439950	6.1777290	1.1088900
H	-0.0004850	7.4370230	0.0000430
H	2.1061380	1.9158430	-0.3177370
H	-2.1064790	1.9155640	0.3176350
H	1.4340630	-3.9070560	0.5437990
H	-0.0662120	-4.4084780	1.3621580
H	-1.4328520	-3.9079860	-0.5428470
H	0.0677240	-4.4087030	-1.3610870
H	-2.2823970	-1.8651530	2.0755880
H	-4.5544530	-2.8133740	2.3039920
H	-6.3582560	-2.1307830	0.7173220
H	-5.8250540	-0.4908700	-1.1086350
H	5.8252040	-0.4902430	1.1080540
H	6.3583940	-2.1298790	-0.7181530

H	4.5544960	-2.8125050	-2.3046990
H	2.2823480	-1.8646050	-2.0759110
H	-0.3565020	-2.6279460	-3.2754240
H	-1.8957970	-2.2085090	-2.4780590
H	-0.6543880	-0.9625730	-2.7356660
H	0.6539380	-0.9617150	2.7361120
H	0.3575160	-2.6272820	3.2760590
H	1.8963080	-2.2066690	2.4783390

10_a (B3LYP/6-31+G*, excited state minima)

N	0.2014260	-2.2948570	1.2141000
P	0.0000200	-1.1287340	0.0000790
C	-1.4215820	-0.0746190	0.2150790
C	-1.2307220	1.3109430	0.2059320
C	-0.0000970	2.0003040	-0.0000420
C	1.2305680	1.3110210	-0.2060500
C	1.4215070	-0.0745270	-0.2152120
C	2.7525960	-0.6825680	-0.3241780
C	3.0318160	-1.8395500	-1.1124730
C	4.3072230	-2.3893590	-1.1950660
C	5.3842020	-1.8168970	-0.5036930
C	5.1542270	-0.6690350	0.2755950
C	3.8795970	-0.1408850	0.3463350
N	-0.2012410	-2.2952540	-1.2135370
C	-0.3738690	-3.6467460	-0.6688550
C	0.3746860	-3.6464070	0.6697550
C	-2.7526270	-0.6827540	0.3239550
C	-3.0317680	-1.8398620	1.1120930
C	-4.3071280	-2.3898000	1.1945690
C	-5.3841340	-1.8173490	0.5032310
C	-5.1542350	-0.6693730	-0.2759110

C	-3.8796500	-0.1410960	-0.3465400
F	-3.7058960	0.9492650	-1.1570070
C	-0.0001380	3.4636710	-0.0000180
C	-1.1647150	4.2033950	-0.3344580
C	-1.1604740	5.5943370	-0.3393280
C	-0.0002110	6.3013130	0.0000330
C	1.1600900	5.5943840	0.3393650
C	1.1644020	4.2034430	0.3344470
F	3.7057670	0.9493650	1.1569370
C	0.8750060	-1.9801410	2.4695170
C	-0.8748930	-1.9811430	-2.4690620
H	-2.0640000	6.1316460	-0.6157920
H	-2.0652300	3.6788330	-0.6360240
H	2.0649450	3.6789140	0.6359880
H	2.0635880	6.1317300	0.6158470
H	-0.0002380	7.3880700	0.0000520
H	2.1078140	1.9126290	-0.4121450
H	-2.1080110	1.9124960	0.4120010
H	1.4410650	-3.8786080	0.5281220
H	-0.0511910	-4.3818730	1.3637940
H	-1.4401440	-3.8793900	-0.5271740
H	0.0523440	-4.3821960	-1.3627060
H	-2.2221140	-2.2808630	1.6826490
H	-4.4676420	-3.2658520	1.8188400
H	-6.3813620	-2.2419450	0.5688570
H	-5.9507870	-0.1903700	-0.8376810
H	5.9507570	-0.1900300	0.8373950
H	6.3814680	-2.2413900	-0.5694150
H	4.4677940	-3.2653180	-1.8194540
H	2.2221800	-2.2805630	-1.6830440
H	-0.5074500	-2.6538430	-3.2533940

H	-1.9667040	-2.0860170	-2.3900300
H	-0.6445310	-0.9548780	-2.7688410
H	0.6441510	-0.9539250	2.7690870
H	0.5079290	-2.6528520	3.2540080
H	1.9668670	-2.0844950	2.3904530

10_b (B3LYP/6-31+G*)

N	-2.2446480	0.0892550	-1.1437380
P	-0.9976630	0.1973250	0.0047260
C	-0.2005920	-1.3728020	0.0461900
C	1.1937590	-1.4704100	0.0339280
C	2.1234210	-0.4183940	-0.0104720
C	1.6629490	0.9078600	-0.0544060
C	0.3357070	1.3480170	-0.0572560
C	0.0861960	2.8135050	0.0466070
C	0.7081310	3.5738710	1.0591640
C	0.5320760	4.9540770	1.1688240
C	-0.2910670	5.6292570	0.2631830
C	-0.9223150	4.9127500	-0.7556150
C	-0.7171190	3.5417580	-0.8390690
N	-2.0876080	0.7621200	1.1787410
C	-3.4918700	0.6528440	0.7991120
C	-3.4945490	0.7209070	-0.7349060
C	-0.9910770	-2.6314540	-0.0627230
C	-0.7259890	-3.5545130	-1.0955550
C	-1.4167370	-4.7616430	-1.2111460
C	-2.4163020	-5.0873820	-0.2903010
C	-2.7067240	-4.2006360	0.7485200
C	-1.9942680	-3.0120160	0.8362350
F	-2.2862620	-2.1869950	1.8853290
C	3.5802200	-0.7072680	-0.0069620

C	4.1128310	-1.7666060	0.7548820
C	5.4812150	-2.0431690	0.7560400
C	6.3622400	-1.2586240	0.0059640
C	5.8534600	-0.1989200	-0.7510780
C	4.4829350	0.0670100	-0.7626850
F	-1.3240290	2.8849210	-1.8720950
C	-2.0044040	-0.1112390	-2.5623720
C	-1.7584120	0.8526850	2.5905630
H	5.8609670	-2.8659050	1.3583260
H	3.4492280	-2.3656690	1.3739470
H	4.1033050	0.8726020	-1.3865350
H	6.5238130	0.4157240	-1.3482800
H	7.4285560	-1.4694880	0.0110790
H	2.4173050	1.6937370	-0.0599970
H	1.5931300	-2.4837570	0.0335680
H	-3.5510790	1.7628440	-1.0869700
H	-4.3447210	0.1701180	-1.1574150
H	-3.9321590	-0.2908680	1.1574140
H	-4.0611090	1.4834460	1.2360940
H	0.0388110	-3.2995760	-1.8241700
H	-1.1806430	-5.4417920	-2.0249330
H	-2.9634490	-6.0222350	-0.3735690
H	-3.4627200	-4.4215420	1.4958420
H	-1.5556990	5.3987690	-1.4917370
H	-0.4394550	6.7027140	0.3422750
H	1.0300960	5.4986200	1.9666720
H	1.3360090	3.0528180	1.7771140
H	-2.1834850	1.7727650	3.0128920
H	-2.1388030	-0.0086940	3.1573820
H	-0.6721610	0.8940140	2.7136360
H	-1.0166600	-0.5576140	-2.7096290

H	-2.7537500	-0.8012120	-2.9722030
H	-2.0442530	0.8325610	-3.1238290

10_c (B3LYP/6-31+G*)

N	0.1690370	-2.3126680	1.2089650
P	0.0007730	-1.1206460	0.0002740
C	-1.3753220	-0.0564220	0.1827550
C	-1.2084230	1.3310360	0.1448930
C	-0.0017160	2.0360160	0.0000190
C	1.2061230	1.3328940	-0.1449650
C	1.3752270	-0.0542470	-0.1827390
C	2.7396370	-0.6441340	-0.2804620
C	3.1044490	-1.5568540	-1.2910050
C	4.3902390	-2.0952720	-1.3732020
C	5.3650000	-1.7304620	-0.4406060
C	5.0418700	-0.8302600	0.5770360
C	3.7518500	-0.3175120	0.6365810
N	-0.1656100	-2.3136740	-1.2075510
C	-0.3466400	-3.6638940	-0.6812930
C	0.3547680	-3.6624660	0.6831820
C	-2.7388340	-0.6485000	0.2800220
C	-3.1024450	-1.5621590	1.2901400
C	-4.3874230	-2.1026160	1.3718430
C	-5.3625540	-1.7389590	0.4391980
C	-5.0406080	-0.8378560	-0.5780290
C	-3.7513700	-0.3231020	-0.6371270
F	-3.4694850	0.5320010	-1.6597100
C	-0.0028400	3.5222950	0.0000260
C	-0.8500810	4.2526990	0.8558720
C	-0.8565900	5.6487240	0.8520760
C	-0.0050530	6.3578290	0.0000370

C	0.8475910	5.6500530	-0.8520140
C	0.8432560	4.2540260	-0.8558180
F	3.4688390	0.5367420	1.6595570
C	0.7557710	-2.0079710	2.5058940
C	-0.7529060	-2.0114300	-2.5047510
H	-1.5196450	6.1833670	1.5285210
H	-1.4949240	3.7203570	1.5508190
H	1.4889160	3.7226870	-1.5507790
H	1.5098000	6.1857500	-1.5284490
H	-0.0058950	7.4448720	0.0000410
H	2.1175980	1.9228720	-0.2217610
H	-2.1208660	1.9195290	0.2215250
H	1.4258490	-3.9003900	0.5748290
H	-0.0914130	-4.4010610	1.3616040
H	-1.4169140	-3.9054170	-0.5729350
H	0.1020840	-4.4012380	-1.3594170
H	-2.3535820	-1.8375990	2.0253980
H	-4.6289610	-2.7974090	2.1719470
H	-6.3672600	-2.1483080	0.5005130
H	-5.7662440	-0.5334630	-1.3262580
H	5.7671790	-0.5250220	1.3252400
H	6.3703210	-2.1382430	-0.5022830
H	4.6326950	-2.7893770	-2.1736260
H	2.3558540	-1.8331140	-2.0262360
H	-0.2718340	-2.6189400	-3.2825750
H	-1.8351690	-2.2115580	-2.5267420
H	-0.5963860	-0.9566360	-2.7480000
H	0.5939520	-0.9541060	2.7497080
H	0.2781670	-2.6182750	3.2836430
H	1.8390450	-2.2026080	2.5272540

10_d (B3LYP/6-31+G*)

N	1.4000010	-1.7065030	1.4590270
P	0.7417920	-0.7783850	0.1932200
C	-1.0083550	-0.9138210	0.1800420
C	-1.8168540	0.2225520	0.1966330
C	-1.3886250	1.5619070	0.2048050
C	-0.0197480	1.8517200	0.1024140
C	1.0475920	0.9436870	0.0413900
C	2.3924180	1.4732840	-0.3201420
C	2.5310030	2.3408210	-1.4251330
C	3.7576720	2.8971470	-1.7887640
C	4.9094070	2.5922230	-1.0580450
C	4.8154780	1.7366410	0.0410410
C	3.5762030	1.2089970	0.3790680
N	1.6303760	-1.6516170	-0.9624990
C	2.2608960	-2.8583660	-0.4329990
C	2.5055370	-2.5692010	1.0550490
C	-1.6322790	-2.2653630	0.0838700
C	-1.4405590	-3.2782870	1.0444230
C	-2.0492550	-4.5300320	0.9320340
C	-2.8815010	-4.8113240	-0.1546900
C	-3.0965670	-3.8339350	-1.1284700
C	-2.4723190	-2.6001370	-0.9905270
F	-2.6902950	-1.6804990	-1.9726470
C	-2.3793230	2.6666920	0.2799150
C	-3.6112960	2.5937680	-0.3998870
C	-4.5397830	3.6336680	-0.3297170
C	-4.2594160	4.7845540	0.4131990
C	-3.0398890	4.8771230	1.0896800
C	-2.1173620	3.8305540	1.0287910
F	3.5285390	0.4028090	1.4821850

C	1.2987720	-1.3162090	2.8553910
C	1.2746010	-1.6377490	-2.3733390
H	-5.4797430	3.5499550	-0.8705700
H	-3.8335050	1.7232920	-1.0121230
H	-1.1890670	3.9057840	1.5899320
H	-2.8098980	5.7610140	1.6803870
H	-4.9805970	5.5963570	0.4640820
H	0.2558610	2.9024800	0.0221480
H	-2.8897790	0.0415300	0.2319200
H	3.4754240	-2.0730640	1.2086580
H	2.4952070	-3.4944810	1.6458180
H	1.6072150	-3.7367710	-0.5622860
H	3.2023910	-3.0532660	-0.9621230
H	-0.8066340	-3.0633090	1.8973830
H	-1.8815590	-5.2804080	1.7001900
H	-3.3640760	-5.7806520	-0.2458160
H	-3.7321820	-4.0121550	-1.9905790
H	5.6803470	1.4835500	0.6469090
H	5.8712000	3.0146450	-1.3354380
H	3.8139260	3.5586850	-2.6490830
H	1.6443490	2.5651680	-2.0117970
H	2.1817310	-1.7226870	-2.9847280
H	0.5935120	-2.4618820	-2.6374180
H	0.7825100	-0.6934390	-2.6222160
H	0.4285210	-0.6672980	2.9954890
H	1.1610830	-2.2082460	3.4820280
H	2.1913540	-0.7784500	3.2031830

10_e (B3LYP/6-31+G*)

N	0.8297440	-2.0688860	1.1595310
P	0.5666500	-0.9016080	-0.0455770

C	-1.1665710	-0.6839800	-0.2181860
C	-1.7431990	0.5851500	-0.1904150
C	-1.0694240	1.8145590	-0.0806630
C	0.3318050	1.8326660	-0.0084090
C	1.2042070	0.7357270	-0.0023590
C	2.6660570	1.0037880	-0.0998940
C	3.1643850	1.8733610	-1.0933100
C	4.5204200	2.1847900	-1.2000210
C	5.4407910	1.6210910	-0.3119660
C	4.9878230	0.7561920	0.6861590
C	3.6300660	0.4760790	0.7679900
N	1.4675120	-1.8115650	-1.1653960
C	1.8177110	-3.1539660	-0.7122550
C	1.8330460	-3.0718120	0.8223070
C	-2.0305230	-1.8948590	-0.3040430
C	-1.9278540	-2.8237120	-1.3564910
C	-2.7446210	-3.9537580	-1.4308540
C	-3.7062030	-4.1875100	-0.4438590
C	-3.8396350	-3.2877550	0.6157850
C	-3.0054000	-2.1775590	0.6637340
F	-3.1472740	-1.3348150	1.7245400
C	-1.8345190	3.0877770	-0.0530880
C	-2.9761390	3.2739160	-0.8572240
C	-3.6974900	4.4686840	-0.8272190
C	-3.2910640	5.5199500	-0.0001690
C	-2.1572400	5.3558560	0.8006480
C	-1.4445140	4.1554400	0.7789160
F	3.2269460	-0.3459860	1.7821530
C	0.5362360	-1.8269900	2.5633890
C	1.4990640	-1.5281850	-2.5874900
H	-4.5726120	4.5826250	-1.4630120

H	-3.2893250	2.4811250	-1.5321540
H	-0.5874780	4.0332600	1.4366300
H	-1.8337870	6.1593360	1.4585400
H	-3.8502580	6.4518940	0.0210470
H	0.8107760	2.8106040	0.0200070
H	-2.8305080	0.6147620	-0.2376820
H	2.8286960	-2.7862990	1.1954780
H	1.5619680	-4.0351170	1.2731120
H	1.0829910	-3.8984600	-1.0605330
H	2.8018200	-3.4375580	-1.1070260
H	-1.2005490	-2.6351810	-2.1409390
H	-2.6383830	-4.6425430	-2.2647870
H	-4.3511180	-5.0604580	-0.4961710
H	-4.5701670	-3.4355770	1.4054160
H	5.6640850	0.3092770	1.4086910
H	6.4995240	1.8525960	-0.3889130
H	4.8571600	2.8596740	-1.9824210
H	2.4576740	2.3013740	-1.7992590
H	2.5189050	-1.6514450	-2.9747250
H	0.8310590	-2.1922640	-3.1605590
H	1.1900700	-0.4942820	-2.7666110
H	-0.2497670	-1.0712420	2.6510300
H	0.1678680	-2.7512580	3.0267390
H	1.4204650	-1.4800020	3.1165000

10_f (B3LYP/6-31+G*)

N	1.2286490	-1.9638430	-0.9687380
P	0.5904240	-0.8914820	0.1792600
C	1.1787520	0.7521250	0.0620890
C	0.2925490	1.8310580	0.0739920
C	-1.1119080	1.7740740	0.1283380

C	-1.7556620	0.5265790	0.1744380
C	-1.1521430	-0.7343490	0.2114460
C	-1.9895470	-1.9666840	0.1893310
C	-1.9116270	-2.9598720	1.1862630
C	-2.7109010	-4.1044460	1.1551260
C	-3.6283800	-4.2915090	0.1177860
C	-3.7346830	-3.3301740	-0.8896040
C	-2.9196970	-2.2059350	-0.8350480
N	1.2319270	-1.8255540	1.4604490
C	2.1979500	-2.8295130	1.0227720
C	1.7727230	-3.1947050	-0.4051000
C	2.6504680	1.0112490	0.0554610
C	3.2967520	1.5556840	1.1820750
C	4.6643780	1.8370890	1.1888240
C	5.4352900	1.5726500	0.0532290
C	4.8281310	1.0434990	-1.0871780
C	3.4623020	0.7824690	-1.0634370
F	2.8992060	0.3132420	-2.2094290
C	-1.9109140	3.0268620	0.1259600
C	-1.5387800	4.1324530	-0.6636580
C	-2.2867890	5.3112760	-0.6598030
C	-3.4387700	5.4158860	0.1251320
C	-3.8278310	4.3265880	0.9100320
C	-3.0712480	3.1533270	0.9147030
F	-3.0339030	-1.3045670	-1.8496310
C	0.7655070	-1.9916180	-2.3479830
C	1.3764250	-1.2893340	2.8038820
H	-1.9761120	6.1450260	-1.2854170
H	-0.6672590	4.0582660	-1.3094010
H	-3.3717540	2.3294300	1.5574270
H	-4.7169000	4.3937540	1.5330750

H	-4.0248260	6.3314450	0.1242330
H	-2.8442600	0.5292810	0.1640840
H	0.7526150	2.8183280	0.0478510
H	1.0156520	-3.9963130	-0.3972000
H	2.6277580	-3.5387950	-1.0009140
H	3.2250130	-2.4278860	1.0369930
H	2.1658020	-3.7032860	1.6863990
H	2.6972080	1.7603390	2.0652390
H	5.1252780	2.2596040	2.0776210
H	6.5010290	1.7848640	0.0477970
H	5.3892230	0.8453400	-1.9954480
H	-4.4297510	-3.4395320	-1.7166150
H	-4.2597090	-5.1755160	0.0900700
H	-2.6246510	-4.8429150	1.9479510
H	-1.2114850	-2.8129610	2.0019700
H	1.2467880	-2.0922030	3.5416770
H	2.3626050	-0.8266730	2.9650940
H	0.6062980	-0.5335360	2.9863610
H	0.3570210	-1.0159340	-2.6225730
H	1.6072460	-2.2061700	-3.0174970
H	-0.0150310	-2.7523970	-2.5025370

10_g (B3LYP/6-31+G*)

N	-1.1737850	-1.9989610	0.9622930
P	-0.5783980	-0.9040000	-0.1881710
C	-1.2019080	0.7242680	-0.0430920
C	-0.3425120	1.8241340	-0.0597590
C	1.0621120	1.7992960	-0.1405730
C	1.7326920	0.5679340	-0.2097150
C	1.1589880	-0.7075610	-0.2492750
C	2.0283500	-1.9168910	-0.2780430

C	1.9354490	-2.9057560	-1.2781960
C	2.7663900	-4.0278790	-1.2897800
C	3.7321470	-4.1968240	-0.2940760
C	3.8550390	-3.2394370	0.7152400
C	3.0083550	-2.1379300	0.7034890
N	-1.2242400	-1.8395630	-1.4665840
C	-2.1637560	-2.8647810	-1.0205710
C	-1.7084390	-3.2334390	0.3971050
C	-2.6783060	0.9430010	0.0368640
C	-3.3972370	1.4533590	-1.0609780
C	-4.7711230	1.6958000	-1.0000340
C	-5.4738090	1.4260480	0.1777140
C	-4.7935560	0.9295170	1.2912280
C	-3.4239740	0.7061180	1.1991850
F	-2.7893830	0.2645830	2.3178940
C	1.8321340	3.0705320	-0.1366300
C	1.3719970	4.2062800	-0.8311040
C	2.0915300	5.4028060	-0.8233890
C	3.3014140	5.4956110	-0.1295930
C	3.7767710	4.3769000	0.5611150
C	3.0497190	3.1851740	0.5625230
F	3.1427740	-1.2397180	1.7198830
C	-0.6668810	-2.0410170	2.3260830
C	-1.4076100	-1.2908110	-2.8002190
H	1.7115020	6.2607670	-1.3735310
H	0.4497950	4.1442580	-1.4038780
H	3.4181000	2.3376580	1.1350750
H	4.7109320	4.4350840	1.1152010
H	3.8648480	6.4252610	-0.1267900
H	2.8192430	0.5975580	-0.2677470
H	-0.8208840	2.7982300	0.0373380

H	-0.9388040	-4.0226580	0.3705250
H	-2.5476820	-3.5964810	1.0040810
H	-3.1982040	-2.4823030	-1.0147370
H	-2.1269620	-3.7329820	-1.6912750
H	-2.8502440	1.6623480	-1.9766910
H	-5.2896650	2.0927170	-1.8686410
H	-6.5434380	1.6085880	0.2364200
H	-5.3010920	0.7276420	2.2296700
H	4.5873240	-3.3351180	1.5112440
H	4.3878950	-5.0632640	-0.3001520
H	2.6666530	-4.7628200	-2.0842990
H	1.1976390	-2.7734330	-2.0623910
H	-1.2760700	-2.0818220	-3.5503660
H	-2.4068920	-0.8482330	-2.9356400
H	-0.6579780	-0.5162490	-2.9891070
H	-0.2617600	-1.0646900	2.6030660
H	-1.4839240	-2.2766100	3.0186480
H	0.1274350	-2.7942710	2.4434900

10_h (B3LYP/6-31+G*)

N	0.1115750	-2.2457740	-1.1438000
P	-0.1174400	-1.0137000	0.0034560
C	1.3823800	-0.0936710	0.0776710
C	1.3683470	1.3048960	0.0638970
C	0.2461970	2.1465790	-0.0025450
C	-1.0376320	1.5813660	-0.0703620
C	-1.3688600	0.2225280	-0.0822940
C	-2.8124050	-0.1451770	-0.0383450
C	-3.6530920	0.3930400	0.9582870
C	-5.0182700	0.1094660	1.0155150
C	-5.5962500	-0.7424430	0.0702440

C	-4.7981790	-1.2950730	-0.9334100
C	-3.4449250	-0.9840110	-0.9638810
N	-0.6195410	-2.1482320	1.1645030
C	-0.3890300	-3.5379720	0.7863880
C	-0.4255210	-3.5432000	-0.7490190
C	2.7050560	-0.7786340	0.0295030
C	3.6416450	-0.4444080	-0.9709330
C	4.9076340	-1.0282160	-1.0322870
C	5.2821600	-1.9878090	-0.0877040
C	4.3836700	-2.3460090	0.9192710
C	3.1354230	-1.7380490	0.9537630
F	2.3004170	-2.0928170	1.9748820
C	0.4162250	3.6233970	-0.0011140
C	1.4734420	4.2393660	-0.6991750
C	1.6381570	5.6256060	-0.6931020
C	0.7414530	6.4414250	0.0028360
C	-0.3176540	5.8493860	0.6969100
C	-0.4730900	4.4620370	0.6990160
F	-2.7082150	-1.5189810	-1.9820910
C	0.3276460	-1.9879730	-2.5570670
C	-0.7660270	-1.8304280	2.5745370
H	2.4623170	6.0700350	-1.2465230
H	2.1625980	3.6259340	-1.2743610
H	-1.2836250	4.0200490	1.2731980
H	-1.0189840	6.4682800	1.2520590
H	0.8661580	7.5212410	0.0044470
H	-1.8787850	2.2694610	-0.1428680
H	2.3440250	1.7840990	0.1332430
H	-1.4517050	-3.6835740	-1.1237590
H	0.2010970	-4.3452090	-1.1599580
H	0.5800630	-3.9007320	1.1632030

H	-1.1792440	-4.1734020	1.2063850
H	3.3499060	0.2875140	-1.7194000
H	5.5957870	-0.7399860	-1.8224410
H	6.2634440	-2.4524920	-0.1283310
H	4.6387060	-3.0740980	1.6833850
H	-5.2081330	-1.9479450	-1.6981200
H	-6.6571160	-0.9740630	0.1076580
H	-5.6263360	0.5465190	1.8030990
H	-3.2067570	1.0414600	1.7075280
H	-1.6519350	-2.3372060	2.9792280
H	0.1146590	-2.1313680	3.1588370
H	-0.9073440	-0.7524090	2.6961140
H	0.7069080	-0.9708080	-2.6917300
H	1.0780750	-2.6857620	-2.9511840
H	-0.5972260	-2.0919230	-3.1411760

10_i (B3LYP/6-31+G*)

N	0.7086050	-2.1159410	1.1486270
P	0.5268420	-0.9287980	-0.0516340
C	-1.1891590	-0.6240100	-0.2575210
C	-1.7018160	0.6727200	-0.2277260
C	-0.9705250	1.8662530	-0.0957420
C	0.4282380	1.8147260	0.0027660
C	1.2433610	0.6743300	0.0192160
C	2.7198990	0.8679520	-0.0116380
C	3.3038590	1.7023340	-0.9882650
C	4.6766650	1.9478170	-1.0362200
C	5.5276080	1.3500430	-0.1024070
C	4.9890910	0.5180100	0.8810710
C	3.6170040	0.3035760	0.9036120
N	1.4082660	-1.8734900	-1.1580940

C	1.6870510	-3.2332550	-0.7078180
C	1.6721330	-3.1616800	0.8273880
C	-2.1091670	-1.7870930	-0.3947880
C	-2.0110900	-2.7135100	-1.4501010
C	-2.8815790	-3.7987570	-1.5680370
C	-3.8944010	-3.9889340	-0.6238620
C	-4.0252990	-3.0903820	0.4371200
C	-3.1375850	-2.0255700	0.5287310
F	-3.2808860	-1.1831130	1.5910370
C	-1.6787710	3.1729950	-0.0633680
C	-2.9022430	3.3188880	0.6192600
C	-3.5738130	4.5425040	0.6503520
C	-3.0353420	5.6616280	0.0087910
C	-1.8192700	5.5372420	-0.6693800
C	-1.1553370	4.3098170	-0.7094000
F	3.1301870	-0.4883730	1.9043580
C	0.3728410	-1.8826020	2.5441340
C	1.4997900	-1.5759690	-2.5743960
H	-4.5140190	4.6240780	1.1910510
H	-3.3192900	2.4688020	1.1534270
H	-0.2271980	4.2264480	-1.2696170
H	-1.3911560	6.3956770	-1.1821470
H	-3.5550350	6.6159930	0.0369170
H	0.9525670	2.7645680	0.0984860
H	-2.7808100	0.7604740	-0.3435150
H	2.6714320	-2.9257710	1.2249740
H	1.3468770	-4.1141990	1.2650940
H	0.9269700	-3.9410520	-1.0773350
H	2.6658520	-3.5586160	-1.0830960
H	-1.2432690	-2.5582240	-2.2023960
H	-2.7766510	-4.4863240	-2.4031260

H	-4.5810050	-4.8265970	-0.7106820
H	-4.7949470	-3.2051490	1.1944380
H	5.6099830	0.0470750	1.6372600
H	6.5986260	1.5301200	-0.1326720
H	5.0806210	2.5978830	-1.8077620
H	2.6501390	2.1561110	-1.7284570
H	2.5267110	-1.7347690	-2.9288710
H	0.8262120	-2.2065110	-3.1779750
H	1.2369110	-0.5288750	-2.7503510
H	-0.3869080	-1.0985870	2.6137010
H	-0.0475440	-2.7984800	2.9791560
H	1.2476330	-1.5771410	3.1350760

10_j (B3LYP/6-31+G*)

N	-0.3425630	-2.3126070	1.1610840
P	-0.0004580	-1.1177840	0.0001550
C	-1.3718290	-0.0457420	-0.1841720
C	-1.2050330	1.3415590	-0.1511010
C	0.0008010	2.0463430	-0.0000040
C	1.2060710	1.3406190	0.1510170
C	1.3718030	-0.0468320	0.1840870
C	2.7311120	-0.6346170	0.3381860
C	3.0708200	-1.4964960	1.3984390
C	4.3498260	-2.0400860	1.5323900
C	5.3436110	-1.7285520	0.6004410
C	5.0462820	-0.8760430	-0.4650090
C	3.7614230	-0.3583450	-0.5727590
N	0.3404570	-2.3133090	-1.1603390
C	-0.0203310	-3.6702330	-0.7679630
C	0.0170200	-3.6699870	0.7691790
C	-2.7315540	-0.6325210	-0.3385040

C	-3.0717390	-1.4939460	-1.3989700
C	-4.3511200	-2.0365900	-1.5331860
C	-5.3447860	-1.7245340	-0.6012830
C	-5.0469730	-0.8724570	0.4643790
C	-3.7617590	-0.3556980	0.5723820
F	-3.5022320	0.4543880	1.6379290
C	0.0013750	3.5330100	0.0000280
C	-1.0222490	4.2631830	0.6346020
C	-1.0256940	5.6592950	0.6297010
C	0.0024690	6.3669240	0.0001050
C	1.0300830	5.6585380	-0.6295240
C	1.0255670	4.2624260	-0.6345060
F	3.5023680	0.4521480	-1.6381150
C	-0.5690680	-2.0216990	2.5644030
C	0.5678080	-2.0231620	-2.5636730
H	-1.8276200	6.1952170	1.1324840
H	-1.8117680	3.7303010	1.1588020
H	1.8146800	3.7289690	-1.1587290
H	1.8324280	6.1938660	-1.1322750
H	0.0028860	7.4540240	0.0001360
H	2.1129200	1.9281900	0.2835110
H	-2.1114050	1.9298470	-0.2836970
H	1.0192160	-3.9439890	1.1379240
H	-0.7045120	-4.3849580	1.1848160
H	-1.0227690	-3.9434520	-1.1366200
H	0.7005630	-4.3859890	-1.1833550
H	-2.3130840	-1.7204790	-2.1421780
H	-4.5744130	-2.6904160	-2.3721040
H	-6.3456920	-2.1353770	-0.7014240
H	-5.7886570	-0.6082430	1.2121430
H	5.7880590	-0.6121890	-1.2128070

H	6.3442350	-2.1401300	0.7003850
H	4.5727440	-2.6942430	2.3711490
H	2.3121040	-1.7226390	2.1417030
H	1.3978240	-2.6322900	-2.9449840
H	-0.3209530	-2.2278360	-3.1830740
H	0.8373220	-0.9699320	-2.6849030
H	-0.8378150	-0.9682340	2.6852990
H	-1.3993270	-2.6301010	2.9463320
H	0.3198220	-2.2267150	3.1835100

10_k (B3LYP/6-31+G*)

N	-0.1389480	-2.2190110	1.2062060
P	-0.0009630	-1.0277110	-0.0005640
C	-1.3784270	0.0475510	-0.0697720
C	-1.2113300	1.4338510	-0.0714320
C	0.0024200	2.1410370	-0.0008690
C	1.2146310	1.4312470	0.0698460
C	1.3788350	0.0445790	0.0682190
C	2.7498000	-0.5378680	0.2031140
C	3.4032190	-0.5893900	1.4484580
C	4.6923980	-1.1085130	1.5873780
C	5.3694870	-1.5999570	0.4677400
C	4.7572980	-1.5566810	-0.7864070
C	3.4751000	-1.0285000	-0.8897840
N	0.1347660	-2.2197850	-1.2071120
C	-0.1762460	-3.5693990	-0.7478820
C	0.1660330	-3.5700300	0.7472520
C	-2.7507060	-0.5321670	-0.2031290
C	-3.4043570	-0.5860840	-1.4482210
C	-4.6948220	-1.1025250	-1.5853070
C	-5.3729480	-1.5887320	-0.4640130

C	-4.7604280	-1.5431430	0.7898970
C	-3.4769080	-1.0178080	0.8913690
F	-2.9248480	-0.9563840	2.1339210
C	0.0040240	3.6265590	-0.0008900
C	-0.9755500	4.3591140	0.6981930
C	-0.9787420	5.7551930	0.6932650
C	0.0071960	6.4625670	-0.0008820
C	0.9915500	5.7529990	-0.6950370
C	0.9852320	4.3569320	-0.6999740
F	2.9238570	-0.9689130	-2.1327700
C	0.0315590	-1.9249250	2.6194940
C	-0.0367320	-1.9252330	-2.6202300
H	-1.7462080	6.2914680	1.2469380
H	-1.7293110	3.8275500	1.2737990
H	1.7378080	3.8236700	-1.2755600
H	1.7602170	6.2875620	-1.2486980
H	0.0084150	7.5496370	-0.0008710
H	2.1318150	2.0140360	0.1495050
H	-2.1272840	2.0185930	-0.1509350
H	1.2290610	-3.8167080	0.9078540
H	-0.4394260	-4.3020060	1.2969730
H	-1.2403860	-3.8113350	-0.9083500
H	0.4258680	-4.3041850	-1.2975090
H	-2.8765320	-0.2063750	-2.3190320
H	-5.1677050	-1.1247590	-2.5634410
H	-6.3766650	-1.9942750	-0.5591090
H	-5.2620620	-1.8956030	1.6861050
H	5.2582770	-1.9129810	-1.6814630
H	6.3721780	-2.0076890	0.5642700
H	5.1650690	-1.1288210	2.5656590
H	2.8761680	-0.2057020	2.3179960

H	0.6501690	-2.5402100	-3.2145210
H	-1.0667190	-2.1192360	-2.9586050
H	0.2052100	-0.8765720	-2.8115910
H	-0.2065430	-0.8752820	2.8102790
H	-0.6586040	-2.5370420	3.2129750
H	1.0602950	-2.1228730	2.9593840

10_l (B3LYP/6-31+G*)

N	1.4006160	-1.7071460	1.4492430
P	0.7512660	-0.7681300	0.1871100
C	-0.9964030	-0.9289520	0.1395040
C	-1.8221690	0.1943280	0.1601280
C	-1.4142160	1.5403990	0.1943180
C	-0.0486790	1.8516420	0.1198800
C	1.0337140	0.9597900	0.0678290
C	2.3830390	1.5189130	-0.2266610
C	2.5510080	2.4038850	-1.3135980
C	3.7795850	2.9914120	-1.6167160
C	4.9050510	2.7006580	-0.8406770
C	4.7824480	1.8276830	0.2416680
C	3.5415700	1.2689050	0.5188180
N	1.6717160	-1.6117690	-0.9656370
C	2.3077180	-2.8189700	-0.4438780
C	2.5249220	-2.5479750	1.0518090
C	-1.5927850	-2.2879620	-0.0123590
C	-1.4070130	-3.3208720	0.9279960
C	-1.9883380	-4.5805020	0.7686270
C	-2.7864110	-4.8492510	-0.3465910
C	-2.9937640	-3.8520340	-1.3017120
C	-2.3961670	-2.6108100	-1.1177800
F	-2.6025600	-1.6723930	-2.0830620

C	-2.4300730	2.6225940	0.2743930
C	-3.5818350	2.4806800	1.0725830
C	-4.5465040	3.4878060	1.1417930
C	-4.3816150	4.6753170	0.4235330
C	-3.2414730	4.8374400	-0.3692220
C	-2.2847210	3.8241470	-0.4465210
F	3.4652720	0.4461970	1.6073980
C	1.2577420	-1.3525010	2.8512790
C	1.3469480	-1.5753110	-2.3834390
H	-5.4228510	3.3484630	1.7707670
H	-3.7117110	1.5780940	1.6646620
H	-1.4238040	3.9569200	-1.0969510
H	-3.1025280	5.7507650	-0.9433580
H	-5.1295860	5.4620770	0.4799750
H	0.2185490	2.9072840	0.1102910
H	-2.8930270	0.0000710	0.1253380
H	3.4855070	-2.0410660	1.2283620
H	2.5181570	-3.4816480	1.6292590
H	1.6662530	-3.7027360	-0.5952790
H	3.2596020	-2.9952050	-0.9607620
H	-0.7992560	-3.1152990	1.8022630
H	-1.8264020	-5.3470000	1.5219600
H	-3.2480210	-5.8246280	-0.4745230
H	-3.6028970	-4.0201250	-2.1847170
H	5.6256510	1.5844080	0.8811370
H	5.8685100	3.1472030	-1.0703830
H	3.8586920	3.6655260	-2.4654070
H	1.6858530	2.6164610	-1.9357140
H	2.2681200	-1.6416780	-2.9758700
H	0.6788760	-2.3998210	-2.6775750
H	0.8524380	-0.6305330	-2.6258540

H	0.3798840	-0.7120260	2.9825620
H	1.1085720	-2.2604380	3.4520780
H	2.1362250	-0.8176960	3.2367960

10_m (B3LYP/6-31+G*)

N	1.3972020	-1.8410960	-1.2220300
P	0.5583000	-0.9214770	-0.0633000
C	1.1851850	0.7079250	0.0642130
C	0.3348490	1.8125030	0.0242990
C	-1.0658950	1.7961620	-0.1154420
C	-1.7397820	0.5680360	-0.1979020
C	-1.1733240	-0.7118370	-0.1890430
C	-2.0527630	-1.9137030	-0.2271510
C	-1.9780310	-2.8850710	-1.2431250
C	-2.8134260	-4.0037210	-1.2633840
C	-3.7659330	-4.1841870	-0.2569270
C	-3.8714740	-3.2423340	0.7688130
C	-3.0194560	-2.1448690	0.7629390
N	0.9593830	-2.0408910	1.1547420
C	1.9994790	-2.9891630	0.7713190
C	1.8563370	-3.1426660	-0.7495620
C	2.6451850	0.9075900	0.3230380
C	3.1259680	1.1549570	1.6226390
C	4.4817710	1.3659710	1.8833120
C	5.4057000	1.3318900	0.8351070
C	4.9653470	1.0990000	-0.4692460
C	3.6078900	0.8971460	-0.6934440
F	3.2109690	0.7043690	-1.9812940
C	-1.8244440	3.0733520	-0.1586260
C	-1.3234070	4.1964460	-0.8454230
C	-2.0295010	5.4005380	-0.8791800

C	-3.2657410	5.5136250	-0.2367490
C	-3.7820280	4.4072120	0.4444780
C	-3.0688070	3.2079510	0.4878990
F	-3.1348240	-1.2600970	1.7938470
C	1.2921280	-1.6194320	-2.6530510
C	0.7743620	-1.7541910	2.5672490
H	-1.6179350	6.2483500	-1.4222920
H	-0.3802080	4.1175560	-1.3805410
H	-3.4700980	2.3700800	1.0525870
H	-4.7378670	4.4810620	0.9582960
H	-3.8184560	6.4492300	-0.2666510
H	-2.8232580	0.6015680	-0.2995660
H	0.8153070	2.7841200	0.1356430
H	1.1333890	-3.9372290	-0.9983860
H	2.8156280	-3.3972540	-1.2180110
H	3.0024230	-2.6143040	1.0360450
H	1.8463850	-3.9460520	1.2862920
H	2.4059730	1.1847330	2.4362330
H	4.8140010	1.5571580	2.9001240
H	6.4631920	1.4932820	1.0263610
H	5.6507780	1.0814070	-1.3111920
H	-4.5939310	-3.3478110	1.5725400
H	-4.4254500	-5.0476720	-0.2679500
H	-2.7287250	-4.7253410	-2.0716280
H	-1.2607080	-2.7396470	-2.0447880
H	0.4954180	-2.6728840	3.0989200
H	1.6851450	-1.3452340	3.0326440
H	-0.0377630	-1.0321800	2.6939950
H	0.8950610	-0.6194490	-2.8460910
H	2.2825600	-1.6832740	-3.1201090
H	0.6321100	-2.3573860	-3.1372440

10_n (B3LYP/6-31+G*)

N	-0.3197070	-2.3043100	1.1687510
P	-0.0011500	-1.1102440	0.0000110
C	-1.3749320	-0.0372220	-0.1548220
C	-1.2072900	1.3495800	-0.1252270
C	0.0021420	2.0540850	-0.0000330
C	1.2101150	1.3470790	0.1250560
C	1.3749050	-0.0400780	0.1545770
C	2.7391000	-0.6312720	0.2520830
C	3.1313390	-1.4555370	1.3233960
C	4.4130660	-2.0034470	1.4068650
C	5.3549060	-1.7332560	0.4103160
C	5.0037640	-0.9187920	-0.6684590
C	3.7172510	-0.3965160	-0.7249440
N	0.3148420	-2.3053350	-1.1683800
C	-0.0415390	-3.6611520	-0.7674340
C	0.0333010	-3.6610840	0.7680780
C	-2.7403420	-0.6256200	-0.2524230
C	-3.1344890	-1.4483560	-1.3242070
C	-4.4173480	-1.9936070	-1.4077460
C	-5.3584010	-1.7221820	-0.4107900
C	-5.0053680	-0.9091590	0.6684540
C	-3.7177820	-0.3895390	0.7249890
F	-3.4001750	0.3788890	1.8042490
C	0.0036580	3.5400460	0.0000490
C	-0.8522910	4.2719860	-0.8458320
C	-0.8561350	5.6680190	-0.8419830
C	0.0065260	6.3755040	0.0001950
C	0.8677550	5.6661860	0.8423000
C	0.8610850	4.2701610	0.8460060

F	3.4014760	0.3732820	-1.8037660
C	-0.4921170	-2.0118730	2.5797880
C	0.4879660	-2.0136390	-2.5794850
H	-1.5258580	6.2039830	-1.5107740
H	-1.5059140	3.7405960	-1.5332820
H	1.5136290	3.7373800	1.5334040
H	1.5385590	6.2007230	1.5111490
H	0.0076250	7.4625460	0.0002550
H	2.1255090	1.9334300	0.1880670
H	-2.1214710	1.9378220	-0.1882580
H	1.0448190	-3.9328040	1.1123910
H	-0.6762370	-4.3777820	1.2010500
H	-1.0537210	-3.9304150	-1.1117230
H	0.6662210	-4.3796960	-1.2002530
H	-2.4163170	-1.6429800	-2.1154610
H	-4.6843860	-2.6177040	-2.2564580
H	-6.3617420	-2.1350290	-0.4705890
H	-5.7055910	-0.6776920	1.4654080
H	5.7046360	-0.6882060	-1.4650990
H	6.3573950	-2.1481720	0.4700680
H	4.6786350	-2.6286600	2.2552170
H	2.4125720	-1.6492470	2.1143300
H	1.2950220	-2.6310620	-2.9949120
H	-0.4272990	-2.2076950	-3.1626030
H	0.7647800	-0.9634010	-2.7095410
H	-0.7662920	-0.9609140	2.7096080
H	-1.3007160	-2.6271740	2.9953580
H	0.4226630	-2.2080920	3.1629460

10_o (B3LYP/6-31+G*)

N	0.7878900	-2.2112050	-1.4043330

P	0.3161150	-1.0795320	-0.2192290
C	1.3395880	0.3402750	-0.2082020
C	0.7831860	1.6213290	-0.2309900
C	-0.5826900	1.9497850	-0.2804310
C	-1.5459910	0.9283740	-0.2634550
C	-1.3160570	-0.4511730	-0.2351290
C	-2.4620100	-1.3974940	-0.1429630
C	-2.6881080	-2.4229540	-1.0805180
C	-3.7662470	-3.3036200	-0.9735710
C	-4.6681180	-3.1801260	0.0866280
C	-4.4797270	-2.1743880	1.0369600
C	-3.3925340	-1.3195260	0.9049250
N	0.5360510	-2.2334290	1.0110480
C	1.2613570	-3.4257180	0.5836920
C	0.9443110	-3.5756150	-0.9113920
C	2.8138140	0.1680570	-0.0582620
C	3.6111090	-0.4732210	-1.0262450
C	4.9917480	-0.6155250	-0.8721110
C	5.6249650	-0.1111710	0.2671700
C	4.8688370	0.5308580	1.2499550
C	3.4959880	0.6513510	1.0691500
F	2.7879600	1.2622770	2.0583330
C	-1.0048910	3.3742700	-0.3283090
C	-0.3007430	4.3190300	-1.0994500
C	-0.6899240	5.6594890	-1.1370100
C	-1.8041220	6.0923070	-0.4125550
C	-2.5186410	5.1671660	0.3544930
C	-2.1208820	3.8298970	0.4004580
F	-3.2294940	-0.3664210	1.8652210
C	0.6306240	-1.9943680	-2.8292120
C	0.6266170	-1.8440920	2.4109840

H	-0.1277950	6.3644990	-1.7453500
H	0.5494980	3.9946170	-1.6946110
H	-2.6669740	3.1330880	1.0314810
H	-3.3810500	5.4898940	0.9334320
H	-2.1104820	7.1348560	-0.4440770
H	-2.5912450	1.2308120	-0.2919370
H	1.4919310	2.4469200	-0.1853400
H	0.0246970	-4.1645340	-1.0622450
H	1.7614950	-4.0795220	-1.4437290
H	2.3465560	-3.3138280	0.7446100
H	0.9204520	-4.2993580	1.1534730
H	3.1279970	-0.8523020	-1.9207100
H	5.5725070	-1.1094770	-1.6466650
H	6.6997290	-0.2114230	0.3922350
H	5.3216590	0.9330600	2.1511500
H	-5.1535250	-2.0470050	1.8788560
H	-5.5143310	-3.8559500	0.1750150
H	-3.9081920	-4.0759720	-1.7249100
H	-2.0095580	-2.5114730	-1.9230060
H	0.2245160	-2.6462990	3.0421370
H	1.6628730	-1.6376890	2.7202990
H	0.0295180	-0.9443470	2.5834410
H	0.4977080	-0.9268110	-3.0300580
H	1.5253110	-2.3363820	-3.3681850
H	-0.2375410	-2.5318370	-3.2435310

10_p (B3LYP/6-31+G*)

N	0.1367820	-2.2262380	-1.2077860
P	0.0003250	-1.0340300	-0.0000420
C	1.3791020	0.0398010	0.0536670
C	1.2133680	1.4262180	0.0562950

C	-0.0006640	2.1342350	-0.0000770
C	-1.2142580	1.4254620	-0.0564280
C	-1.3791230	0.0389450	-0.0537870
C	-2.7546240	-0.5447260	-0.1258090
C	-3.4333370	-0.6735070	-1.3515440
C	-4.7257730	-1.1975620	-1.4308210
C	-5.3805150	-1.6135750	-0.2682480
C	-4.7421040	-1.4932180	0.9677720
C	-3.4569110	-0.9642870	1.0106210
N	-0.1354050	-2.2262510	1.2077720
C	0.1719990	-3.5765500	0.7475890
C	-0.1697820	-3.5766970	-0.7475210
C	2.7549620	-0.5430080	0.1257930
C	3.4337270	-0.6711710	1.3515670
C	4.7264920	-1.1943960	1.4309610
C	5.3815240	-1.6101780	0.2684690
C	4.7430630	-1.4904270	-0.9675840
C	3.4575350	-0.9623170	-1.0105500
F	2.8763210	-0.8338710	-2.2341580
C	-0.0011220	3.6196660	-0.0000820
C	0.8989690	4.3518310	0.7990070
C	0.9036320	5.7478550	0.7950060
C	-0.0020410	6.4562500	-0.0000370
C	-0.9072670	5.7472920	-0.7950890
C	-0.9017070	4.3512710	-0.7991270
F	-2.8757270	-0.8353320	2.2341890
C	-0.0528640	-1.9320950	-2.6190210
C	0.0540930	-1.9318910	2.6189800
H	1.6082130	6.2833110	1.4274640
H	1.5879480	3.8200540	1.4506880
H	-1.5903730	3.8190670	-1.4507910

H	-1.6122060	6.2823090	-1.4275180
H	-0.0023900	7.5432840	-0.0000210
H	-2.1341330	2.0091310	-0.0727710
H	2.1328770	2.0104590	0.0726750
H	-1.2331620	-3.8216070	-0.9084780
H	0.4343900	-4.3101580	-1.2966900
H	1.2355280	-3.8207980	0.9085610
H	-0.4317190	-4.3103630	1.2967910
H	2.9243430	-0.3442100	2.2542130
H	5.2194330	-1.2751500	2.3960410
H	6.3871740	-2.0190430	0.3180860
H	5.2255370	-1.7893590	-1.8932520
H	-5.2243680	-1.7923120	1.8934970
H	-6.3859090	-2.0230780	-0.3177740
H	-5.2186810	-1.2787930	-2.3958780
H	-2.9241850	-0.3463740	-2.2542580
H	-0.6337180	-2.5377360	3.2215220
H	1.0850810	-2.1391730	2.9466500
H	-0.1725200	-0.8802090	2.8118370
H	0.1730350	-0.8802680	-2.8119300
H	0.6353910	-2.5375000	-3.2214970
H	-1.0836930	-2.1401040	-2.9467300

10_q (B3LYP/6-31+G*)

N	-0.0451180	-2.3356810	-1.4179790
P	-0.0697220	-1.1182660	-0.2240720
C	1.3649080	-0.1165330	-0.2487820
C	1.2595340	1.2774220	-0.2609580
C	0.0784980	2.0381720	-0.2739050
C	-1.1680400	1.3912080	-0.2257640
C	-1.4018520	0.0137940	-0.1985670

C	-2.7905060	-0.5070090	-0.0448030
C	-3.4219210	-1.2963420	-1.0234030
C	-4.7240720	-1.7751670	-0.8631250
C	-5.4435450	-1.4679570	0.2948650
C	-4.8516380	-0.6852610	1.2884770
C	-3.5517060	-0.2314610	1.1004420
N	-0.2071220	-2.2862710	1.0058670
C	0.0615940	-3.6498500	0.5596850
C	-0.3406590	-3.6757160	-0.9215610
C	2.7067240	-0.7611510	-0.1641230
C	3.1960790	-1.6512460	-1.1403470
C	4.4606770	-2.2354180	-1.0425930
C	5.2868170	-1.9411710	0.0455440
C	4.8381530	-1.0631860	1.0343430
C	3.5726670	-0.5026410	0.9103480
F	3.1614140	0.3325870	1.9050550
C	0.1465080	3.5221940	-0.3190650
C	1.1142480	4.2316830	0.4185940
C	1.1808950	5.6253070	0.3732400
C	0.2728520	6.3524800	-0.4022610
C	-0.6989080	5.6656030	-1.1354410
C	-0.7563750	4.2709470	-1.0983940
F	-2.9972710	0.5094740	2.0986670
C	-0.1891760	-2.0687410	-2.8359210
C	0.0738140	-1.9589080	2.3968310
H	1.9358220	6.1450820	0.9588760
H	1.8052230	3.6857180	1.0560800
H	-1.4980680	3.7522790	-1.7010700
H	-1.4077360	6.2156450	-1.7503680
H	0.3213200	7.4380310	-0.4337510
H	-2.0543850	2.0220180	-0.1752460

H	2.2021020	1.8217700	-0.2883460
H	-1.4100320	-3.9176780	-1.0355360
H	0.2386580	-4.4231620	-1.4791370
H	1.1270660	-3.9071990	0.6805660
H	-0.5302460	-4.3631930	1.1470060
H	2.5675030	-1.8706170	-1.9967460
H	4.8040190	-2.9100990	-1.8224230
H	6.2749790	-2.3859780	0.1258200
H	5.4470400	-0.8109050	1.8972490
H	-5.3754650	-0.4279380	2.2041200
H	-6.4596910	-1.8298050	0.4263540
H	-5.1789540	-2.3757300	-1.6464050
H	-2.8782700	-1.5176100	-1.9368510
H	-0.5461900	-2.5819320	3.0533130
H	1.1319480	-2.1182670	2.6568860
H	-0.1756510	-0.9117330	2.5885190
H	0.0304270	-1.0162710	-3.0404200
H	0.5146920	-2.6841240	-3.4136990
H	-1.2053100	-2.2855100	-3.2025700

10_r (B3LYP/6-31+G*)

N	1.3819890	-1.8429270	-1.2164330
P	0.5550420	-0.9269650	-0.0441730
C	1.1911440	0.6993020	0.0687770
C	0.3469520	1.8083690	0.0331210
C	-1.0558930	1.7989610	-0.0841860
C	-1.7371410	0.5735360	-0.1523150
C	-1.1774090	-0.7090540	-0.1458510
C	-2.0626400	-1.9077170	-0.1492540
C	-2.0194170	-2.8829940	-1.1634410
C	-2.8582900	-3.9992160	-1.1560210

C	-3.7831990	-4.1722520	-0.1228380
C	-3.8568160	-3.2262860	0.9018290
C	-3.0013430	-2.1316810	0.8687210
N	0.9626250	-2.0545490	1.1639530
C	1.9894050	-3.0106540	0.7645620
C	1.8317570	-3.1522670	-0.7559170
C	2.6646520	0.8917920	0.2437670
C	3.2355250	1.0417600	1.5209790
C	4.6066010	1.2400740	1.7003340
C	5.4530330	1.2940990	0.5894830
C	4.9217160	1.1582820	-0.6946710
C	3.5523160	0.9629660	-0.8363920
F	3.0643710	0.8552230	-2.1023120
C	-1.8109980	3.0778630	-0.1262460
C	-1.4340260	4.1786290	0.6680080
C	-2.1405030	5.3818080	0.6233680
C	-3.2554460	5.5171660	-0.2090050
C	-3.6485930	4.4338090	-0.9999100
C	-2.9328060	3.2357140	-0.9634840
F	-3.0832140	-1.2441220	1.8991020
C	1.2418010	-1.6241750	-2.6454090
C	0.7909030	-1.7785430	2.5801140
H	-1.8265660	6.2113840	1.2529290
H	-0.5911810	4.0831120	1.3481470
H	-3.2333130	2.4169020	-1.6125240
H	-4.5080700	4.5248660	-1.6602870
H	-3.8090360	6.4521610	-0.2407520
H	-2.8246620	0.6121100	-0.1903430
H	0.8401100	2.7787480	0.0771110
H	1.0981940	-3.9375070	-1.0034590
H	2.7841070	-3.4142680	-1.2343290

H	2.9983170	-2.6481370	1.0234150
H	1.8308200	-3.9696730	1.2739490
H	2.5747930	1.0069260	2.3831940
H	5.0105800	1.3550150	2.7025680
H	6.5211420	1.4483780	0.7171000
H	5.5460650	1.2080180	-1.5816190
H	-4.5570160	-3.3260780	1.7257220
H	-4.4456420	-5.0335120	-0.1120030
H	-2.7981410	-4.7244920	-1.9632000
H	-1.3235290	-2.7433860	-1.9850570
H	0.4998600	-2.6973380	3.1051450
H	1.7117150	-1.3903510	3.0439190
H	-0.0075410	-1.0438500	2.7195960
H	0.8544600	-0.6192610	-2.8313040
H	2.2187650	-1.7037700	-3.1378910
H	0.5597160	-2.3548690	-3.1098980

10_ω_TS_a (B3LYP/6-31+G*)

N	-0.9022990	-2.1088700	1.0048530
P	-0.4166860	-1.0364740	-0.2240250
C	-1.3306330	0.4528130	-0.1928660
C	-0.6848700	1.6902690	-0.2173290
C	0.7014340	1.9195720	-0.2708060
C	1.5876180	0.8304510	-0.2679410
C	1.2585680	-0.5294670	-0.2560370
C	2.3371880	-1.5547160	-0.1769330
C	2.4766220	-2.6017960	-1.1100450
C	3.4994310	-3.5477510	-1.0164170
C	4.4306410	-3.4740930	0.0228700
C	4.3273980	-2.4513080	0.9679880

C	3.2948500	-1.5291280	0.8507630
N	-0.7888170	-2.1926660	-1.4211350
C	-1.4896620	-3.3725070	-0.9249460
C	-1.0866070	-3.4844200	0.5522570
C	-2.8143760	0.3851110	-0.0416690
C	-3.6655170	-0.0310890	-1.0807000
C	-5.0529810	-0.0813100	-0.9257530
C	-5.6330060	0.2931880	0.2893210
C	-4.8200870	0.7131820	1.3443990
C	-3.4432260	0.7453390	1.1579520
F	-2.6768050	1.1430940	2.2097810
C	1.2261800	3.3096100	-0.3079760
C	0.5904530	4.3106070	-1.0676920
C	1.0777290	5.6188670	-1.0961570
C	2.2242510	5.9618220	-0.3739830
C	2.8714880	4.9799050	0.3820810
C	2.3757050	3.6755180	0.4193960
F	3.2160250	-0.5610100	1.8065860
C	-0.5130470	-1.8977170	2.3923130
C	-0.8607790	-1.8827990	-2.8361260
H	0.5668150	6.3687490	-1.6959570
H	-0.2836350	4.0552460	-1.6619580
H	2.8709230	2.9351030	1.0426170
H	3.7580270	5.2328370	0.9592790
H	2.6070340	6.9789850	-0.3988270
H	2.6516870	1.0570720	-0.3026340
H	-1.3335770	2.5634500	-0.1598370
H	-0.1577820	-4.0684880	0.6624360
H	-1.8699280	-3.9751240	1.1436460
H	-2.5815470	-3.2618710	-1.0287800
H	-1.1827200	-4.2622140	-1.4902700

H	-3.2199740	-0.3015030	-2.0335780
H	-5.6785540	-0.4009900	-1.7549230
H	-6.7115900	0.2636880	0.4183340
H	-5.2331760	1.0101160	2.3036620
H	5.0264530	-2.3601920	1.7938330
H	5.2339540	-4.2018350	0.0989690
H	3.5750530	-4.3338950	-1.7631110
H	1.7683420	-2.6565770	-1.9293750
H	-0.3899450	-2.6814480	-3.4265590
H	-1.8986930	-1.7707940	-3.1878070
H	-0.3273570	-0.9491290	-3.0399260
H	-0.4210480	-0.8267380	2.5916540
H	-1.2875600	-2.3009910	3.0562860
H	0.4453330	-2.3832620	2.6340780

10_ω_TS_b (B3LYP/6-31+G*)

N	-1.1564300	-1.9907800	-1.1580310
P	-0.5193680	-1.0023170	0.0766900
C	1.2212520	-0.7205780	-0.0158290
C	1.6682020	0.6182120	0.0378070
C	0.9142780	1.7937480	0.1340300
C	-0.4891950	1.7387550	0.1973490
C	-1.2585200	0.5819270	0.2006880
C	-2.7463080	0.6720700	0.2534320
C	-3.5086400	0.1422830	1.3131310
C	-4.8998020	0.2557500	1.3517120
C	-5.5783340	0.9135100	0.3217270
C	-4.8574680	1.4547760	-0.7446070
C	-3.4741170	1.3208670	-0.7555860
N	-1.0895870	-2.0728730	1.2748550
C	-1.7764760	-3.2570630	0.7753200

C	-2.1634770	-2.9358240	-0.6792410
C	2.1670050	-1.8661110	-0.1394280
C	1.7121830	-3.2093230	-0.1228520
C	2.5524470	-4.3157720	-0.2194680
C	3.9319620	-4.1458110	-0.3449730
C	4.4347240	-2.8471820	-0.3746080
C	3.5710240	-1.7635110	-0.2753460
F	4.1909730	-0.5440660	-0.3228360
C	1.6033800	3.1120470	0.1714130
C	2.8043250	3.2914830	0.8843970
C	3.4511290	4.5283690	0.9149670
C	2.9087610	5.6269890	0.2420150
C	1.7146500	5.4687530	-0.4672400
C	1.0750940	4.2282800	-0.5059110
F	-2.8048250	1.8468230	-1.8192160
C	-1.2963140	-1.4987550	-2.5216840
C	-0.5535360	-2.1069240	2.6244360
H	4.3754510	4.6359350	1.4780140
H	3.2258370	2.4569510	1.4391150
H	0.1656390	4.1170630	-1.0913030
H	1.2849010	6.3103840	-1.0057920
H	3.4094930	6.5914960	0.2690350
H	-1.0298190	2.6796430	0.2852870
H	2.7363450	0.7591520	-0.0276460
H	-3.1741550	-2.4987730	-0.7284100
H	-2.1572020	-3.8404210	-1.3010250
H	-1.1233250	-4.1449610	0.8277420
H	-2.6665140	-3.4691500	1.3828220
H	0.6515460	-3.3940180	-0.0528620
H	2.1204180	-5.3130210	-0.2004800
H	4.6015880	-4.9974080	-0.4226830

H	5.4966900	-2.6445010	-0.4754850
H	-5.3464700	1.9728950	-1.5639770
H	-6.6601950	1.0117650	0.3473260
H	-5.4512290	-0.1579990	2.1918770
H	-2.9840230	-0.3541130	2.1227200
H	-1.3630800	-2.2558410	3.3520570
H	0.1824510	-2.9160870	2.7522910
H	-0.0599900	-1.1577090	2.8530890
H	-0.4987680	-0.7831790	-2.7403400
H	-1.2038780	-2.3366470	-3.2237790
H	-2.2637740	-1.0017160	-2.6911310

10_ω_TS_c (B3LYP/6-31+G*)

N	-1.0423560	-2.0132300	-1.3169280
P	-0.4960130	-0.9625460	-0.0956790
C	1.2153470	-0.6121700	-0.1976240
C	1.6736410	0.7102160	-0.1958110
C	0.8979300	1.8770830	-0.1163930
C	-0.4981390	1.7758690	0.0320940
C	-1.2553100	0.6048330	0.0783600
C	-2.7198480	0.6885020	0.3712470
C	-3.1874490	0.8835990	1.6842600
C	-4.5481840	0.9936870	1.9764270
C	-5.4916400	0.9051840	0.9485260
C	-5.0658910	0.7172040	-0.3675930
C	-3.7024120	0.6170550	-0.6215330
N	-1.0063050	-2.0490390	1.1132200
C	-1.3857590	-3.3649450	0.6076460
C	-1.8529610	-3.1259600	-0.8348680
C	2.1909050	-1.7382770	-0.2103000
C	2.1877870	-2.7501960	-1.1913810

C	3.1180430	-3.7916120	-1.1849410
C	4.0962270	-3.8532640	-0.1888610
C	4.1324430	-2.8698090	0.8020070
C	3.1886820	-1.8505690	0.7716960
F	3.2418620	-0.9247390	1.7710960
C	1.5457980	3.2135610	-0.1655760
C	2.7808530	3.4533110	0.4685830
C	3.3904040	4.7080510	0.4163640
C	2.7765930	5.7659780	-0.2610540
C	1.5477160	5.5486070	-0.8905730
C	0.9455300	4.2895730	-0.8482780
F	-3.3189320	0.4553800	-1.9203350
C	-1.1345140	-1.6411400	-2.7190700
C	-0.4899000	-1.9753180	2.4711630
H	4.3418290	4.8628280	0.9202850
H	3.2567760	2.6533880	1.0303430
H	0.0072160	4.1310020	-1.3742350
H	1.0611670	6.3577860	-1.4306130
H	3.2489680	6.7443730	-0.2978500
H	-1.0550630	2.7035990	0.1590370
H	2.7508150	0.8351630	-0.2912880
H	-2.9277780	-2.8832220	-0.8693730
H	-1.6869420	-4.0138510	-1.4587480
H	-0.5303950	-4.0608170	0.6319240
H	-2.1887410	-3.7901740	1.2232140
H	1.4427040	-2.7005400	-1.9777610
H	3.0854960	-4.5467830	-1.9659050
H	4.8285950	-4.6559440	-0.1809780
H	4.8718820	-2.8827480	1.5970540
H	-5.7667870	0.6577480	-1.1947630
H	-6.5532990	0.9878860	1.1651650

H	-4.8702390	1.1467980	3.0029010
H	-2.4532780	0.9511010	2.4825810
H	-1.2656800	-2.2875670	3.1819550
H	0.3942980	-2.6163430	2.6135510
H	-0.2051430	-0.9456820	2.7057300
H	-0.5288320	-0.7493900	-2.9057380
H	-0.7514820	-2.4555800	-3.3503420
H	-2.1669880	-1.4190520	-3.0189520

10_ω_TS_d (B3LYP/6-31+G*)

N	-0.9787580	-1.9157500	-1.2056490
P	-0.3711610	-0.9787600	0.0664890
C	1.3473840	-0.5672630	0.0027680
C	1.6729700	0.8105440	0.0407130
C	0.8473440	1.9300140	0.1403080
C	-0.5510870	1.7725280	0.2242700
C	-1.2169070	0.5609270	0.2325480
C	-2.7076570	0.5329870	0.2985420
C	-3.4158710	-0.0329420	1.3766300
C	-4.8110720	-0.0240050	1.4300970
C	-5.5486740	0.5591680	0.3957610
C	-4.8826600	1.1326450	-0.6890470
C	-3.4931450	1.1047510	-0.7132360
N	-0.9213660	-2.0839320	1.2295910
C	-1.5718350	-3.2755240	0.6910050
C	-1.9053550	-2.9608620	-0.7816240
C	2.4718440	-1.5519680	-0.1141690
C	3.8300300	-1.1319220	-0.1300040
C	4.9087600	-2.0044400	-0.2450200
C	4.7036630	-3.3818040	-0.3499060
C	3.3935160	-3.8501510	-0.3298530

C	2.3416980	-2.9497160	-0.2119600
F	1.1009640	-3.5195220	-0.1850130
C	1.4418270	3.2928460	0.1549480
C	2.6233920	3.5710310	0.8692890
C	3.1866140	4.8487100	0.8703880
C	2.5749360	5.8910460	0.1680970
C	1.3955300	5.6362350	-0.5380050
C	0.8405340	4.3552300	-0.5478240
F	-2.8777010	1.6630010	-1.7936560
C	-1.1134290	-1.4000240	-2.5580560
C	-0.3644140	-2.1632930	2.5678480
H	4.0971970	5.0332640	1.4359410
H	3.0912360	2.7822660	1.4536920
H	-0.0585130	4.1688400	-1.1301740
H	0.9114640	6.4344560	-1.0961340
H	3.0094710	6.8874460	0.1727590
H	-1.1597120	2.6701300	0.3202920
H	2.7241860	1.0520430	-0.0493020
H	-2.9479060	-2.6174940	-0.8823380
H	-1.7823320	-3.8524050	-1.4103320
H	-0.9090490	-4.1472620	0.7704940
H	-2.4868960	-3.4924340	1.2588800
H	4.0683040	-0.0801470	-0.0486750
H	5.9162530	-1.5969620	-0.2512890
H	5.5368880	-4.0723180	-0.4414780
H	3.1574480	-4.9076080	-0.4019210
H	-5.4182910	1.5961840	-1.5119590
H	-6.6346030	0.5746470	0.4321700
H	-5.3204210	-0.4624250	2.2841790
H	-2.8462880	-0.4736400	2.1877340
H	-1.1597290	-2.3667310	3.2982630

H	0.3919400	-2.9595850	2.6499660
H	0.1066150	-1.2123310	2.8345240
H	-0.3822640	-0.6042210	-2.7266220
H	-0.9163220	-2.2023850	-3.2808230
H	-2.1173760	-0.9919600	-2.7518000

10_θ_TS_a (B3LYP/6-31+G*)

N	0.1860070	2.3165230	-1.2051320
P	0.0003010	1.1212570	0.0001820
C	-1.3731840	0.0586390	-0.2047450
C	-1.2058230	-1.3316060	-0.1651750
C	-0.0004670	-2.0271290	0.0000000
C	1.2052530	-1.3322220	0.1650660
C	1.3733140	0.0579410	0.2046630
C	2.7345890	0.6468360	0.3371900
C	3.0750010	1.5659210	1.3507690
C	4.3593320	2.1022030	1.4636300
C	5.3583080	1.7287910	0.5605870
C	5.0604500	0.8216200	-0.4586130
C	3.7713350	0.3114300	-0.5491020
N	-0.1848380	2.3161020	1.2060410
C	-0.3610370	3.6655620	0.6770960
C	0.3623910	3.6657600	-0.6756830
C	-2.7341470	0.6482060	-0.3375310
C	-3.0739380	1.5673130	-1.3512930
C	-4.3579820	2.1042210	-1.4644320
C	-5.3572810	1.7314400	-0.5614830
C	-5.0600380	0.8242630	0.4578910
C	-3.7711910	0.3134400	0.5486480
F	-3.5152460	-0.5481790	1.5733290
C	-0.0008720	-3.5264040	0.0000000

C	0.1512090	-4.2478390	-1.1955230
C	0.1507130	-5.6458090	-1.1978750
C	-0.0016930	-6.3494790	0.0001060
C	-0.1536880	-5.6456510	1.1980450
C	-0.1533710	-4.2476800	1.1956120
F	3.5148040	-0.5501890	-1.5736370
C	0.7981080	2.0131780	-2.4902700
C	-0.7971160	2.0123470	2.4910060
H	0.2705120	-6.1843280	-2.1351650
H	0.2724430	-3.7038040	-2.1293260
H	-0.2742950	-3.7035240	2.1293850
H	-0.2738090	-6.1840460	2.1353660
H	-0.0020170	-7.4368170	0.0001380
H	2.1088350	-1.9289760	0.2812820
H	-2.1096890	-1.9279080	-0.2815170
H	1.4315290	3.9040980	-0.5493700
H	-0.0728180	4.4052280	-1.3603990
H	-1.4301390	3.9041010	0.5508530
H	0.0742690	4.4047160	1.3620880
H	-2.3066530	1.8499700	-2.0644110
H	-4.5797390	2.8034850	-2.2664090
H	-6.3613230	2.1379500	-0.6476160
H	-5.8047740	0.5121580	1.1838710
H	5.8049150	0.5090410	-1.1846670
H	6.3625700	2.1348030	0.6465110
H	4.5815610	2.8014700	2.2654740
H	2.3079690	1.8490800	2.0639620
H	-0.3345050	2.6222170	3.2782210
H	-1.8805560	2.2078660	2.4913150
H	-0.6408730	0.9583160	2.7379130
H	0.6416870	0.9592710	-2.7375850

H	0.3355280	2.6234310	-3.2772090
H	1.8815790	2.2085380	-2.4906000

10_θ_TS_b (B3LYP/6-31+G*)

N	-0.1735110	2.3270400	1.2079540
P	-0.0001610	1.1363350	0.0000000
C	1.3673210	0.0656430	0.1911020
C	1.1994430	-1.3205440	0.1507820
C	0.0003240	-2.0423420	0.0000000
C	-1.1990140	-1.3209060	-0.1507920
C	-1.3673040	0.0652270	-0.1910960
C	-2.7325950	0.6499760	-0.3126690
C	-3.0866330	1.5547220	-1.3340810
C	-4.3726810	2.0889480	-1.4366630
C	-5.3584020	1.7283890	-0.5140160
C	-5.0463760	0.8357670	0.5137050
C	-3.7560080	0.3266110	0.5929610
N	0.1728210	2.3272290	-1.2076660
C	0.3535530	3.6773070	-0.6801740
C	-0.3550050	3.6770590	0.6805590
C	2.7324430	0.6508030	0.3125740
C	3.0862590	1.5557520	1.3338830
C	4.3721530	2.0903660	1.4363620
C	5.3579440	1.7300070	0.5137120
C	5.0461370	0.8371970	-0.5139130
C	3.7559160	0.3276570	-0.5930700
F	3.4849280	-0.5214380	-1.6240840
C	0.0005490	-3.5370750	0.0000000
C	1.1881020	-4.2851220	0.1555090
C	1.1900410	-5.6803030	0.1552010
C	0.0009760	-6.3957890	0.0000000

C	-1.1883030	-5.6806590	-0.1551640
C	-1.1867810	-4.2854780	-0.1554790
F	-3.4848110	-0.5223150	1.6240580
C	-0.7693050	2.0224220	2.5010240
C	0.7686670	2.0230380	-2.5008050
H	2.1331500	-6.2081070	0.2780550
H	2.1419860	-3.7846350	0.2803480
H	-2.1408150	-3.7852770	-0.2803140
H	-2.1312550	-6.2087460	-0.2780140
H	0.0011390	-7.4826190	0.0000000
H	-2.1210230	-1.8832080	-0.2577530
H	2.1216260	-1.8825690	0.2576910
H	-1.4252750	3.9157900	0.5664670
H	0.0883220	4.4153190	1.3611200
H	1.4236910	3.9166230	-0.5660730
H	-0.0901900	4.4153740	-1.3606740
H	2.3292270	1.8287510	2.0615810
H	4.6058890	2.7782230	2.2447050
H	6.3631910	2.1352780	0.5909700
H	5.7803670	0.5353000	-1.2547370
H	-5.7805490	0.5337220	1.2545250
H	-6.3637640	2.1333590	-0.5913530
H	-4.6065870	2.7766570	-2.2450830
H	-2.3296560	1.8278680	-2.0617800
H	0.2957880	2.6331210	-3.2815100
H	1.8518350	2.2187040	-2.5145500
H	0.6093090	0.9691150	-2.7458940
H	-0.6090650	0.9686560	2.7462130
H	-0.2970450	2.6329690	3.2817380
H	-1.8526410	2.2171590	2.5146190
